# Biosynthesis of cinchona alkaloids

**DOI:** 10.1038/s41586-026-10227-x

**Published:** 2026-03-18

**Authors:** Blaise Kimbadi Lombe, Tingan Zhou, Gyumin Kang, Joshua C. Wood, John P. Hamilton, Klaus Gase, Yoko Nakamura, Ryan M. Alam, Ron P. Dirks, Lorenzo Caputi, C. Robin Buell, Sarah E. O’Connor

**Affiliations:** 1 Department of Natural Product Biosynthesis, Max Planck Institute for Chemical Ecology, Jena, Germany; 2 Center for Applied Genetic Technologies, University of Georgia, Athens, GA USA; 3 Department of Crop and Soil Sciences, University of Georgia, Athens, GA USA; 4 Future Genomics Technologies, Leiden, The Netherlands; 5 Institute of Plant Breeding, Genetics and Genomics, University of Georgia, Athens, GA USA; 6 The Plant Center, University of Georgia, Athens, GA USA

**Keywords:** Biochemistry, Secondary metabolism

## Abstract

Cinchona alkaloids, which have been studied for more than 250 years, are plant-derived natural products that have collectively had a substantial impact in medicine and basic science^1–5^. Examples of cinchona alkaloids include quinine, a historically important antimalarial drug, and cinchonidine, a chiral catalyst widely used in process chemistry. However, it is still largely unknown how plants synthesize these well-known compounds. Here we report the discovery of genes responsible for the biosynthesis of the distinctive quinoline–quinuclidine scaffold of cinchona alkaloids. A combination of isotopic labelling, gene silencing, single-nucleus RNA sequencing and comparative transcriptomics revealed the involvement of several unexpected biosynthetic transformations. We also describe a previously unreported quaternary amine intermediate that is generated through an unusual enzymatic cyclization. We show that dihydroquini(di)none, dihydrocinchoni(di)none and cinchoni(di)none can be produced when these genes are heterologously expressed in *Nicotiana benthamiana*. Furthermore, we demonstrate that this *N. benthamiana* expression platform can convert non-native fluorinated and chlorinated tryptamine substrates into dihydrocinchoni(di)none analogues, which suggests that these biosynthetic enzymes can be leveraged to produce halogenated cinchona alkaloid derivatives. These discoveries uncover the long-standing mystery of how the cinchona alkaloid scaffold is biosynthesized and highlight prospects for access to these compounds through metabolic engineering approaches.

## Abstract (web summary)

A combination of isotopic labelling, gene silencing, single-nucleus RNA sequencing and comparative transcriptomics reveal the genes that mediate the biosynthesis of cinchona alkaloids in plants.

## Main

Cinchona alkaloids are a group of structurally diverse, nitrogen-containing compounds that are primarily found in *Cinchona* plants. The best known of these alkaloids, quinine (**1a**), is the earliest known naturally occurring treatment for malaria^6^ and is used as a bittering agent in foods and beverages such as tonic water and bitter lemon. Cinchona alkaloids also include quinidine (**2a**), an antiarrhythmic drug that acts through the inhibition of sodium and potassium channels^7–9^, and the non-methoxylated analogues cinchonidine (**1**) and cinchonine (**2**), which are widely used as chiral catalysts for synthetic chemistry^10,11^. Dihydro analogues of these alkaloids (dihydrocinchonidine (**1′**), dihydrocinchonine (**2′**), dihydroquinine (**1a′**) and dihydroquinidine (**2a′**), congeners with a single bond at C-18–C-19) are produced in parallel by plants and display comparable properties to **1**(**a**)–**2**(**a**)^1,12^. Cinchona alkaloids have also been the subject of longstanding investigation in the field of organic chemistry^2,13^. Quinine was isolated in 1820 (ref. ^4^), subjected to structural characterization in 1908 (ref. ^14^) and the chemical structure was fully established in the 1940s^15^. Unsuccessful attempts to synthetize quinine in the 1850s resulted in a serendipitous discovery of mauve, the world’s first synthetic dye^16–18^. A racemic formal synthesis process was eventually accomplished in 1945 (ref. ^19^) and a stereoselective total synthetic route was achieved in 2001 (ref. ^20^). Overall, cinchona alkaloids have had seminal roles in the fields of medicine, organic synthesis and natural product chemistry^1,2^.

**Fig. 1:**
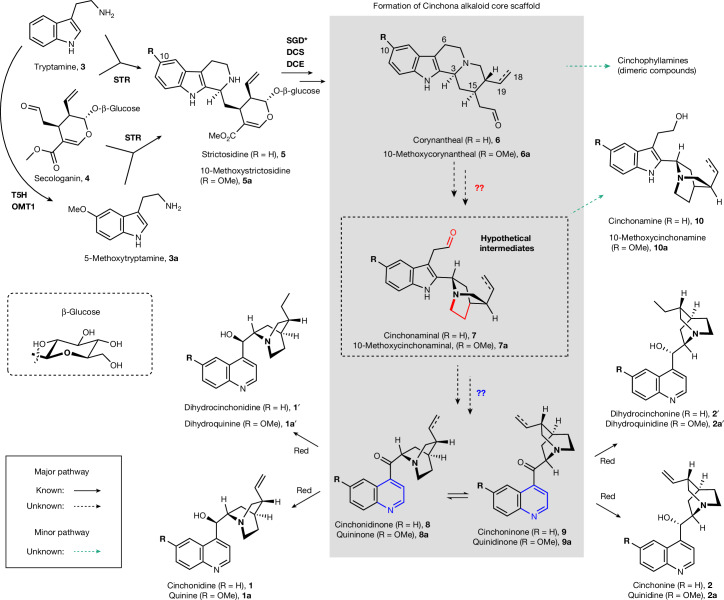
Proposed biosynthetic pathways to cinchona alkaloids. Methoxylated alkaloids and non-methoxylated analogues are produced in a similar manner via parallel routes by the same enzymes. An unknown series of transformations generating the cinchona alkaloid scaffold core is shaded in grey. The dashed double bond at C_18_−C_19_ shown in **6**,**6a**–**10** and **10a** indicates that these compounds also occur in the dihydro form (that is, with a single bond) and are biogenetic precursors to the corresponding alkaloids, for example, **1′**–**2′**. This hypothetical pathway is based on early investigations in plants fed with isotopically labelled precursors. A detailed and updated biosynthetic schematic and a summary of key feeding experiments are given in Supplementary Figs. 1 and 2. The asterisk on SGD indicates that functional characterization of this enzyme is reported here (Supplementary Fig. 3). Red designates previously reported catalytic activity that has been observed in crude samples of *Cinchona* tissues, but the corresponding protein and gene were not reported^40^. For a complete list of compound names, structures and numbers mentioned in this work, see Supplementary Information.

From a biosynthetic perspective, quinine and other cinchona alkaloids are classified as monoterpene indole alkaloids^12,21^. Monoterpene indole alkaloids are natural products that originate from the enzymatic condensation of tryptamine (**3**, derived from tryptophan^22^) and the geraniol-derived monoterpene^23^ secologanin (**4**) to form the tetrahydro-β-carboline intermediate strictosidine (**5**)^21^. This reaction is catalysed by the enzyme strictosidine synthase (STR) (note that throughout, protein and gene symbols relate to *Cinchona pubescens* unless otherwise indicated) (Fig. 1 and Supplementary Fig. 1). Strictosidine, after enzymatic deglucosylation, is acted on by a reductase (dihydrocorynantheine aldehyde synthase (DCS)) and an esterase (dihydrocorynantheine aldehyde esterase (DCE)) to form the dihydro congener of corynantheal (dihydrocorynantheal (**6′**))^24^. Plant feeding studies suggest that both **6′** and corynantheal (**6**) congeners undergo, in parallel, a series of unknown steps to form the hypothetical indole–quinuclidine intermediates cinchonaminal (**7**) and dihydrocinchonaminal (**7′**)^25^, which are thought to undergo conversion, again through a series of unknown chemical transformations, to form the quinoline core of **1**, **1′**, **2** and **2′**. Recent work has shown that the methoxy group observed in **1a**, **1a′**, **2a** and **2a′** is introduced onto the starting precursor tryptamine by tryptamine-5-hydroxylase (T5H) and the *O*-methyltransferase OMT1 (ref. ^26^). Feeding studies have confirmed that the biosynthesis of methoxylated congeners proceeds in the same way as for **1** and **2** via a parallel biosynthetic pathway, with 5-methoxytryptamine (**3a′**) instead of **3** as the indole starting precursor^26^ (Fig. 1 and Supplementary Figs. 1 and 2). However, despite these insights into the biosynthetic process of cinchona alkaloids, the mechanism by which the corynantheal scaffold is converted to these alkaloids has remained elusive.

## Discovery of additional biosynthetic intermediates

**Fig. 2:**
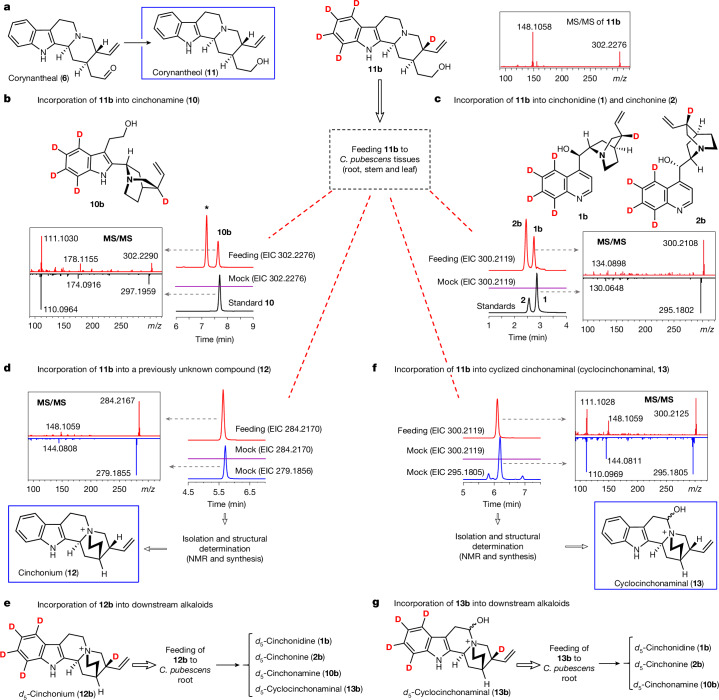
Identification of three key on-pathway intermediates (**11**–**13**) through feeding studies. **a**, Schematic illustrating the natural conversion of **6** into the corresponding alcohol congener **11**, along with the structure of the synthetic isotopically labelled analogue **11b** that was used to feed tissues from *C. pubescens* plantlets. MS/MS (20 to 50 eV) spectrum of the synthetic subtract **11b** is shown. **b**,**c**, Extracted ion chromatograms (EICs) and MS/MS (20 to 50 eV) spectra evidencing the incorporation of **11b** into **1**, **2** and **10**. Labelled compounds elute slightly faster than the non-labelled counterparts (a retention time difference of about 0.05 min). In **b**, the asterisk indicates an unknown compound that is not part of the cinchona alkaloid pathway as evidenced by re-feeding experiments with partially purified material of that labelled compound. Data showing incorporation of **11b** into keto-quinoline intermediates **8** and **9** are provided in Supplementary Fig. 4. **d**–**g**, Identification of a previously unknown compound, **12**, and cyclized cinchonaminal (**13**) as on-pathway intermediates. EIC and MS/MS spectra evidencing the incorporation of **11b** into **12** and **13** are shown. Details on the structural characterization and synthesis of **12** and **13** are provided in the Supplementary Information. EIC and MS/MS spectra demonstrating the incorporation of **12b** and **13b** into downstream alkaloids **1**, **2**, **10** and **13** are similar to the data shown in **b**, **c** and **f** (Supplementary Fig. 5). Mock denotes samples fed with water instead of the labelled compound (control group).

As there is no substantiated hypothesis for how **6** is converted to the hypothetical indole–quinuclidine intermediate **7**, we set out to identify additional on-pathway biosynthetic intermediates. Early experiments showed that **6** was rapidly reduced to the corresponding alcohol corynantheol (**11**) when fed to cell cultures of *Cinchona ledgeneria*^27^. These observations led us to speculate that corynantheol—as well as the dihydro and methoxy corynantheol congeners—could be intermediates of downstream cinchona alkaloids. To test this hypothesis, we chemically synthesized the isotopically labelled analogue *d*_5_-corynantheol (**11b**) and fed it to *C. pubescens* tissues (leaf, stem and root). Liquid chromatography and mass spectrometry (LC–MS) analyses of fed tissues confirmed the formation of both *d*_5_-cinchonidine (**1b**) and *d*_5_-cinchonine (**2b**), and the indole–quinuclidine *d*_5_-cinchonamine (**10b**). This result establishes **11** as an on-pathway intermediate (Fig. 2a–c).

Notably, we also detected efficient isotopic incorporation of **11b** into an unknown metabolite with a pseudomolecular ion at *m/z* 279.1856 (denoted compound **12**) (Fig. 2d and Supplementary Fig. 4). To test whether **12** is also an on-pathway intermediate, we fractionated the isotopically labelled form of this compound from *C. pubescens* tissues that had been fed with **11b**. The fractionated part was then re-fed to fresh *C. pubescens* tissues. Subsequent LC–MS analyses confirmed that this metabolite is a precursor for the alkaloids **1**, **2** and cinchonamine (**10**) (Fig. 2e, and Supplementary Fig. 5). Large-scale isolation, semi-synthesis and NMR analyses established that this major metabolite is a quaternary ammonium, a scaffold previously unreported in the metabolism of *Cinchona*. Therefore we named **12** as cinchonium (Fig. 2d).

Feeding of **11b** and *d*_5_-cinchonium (**12b**) to *C. pubescens* tissues also led to the detection of a native compound with a molecular mass (*m/z* of 295.1805) and a tandem MS (MS/MS) fragmentation pattern similar to commercially available **10** (an alcohol with an *m/z* of 297.1961), which suggested that the detected metabolite was the long-predicted^25^ aldehyde intermediate cinchonaminal (Figs. 1 and 2f and Supplementary Fig. 6). As with **12**, an isotope-labelled derivative of this target (putative cinchonaminal) was partially purified from fed plant tissues, tested as an intermediate by re-feeding to *C. pubescens* tissues and confirmed by LC–MS analysis to be incorporated into **1** and **2** (Fig. 2g). Isolation, semi-synthesis and NMR and MS/MS analyses confirmed that the target compound is the predicted cinchonaminal, but predominantly existing in the hemiaminal cyclized form **13** (herein referred to as cyclocinchonaminal; Fig. 2f). To our knowledge, this result marks the first time that this intermediate has been isolated and structurally characterized (Supplementary Fig. 7).

In summary, the identification and incorporation of these three intermediates—**11**, **12** and (cyclo)cinchonaminal (**13**)—led us to propose a revised biosynthetic hypothesis. Corynantheal is reduced to **11**, which is then cyclized to form the newly identified quaternary amine **12**. Cinchonium is converted into **7** (or the cyclized form **13**), the first biosynthetic intermediate with the distinctive indole–quinuclidine moiety of cinchona alkaloids. (Cyclo)cinchonaminal then undergoes oxidative rearrangement^25^ to form the quinoline scaffold found in all cinchona alkaloids, followed by reduction of the ketone function to produce the end products **1** and **2**.

## Conversion of corynantheal to malonyl-corynantheol

**Extended Data Fig. 1:**
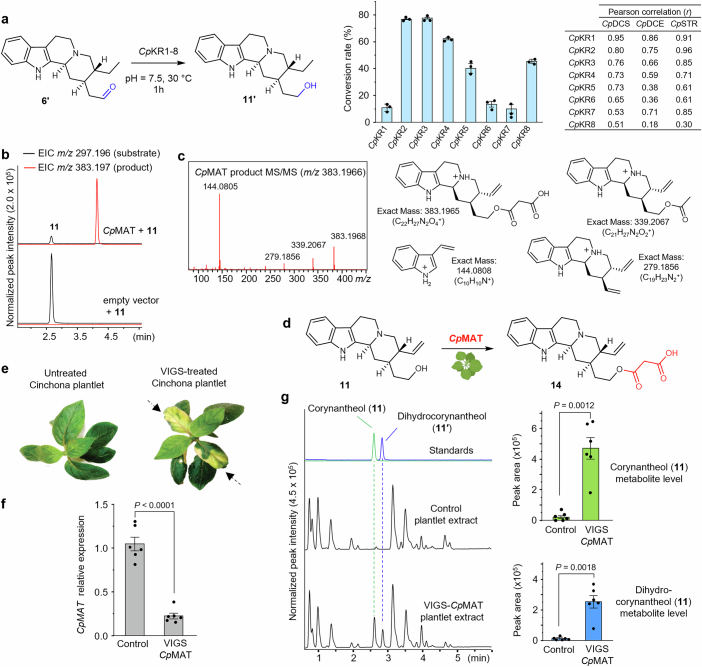
Functional characterization of corynantheal reductases (*Cp*KR1-8) and corynantheol malonyltransferase *Cp*MAT. **a**. Reaction catalyzed by *Cp*KR1-8 with varied level of catalytic efficiency and co-expression with other pathway upstream genes *CpDCS, CpDE* and *CpSTR*. Data on *Cp*KR1-8 activity are mean ± s.e.m. (*n* = 3). This experiment was repeated two times with similar results. **b**. Transient expression of *Cp*MAT in *N. benthamiana* with co-infiltration of corynantheol (**11**). Extracted ion chromatograms for corynantheol (**11**) (*m/z* 297.196 ± 0.05 Da [M + H]^+^, green-highlighted) and malonyl-corynantheol (**14**) (*m/z* 383.197 ± 0.05 Da [M + H]^+^, red-highlighted). This experiment was repeated three times. Similar results were obtained in vitro using purified recombinant enzyme (see Supplementary Fig. 8). **c**. MS/MS (20 to 50 eV) spectrum of the *Cp*MAT product and putative ion fragments of generated *m/z* 383.1966 [M + H]^+^. **d**. Reaction catalyzed by *Cp*MAT. **e**. VIGS-treated *C. pubescens* plantlets (transformed with a vector designed to silence magnesium chelatase subunit H (*Cp*MgChl) encoding gene) and untreated plantlets. Leaf bleaching (the yellow areas indicated by arrows) is the phenotypic characteristic of effective *CpMgChl* silencing. **f**. Relative expression of the gene encoding *Cp*MAT in *C. pubescens* plantlets transformed with a vector designed to silence both *Cp*MAT encoding gene and the marker gene *CpMgChl* (VIGS-*Cp*MAT) and in control plantlets (transformed with a vector designed to silence only the marker gene *CpMgChl*) indicate the knockdown of *CpMAT*. **g**. Representative total ion chromatogram (TIC) of leaf tissue extracts from VIGS-*Cp*MAT-transformed *C. pubescens* plantlets and control plantlets. Chromatograms of the standards (dihydro)corynantheol (**11** and **11′**) and bar graphs for metabolites levels of the *Cp*MAT knockdown tissue in comparison to controls are shown, corroborating the effective silencing of *Cp*MAT encoding gene. Data in **f** and **g** are mean ± s.e.m. (*n* = 6 biological replicates) and statistical analyses were performed using a two-sided, unpaired Welch’s t-test. VIGS assays were repeated two times and one additional time with *C. pubescens* plantlets grown under various conditions with similar results. The icon in **d** was created in BioRender; Lombe, B. https://biorender.com/8hz4n59 (2025). Source data

With this revised biosynthetic hypothesis in hand, we set out to identify the reductase responsible for converting **6** to **11**. We mined a RNA sequencing (RNA-seq) dataset from *C. pubescens* and selected eight putative reductases that were co-expressed with DCS (Pearson’s coefficient (*r*) > 0.5; Extended Data Fig. 1a). During reconstruction of the early-stage pathway in *N. benthamiana*, the last known intermediate, **6′**, was observed to undergo reduction to its corresponding alcohol, dihydrocorynantheol (**11′**)^26^, probably through the action of native reductases in *N. benthamiana*. Therefore, the selected candidates were expressed in *Escherichia coli*, purified and tested in vitro using the enzymatically accessible dihydro analogue of **6′** as the substrate. All tested enzymes (named KR1–KR8) displayed various levels of reductive activity. This result suggests that **6′** can be readily reduced in *C. pubescens* (Extended Data Fig. 1a).

We next investigated how the scaffold **11** is converted to **12**. Model chemistry has shown that **11′** can be converted to dihydrocinchonium (**12′**) via tosylation of the alcohol followed by refluxing^28,29^. We proposed that biosynthesis in *Cinchona* could proceed in a similar manner if a suitable leaving group is enzymatically installed on the corynantheol scaffold. This leaving group could then be eliminated through intramolecular nucleophilic substitution to generate the quinuclidine ring of **12**. We mined transcriptomic data obtained from *C. pubescens* bulk tissue for genes encoding putative transferases (sulfotransferases, acyltransferases, kinases and *O*-methyltransferases). A total of 30 candidates that were co-expressed with genes encoding the upstream DCE, DCS and STR enzymes were selected and transiently expressed in *N. benthamiana* leaves, followed by infiltration of **11**. LC–MS analyses of crude extracts of the transformed leaves indicated that only one enzyme, encoded by a putative BAHD-acyltransferase gene^30,31^, could metabolize **11** to produce a new compound with a mass that corresponded to a malonylated derivative of corynantheol (*m/z* of 383.197; Extended Data Fig. 1b,c). The same product was observed when the enzyme was assayed in vitro with corynantheol in the presence of malonyl-CoA (Supplementary Fig. 8a,b). MS/MS analysis (Extended Data Fig. 1c) and NMR data (Supplementary Fig. 8c) of the partially purified product established that the produced compound is the *O*-malonylated derivative of corynantheol (**14**, herein referred to as malonyl-corynantheol), thereby confirming that this enzyme is an *O*-malonyltransferase (MAT). Although MAT showed acetyltransferase activity in vitro, this substrate specificity was not observed in *N. benthamiana*, even when acetyl-CoA was exogenously infiltrated along with **11**. This result suggests that this observed in vitro acetylation is probably due to promiscuous enzyme activity (Supplementary Fig. 8d).

Notably, **14** could not be detected in any *Cinchona* tissues. To validate that **14** is a true pathway intermediate, we attempted to feed enzymatically synthesized isotopically labelled *d*_5_-malonyl-corynantheol (**14b**) to *C. pubescens* root tissue. However, NMR analysis in protic solvent (methanol-*d*_3_) indicated that **14b** hydrolyses to corynantheol over time (Supplementary Fig. 9). Moreover, LC–MS analysis of *C. pubescens* tissues fed with *d*_5_-malonyl-corynantheol revealed the presence of the demalonylated compound (**11b**). Therefore, although we observed isotopically labelled late-stage alkaloids (for example, **2**) in these feeding experiments (Supplementary Fig. 10), we could not unequivocally establish whether it was **11b** or **14b** that was being incorporated. We therefore developed a virus-induced gene silencing (VIGS) assay using in vitro germinated *C. pubescens* plantlets to assess the function of MAT in vivo. After silencing *MAT*, both congeners **11** and **11′**, and methoxylated analogues, accumulated in high levels in the VIGS-treated *Cinchona* leaves (Extended Data Fig. 1e,f and Supplementary Fig. 11a). Although levels of the downstream alkaloids were not affected after silencing (Supplementary Fig. 11b), presumably because leaves have high levels of the downstream **12** intermediate that would compensate for low levels of the upstream intermediate **11**, the substantial accumulation of corynantheol congeners strongly suggests that MAT acts on these substrates in vivo.

## Conversion of malonyl-corynantheol to cinchonium

**Fig. 3:**
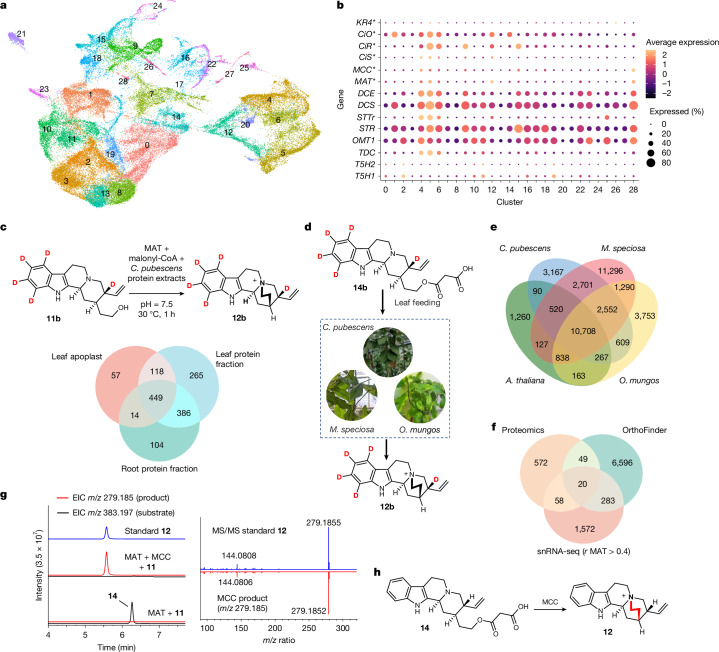
Discovery and functional characterization of MCC. **a**, Uniform manifold approximation and projection (UMAP) plot of *C. pubescens* leaf nuclei with high-quality snRNA-seq data, coloured by cell clusters. **b**, Gene expression heatmap of *C. pubescens* known upstream alkaloid biosynthetic genes across cell clusters shown in **a**. Genes marked with an asterisk are reported herein. **c**, Top, schematic showing cyclization activity of protein extracts from *C. pubescens*. Bottom, Venn diagram showing shared peptides among the proteomic fractions from *C. pubescens* that displayed catalytic activity. Assay results with protein extracts are shown in Supplementary Fig. 13. **d**, Detection of cyclization activity of *C. pubescens*,* O. mungos*and* M. speciosa* by feeding isotopically labelled **14b** to leaves. For LC–MS data showing results of incorporation, see Supplementary Fig. 14. **e**, Comparison of cross-species transcriptomes using the OrthoFinder algorithm (with *A. thaliana* included in the analysis to exclude broadly conserved plant genes). The numbers shown in the diagram are numbers of orthologue clusters. **f**, Integration of proteomics, cross-species transcriptomic comparison and leaf snRNA-seq data to refine candidate gene selection. The numbers shown in the diagram are numbers of genes. **g**, Transient expression of *MAT* alone and with *MCC* in *N. benthamiana* along with co-infiltration of **11**. Left, EICs for **14** (*m/z* 383.197 ± 0.05 Da [M+H]^+^, black) and for **12** (*m/z*  279.185 ± 0.05 Da, red). Chromatogram of the standard **12** is also shown (blue). This experiment was repeated three times with similar results. Right, MS/MS (20–50 eV) spectra of the standard **12** and the MCC product (*m/z* 279.1852 Da). **h**, Schematic of the reaction catalysed by MCC. Source data

With the biosynthetic function of MAT established in planta, we set out to identify the enzyme responsible for the proposed unusual cyclization-mediated displacement of malonate that would generate **12** from **14**. A wide range of candidate enzymes, including decarboxylases, esterases, hydrolases and oxidases, were tested in *N. benthamiana*. However, none exhibited detectable catalytic activity on **14**. At this point, annotation-based candidate selection became uninformative. Because a large number of putative biosynthetic genes (>5,000) were co-expressed with *MAT* in the RNA-seq dataset, we used a combination of approaches to narrow down the number of candidates to be screened. First, we generated a cell-type-resolved transcriptome through single-nucleus RNA sequencing (snRNA-seq) of *C. pubescens* young leaves (Fig. 3a and Supplementary Fig. 12). This dataset revealed a high enrichment of previously identified pathway genes that encode TDC, STR, STTr, DCS and DCE, as well as the newly identified MAT, in three specific clusters (clusters 4, 5 and 6; Fig. 3b). These clusters were putatively assigned as epidermal cells based on their homology to cell markers from the phylogenetically related plant *Catharanthus roseus*^32^. Second, crude soluble protein extracts obtained from *C. pubescens* root, leaf and leaf apoplastic fluid could cyclize **14** to form **12** (Fig. 3c and Supplementary Fig. 13). These protein extracts were then partially purified using ion exchange and size-exclusion chromatography, and catalytically active fractions were subjected to proteomic analyses. The proteomes of these three samples were then compared with each other and with the available transcriptomic data (Fig. 3c). Third, we noted a previously published report describing the presence of a cinchonium analogue (hydroxylated dihydrocinchonium), ophiorrhizine^33^, in *Ophiorrhiza major*, which, like *Cinchona*, is a member of the Rubiaceae family. Feeding the leaves of an available species from the *Ophiorrhiza* genus (*Ophiorrhiza mungos*) with **14b** led to high incorporation into *d*_*5*_-cinchonium (**12b**). This result suggests that the cyclase is conserved in this phylogenetically related plant (Fig. 3d and Supplementary Fig. 14). Notably, feeding *d*_*5*_-malonyl-corynantheol to another Rubiaceae species, *Mitragyna speciosa*, also led to the formation of **12b**, although cinchonium is not normally present in the metabolome of this plant. On the basis of these findings, we generated an *O. mungos* leaf transcriptomic dataset and compared it to available *C. pubescens* and *M. speciosa* transcriptome datasets to identify conserved biosynthetic genes^34,35^. *Arabidopsis thaliana*, which did not show cyclization activity and is phylogenetically distant to the three other plant species (Supplementary Figs. 14 and 15), was included in this analysis to exclude broadly conserved plant genes (Fig. 3e).

By integrating data from snRNA-seq (genes coexpressed with MAT, *r*  > 0.4), proteomics (shared peptides among all three *C. pubescens* protein fractions) and transcriptomic cross-species comparison (orthologous genes present in *C. pubescens*, *O. mungos* and *M. speciosa* but absent in *A. thaliana*), we compiled a list of 20 candidate genes (Fig. 3f). We expressed *MAT* and all these candidates together in *N. benthamiana*. Infiltration of **11** and subsequent LC–MS analyses revealed the formation of **12**. Unexpectedly, deconvolution of the enzyme candidates traced the cyclization activity to a gene annotated as a BAHD malonyltransferase, which we thereafter named malonyl-corynantheol cyclase (MCC) (Fig. 3g,h). Transiently knocking down the MCC-encoding gene in *C. pubescens* plantlets using VIGS led to an accumulation of **14** and related dihydro and methoxylated congeners. This result confirmed the function of this cyclase in vivo (Supplementary Fig. 16a). In vitro assays showed that MCC did not require cofactors for catalysis and exhibited strict substrate specificity, accepting only malonylated, but not acetylated, corynantheol (Supplementary Fig. 16b). Notably, although both MCC and MAT belong to the BAHD acyltransferase family, these two enzymes do not share high sequence similarity (19.5%) and are phylogenetically distant. MAT clusters with other known BAHD acyltransferases involved in monoterpene indole alkaloid biosynthesis, whereas MCC appears in an evolutionary distant clade (Supplementary Fig. 17). Moreover, MCC did not show malonylation activity and could not install the malonyl group on **11** required for subsequent cyclization to **12** (Supplementary Fig. 18). A potential explanation for the loss of this loss of acyl transferase activity is that its binding pocket for the malonyl-CoA substrate has been extensively mutagenized (Supplementary Fig. 19). A homology model indicates the presence of a binding pocket deeper in the active site. This could conformationally orient **14** such that the nucleophilic amine is proximal to the electrophilic carbon, and the alkyl-chain-bearing malonyl group is in the axial conformation, which would lead to the observed product stereochemistry (Supplementary Fig. 20). The discovery of MAT and MCC as key enzymes that mediate malonylation and subsequent demalonylative cyclization reveals a previously unknown biosynthetic strategy to form this quaternary amine and broadens the functional range of BAHD acyltransferases beyond canonical acyl transfer reactions^36^.

## Conversion of cinchonium to quinoline alkaloids

**Extended Data Fig. 2:**
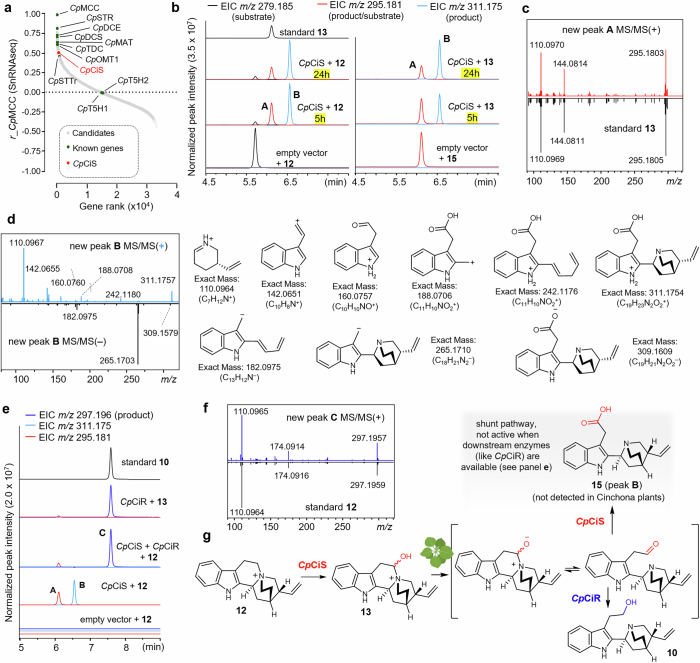
Functional characterization of cinchonaminal synthase (*Cp*CiS) and cinchonaminal reductase *Cp*CiR. **a**. Ranking of hydroxylase (OGD and P450) gene candidates based on coexpression with *Cp*MCC. **b**. Extracted ion chromatograms of the extract from the transient expression of *Cp*CiS in *N. benthamiana* with infiltration of cinchonium (**12**, left) and cyclocinchonaminal (**13**, right), which show that *Cp*CiS converts **12** into **13** (peak A) and then transforms **13** into an overoxidized compound (peak B). This experiment was repeated five times with similar results. **c**. MS/MS (20 to 50 eV) spectra of the *Cp*CiS product peak A and a standard cinchonaminal (**13**). **d**. MS/MS (20 to 50 eV) spectra in positive (top) and negative (bottom) mode of the *Cp*CiS product peak B and putative fragments revealing the compound to be the carboxylic acid analog of cinchonaminal, herein named cinchonaminic acid (**15**). **e**. Extracted ion chromatograms of the extract from the transient expression of *Cp*CiS and/or *Cp*CiR in *N. benthamiana* with infiltration of cinchonium (**12**) or cyclocinchonaminal (**13**), which show that *Cp*CiR reduces **13** into cinchonamine **10** (peak C) and prevents an over oxidation of **13** by *Cp*CiS. This experiment was repeated five times with similar results. **f**. MS/MS (20 to 50 eV) spectra of the *Cp*CiR product peak C and a standard cinchonamine (**10**). **g**. Reaction catalyzed by *Cp*CiS and *Cp*CiR. The icon in **g** was created in BioRender; Lombe, B. https://biorender.com/8hz4n59 (2025).

We then set out to find the gene responsible for the conversion of **12** to the next known biosynthetic intermediate cinchonamimal **7**. Hydroxylation of cinchonium would directly produce **13**, the cyclic form of **7**. Therefore, we searched for biosynthetic genes predicted to encode oxidases. All annotated cytochrome P450 enzymes and oxoglutarate-dependent dioxygenases were ranked on the basis of Pearson’s correlation coefficients with MCC using the snRNA-seq data (Extended Data Fig. 2a). A candidate annotated as a 2-oxoglutarate-dependent dioxygenase was confirmed to catalyse this transformation in both *N. benthamiana* and in vitro enzymatic assays, and was subsequently designated as cinchonaminal synthase (CiS) (peak A, Extended Data Fig. 2b,c and Supplementary Fig. 21a). Notably, CiS further oxidized cinchonaminal to form a compound with spectral data consistent with the carboxylic acid (herein named as cinchonaminic acid (**15**); peak B, Extended Data Fig. 2b–d). This compound was not observed in *Cinchona*, which indicated that the next pathway intermediate rapidly intercepts the initial enzymatic product before overoxidation can occur. Conversely, however, *Cinchona* is postulated to reduce **7** to form cinchonamine **10** (Fig. 1). Screening of candidate reductases, obtained through coexpression correlation with CiS (Supplementary Fig. 21b), led to the identification of an alcohol dehydrogenase that converts **7** to **10**, which we named cinchonaminal reductase (CiR) (peak C, Extended Data Fig. 2e–g). *CiS* and *CiR* were also enriched in the same clusters (clusters 4–6; Fig. 3b) as the upstream biosynthetic genes (for example, *MAT* and *MCC*) according to the snRNA-seq data. This result suggests that cell-type localization is maintained in these downstream pathway steps.

**Extended Data Fig. 3:**
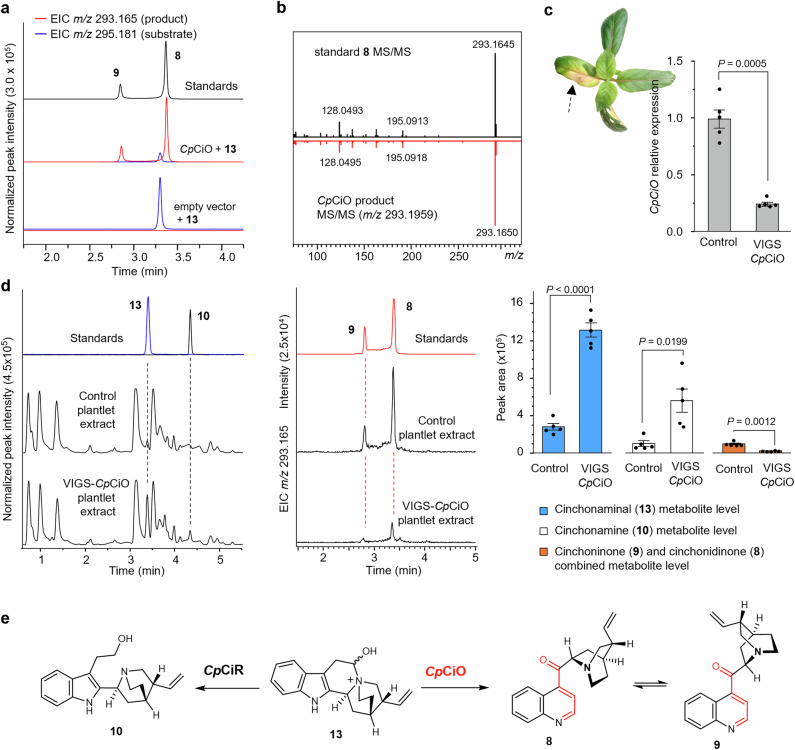
Functional characterization of cinchonamine oxidase (*Cp*CiO). **a**. Extracted ion chromatograms (EIC) of the extract from the transient expression of *Cp*CiS in *N. benthamiana* with infiltration of cinchonaminal (**13**), showing that *Cp*CiO converts **13** into quinoline compounds cinchonidinone (**8**) and cinchoninone (**9**), which exist in a tautomeric equilibrium. This experiment was repeated five times with similar results. **b**. MS/MS (20 to 50 eV) spectra of the *Cp*CiO product and standard **8**. **c**. VIGS-treated *C. pubescens* plantlets transformed with a vector designed to silence *CpCiO* and the marker gene *CpMgChl*. The phenotypic characteristic resulting from *CpMgChl* silencing (leaf bleaching) is indicated by an arrow and the relative expression of *CpCiO* is shown. **d**. Representative total ion chromatogram (TIC) of leaf tissue extracts from VIGS-*Cp*CiO-transformed *Cinchona* plantlets and control plantlets, along with standards cinchonamine and cyclocinchonaminal (**10** and **13**); EIC of cinchonidinone (**8**) and cinchoninone (**9**), and bar graphs for metabolite levels of the *Cp*CiO knockdown tissue in comparison to controls are shown, corroborating the effective silencing of *CpCiO*. In **c** and **d**, the values represent mean ± s.e.m. (*n* = 5 biological replicates) and statistical analyses were performed using a two-sided, unpaired Welch’s t-test. **e**. Reaction catalyzed by *Cp*CiO. Source data

Conversion of cinchonaminal **7** into a quinoline moiety probably involves oxidative opening of the indole, followed by cyclization and dehydration to form the quinoline scaffold. As the phylogenetically related plant *M. speciosa* uses a cytochrome P450 enzyme (family CYP71) to oxidize an indole to a spirooxindole^35,37^, we speculated that a *Cinchona* CYP71 P450 enzyme might also be involved in the oxidative transformation of this indole substrate. Because the feeding studies and metabolite accumulation levels indicated that these late-stage steps were more highly expressed in root and stem tissues (Supplementary Figs. 4 and 22–27), we performed a clustering analysis to prioritize gene candidates based on this expression pattern (Supplementary Fig. 28). Among the top CYP71 P450 candidates selected on the basis of the coexpression analysis with CiS, a functional enzyme was identified, which we named cinchonaminal oxidase (CiO) (Extended Data Fig. 3a,b). CiO catalysed the oxidation of **7**, which produced the two ketone quinoline isomers cinchonidinone (**8**) and cinchoninone (**9**). These stereoisomers, which are known to exist in equilibrium^38,39^, were observed in a 1:0.23 ratio, which is approximately the same ratio observed in *C. pubescens*. The enzymatic activity of CiO was further confirmed through in vitro assays using yeast microsomes (Supplementary Fig. 29), which demonstrated that this single P450 enzyme can catalyse the indole-to-quinoline ring expansion. VIGS of CiO was also conducted in *C. pubescens*. Leaves in which *CiO* was silenced showed a significant accumulation of **13** and **10** along with decreased biosynthesis of both ketone quinoline alkaloid stereoisomers (**8** and **9**). This result provides evidence for the physiological function of CiO in planta (Extended Data Fig. 3c–e).

**Extended Data Fig. 4:**
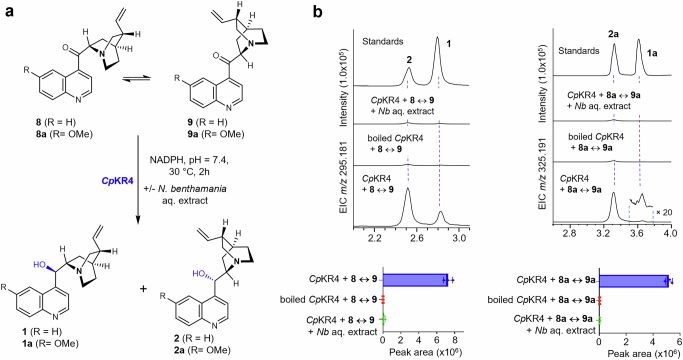
Functional characterization of the keto-reductase *Cp*KR4. **a**. in vitro reaction of *Cp*KR4 in the presence or absence of *N. benthamania* aqueous extract. **b**. EIC along with the LC-MS peak areas of reactions products, showing that *Cp*KR4 reduces the ketone-quinolines to the Cinchona alkaloids, but this activity is inhibited by *N. benthamania* metabolites present in the aqueous extract. Another stereoisomer is also produced (Supplementary Fig. 31). Data are mean ± s.d. (*n* = 3). This experiment was repeated three times with similar results. Source data

The final step in cinchona alkaloid biosynthesis involves the reduction of the ketone moiety that is generated after the formation of the quinoline scaffold. Previous studies have suggested that this transformation is probably catalysed by a NADPH-dependent oxidoreductase^40^. A total of 60 NADPH-dependent reductase genes that were coexpressed with CiO in the bulk transcriptomic dataset and/or snRNA-seq were cloned and tested (Supplementary Dataset 1). Screening in *N. benthamiana* did not lead to an active candidate. Therefore, we prioritized the selected candidate genes on the basis of the analysis of expression trends (Supplementary Fig. 28a–c) and re-expressed 15 putative aldo-keto reductases and alcohol dehydrogenases (including the above mentioned KR1–KR8) in *E. coli* for assays in vitro. Only one enzyme, KR4, displayed clear reductase activity, converting both non-methoxylated and methoxylated quinoline ketone stereoisomers into the corresponding alcohols (Extended Data Fig. 4a,b). This functional enzyme also reduced the aldehyde function of corynantheal scaffolds (Extended Data Fig. 1a), a result that further supported its involvement in the biosynthesis of cinchona alkaloids. Notably, despite robust activity in vitro, KR4 did not show detectable activity when assayed in *N. benthamiana* leaves after transient expression and infiltration of quinoline ketones. We designed a fusion protein comprising CiO and KR4 for transient expression in *N. benthamiana*. Although oxidation activity of CiO was observed, no reduced product was obtained. To test whether *N. benthamiana* contained endogenous components that inhibit this aldo-keto reductase, diluted crude extracts from *N. benthamiana* leaves were added to in vitro reactions, substituting 10% of the reaction volume. The addition of *N. benthamiana* extract abolished KR4 activity in vitro, a result that implicates the presence of an unknown inhibitory factor that suppresses KR4 function (Extended Data Fig. 4a,b and Supplementary Fig. 30a,b). These results provide a reasonable explanation for the lack of detectable activity in *N. benthamiana* and highlights a potential limitation in using this heterologous system for functional validation of reductases.

Although the two late-stage genes *CiO* and *KR4* were selectively and highly expressed in stem and root tissues, these genes also showed clear enrichment in clusters 4–6 of the leaf snRNA-seq dataset, clusters previously associated with nearly all upstream alkaloid biosynthetic genes (Fig. 3b). Notably, the expression of monoterpene indole alkaloid pathway genes in the Apocynaceae species *C*. *roseus* seems to switch from epidermal cells to specialized idioblast cells in the late stages of alkaloid biosynthesis^32^. By contrast, in *Cinchona*, a member of the Rubiaceae family, all alkaloid biosynthetic genes identified in this study were selectively enriched in epidermal cells (clusters 4–6). This consistent cell-type-specific expression pattern of the biosynthetic genes in *Cinchona*, along with high coexpression in bulk tissue (Supplementary Fig. 31), provides further support that these newly identified genes function in the same metabolic pathway and contribute to the biosynthesis of cinchona alkaloids. However, we note that KR4 did not exhibit absolute stereoselectivity with the ketone quinoline alkaloids (**8**, **9**, **8a** and **9a**). In particular, with the methoxylated analogues **8a**–**9a**, a stereoisomer that is not observed in the plant was formed in higher levels than the naturally observed stereoisomers (Supplementary Fig. 30c,d). Although this reductase showed clear activity with the quinolines, it is possible that a reductase with higher stereoselectivity remains to be identified.

## Reconstitution of natural and unnatural alkaloids

**Fig. 4:**
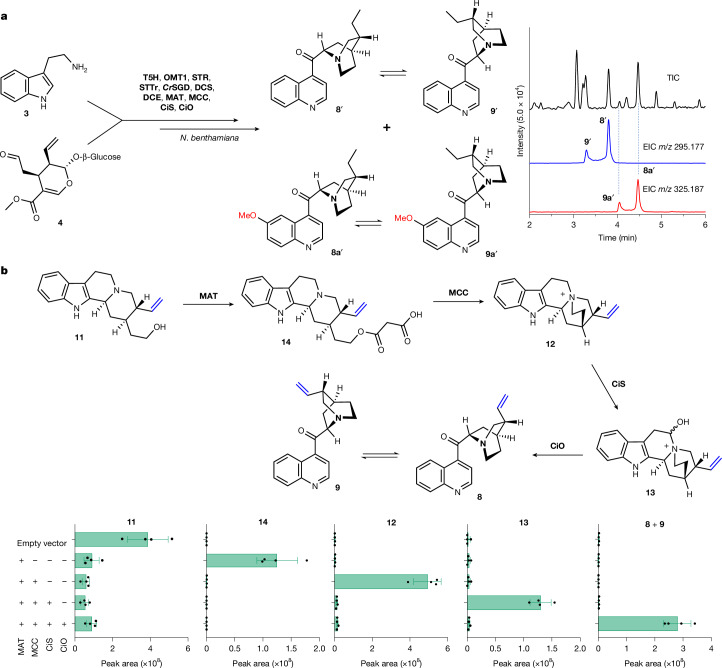
Biosynthesis of quinoline cinchona alkaloids. **a**, Production of both methoxylated and non-methoxylated cinchona alkaloids in *N. benthamiana* following transient expression of the indicated biosynthetic enzymes and co-infiltration of **3** and **4**. TIC, total ion chromatogram. **b**, LC–MS peak areas of products detected in *N. benthamiana* following transient expression (+) or not (–) of the indicated biosynthetic enzymes from *C. pubescens* and incubation with **11** using a leaf-disc assay. Data are the mean ± s.d. (*n* = 4 biological replicates). Source data

**Extended Data Fig. 5:**
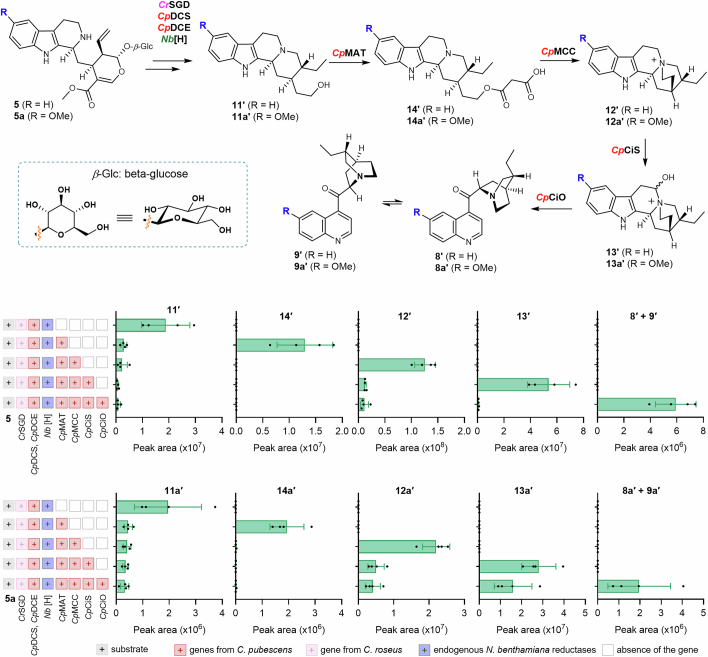
Step-by-step biosynthesis of Cinchona alkaloids in *N. benthamiana.* LC-MS peak areas of products detected in *N. benthamiana* following transient expression of the indicated biosynthetic enzymes and incubation with either strictosidine (**5**) or 10-methoxystrictosidine (**5a**). Data are presented as mean ± s.d. (*n* = 4 biological replicates). Source data

With these enzymes identified and functionally characterized, the cinchona alkaloid biosynthetic pathway could be reconstituted in the heterologous host *N. benthamiana*. The well-known central monoterpene indole alkaloid precursor **5** was externally supplied as a substrate to discs taken from *N. benthamiana* leaves that had been transformed with DCS, DCE, MAT, MCC, CiS and CiO, along with a construct encoding strictosidine β-glucosidase (SGD) from the phylogenetically related plant *C. roseus* (*Cr*SGD), an orthologue of SGD with high catalytic activity (Supplementary Fig. 3). No corynantheal reductase gene was included because the endogenous *N. benthamiana* enzymes efficiently reduced (dihydro)corynantheal. As anticipated, strictosidine was efficiently converted to **8′** and **9′** (Extended Data Fig. 5 and Supplementary Figs. 32 and 33). Reconstruction with the methoxylated analogue 10-OMe strictosidine (**5a**) instead led to the formation of **8a′** and **9a′**. This result confirms the substrate flexibility of the downstream enzymes and provides clear experimental support for the parallel biosynthetic route previously proposed for methoxylated alkaloids^26^ (Extended Data Fig. 5). Notably, the addition of STR, T5H, OMT1 and the vacuolar strictosidine transporter^26^ STTr to the gene stack, along with replacement of **5** with **3** and **4**, led to the formation of a mixture of methoxylated and non-methoxylated keto quinolines (**8′**, **9′**, **8a′** and **9a′**), which mimicked the occurrence of these dihydro alkaloids in *Cinchona* (Fig. 4a and Supplementary Fig. 34). Because under these assay conditions, DCS leads to **6′**, we tested synthetic **11** as an exogenous substrate for *N. benthamiana* leaf discs (transformed with MAT, MCC, CiS and CiO) to observe the formation of **8** and **9** (Fig. 4b). Moreover, intermediates that we had identified at the outset of this study—**11**, **12** and **13**—were detected when these transformed *N. benthamiana* leaves were subjected to analysis. By contrast, **14** was undetectable when enzymes downstream of MAT (MCC, CiS and CiO) were present, a result consistent with the absence of this transient intermediate in native *C. pubescens* metabolite profiles (Fig. 4b). Collectively, these findings highlight the potential for the biosynthetic production of medicinally relevant quinoline alkaloids using these biosynthetic genes.

**Fig. 5:**
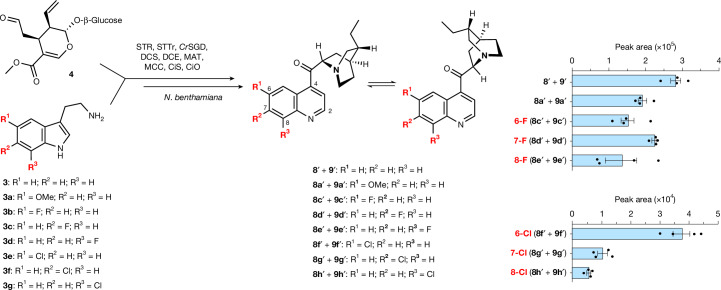
Biosynthesis of halogenated analogues of quinoline cinchona alkaloids. Directed biosynthesis of fluorinated and chlorinated analogues of cinchona alkaloids in *N. benthamiana* following transient expression of the indicated biosynthetic enzymes and co-infiltration of **4** and halogenated tryptamines (**3b**–**3g**). LC–MS peak areas are shown and data are the mean ± s.e.m. (*n* = 4 biological replicates). Data from assays using the native substrates, **3** and **3a**, are included for relative comparison of the efficiency of the enzymes. For EICs and MS/MS data, see Supplementary Figs. 35 and 36. Source data

Directed biosynthesis, in which the host generating the product is supplied with an exogenous unnatural starting substrate, is a method that has been used for decades to produce industrially important natural product analogues^34,41–45^. However, for successful production of end-product analogues, this approach requires that all of the downstream enzymes catalyse conversion of all corresponding unnatural biosynthetic intermediates. As *Cinchona* biosynthetic enzymes naturally act on both methoxy and dihydro derivatives, we rationalized that these biosynthetic enzymes would be well suited for the production of unnatural alkaloid analogues. Transformed *N. benthamiana* leaves (STR, *Cr*SGD, DCS, DCE, MAT, MCC, CiS and CiO) were infiltrated with a series of analogues of **3** (5-fluorotryptamine, 5-chlorotryptamine, 6-fluorotryptamine, 6-chlorotryptamine, 7-fluorotryptamine and 7-chlorotryptamine), along with the natural co-substrate **4**. In all cases, we saw consumption of the halogenated tryptamine substrate, along with the presence of compounds with a mass and MS/MS pattern consistent with the formation of the corresponding dihydrocinchoninone and dihydrocinchonidinone analogues (Fig. 5 and Supplementary Figs. 35 and 36). These results suggest that the identified biosynthetic genes can be used to produce halogenated cinchona alkaloid analogues, which will be potentially useful for medicinal chemistry applications given the clinical use and attractiveness of halogenated quinolines^46^.

## Conclusion

Here we described the genetic basis that underlies the biosynthesis of the quinoline–quinuclidine scaffold that characterizes cinchona alkaloids. Although aspects of the biosynthesis, such as the mechanism underlying the formation of the C-18–C-19 double bond, remain to be resolved, the discovery of these genes enables biosynthetic conversion from known starting materials to (dihydro)cinchoni(di)ne and (dihydro)quini(di)ne. These discoveries were achieved by integrating modern omics approaches—namely single nucleus sequencing and comparative transcriptomics—along with classical methods of isotopic feeding and enzyme activity fractionation from plant tissues, and the development of a functional in planta assay using VIGS. We demonstrated how simultaneous application of these approaches can substantially streamline the list of gene candidates to be screened. We further demonstrated the application of these biosynthetic genes for the production of natural cinchona alkaloids and non-natural halogenated alkaloid analogues in a *N. benthamiana* host. These discoveries reveal how the distinctive cinchona alkaloid scaffold is biosynthesized and highlights prospects for the metabolic engineering of these complex bioactive alkaloids.

### Reporting summary

Further information on research design is available in the Nature Portfolio Reporting Summary linked to this article.

## Online content

Any methods, additional references, Nature Portfolio reporting summaries, source data, extended data, supplementary information, acknowledgements, peer review information; details of author contributions and competing interests; and statements of data and code availability are available at 10.1038/s41586-026-10227-x.

## Supplementary information

Supplementary Information This file contains Supplementary Materials and Methods, supplementary list of compound names, structures and numbers mentioned in this work, Supplementary Figs. 1–70, Supplementary Tables 1–11 and Supplementary References.

Reporting Summary

Supplementary Data List of 20 genes screened for cyclase activity.

Supplementary Data FASTA file of all genes discovered in this study.

Supplementary Data Source data for Supplementary Figs. 4, 11, 16, 18, 22 and 31.

Peer Review File

## Source data

Source Data Fig. 3

Source Data Fig. 4

Source Data Fig. 5

Source Data Extended Data Fig. 1

Source Data Extended Data Fig. 3

Source Data Extended Data Fig. 4

Source Data Extended Data Fig. 5

## Data Availability

Data supporting the findings of this work are available within this paper and its Supplementary Information. Sequences of reported genes are included in the Supplementary Information and have been deposited into the National Center for Biotechnology (NCBI) GenBank under the following accession numbers: *MAT* (PX842829), *MCC* (PX842830), *CiS* (PX842831), *CiR* (PX842832), *CiO* (PX842833), *SGD1* (PX842834), *SGD2* (PX842835), *SGD3* (PX842836), *KR1* (PX842837), *KR2* (PX842838), *KR3* (PX842839), *KR4* (PX842840), *KR5* (PX842841), *KR6* (PX842842), *KR7* (PX842843) and *KR8* (PX842844). *C. pubescens* bulk RNA-seq and snRNA-seq, ONT full-length cDNA and ONT gDNA reads have been deposited into the NCBI under BioProject PRJNA1347772. Raw reads from RNA-seq of *O. mungos* leaves have been deposited into the NCBI (accession PRJNA1413194). Bulk RNA-seq of *M. speciosa* from a previous study was downloaded from the NCBI (accession PRJNA1244102) and the transcriptome of *A. thaliana* used is implemented and available in OrthoVenn3 (https://orthovenn3.bioinfotoolkits.net). Proteomic data have been deposited into the ProteomeXchange Consortium (dataset identifier PXD068683). Source data are provided with this paper.
