# Supplementary material for: Biosynthesis of cinchona alkaloids
Source: Nature. 2026 Mar 18;653(8113):306–14. doi: 10.1038/s41586-026-10227-x (PMC13149305; doi:10.1038/s41586-026-10227-x)
Supplement: Supplementary file 1 — This file contains Supplementary Materials and Methods, supplementary list of compound names, structures and numbers mentioned in this work, Supplementary Figs. 1–70, Supplementary Tables 1–11 and Supplementary References. [file 41586_2026_10227_MOESM1_ESM.pdf]

nature portfolio

https://doi.org/10.1038/s41586-026-10227-x

Supplementary information

Biosynthesis of cinchona alkaloids

In the format provided by the authors and unedited

Supplementary information

Biosynthesis of Cinchona alkaloids

Blaise Kimbadi Lombe[1,#], Tingan Zhou (周庭安)[1,#], Gyumin Kang[1], Joshua C. Wood[2], John P. Hamilton[2,3], Klaus Gase[1], Yoko Nakamura[1], Ryan M. Alam[1], Ron P. Dirks[4], Lorenzo Caputi[1], C. Robin Buell[2,5,6]*, Sarah E. O'Connor[1]*

1. Department of Natural Product Biosynthesis, Max Planck Institute for Chemical Ecology, Jena, Germany
2. Center for Applied Genetic Technologies, University of Georgia, Athens, GA, USA
3. Department of Crop and Soil Sciences, University of Georgia, Athens, GA, USA
4. Future Genomics Technologies, Leiden, The Netherlands
5. Institute of Plant Breeding, Genetics and Genomics, University of Georgia, Athens, GA, USA
6. The Plant Center, University of Georgia, Athens, GA, USA

* Corresponding author; # equal contribution

Table of contents

Materials and Methods……………………………………………………………………………1
Supplementary list of compound names, structures, and numbers mentioned in this work…………..21
Supplementary Fig. 1. Biosynthetic pathways to Cinchona alkaloids………………………………...23
Supplementary Fig. 2. Summary of feeding experiments with labeled substrates……………………25
Supplementary Fig. 3. Identification of *C. pubescens* strictosidine *β*-glucosidases ..……..………..26
Supplementary Fig. 4. Untargeted metabolomics analysis of samples from feeding assays with d_5-corynantheol……………………………………………………..27
Supplementary Fig. 5. Incorporation of d_5-cinchonium into later-stage Cinchona alkaloids………...28
Supplementary Fig. 6. MS/MS spectra of cinchonamine and cinchonaminal………………………...29
Supplementary Fig. 7. Structural characterization of cinchonaminal………………………………....31
Supplementary Fig. 8. Additional data on *Cp*MAT activity and structural characterization of *Cp*MAT product………………………………………………………32
Supplementary Fig. 9. NMR spectra showing degradation over time of malonyl-corynantheol……..34
Supplementary Fig. 10. *C. pubescens* feeding with d_5-malonyl-corynantheol………………………..35
Supplementary Fig. 11. Additional results from *Cp*MAT silencing…………………………………...36
Supplementary Fig. 12. *C. pubescens* leaf single nuclei RNA-seq libraries…………………………37
Supplementary Fig. 13. Malonyl-corynantheol cyclization activity of *Cinchona* protein extracts…...38
Supplementary Fig. 14. Cyclization of d_5-malonyl-corynantheol by different plant species ………39
Supplementary Fig. 15. Phylogenetic tree of the four plants used in the cross-species comparison…39
Supplementary Fig. 16. Additional results on *Cp*MCC functional characterization …………………40
Supplementary Fig. 17. Phylogenetic tree of *Cp*MAT and *Cp*MCC with BAHD acyltransferases ……………..…………………………………………....42
Supplementary Fig. 18. Enzymatic activity comparison between *Cp*MAT and *Cp*MCC……………43
Supplementary Fig. 19. Comparison of *Cp*MAT and *Cp*MCC with a previously characterized BAHD malonyltransferase ………………………………………………45
Supplementary Fig. 20. Docking model of *Cp*MCC with malonyl-corynantheol……………………46
Supplementary Fig. 21. a. *In vitro* enzymatic assay of *Cp*CiS and candidate reductases ranking …...47
Supplementary Fig. 22-27. Metabolic profiling of *C. pubescens* tissues……………………………..48
Supplementary Fig. 28. Bulk-tissue transcriptomic analysis for prioritizing gene candidates……….54
Supplementary Fig. 29. *In vitro* enzymatic assay of *Cp*CiO……………………………………….55
Supplementary Fig. 30. Additional data on the characterization of *Cp*KR4…………………………57
Supplementary Fig. 31. Expression profiles of Cinchona alkaloid pathway genes …………………59
Supplementary Fig. 32-33. LC-MS data evidencing formation of dihydrocorynantheol and malonyl-dihydrocorynantheol in reconstruction assays …………………60
Supplementary Fig. 34. Biosynthesis of *Cinchona* quinoline alkaloid scaffolds from secologanin and tryptamine……………………………………………………..........62
Supplementary Fig. 35-36. LC-MS data evidencing directed biosynthesis of fluorinated and chlorinated quinoline compounds…………………...…………………………..63
Supplementary Fig. 37-38. NMR spectra of d_5-corynantheol ………………………..……………65
Supplementary Fig. 39-45. NMR spectra of corynantheol ……………….…………….……………67
Supplementary Fig. 46-51. NMR spectra of cinchonium……………………...………………………..74
Supplementary Fig. 52-59. NMR spectra of cyclocinchonaminal………………………… ….…...80
Supplementary Fig. 60-70. NMR spectrum of malonyl-corynantheol………………………………..88

Table S1: General assembly statistics for the *Cinchona pubescens* genome……………………….99
Table S2: General statistics for *C. pubescens* leaf snRNA-seq……………………………………..99
Table S3. Nucleotide sequences for genes described and used in this study ……………………….100
Table S4. Primers used for genes cloning in this study…………………………………………….113
Table S5-S7. Primers for VIGS gene fragments amplification and qPCR,
and VIGS gene fragments……………………………………………………………114
Table S8-S11. Tabulated NMR data of corynantheol, cinchonium, cyclocinchonaminal
and malonyl-corynantheol…………………………………………………………...116
References……………………………………………………………………………………..121

Materials and Methods

Plant materials

Cinchona pubescens seeds were obtained from the Edinburgh Botanical Garden under agreement 20061109820061110 and germinated in-house, as previously reported[1,2]. *Mitragyna speciosa* (Thailand variety) were kindly provided with permission by the University of Florida, USA in 2014 and *Ophiorrhiza mungos* plants from the Palmengarten Frankfurt Main botanical garden (IPEN-number: XX-0-FRP-16457), respectively. All three species were cultivated in a standard soil mix under greenhouse conditions at the Max Planck Institute for Chemical Ecology (MPI-CE, Jena, Germany), with temperatures maintained between 24-28 °C, a 12 h light/12 h dark photoperiod, and relative humidity ranging from 60% to 80%.

Nicotiana benthamiana plants were grown in a standard soil mix in the MPI-CE greenhouse at 22 °C under a 16 h light/8 h dark cycle and 60% relative humidity. *N. benthamiana* plants were typically cultivated for 3 to 4 weeks prior to infiltration with *Agrobacterium tumefaciens* strain GV3101.

Chemicals

All solvents used for extraction, chemical synthesis, and semi-preparative HPLC were of HPLC grade, while those used for LC-MS analyses were of MS grade; all solvents were purchased from Fisher Scientific. Cinchonidine (**1**), cinchonine (**2**), quinine (**1a**), quinidine (**2a**), dihydroquinine (**1a′**), and dihydroquinidine (**2a′**) standards were obtained from Sigma-Aldrich. Dihydrocinchonidine (**1′**) and dihydrocinchonine (**2′**) were purchased from TCI, and cinchonamine (**10**) was sourced from ChemSpace.

Cinchona intermediates including dihydrocorynantheal (**6′**), cinchonidinone (**8**), cinchoninone (**9**), quininone (**8a**), and quinidinone (**9a**) were obtained through enzymatic or semi-synthesis as previously described[1]. Strictosidine (**5**) and its methoxylated analog (**5a**) were enzymatically synthesized by coupling secologanin (**4**) with tryptamine (**3**) or 5-methoxytryptamine (**3a**), followed by purification via semi-preparative HPLC[3]. Malonyl-corynantheol (**14**) was produced enzymatically, as detailed in the ***Synthesis of compounds*** section. Cinchonium (**12**) and cyclized cinchonaminal (cyclocinchonaminal, **13**) standards were either isolated from *C. pubescens* leaf extracts via semi-preparative HPLC or semi-synthesized as described in the same section.

RNA extraction, purification, and sequencing

Bulk-tissue RNA-seq dataset from five *C. pubescens* organs (1st leaf, 2nd leaf, 3rd leaf, stem, and root)

was generated as previously reported[2]. For generating the *O. mungos* RNA-seq dataset used for cross-species transcriptomics analysis, total RNA was extracted from *O. mungos* young and mature leaves using the RNeasy Plant Mini Kit (Qiagen), followed by further purification and concentration with the RNA Clean & Concentrator-5 Kit (Zymo Research). All of these procedures were performed according to manufacturer's guidelines. RNA quality was assessed using an Implen NanoPhotometer N60. Two RNA samples, one from young leaves and another from mature leaves meeting quality thresholds required for sequencing (≥ 400 ng total RNA; A260/280 = 1.8-2.2; A260/230 ≥ 1.8), were submitted to Novogene, where sequencing, transcript assembly and annotation were performed following standard protocols. Geneious Prime 2024 was used for primers design, sanger sequencing analysis, and translation of transcriptomes to proteomes.

Genome assembly and annotation

To enable the omics driven pathway discovery, we generated a *de novo* genome assembly using Oxford Nanopore Technologies (ONT) long-reads. DNA was extracted from young leaves of *C. pubensces* using the Qiagen DNeasy Plant Mini Kit. The quality of the DNA was analyzed using Genomic DNA ScreenTape on an Agilent 4200 TapeStation System (Agilent Technologies Netherlands BV, Amstelveen, The Netherlands) and the quantity was measured using a Qubit 3.0 Fluorometer (Life Technologies Europe BV, Bleiswijk, The Netherlands). Nanopore sequencing libraries were prepared using the Ligation Sequencing Kit (PromethION SQK-LSK109) according to the manufacturer's instructions (Oxford Nanopore Technologies, Oxford, UK). Each library was run on an R9.4.1 PromethION flowcell (FLO-MIN106; Oxford Nanopore Technologies (ONT), Oxford, UK) and reloaded on a daily basis after a nuclease flush with Flow Cell wash kit (EXP-WSH004). Illumina DNAseq libraries were prepared using the Nextera Flex kit according to the manufacturer's instructions (Illumina, San Diego, CA, USA) and were sequenced in paired-end mode (2 × 150 bp) using Illumina's NovaSeq 6000 technology. Reads were basecalled using Guppy (v6.3.2)[4] in super high accuracy (SUP) mode. Reads were filtered to remove reads less than 10kb with SeqKit (v0.16.1)[5] and then input into the Flye (v2.9.1)[6] assembler with the following parameters: --iterations 0 --scaffold. The assembly was then polished using in two rounds using the raw nanopore data with Medaka (v1.7.1)[7] with the option -m r941_min_sup_g507 set. The assembly was then polished using Illumina short reads (NCBI: SRR20784021)[8] with two rounds of Hapo-G (v1.0)[9] with the -u option set. After polishing the assembly was filtered for contigs less than 10kb using SeqKit and then the contigs were named and ordered based on size. The final genome assembly metrics are listed in Table S1. Benchmarking Universal Single Copy Orthologs[10] revealed 99% complete orthologs, of which 91.1 are single copy and 7.9% are duplicated.

To provide transcript evidence for gene annotation, RNA from leaves, stems and roots was used as input

into the Oxford Nanopore SQK-PCB109 kit to create full-length cDNA libraries, which were subsequently sequenced on a MinION using a FLO-MIN106 RevD flowcell. The *C. pubescens* genome assembly was repeat masked with RepeatMasker (v4.1.2-p1)[11] using a custom repeat library constructed with RepeatModeler (v2.0.3)[12] and ProtExcluder (v1.2)[13], then merged with Viridiplantae repeats from RepBase (v20150807)[14]. Illumina RNA-seq reads were trimmed with Cutadapt (v2.10)[15] with a minimum length of 100 nt and quality cutoff of 10 and aligned with HISAT2 (v2.1.0)[16]. The ONT cDNA reads were processed with Pychopper (v2.5.0)[17] and reads > 500 nt were aligned with minimap2 (v2.17-r941)[18]. The alignments were assembled with StringTie (v2.2.1)[19] and transcripts <500 nt were removed. Gene models were predicted using BRAKER2 (v2.1.6)[20] on the soft-masked genome, then refined with two rounds of PASA2 (v2.5.2)[21] yielding the final working set of gene models. Functional annotations were assigned from the top significant hit of BLASTP (v2.12.0)[22] searches against the TAIR (v10)[23] and Swiss-Prot Plant (2015_08)[24] databases or PfamScan (v1.6)[25] matches against PFAM (v35.0)[26].

Nuclei isolation, single-nucleus library preparation, and sequencing

Nuclei were isolated from young leaf tissue following a previously described protocol with 0.025% triton[27]. RNase inhibitor (Sigma Protector RNase Inhibitor, Cat. No. 3335402001) was added to the nuclei isolation buffer for a final concentration of 0.2U/μL. Nuclei were concentrated by spinning at 300G for 5 minutes. A total of 30,400 and 32,400 nuclei (replicate 1 and 2 respectively) were input into the PIPseq T20 v4.0Plus protocol along with 2 μL of RNase Inhibitor. Subsequent libraries were sequenced on an Element Biosciences Aviti Instrument in paired end 75nt mode at the UC Davis DNA Technologies & Expression Analysis Core.

Reads were processed using the "barcode" command from pipseeker-v3.1.3 from Fluent Biosciences. The pipseeker processed fastq files and the generated barcode whitelist were used as input into the STARsolo (v2.7.10b) alignment program. The following parameters were employed; --alignIntronMax 5000, --soloUMIlen 12, --soloCellFilter EmptyyDrops_CR, --soloFeatures GeneFull, --soloMultiMappers EM, --soloType CB_UMI_Simple. Alignments were done against the v1 genome assembly. Seurat (v4.3.0.1)[28] was used for downstream analysis. Samples were filtered using the criteria in Table S2. Samples were also run through DoubletFinder[29] to remove suspected doublets. Reciprocal PCA (RPCA) was used to integrate the two replicates together using the top 3,000 variable genes. The top 60 principal components were used with a resolution parameter of 0.6 to calculate the Uniform manifold approximation and projection (UMAP).

Gene candidate selection based on single-nuclei transcriptome

Co-expression analysis at the single-nuclei level was performed using normalized expression values

from each cluster, obtained from the single-nuclei RNA-seq dataset. Pearson correlation coefficients (*r*) were calculated in Microsoft Excel using the expression profiles of the pathway immediate upstream gene (*Cp*MAT, *Cp*MCC, *Cp*CiO, or *Cp*CiS) as bait genes. Candidate genes from the *C. pubescens* single-nuclei transcriptome showing moderate to strong co-expression with the bait ($r > 0.40$, as performed in Microsoft Excel 2014) were selected for functional screening via transient expression in *N. benthamiana* (see section ***Transient gene expression in N. benthamiana***). In parallel, bulk-tissue RNA-seq data from *C. pubescens* were used to support candidate selection based on functional annotation. Putative gene functions were inferred via BLAST against multiple databases, including InterPro, UniProt, NCBI, KEGG, and KOG.

Tissue-specific expression analysis and gene candidate selection

The bulk-tissue RNA-seq dataset from *C. pubescens* was used to assess tissue-specific gene expression. Transcript abundance was quantified as FPKM (Fragments Per Kilobase of exon model per Million mapped fragments) and provided by the sequencing company (BGI). FPKM values were $\log_2$-transformed and z-score normalized before visualization and clustering using ClusterGVis (v0.1)[30]. Hierarchical clustering and heatmap analysis were employed to explore expression patterns across tissues. For late-stage *CpCiO* gene discovery, Cluster 4, characterized by consistently low expression in leaf samples and high expression in both stem and root, was selected for candidate prioritization. To further narrow down the candidate set, differential expression analysis was performed between the 1st leaf and root samples using DESeq2 (v1.51.6)[31]. Genes were considered differentially expressed if they met the thresholds of adjusted p-value < 0.05 and $|\log_2(\text{fold change})| \geq 1$. A volcano plot was used to visualize differentially expressed gene (DEG) distributions. Genes overlapping between Cluster 4 and root-upregulated DEGs were selected as candidate *CpCiO* genes. Final candidates were further filtered based on the presence of "P450" in their putative functional annotation. The volcano plot and Venn map were generated using SRplot v2025[32].

Cross-species comparison and orthologue analysis

Cross-species orthologous gene cluster analysis was conducted using OrthoVenn3, an online platform for comparative analysis of orthologous gene families across multiple species[33]. Protein sequences (peptide FASTA files) of *C. pubescens*, *M. speciosa*, and *O. mungos* were derived from transcriptome assemblies using Geneious and uploaded to the platform. The *A. thaliana* proteome, used as a reference dataset within OrthoVenn3, was included as a negative control. Orthologous clustering was performed using the OrthoFinder algorithm, with default parameters (E-value cutoff: 1e-2; inflation value: 1.50). Clusters containing orthologs shared among *C. pubescens*, *M. speciosa*, and *O. mungos*, but absent in

A. thaliana, were considered as candidate clusters. Notably, *CpSTR*, *CpDCS*, *CpDCE,* and *CpMAT* were confirmed in the above target cluster; thus, the *C. pubescens* genes in that cluster were selected as candidates for the discovery of *CpMCC*.

Cloning and heterologous expression of candidate genes

DNAse I-treated RNA from root, stem, and youngest leaves of *C. pubescens* was reverse-transcribed using the Superscript IV VILO Master Mix (Thermo Fisher) to synthesize cDNA, which was stored at –20 °C and used for amplification of all biosynthetic genes (Table S3). Genes of interest were amplified using Invitrogen Platinum SuperFi II PCR Master Mix (Thermo Fisher), with gene-specific primers synthesized by Sigma-Aldrich. All primers sequences can be found in Table S4. PCR products were separated on 1% agarose gels after mixing with TriTrack DNA loading dye (Thermo Fisher). DNA fragments of the expected size were excised and purified using the Zymoclean Gel DNA Recovery Kit (Zymo Research). All amplicons contained appropriate 5′ and 3′ overhangs for cloning into plant or microbial expression vectors. Restriction enzymes were purchased from New England Biolabs. All molecular biology kits and enzymatic reactions were performed according to the manufacturers' instructions.

For transient gene expression in *N. benthamiana*, the purified PCR products were ligated into the modified 3Ω1 vector[34] (pre-linearized with BsaI) through In-Fusion (Takara) cloning. In-Fusion reactions were transferred into *E. coli* Top 10 competent cells. Recombinant colonies were selected on LB agar containing spectinomycin (200 µg/mL), and confirmed colonies were cultured in LB liquid medium with the same antibiotic for plasmid extraction using the Wizard Plus SV Minipreps DNA Purification Kit (Promega). Insert sequences were validated by Sanger sequencing (Azenta Life Sciences). Verified constructs were electroporated into *A. tumefaciens* GV3101 using a MicroPulser (BioRad). Transformed colonies were selected on LB agar supplemented with rifampicin (50 µg/mL), gentamicin (50 µg/mL), and spectinomycin (200 µg/mL), and incubated at 28 °C for 2 days. Single colonies were inoculated in 7-10 mL LB containing the same antibiotics and cultured at 28 °C, 250 rpm for 24 h. Glycerol stocks (25%) were prepared and stored at –80 °C for future use.

For soluble protein expression in *E. coli*, purified PCR products of genes of interest were inserted into HindIII and KpnI-linearized pOPINF vector[35] using In-Fusion cloning. Assemblies were transformed into *E. coli* Top10 cells, and transformants were selected on LB agar containing carbenicillin (100 µg/mL). Plasmids were extracted and sequence-verified as described above. Verified constructs were introduced into *E. coli* BL21 (DE3) for protein expression. Colonies were selected on carbenicillin plates, confirmed by colony PCR, and used to inoculate 2-3 mL LB medium with carbenicillin

(100 µg/mL). Overnight cultures were grown at 37 °C, 200 rpm, and stored as 25% glycerol stocks at –80 °C.

For yeast expression, purified PCR products of genes of interest were inserted into the MCS2 sites of pESC-His vectors (pre-linearized by SalI and BamHI) by In-Fusion assembly as previously described[36]. Assemblies were transformed into *E. coli* Top10 cells, and transformants were selected on LB agar containing carbenicillin (100 µg/mL). Plasmids were extracted and sequence-verified following the procedures described above. Verified constructs were transformed into yeast strain *S. cerevisiae* WAT11 (ade2; contains the *Arabidopsis thaliana* cytochrome P450 reductase I gene, ATR1)[37] using the Frozen-EZ Yeast Transformation II Kit (Zymo Research). Transformants were selected on SD-His medium (6.7 g/L yeast nitrogen base without amino acids, 2 g/L drop-out mix lacking histidine) supplemented with 2% glucose (w/v), and incubated at 30 °C for 48 h. Positive colonies were identified by colony PCR and inoculated into 2 mL SD-His medium containing 2% glucose, followed by incubation at 30 °C and 220 rpm for 48 h. Cultures were stored as 25% glycerol stocks at –80 °C for future use.

Transient gene expression *in N. benthamiana*

Transient gene expression of candidate genes in *N. benthamiana* was performed following the protocol of Hawes *et al.*[38] with minor modifications. Briefly, *A. tumefaciens* strains harboring the respective gene constructs were grown overnight at 28 °C, 250 rpm in 10 mL LB medium containing rifampicin (20 µg/mL), gentamicin (30 µg/mL), and spectinomycin (200 µg/mL). Cells were harvested by centrifugation at 4,000 × *g* for 10 min, and the supernatant was discarded. Pellets were gently resuspended in infiltration buffer (50 mM MES, 2 mM Na_3PO_4, 27.8 mM glucose, 10 mM $MgCl_2$, 100 µM acetosyringone) and washed once to remove residual medium and antibiotics. After a second centrifugation (4,000 × *g*, 10 min), cells were resuspended in fresh infiltration buffer.

For single-gene assays, bacterial suspensions were adjusted to an OD_{600} of approximately 0.4. For multiple-gene assays, strains were pooled at equal cell densities, ensuring a minimum final OD_{600} of 0.1 per strain. Suspensions were incubated at room temperature in the dark with gentle rocking for 1 h before infiltration. Leaves were infiltrated from the abaxial side using a 1 mL needleless syringe, covering the entire leaf surface. Only the second pair of fully expanded leaves (counting from the apical meristem) was used for infiltration on each plant. At 3 days post-infiltration, substrates dissolved in water with 1% DMSO (50-250 µM) were infiltrated into the same leaf areas. Treated regions were marked for sampling. After 24 h, four 10 mm-diameter leaf disks were excised from each marked area, flash-frozen in liquid nitrogen, and stored at –80 °C. A positive infiltration experiment was repeated at least twice, using biological replicates consisting of at least four leaves from two independent *N.*

benthamiana plants.

Metabolite extraction from the harvested *N. benthamiana* leaves

Harvested and flash-frozen *N. benthamiana* leaf disks were ground to a fine powder in 2 mL microcentrifuge tubes using two 2 mm tungsten carbide beads on a TissueLyser II (Qiagen) at 22 Hz for 1.5 min. Each sample was extracted with 300 µL of methanol, followed by vigorous vortexing for 30 s. Samples were sonicated at room temperature for 15 min to facilitate extraction. The extracts were then centrifuged at >13,000 × *g* for 5 min, and the supernatants were filtered through 0.22 µm PTFE syringe filters before analysis.

Expression and purification of soluble proteins from *E. coli*

Single colonies of *E. coli* BL21 (DE3) harboring the expression plasmids were grown overnight in 10 mL LB medium (containing 100 µg/mL carbenicillin) at 37 °C. The next day, 1 mL of the overnight culture was used to inoculate 100 mL of 2×YT medium. Cultures were grown at 37 °C until an OD_{600} of 0.6-0.8 was reached, followed by induction with 100 µM IPTG and incubation at 18 °C for at least 16 h. Cells were harvested by centrifugation at 4,000 × *g* for 10 min and resuspended in 10 mL of A1 buffer (50 mM Tris-HCl pH 8.0, 50 mM glycine, 500 mM NaCl, 20 mM imidazole, 5% [v/v] glycerol), supplemented with 0.2 mg/mL lysozyme and an EDTA-free protease inhibitor cocktail (Roche cOmplete). The suspension was incubated on ice, and cells were lysed by sonication using a Vibra-Cell sonicator (Sonics) at 40% amplitude with a cycle of 3 s on / 2 s off for 5 min total. Crude lysates were cleared by centrifugation at 4,000 × *g* for 15 min at 4°C. The supernatant was filtered with hydrophilic glass filters and loaded onto a pre-equilibrated HisTrap HP column (Cytiva) connected to an ÄKTA pure system (Cytiva). The column was pre-washed with A1 buffer until baseline stabilization before loading the protein samples. After sample loading, the B1 buffer (50 mM Tris-HCl pH 8.0, 50 mM glycine, 500 mM NaCl, 500 mM imidazole, 5% [v/v] glycerol) was applied to elute the binding proteins from the column. Fractions containing the target protein were pooled and subjected to buffer exchange into A4 buffer (20 mM HEPES pH 7.5, 150 mM NaCl) using centrifugal concentrators with molecular weight cutoffs of 10 or 30 kDa, depending on the protein. Purified proteins were aliquoted and stored at –80 °C.

Expression and isolation of microsomes from yeast

S. cerevisiae WAT11 harboring expression constructs or empty vector controls was streaked onto SD-His agar plates (6.7 g/L yeast nitrogen base without amino acids, 2 g/L drop-out mix lacking histidine, 2% glucose, and 20 g/L agar) from glycerol stocks and incubated at 30 °C for 48 h for strain recovery.

Single colonies were inoculated into 2 mL SD-His liquid medium with 2% glucose and cultured overnight at 30 °C, 200 rpm. The overnight culture was then transferred into 100 mL fresh YPD medium (10 g/L yeast extract, 20 g/L peptone, 2% glucose), followed by incubation at 30 °C, 200 rpm for another 24 h. Cells were harvested by centrifugation at 4,000 × *g* for 5 min at room temperature and resuspended in 100 mL YPG medium (10 g/L yeast extract, 20 g/L peptone, 2% galactose) to induce protein expression. Cultures were incubated at 30 °C, 200 rpm for 18-24 h.

Following induction, cells were harvested by centrifugation (7,500 × *g*, 4 °C, 10 min) for microsome isolation, following the method of Pompon *et al.*[39] with modifications. Cell pellets were resuspended in 20 mL TEK buffer (50 mM Tris-HCl pH 7.4, 1 mM EDTA, 100 mM KCl) and centrifuged again under the same conditions. Pellets were then resuspended in 2 mL ice-cold TES buffer (50 mM Tris-HCl pH 7.4, 1 mM EDTA, 600 mM sorbitol, 10 g/L BSA). Cells were disrupted by bead beating using 0.5 mm glass beads (equal volume) in a cold room (4 °C) for six cycles of 1 min beating at 5,000 rpm, with 1 min cooling on ice between cycles. After lysis, 5 mL ice-cold TES buffer was added, and lysates were collected by decanting into pre-chilled tubes. Glass beads were washed three times with TES buffer, and combined lysates were centrifuged at 7,500 × *g* for 10 min at 4 °C to remove debris. The supernatant was ultra-centrifuged at 100,000 × g for 90 min at 4 °C. The resulting microsomal pellet was washed sequentially with 1 mL ice-cold TES buffer and 1 mL TEG buffer (50 mM Tris-HCl pH 7.4, 1 mM EDTA, 20% glycerol), then resuspended in 1 mL TEG buffer using a Dounce homogenizer. Microsomes were aliquoted and stored at –80 °C for subsequent *in vitro* assays.

LC-MS analysis

Method 1

LC-MS analysis was routinely performed on an UltiMate 3000 Ultra-High Performance Liquid Chromatography system (UHPLC; Thermo Fisher) connected to an Impact II UHR-Q-ToF (Ultra-High Resolution Quadrupole Time-of-Flight) mass spectrometer (Bruker). Separations were performed on a Phenomenex Kinetex XB-C18 column (100 × 2.1 mm, 2.6 µm, 100 Å) maintained at 40 °C. The mobile phase consisted of solvent A (Milli-Q water with 0.1% formic acid) and solvent B (acetonitrile), with the following gradient: 10% B for 1 min; linear increase to 30% B over 6 min; 90% B for 1.5 min; and re-equilibration at 10% B for 2.5 min. The flow rate was set to 0.6 mL/min, and the injection volume was 2 µL. All mass spectrometry data were acquired under the following conditions. Ionization was performed in positive mode using pneumatically assisted electrospray ionization (ESI+) with a capillary voltage of 3500 V and an endplate offset of 500 V. The nebulizer gas pressure was set to 2.5 bar, and nitrogen was used as the drying gas at 250 °C with a flow rate of 11 L/min. Data were acquired at 12 Hz over a mass range of *m/z* 80-1000, employing data-dependent MS/MS with active exclusion set to

0.2 min and a reconsideration threshold of 1.8-fold. Fragmentation was triggered above an absolute intensity threshold of 400, with a total cycle time of 0.5 s. The collision energy was stepped from 20 to 50 eV. Mass calibration was performed at the beginning and end of each run using sodium formate solution in isopropanol, automatically infused by an external syringe pump at 0.18 mL/min (5 mL syringe, 10.3 mm inner diameter). To avoid salt contamination and minimize injection peaks, the first 0.5 minute of each chromatographic run was diverted to waste before directing the flow into the mass spectrometer. Chromatographic separation conditions were optimized according to the physicochemical properties of the target analytes. Data were acquired using Bruker Compass qtofControl 5.2.109 / Hystar 5.1.5.1 and their processing and analysis were performed using Bruker Compass DataAnalysis 6.1 and MetaboScape 2024b.

Method 2

For high-resolution metabolite analysis, UHPLC-HRMS was performed using a Vanquish UHPLC system coupled to a Q-Exactive Plus Orbitrap mass spectrometer (Thermo Fisher). Metabolite separation was carried out on a Waters Acquity UPLC BEH C18 column (2.1 × 50 mm, 1.7 µm, 130 Å) maintained at 40 °C. The mobile phase consisted of solvent A (Milli-Q water with 0.1% formic acid) and solvent B (acetonitrile), with the following gradient: 5% B for 0 min, linear increase to 30% B over 10 min, rapid increase to 100% B within 1 min, hold at 100% B for 1 min, and re-equilibration at 5% B for 2.5 min. The flow rate was set to 0.6 mL/min with an injection volume of 4 µL. The Q-Exactive Plus Orbitrap mass spectrometer was operated with a heated electrospray ionization (HESI) source. Source parameters were optimized for the UHPLC flow rate of 0.6 mL/min as follows: sheath gas flow rate, 55; auxiliary gas flow rate, 15; sweep gas flow rate, 3; spray voltage, 3.50 kV; capillary temperature, 275 °C; auxiliary gas heater temperature, 450 °C; and S-lens RF level, 50. Data acquisition was performed in positive ion mode using full-scan MS at a resolution of 70,000 over the *m/z* range 100-1000. Full-scan MS combined with data-dependent MS/MS (full MS/dd-MS2 Top 5) was used to simultaneously acquire precursor and fragmentation spectra. The dd-MS2 acquisition parameters were set as follows: resolution, 17,500; mass isolation window, 4.0 *m/z*; and collision energy (NCE), 35-40%. All spectral data were recorded in centroid mode. The mass spectrometer was calibrated using Pierce™ positive and negative ion calibration solutions (Thermo Fisher). Samples for LC-MS-Orbitrap analysis were further diluted 10-fold compared with those prepared for LC-MS-QTOF analysis. This method was used as the standard protocol for all biochemical assay samples unless stated otherwise. Data were acquired using Thermo Scientific Xcalibur 4.7.69.37. LC-MS related graphs were prepared using OriginPro 9.6.0, GraphPad Prism 10.4.1, or SRplot v2025 (https://www.bioinformatics.com.cn), and were assembled in Microsoft PowerPoint 2024.4b.

Extraction and isolation of compounds from *C. pubescens* leaves

Immature leaves (600 g) from *C. pubescens* plants were snap frozen in liquid nitrogen and ground using a mortar and pestle. The resulting powder was extracted with 500 mL of methanol by sonication for 1h (x 2). After filtration, the solvent was evaporated under reduced pressure, and the resulting crude extract (12 g) was resuspended in 50 mL water containing 0.1% formic acid and exhaustively washed with n-hexane. The washed aqueous phase was dried and re-extracted with 20 mL of methanol (x 5). After evaporation of solvent, the obtained alkaloid-enriched concentrate (about 1 g) was redissolved in 3 mL of methanol, filtrated over a 0.2 µm PTFE membrane and resolved on a Phenomenex Luna C18 column (250 × 30 mm, 5 µm, 100 Å). The separation was achieved using water containing 0.1% formic acid (solvent A) and acetonitrile (solvent B) with a linear gradient from 10% to 41% solvent B over 25 min and a flow rate of 30 mL/min. In the course of the separation, 16 fractions were collected and analyzed by LC-MS (Method 1). Fraction 9 (17.1-18.1 min) was further fractionated on a Phenomenex Kinetex XB-C18 column (250 × 10 mm, 5 µm, 100 Å) using a linear gradient from 10% to 30% acetonitrile in water (supplemented with 0.1% FA) and a flow rate of 7 mL/min, to yield 9 subfractions. Subfractions 9-2 (13.8-14.8 min) and 9-3 (14.8-15.8 min) were combined and a portion was subjected to purification (see details below), affording 0.3 mg and 0.15 mg of compounds revealed as cinchonium (**12**) and cyclocinchonaminal (**13**) by LC-MS dereplication with standards prepared by semi-synthesis. These chromatographic separations were conducted on an Agilent 1260 Infinity II HPLC system (Agilent Technologies) with an Agilent 1290 Infinity II fraction collector. The monitoring of the elution of the compounds was done at four wavelengths: 210, 240, 290, and 360 nm. Data were acquired using Agilent OpenLAB CDS ChemStation 35900.

Purification of compounds by HPLC

Compound purification was performed using an Agilent 1260 Infinity HPLC system (Agilent Technologies) equipped with an autosampler, binary pump, column oven, diode array detector (DAD), and fraction collector. Separations were carried out on a Phenomenex Kinetex XB-C18 column (4.6 × 100 mm, 5 µm, 100 Å) maintained at 40 °C, with water containing 0.1% formic acid (solvent A) and acetonitrile (solvent B) as the mobile phases. A linear gradient from 10% to 20% solvent B over 17 min was applied, with a flow rate of 0.8 mL/min. Compounds were detected by UV absorption at 195, 214, 254, and 290 nm. Collected fractions were analyzed by LC-MS-QTOF using LC-MS Method 1 (described above), and fractions containing the target compound were pooled and concentrated using a Genevac EZ-2 Plus evaporator. The purified products were dissolved in 1.5 mL methanol, transferred to glass vials, and dried under nitrogen at room temperature.

Feeding experiments

For feeding experiments, d_5-corynantheol (**11b**) was prepared as a 100 µM solution in water with 1% (v/v) DMSO. Similarly, d_5-malonyl-corynantheol (**14b**), d_5-cinchonium (**12b**), and d_5-cyclocinchonaminal (**13b**) were prepared. Plant tissues for feeding assays were freshly harvested from *in vitro*-grown *C. pubescens* plantlets cultivated under previously reported conditions[2]. 1st leaf, 2nd leaf, 3rd leaf, along with young stem and young secondary root tissues, were collected and cut into small pieces suitable for incubation in 48-well plates. Each well contained one piece of tissue submerged in 200 µL of the labeled compound solution, followed by incubation for 24 h. Sterile Milli-Q water was used as a negative control, with other tissues incubated under identical conditions. When necessary, the incubation period was extended to up to 7 days. Plates were sealed with parafilm and incubated at 25 °C with gentle agitation using a FinePCR Confide-S202H thermo mixer (Rose Scientific) at the lowest shaking speed. Following incubation, tissues were extracted by adding 100 µL methanol per mg of fresh weight, followed by sonication for 15 min to facilitate extraction. Methanolic extracts were filtrated and subsequently analyzed by LC-MS as described above.

Untargeted metabolomics analysis of samples from feeding assays

Stem samples from feeding experiments were analyzed by LC-MS-QTOF analysis using LC-MS Method 1 (described above). Raw data were analyzed using Bruker Compass MetaboScape 2024b software. Peak detection and quantification were carried out using the T-Rex 3D algorithm within the untargeted metabolomics workflow, with an intensity threshold of 1,000. The resulting output included a list of mass features with retention times and peak intensities, based on automated integration of extracted ion chromatograms. To identify potentially newly formed metabolites, samples fed with d_5-corynantheol were compared to negative controls (water-fed samples). A minimum fold change of 1,000 was applied for candidate selection; peak areas below detection in controls were set to 1 for fold-change calculations. Volcano plots were generated using SRplot[32,40]. To further refine peak candidate selection, a targeted search for compounds containing the conserved labeled indole structure (*m/z* 148.10586) was performed in MetaboScape 2024b.

***C. pubescens* protein extraction, fractionation and activity assays**

Extraction and fractionation of leaf and root proteins. Young (immature) leaves were harvested from 1.5-year-old *C. pubescens* plants and were snap frozen in liquid nitrogen. Three of these plants were uprooted to collect young roots, which were thoroughly washed with water and then snap frozen in liquid nitrogen. The frozen tissues were separately ground using a mortar and pestle with a small scoop of polyvinylpolypyrrolidone (PVPP, 100-150 mg) and were stored at –80 ºC. To extract crude

proteins, a total of 15 g leaf powder and 4 g root powder were transferred to prechilled 15-mL tubes, mixed with ice-cold extraction buffer (50 mM Tris-HCl pH 7.4, 50 mM glycine, 500 mM NaCl, 20 mM imidazole, 10% [v/v] glycerol, 1 mM PMSF, EDTA-free protease inhibitor cocktail) in a 1:4 (w/v) ratio and then incubated with stirring at 20 rpm in a cold room. After 1h of incubation, samples were centrifuged at 4,000 rpm for 10 min at 4 °C to pellet plant debris. The supernatants were filtered through Miracloth (Merck-Millipore), yielding crude extracts. To precipitate proteins, solid $(NH_4)_2SO_4$ (up to 70%, w/v) was added to the crude extracts, the suspensions were then centrifuged at 10,000 rpm for 10 min at 4 ºC, and the pellets resuspended in 5-10 mL of the extraction buffer. The resulting leaf and root protein concentrates were dialyzed overnight in 5 L of Tris-HCl buffer (25 mM, pH 8.0) in a cold room and then applied to a HiScreen Capto Q ImpRes column. After removal of unbound compounds with the buffer (25 mM Tris–HCl pH 8.0) at 1.2 mL/min flow rate, proteins were eluted with increasing NaCl concentration and 2 mL fractions were collected and tested by adding d_5-malonyl-corynantheol and using LC-MS to detect the cyclized product. Catalytically active fractions were pooled, subjected to a HiLoad 16/60 Superdex 200 PG column and eluted with A4 buffer (20 mM HEPES pH 7.5, 150 mM NaCl) at a 1.0 mL/min flow rate. Fractions of 1.5 mL were collected and again tested for d_5-malonyl-corynantheol cyclization activity. Fractions with the highest enzymatic activity were combined and concentrated using Amicon Ultra Centrifugal Filters (30 kDa MWCO, Millipore UFC9030). The concentrated leaf and root protein fractions were ultimately verified for the cyclization activity, aliquots were prepared, snap frozen in liquid nitrogen and stored at −80 °C. Total protein concentrations were measured using the Pierce Detergent Compatible Bradford Assay Kit.

Extraction of leaf apoplast proteins. The leaf apoplast protein extract was obtained following the infiltration–centrifugation method[41]. Fifteen young leaves were excised from 6-month-old *C. pubescens* plants using a scalpel, rinsed in distilled water, and then dried by gently blotting on soft paper tissues. One to three leaves were placed in a 60 mL plastic syringe, which was then filled up to the 40 mL mark with ice-cold apoplast MES buffer (50 mM). Buffer infiltration into leaf apoplastic spaces was performed by pulling and gently releasing the plunger while covering the syringe tip to create negative pressure. Buffer-infiltrated leaves were blotted dry with paper tissues and two to four such leaves were stacked together in the same orientation and carefully rolled up within Parafilm, then pushed into a 5 ml plastic syringe without plunger with the petioles facing to the bottom of the syringe barrel. The syringe with buffer-infiltrated leaves was placed in 13 mL tube and centrifuged at 1,000 rpm and 4 °C for 10 min to collect apoplast extract. After a positive test of d_5-malonyl-corynantheol cyclization with an aliquot of apoplast extract was detected, the whole apoplastic extract was centrifuged at 10,000 rpm and 4 °C for 10 min and the supernatant was concentrated using centrifugal concentrators (Amicon Ultra Centrifugal Filters, 30 kDa MWCO, Millipore UFC5030). Aliquots of the verified active apoplastic

concentrate were snap frozen in liquid nitrogen and stored at −80 °C. The protein concentration was estimated using the Pierce Detergent Compatible Bradford Assay Kit.

Enzyme assays with *C. pubescens* protein extracts. The enzyme assays for cyclization activity that guided the fractionation of *C. pubscens* protein extracts were conducted in a volume of 100 µL containing HEPES (50 mM, pH 7.4), d_5-corynantheol (20 µM), malonyl-CoA (100 µM), *Cp*MAT (2.0 µM) and variable concentration of the test plant protein extracts, depending on the anticipated complexity of the extracts/fractions. Thus, for initial crude protein extracts 30 µg were used, for dialyzed extracts 25 µg, and for fractions after ion exchange and size exclusion chromatography 1-10 µg was used. When centrifugal concentrators were utilized, 20 µg of membrane-retained proteins and 25 µg for the flow through were used. The reactions were run at 30 ºC for 1 h, after which they were quenched by adding 200 µL of methanol. Samples were centrifuged at 15,000 rpm for 2-5 min, followed by filtration through 0.22 µm PTFE filters and LC-MS analysis using Method 1 described above.

Proteomics analysis

Sample preparation. Leaf, root, and apoplast protein fractions with verified cyclization activity obtained as described above were subjected to proteomics analysis, performed at the Proteomics core facility, EMBL (Heidelberg, Germany), following the previously published SP3 protocol conducted on the Thermo Fisher KingFisher Apex™ platform[42]. For digestion, trypsin was used in a 1:20 ratio (protease:protein) in 50 mM triethylammonium bicarbonate (TEAB) supplemented with 5 mM tris(2-carboxyethyl)phosphine hydrochloride (TCEP) and 20 mM 2-chloroacetamide (CAA). Digestion was carried out for 5 hours at 37 °C. The samples were dried down and reconstituted in 4% acetonitrile, 1% formic acid in LC-MS grade water for injection.

MS data acquisition. Samples were run on an UltiMate 3000 RSLCnano LC system (Thermo Fisher Scientific) equipped with a trapping µ-Precolumn C18 PepMap™ 100 cartridge (300 µm × 5 mm, 5 µm, 100 Å, Thermo Fisher Scientific) and an analytical nanoEase™ M/Z HSS T3 column (75 µm × 250 mm, 1.8 µm, 100 Å; Waters). Samples were trapped at a constant flow rate of 30 µL/min using 0.05% trifluoroacetic acid (TFA) in water for 6 minutes. After switching in-line with the analytical column, which was pre-equilibrated with solvent A (3% dimethyl sulfoxide [DMSO], 0.1% formic acid in water), the peptides were eluted at a constant flow rate of 0.3 µL/min using a gradient of increasing solvent B concentration (3% DMSO, 0.1% formic acid in acetonitrile). Peptides were introduced into an Orbitrap Fusion™ Lumos™ Tribrid™ mass spectrometer (Thermo Fisher Scientific) via a Pico-Tip emitter (360 µm OD × 20 µm ID; 10 µm tip, CoAnn Technologies) using an applied spray voltage of 2.2 kV. The capillary temperature was maintained at 275 °C. Full MS scans were acquired in profile mode over an *m/z* range of 300–1,500, with a resolution of 120,000 at *m/z* 200 in the Orbitrap. The

maximum injection time was set to 250 ms, and the AGC target limit was set to 50%. The instrument was operated in data-dependent acquisition (DDA) mode, with MS/MS scans acquired in the Iontrap in rapid scan mode. The maximum injection time was set to 35 ms, with an AGC set to 'standard'. Fragmentation was performed using higher-energy collisional dissociation (HCD) with a normalized collision energy of 30%, and MS2 spectra were acquired in centroid mode. The quadrupole isolation window was set to 1.6 *m/z*, and dynamic exclusion was enabled with a duration of 60 seconds. Only precursor ions with charge states 2–7 were selected for fragmentation.

MS data analysis. Raw files were then searched using MaxQuant (version 2.4.9.0)[43] against a FASTA database of *C. pubescens* proteome. The following modifications were included into the search parameters: Carbamidomethylation on C as fixed modification; Oxidation (M) and Acetylation (protein N-terminus) as variable modifications. For the full scan (MS1) a mass error tolerance of 20 ppm and for MS/MS (MS2) spectra of 0.5 Da was set. For protein digestion, 'trypsin' was used as protease with an allowance of maximum 2 missed cleavages requiring a minimum. The mass spectrometry proteomics data have been deposited to the ProteomeXchange Consortium via the PRIDE[44] partner repository with the dataset identifier PXD068683.

Virus induced gene silencing (VIGS) in *C. pubescens*

Susceptibility assays of *C. pubescens* to VIGS. The VIGS experiments in *C. pubescens* were adapted from previously described procedures[45-49]. VIGS susceptibility investigations were performed targeting magnesium-chelatase subunit H gene as a potential VIGS phenotype marker. Briefly, *C. pubescens* magnesium-chelatase subunit H (*Cp*MgChl) gene sequence was retrieved from *C. pubescens* transcriptome by BLAST-homology search using known sequences of magnesium-chelatase subunit H genes from *Catharanthus roseus* (transcript CRO_04G021190.1)[50] and *Papaver rhoeas*[51]. A 300 bp fragment of the coding region of *Cp*MgChl was amplified from *C. pubescens* leaf cDNA by PCR using Phusion™ High-Fidelity DNA Polymerase (Thermo Fischer scientific) according to the instructions of the manufacturer. Leaf cDNA was obtained as described above by converting isolated total RNA using SuperScript IV VILO reverse transcriptase (Thermo Fisher Scientific). Gene fragment and primers sequences are reported in Tables S5 and S6. The obtained gene fragment was gel-purified and ligated with the *Eco*RI and BamHI XhoI digested VIGS vector pTRV2[45] using the In-Fusion Snap Assembly Master Mix (Takara bioscience), yielding pTRV2-*Cp*MgChl which was sequence verified by Sanger sequencing. This construct was used to transform *A. tumefaciens* GV3101, which was then applied to sterile *Cinchona* plantlets, as described below. *A. tumefaciens* with a pTRV2 derivative containing no MgChl gene fragment (empty vector) was used to transform plantlets serving as the control group. Twelve days post infection, spots of bleaching (yellowing) could be observed on young leaves of pTRV2-*Cp*MgChl treated plants, which is a phenotype characteristic of MgChl silencing. No bleaching

was observed in plantlet leaves infected with empty pTRV2 plasmid, even after 1 month. Bleached areas of leaves were excised 3 weeks post infection and subsequent comparative qPCR analysis (details below) confirmed the MgChl silencing and comparative LC-MS analysis showed no change in alkaloid composition, thereby establishing VIGS as a useful tool for studying the *in vivo* function of alkaloid biosynthetic genes in *Cinchona* plantlets.

Construction of *Agrobacterium* strains for alkaloid biosynthetic gene silencing. A fragment of the coding region of the biosynthetic gene of interest (300 bp for *Cp*MAT, 298 bp for *Cp*MCC and 303 bp for *Cp*CiO, Table S6) were chosen based on lack of redundancy in the *Cinchona* genome. Fragments were amplified from *C. pubescens* leaf cDNA, by PCR with primers designed for In-Fusion HD cloning (Takara bioscience) (Table S5). The obtained PCR fragments with *Bam*HI and *Xho*I overhangs were gel-purified and then cloned in the *Bam*HI and *Xho*I digested VIGS vector pTRV2-*Cp*MgChl, giving pTRV2-*Cp*MMAT, pTRV2-*Cp*MMCC, and pTRV2-*Cp*MCO constructs. The constructed plasmids were sequence verified by Sanger sequencing and then used to transform *A. tumefaciens* GV3101 cells by electroporation. Transformed bacterial cells were plated on LB agar containing 50 mg/L of kanamycin, 25 mg/L of gentamycin, and 100 mg/L of rifampicin, and plates were incubated at 28 °C for 2 days. *Agrobacterium* transformant single colonies were grown overnight in LB medium supplemented with the same antibiotics and were further verified by Sanger sequencing of re-isolated plasmids. Aliquots were stored as 24% glycerol stocks at –80 °C.

VIGS inoculum preparation and *C. pubescens* plantlets transformation. *A. tumefaciens* GV3101 carrying pTRV2-*Cp*MgChl, pTRV2-*Cp*MMAT, pTRV2-*Cp*MMCC, pTRV2-*Cp*MCO, and pTRV1[45] plasmids were separately grown overnight in 3 mL of LB medium supplemented with 50 mg/L kanamycin, 25 mg/L gentamicin and 100 mg/L rifampicin at 28 °C and 300 rpm to an OD_{600} of approximately 2. Cells were pelleted for 10 min at 3,500 × *g*, resuspended in infiltration buffer (10 mM MES, 100 µM acetosyringone, 10 mM NaCl and 1.75 mM $CaCl_2$) to an OD_{600} of 2.0, covered with an aluminum foil and placed on a rotary shaker at room temperature and 60 rpm. After 2 h of incubation, 450 µL of each bacterial strain containing a pTRV2 plasmid were mixed with the same volume of the bacterial strain harboring pTRV1. These inoculums were used to transform sterile 2-month-old *C. pubescens* plantlets, germinated *in vitro* as previously reported[2]. For this, the plantlet stem was first pierced between the nodes of the two top leaf pairs with a ø 0.40 x 25 mm Sterican needle. The inoculum (10 µL) was then pipetted at the created hole and further infiltrated into the stem by gently pulling and pushing the needle 3-4 times. These procedures were performed rapidly in a sterile fume hood to minimize plantlet collapse and microbial contaminations. After VIGS inoculation, treated plantlets were

placed in a growth chamber at 25 °C, with a 16 h/8 h photoperiod under LED light and 70% humidity. The first VIGS symptoms (bleaching or yellowing of the leaves due to magnesium chelatase subunit H gene silencing) could be seen after 12 days. The plantlets inoculated with pTRV2-*Cp*MgChl strain served as positive controls for the bleaching phenotype and negative controls for target gene silencing. VIGS experiments were also conducted with non-sterile 4-month-old plants propagated on sand, and similar results were obtained.

VIGS tissues harvesting and samples preparation for LC-MS and qPCR analyses. *C. pubescens* plantlets infected with the VIGS constructs, pTRV2-*Cp*MgChl and pTRV2-*Cp*MgChl-insert, and whose leaves showed the photobleached phenotype, were collected 21 days post infection. The bleached areas were excised and the remaining green tissues discarded. The collected leaf affected areas from each VIGS-treated individual were pooled together, flash frozen in liquid nitrogen, ground with a Qiagen TissueLyser II, and stored at –80 °C. For LC-MS analysis, samples were aliquoted, mixed with methanol and sonicated for 10 min. The volume of methanol was normalized on fresh tissue weight to have a concentration of 10 mg/mL. Sonicated mixtures were centrifuged at 15,000 rpm for 5 minutes and the supernatant filtered with Macherey-Nagel CHROMAFIL polytetrafluoroethylene syringe filters (0.2 µm pore size, Ø 3 mm). The resulting methanolic extracts were analyzed by untargeted UPLC-MS (Method 1). The remaining fresh VIGS leaf powder was used to extract total RNA using RNeasy Plant Mini Kit (Qiagen), which was then converted to cDNA with SuperScript IV VILO reverse transcriptase (Thermo Fisher Scientific) for qPCR analysis.

qPCR analysis. The qPCR experiment was performed using the delta delta cycle method, as previously described[47]. Actin and the methyltransferase N2227 gene sequences were retrieved in *C. pubescens* transcriptome by BLAST-search and used as reference genes for expression normalization. Both references gave similar results. Gene-specific primers designed to target 100-130 bp of the gene of interest are listed in Table S7. The efficiency of these qPCR primers was between 95–100%. Gene fragment PCR-amplification was performed with Fast SYBR Green Master Mix (ThermoFischer Scientific) on a QuantStudio 1 cycler (Applied Biosystems). Statistical analysis (two-sided, unpaired Welch's t-test, or One-way ANOVA with post-hoc Tukey test) was performed using OriginPro (version 2023).

Phylogenetic analysis

Phylogenetic trees were generated in MEGA11[52]. The Maximum Likelihood method and Poisson correction model[53] were used to infer the evolutionary history. Initial tree(s) for the heuristic search were obtained automatically by applying Neighbor-Join and BioNJ algorithms to a matrix of pairwise

distances estimated using the Poisson model, and then selecting the topology with superior log likelihood value. The tree was drawn to scale, with branch lengths measured in the number of substitutions per site. Sequences alignment was performed with Clustal Omega[54].

Synthesis of compounds

Synthesis of corynantheol (11) and d_5-corynantheol (11b).

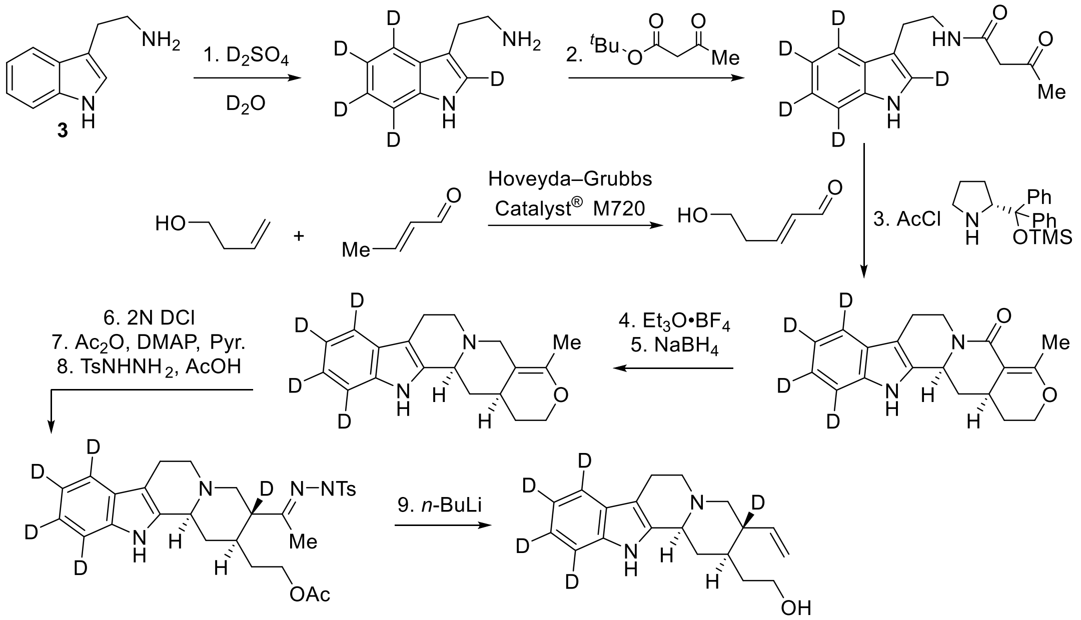

d_5-corynantheol (**11b**)

The synthesis of d_5-corynantheol (**11b**) was carried out from d_5-tryptamine,[55] through a reported procedure.[56] The unlabeled corynantheol (**11**) was prepared analogously, omitting the deuteration steps (step 1 and step 6). The NMR spectra of corynantheol (**11**) were consistent with the literature,[57] and a comparison of its ^{1}H NMR spectrum with that of d_5-corynantheol (**11b**) is shown in Supplementary Fig. 37 and the ^{1}H–^{13}C HSQC spectrum of the d_5-labeled corynantheol (**11b**) is also provided (see Supplementary Fig. 38). 1D and 2D NMR spectra of corynantheol (**11**) in methanol-d_3 are shown (Supplementary Fig. 39-S45, Table S8).

Synthesis of cinchonium (12).

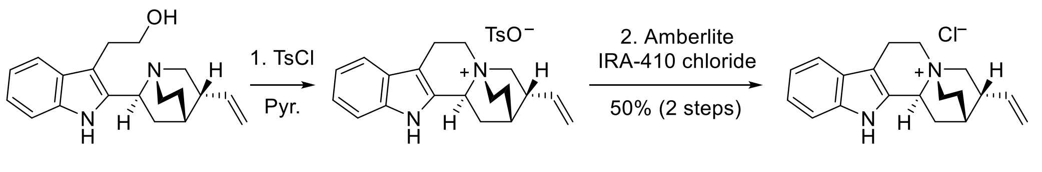

cinchonamine (**10**) cinchonium tosylate cinchonium chloride (**12**)

Tosyl chloride (152.5 mg, 0.80 mmol, 2 equiv) was added to a solution of cinchonamine (118.6 mg, 0.40 mmol, 1 equiv) in anhydrous pyridine (4.0 mL, 0.1 M) at 0 °C under argon atmosphere. The resulting mixture was stirred in a cold room maintained at 4 °C, resulting a formation of white precipitate. After 48 h, methanol (5 mL) was added to the reaction mixture to quench excess tosyl chloride, and volatiles were evaporated through azeotrope with toluene (3 × 25 mL). Acetone (25 mL) was added to the mixture to precipitate out the cinchonium tosylate, and the mixture was filtered through

a pad of celite, followed by washing with acetone (50 mL). Cinchonium tosylate was eluted by using a boiling hot methanol (total 100 mL), and concentrated to give pale yellow powder. Finally, 5.0 g of ion exchange resin (Amberlite IRA-410 chloride form) in methanol (10 mL) was added to the solution of cinchonium tosylate and the mixture was stirred for 15 min, then filtered through a pad of celite. Evaporation of the solvent afforded the title compound as a chloride salt (**12**, 63.5 mg, 50% yield, white powder).

^{1}H NMR (400 MHz, methanol-d_4): δ 7.49 (dt, J = 7.9, 1.0 Hz, 1H), 7.40 (dt, J = 8.2, 0.9 Hz, 1H), 7.17 (ddd, J = 8.2, 7.0, 1.2 Hz, 1H), 7.08 (ddd, J = 8.1, 7.1, 1.0 Hz, 1H), 6.16 (ddd, J = 17.3, 10.5, 6.9 Hz, 1H), 5.39 (dt, J = 17.2, 1.2 Hz, 1H), 5.34 (dt, J = 10.5, 1.2 Hz, 1H), 4.98 (t, J = 8.9 Hz, 1H), 3.95 (dd, J = 12.8, 10.4 Hz, 1H), 3.71 – 3.64 (m, 3H), 3.58 (dt, J = 12.9, 3.3 Hz, 1H), 3.38 (dddd, J = 12.6, 10.8, 3.2, 1.8 Hz, 1H), 3.16 (td, J = 8.8, 2.4 Hz, 1H), 3.10 – 3.00 (m, 2H), 2.89–2.77 (m, 1H), 2.35 – 2.28 (m, 1H), 2.07 (dddt, J = 12.9, 10.3, 7.8, 2.3 Hz, 1H), 2.00–1.93 (m, 2H).
^{13}C NMR (100 MHz, methanol-d_4): δ 139.4, 138.9, 129.6, 127.0, 123.8, 121.0, 119.4, 118.1, 112.8, 105.6, 63.5, 62.3, 61.3, 49.5*, 39.8, 27.7, 27.4, 25.8, 18.3.
* Overlaps with the signal of solvent, confirmed by ^{1}H–^{13}C HSQC. For the assignment of NMR signals, see Table S9. For NMR spectra, see Supplementary Fig. 46-51.
HRMS (ESI): Calculated for $C_{19}H_{23}N_2$, $[M+H]^+$: 279.1856, found: 279.1855.

Synthesis of cinchonaminal (7).

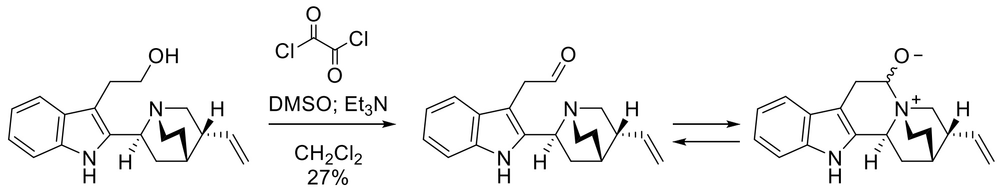

cinchonamine (**10**) cinchonaminal (**7**) (open form) cinchonaminal (**13**) (cyclized form)

Dimethyl sulfoxide (35 µL, 0.50 mmol, 2 equiv) was added to a solution of oxalyl chloride (21 µL, 0.25 mmol, 1 equiv) in anhydrous dichloromethane (2.5 mL, 0.1 M) at –84 °C (ethyl acetate / liq. N_2) under argon atmosphere. The resulting mixture was stirred at –84 °C for 10 min, then cinchonamine (**10**, 74.1 mg, 0.25 mmol, 1 equiv) in anhydrous dichloromethane / dimethyl sulfoxide (10 / 1, 2.2 mL) was added dropwise over 5 min at –84 °C. After 30 min, triethylamine (175 µL, 1.25 mmol, 5 equiv) was added dropwise over 5 min, and the reaction mixture was further stirred at –84 °C for 15 min. The reaction mixture was warmed to 23 °C, and stirred for 10 min. The reaction was quenched with brine (50 mL), and the aqueous layer was extracted with dichloromethane (3 × 50 mL). The combined organic layer was dried over anhydrous sodium sulfate, and filtered through cotton. The resulting filtrate was

concentrated under reduced pressure. The resulting crude mixture was purified by flash column chromatography (silica gel: diam. 1.5 cm, ht. 8 cm; eluent: ethyl acetate : methanol = 1 / 1) to afford cinchonaminal (20.0 mg, 27% yield) with a minor impurity as a pale yellow oil. Analytically pure compound was obtained through HPLC purification as described in the section ***Purification of Compounds by HPLC***. For NMR data, see Supplementary Fig. 52-59 and Table S10. **HRMS (ESI)**: Calculated for $C_{19}H_{23}N_2O$, $[M+H]^+$: 295.1805, found: 295.1799. Note: In solution, the title compound exists predominantly in the cyclized form (herein referred to as cyclocinchonaminal, **13**, further details in Supplementary Fig. 7).

NMR analysis

NMR spectra were recorded on a Bruker AV 400 Avance III HD, Bruker AV 500 Avance III HD (cryoprobe), or Bruker AV 700 Avance III HD (cryoprobe) spectrometer. Methanol-d_4, methanol-d_3, chloroform-d_3, acetonitrile-d_3 or DMSO-d_6 were used as solvents. NMR spectra were calibrated using the residual solvent signals as the internal reference (δ_H 3.31 ppm and δ_C 49.15 ppm for methanol-d_3 and methanol-d_4, δ_H 7.26 and δ_C 77.0 ppm for chloroform-d_3, δ_H 1.96 and δ_C 118.26 ppm for acetonitrile-d_3, and δ_H 2.50 and δ_C 39.52 ppm for DMSO-d_6). Measurements were performed at 298K, unless otherwise indicated. Data processing and analysis were performed with Bruker TopSpin ver. 3.6.1.

Supplementary list of compound names, structures, and numbers mentioned in this work.

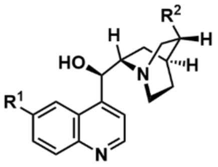

Cinchonidine (R^1 = H; R^2 = Vinyl), **1**
Quinine (R^1 = OMe; R^2 = Vinyl), **1a**
Dihydrocinchonidine (R^1 = H; R^2 = Et), **1'**
Dihydroquinine (R^1 = OMe; R^2 = Et), **1a'**

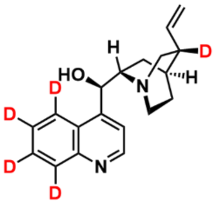

*d*5-Cinchonidine, **1b**

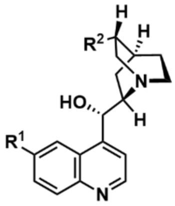

Cinchonine (R^1 = H; R^2 = Vinyl), **2**
Quinidine (R^1 = OMe; R^2 = Vinyl), **2a**
Dihydrocinchonine (R^1 = H; R^2 = Et), **2'**
Dihydroquinidine (R^1 = OMe; R^2 = Et), **2a'**

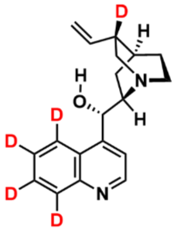

*d*5-Cinchonine, **2b**

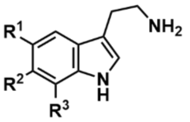

Tryptamine (R^1 = H; R^2 = H; R^3 = H), **3**
5-Methoxytryptamine (R^1 = OMe; R^2 = H; R^3 = H), **3a**
5-Fluorotryptamine (R^1 = F; R^2 = H; R^3 = H), **3b**
6-Fluorotryptamine (R^1 = H; R^2 = F; R^3 = H), **3c**
7-Fluorotryptamine (R^1 = H; R^2 = H; R^3 = F), **3d**
5-Chlorotryptamine (R^1 = Cl; R^2 = H; R^3 = H), **3e**
6-Chlorotryptamine (R^1 = H; R^2 = Cl; R^3 = H), **3f**
7-Chlorotryptamine (R^1 = H; R^2 = H; R^3 = Cl), **3g**

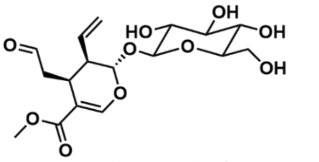

Secologanin, **4**

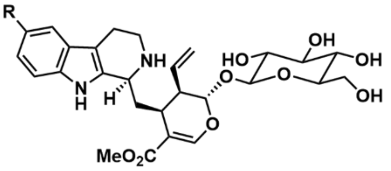

Strictosidine (R = H), **5**
10-Methoxystrictosidine (R = OMe), **5a**

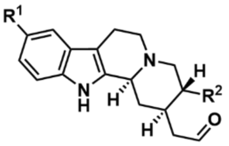

Corynantheal (R^1 = H; R^2 = Vinyl), **6**
10-Methoxycorynantheal (R^1 = OMe; R^2 = Vinyl), **6a**
Dihydrocorynantheal (R^1 = H; R^2 = Et), **6'**
10-Methoxydihydrocorynantheal (R^1 = OMe; R^2 = Et), **6a'**

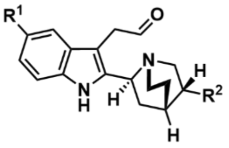

Cinchonaminal (R^1 = H; R^2 = Vinyl), **7**
10-Methoxycinchonaminal (R^1 = OMe; R^2 = Vinyl), **7a**
Dihydrocinchonaminal (R^1 = H; R^2 = Et), **7'**
10-Methoxydihydrocinchonaminal (R^1 = OMe; R^2 = Et), **7a'**

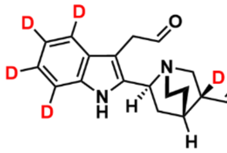

*d*5-Cinchonaminal, **7b**

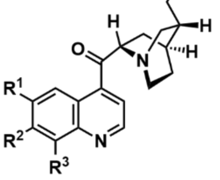

6-Fluorodihydrocinchonidinone (R^1 = F; R^2 = H; R^3 = H), **8c'**
7-Fluorodihydrocinchonidinone (R^1 = H; R^2 = F; R^3 = H), **8d'**
8-Fluorodihydrocinchonidinone (R^1 = H; R^2 = H; R^3 = F), **8e'**
6-Chlorodihydrocinchonidinone (R^1 = Cl; R^2 = H; R^3 = H), **8f'**
7-Chlorodihydrocinchonidinone (R^1 = H; R^2 = Cl; R^3 = H), **8g'**
8-Chlorodihydrocinchonidinone (R^1 = H; R^2 = H; R^3 = Cl), **8h'**

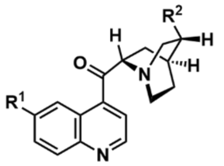

Cinchonidinone (R^1 = H; R^2 = Vinyl), **8**
Quininone (R^1 = OMe; R^2 = Vinyl), **8a**
Dihydrocinchonidinone (R^1 = H; R^2 = Et), **8'**
Dihydroquininone (R^1 = OMe; R^2 = Et), **8a'**

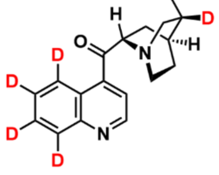

*d*5-Cinchonidinone, **8b**

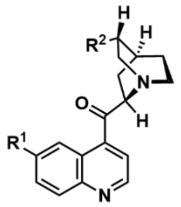

Cinchoninone (R^1 = H; R^2 = Vinyl), **9**
Quinidinone (R^1 = OMe; R^2 = Vinyl), **9a**
Dihydrocinchoninone (R^1 = H; R^2 = Et), **9'**
Dihydroquinidinone (R^1 = OMe; R^2 = Et), **9a'**

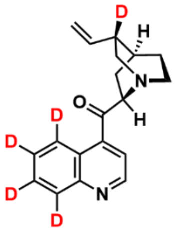

*d*5-Cinchoninone, **9b**

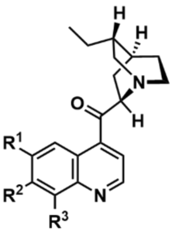

6-Fluorodihydrocinchoninone (R^1 = F; R^2 = H; R^3 = H), **9c'**
7-Fluorodihydrocinchoninone (R^1 = H; R^2 = F; R^3 = H), **9d'**
8-Fluorodihydrocinchoninone (R^1 = H; R^2 = H; R^3 = F), **9e'**
6-Chlorodihydrocinchoninone (R^1 = Cl; R^2 = H; R^3 = H), **9f'**
7-Chlorodihydrocinchoninone (R^1 = H; R^2 = Cl; R^3 = H), **9g'**
8-Chlorodihydrocinchoninone (R^1 = H; R^2 = H; R^3 = Cl), **9h'**

(continues on next page)

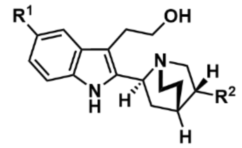

Cinchonamine (R^1 = H; R^2 = Vinyl), **10**
10-Methoxycinchonamine
(R^1 = OMe; R^2 = Vinyl), **10a**
Dihydrocinchonamine
(R^1 = H; R^2 = Et), **10'**
10-Methoxydihydrocinchonamine
(R^1 = OMe; R^2 = Et), **10a'**

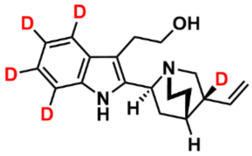

*d*5-Cinchonamine, **10b**

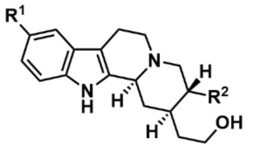

Corynantheol (R^1 = H; R^2 = Vinyl), **11**
10-Methoxycorynantheol
(R^1 = OMe; R^2 = Vinyl), **11a**
Dihydrocorynantheol
(R^1 = H; R^2 = Et), **11'**
10-Methoxydihydrocorynantheol
(R^1 = OMe; R^2 = Et), **11a'**

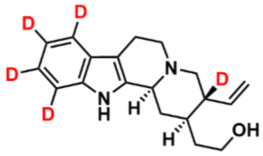

*d*5-Corynantheol, **11b**

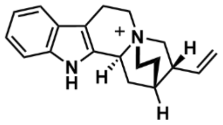

Cinchonium (R^1 = H; R^2 = Vinyl), **12**
10-Methoxycinchonium
(R^1 = OMe; R^2 = Vinyl), **12a**
Dihydrocinchonium
(R^1 = H; R^2 = Et), **12'**
10-Methoxydihydrocinchonium
(R^1 = OMe; R^2 = Et), **12a'**

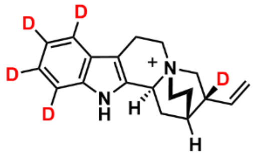

*d*5-Cinchonium, **12b**

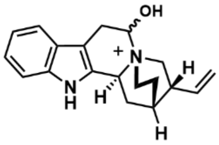

Cyclocinchonaminal (R^1 = H; R^2 = Vinyl), **13**
10-Methoxycyclocinchonaminal
(R^1 = OMe; R^2 = Vinyl), **13a**
Dihydrocyclocinchonaminal
(R^1 = H; R^2 = Et), **13'**
10-Methoxydihydrocyclocinchonaminal
(R^1 = OMe; R^2 = Et), **13a'**

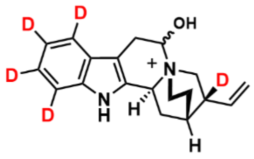

*d*5-Cyclocinchonaminal, **13b**

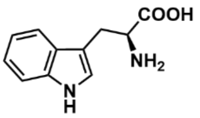

L-Tryptophan, **16**

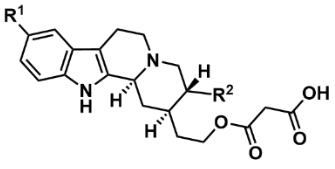

Malonyl-corynantheol
(R^1 = H; R^2 = Vinyl), **14**
10-Methoxymalonyl-corynantheol
(R^1 = OMe; R^2 = Vinyl), **14a**
Malonyl-dihydrocorynantheol (R^1 = H; R^2 = Et), **14'**
10-Methoxymalonyl-dihydrocorynantheol
(R^1 = OMe; R^2 = Et), **14a'**

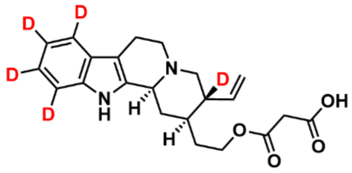

*d*5-Malonyl-corynantheol, **14b**

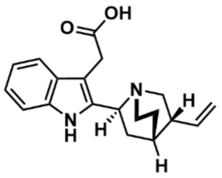

Cinchonaminic acid, **15**

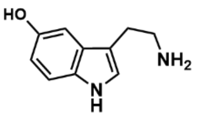

Serotonin, **17**

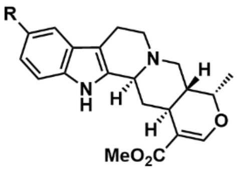

Tetrahydroalstonine, (R = H), **18**
Aricine (R = OMe), **18a**

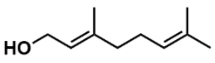

Geraniol, **19**

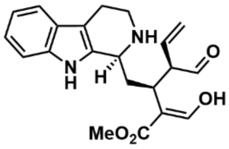

Strictosidine aglycone, **20**

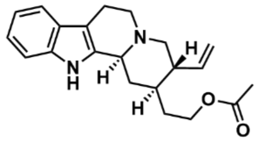

Acetyl-corynantheol, **21**

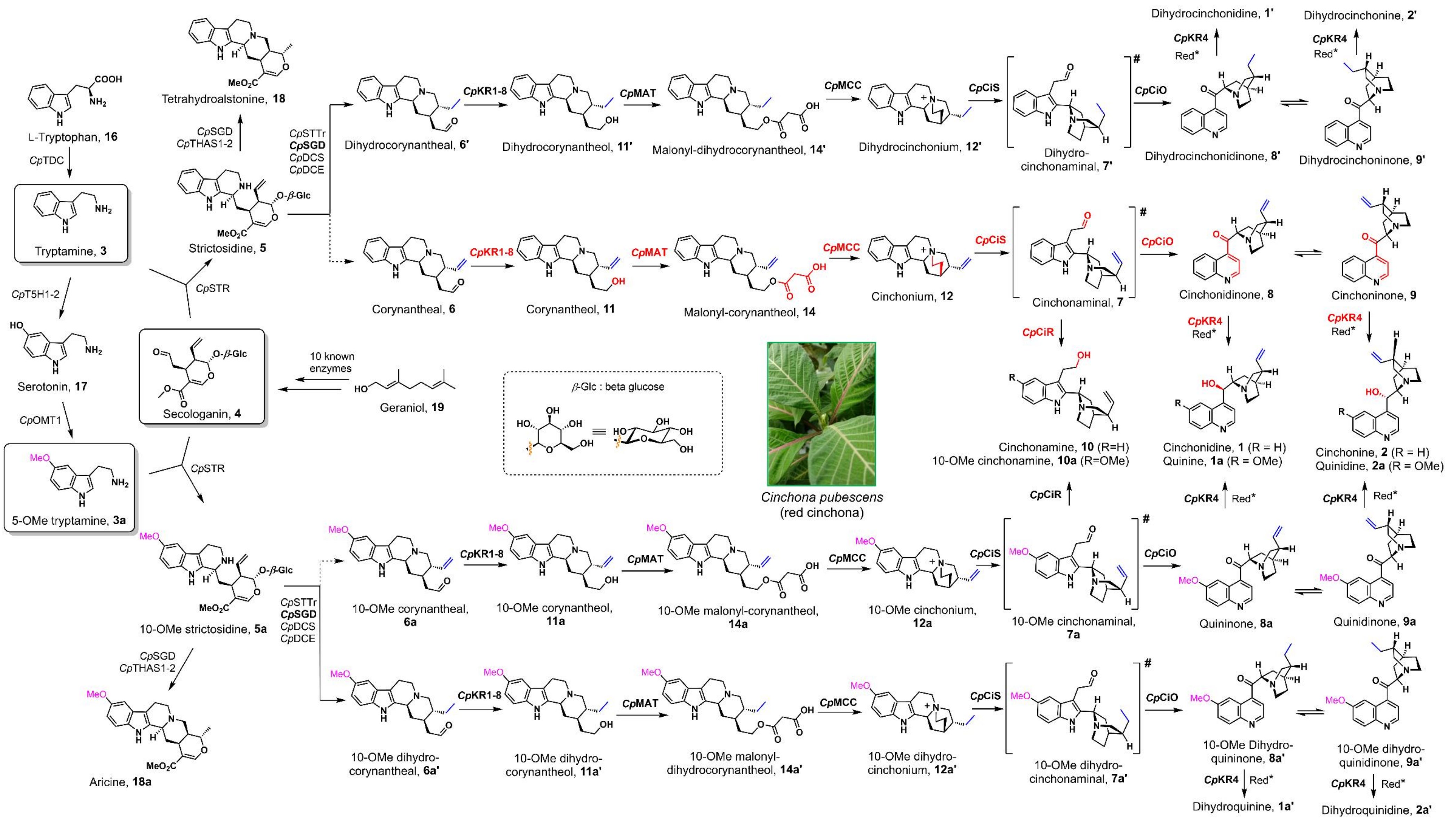

Supplementary Fig. 1. Biosynthetic pathways to Cinchona alkaloids. Enzymes reported herein are shown in bold, with key chemical transformations and involved enzymes highlighted in red for the pathway to cinchonidine (**1**) and cinchonine (**2**). Red* designates previously reported catalytic activity that has been observed in crude *Cinchona*

tissues, but the corresponding protein and gene remain undiscovered[58]. # indicates that the compound exists in a chemical equilibrium where the most stable and herein characterized structure is the cyclized hemiaminal form. The dashed arrows to corynantheal (**6**) and methoxylated analog (**6a**) denotes that the enzymatic formation to this dehydro congener is yet unknown.

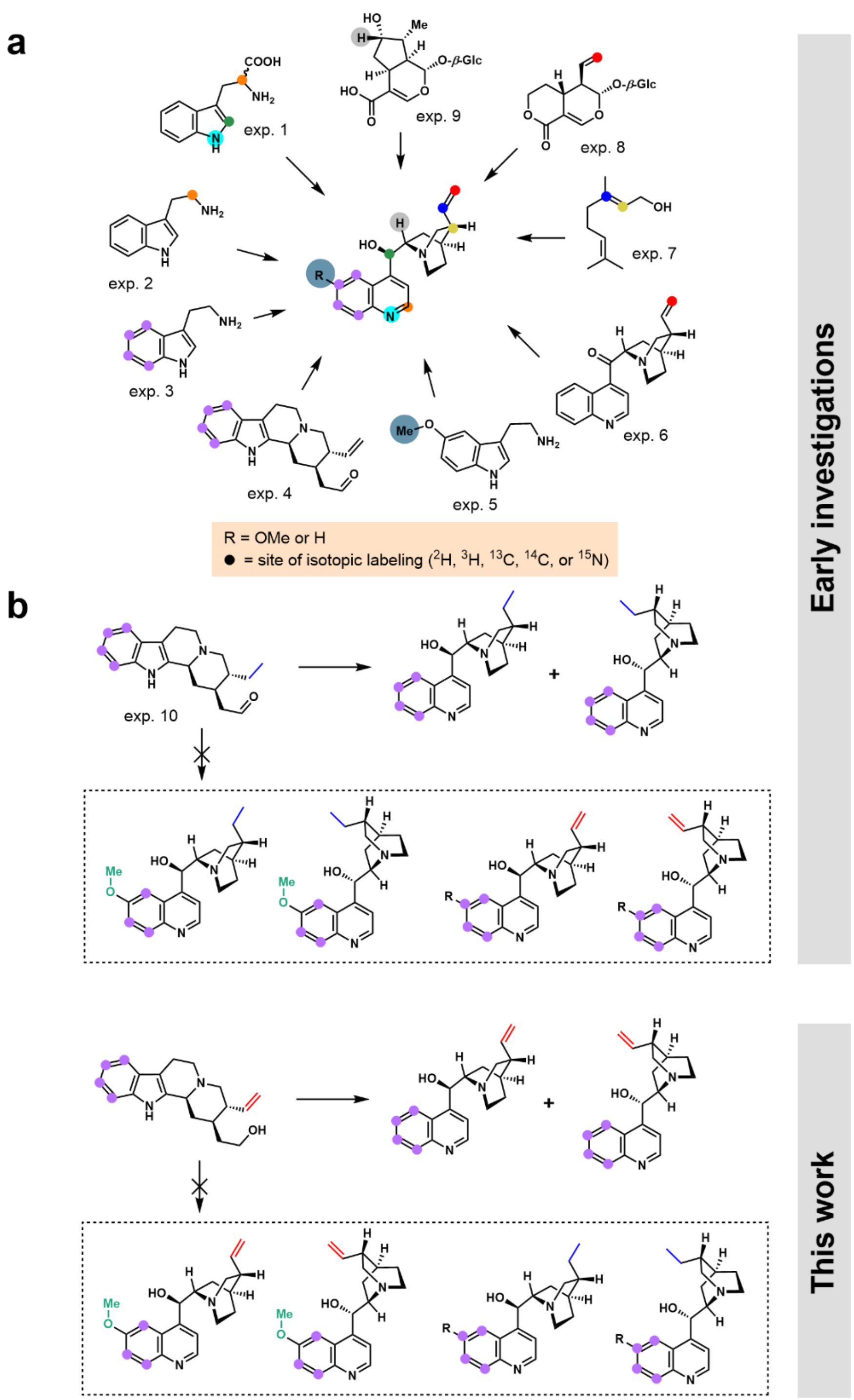

Supplementary Fig. 2. Summary of feeding experiments with labeled substrates. a. Selected experiments establishing the biosynthetic origin or connection of Cinchona alkaloid scaffolds, here representatively shown for quinine (R = OMe) and cinchonidine (R = H). Note that the order or numbering of the displayed experiments is arbitrary. For exp.1, see refs.[59-61]; exp. 2: ref.[62]; exp. 3: ref.[2]; exp. 4: ref.[63]; exp. 5: ref.[2] ; exp. 6: ref.[62]; exp. 7: refs.[64-66]; exp. 8: refs.[67,68]; and exp. 9: refs.[61,69]. **b**. Key experiments demonstrating that: 1) the splitting of pathways to alkaloids with a vinyl group (highlighted in red, e.g quinine and cinchonidine) and those with blue-highlighted ethyl group (the dihydro alkaloids, e.g. dihydroquinine and dihydrocinchonidine) occurs at the early stage, during the transformation of strictosidine to (dihydro)corynantheal (as shown in Supplementary Fig. 1); 2) the methoxy group present in alkaloids (dihydro)quinine and (dihydro)quinidine is introduced at the very early stage, the methoxylation of tryptamine (as indicated in Fig. 1 and Supplementary Fig. 1). For exp. 10, see ref.[2]

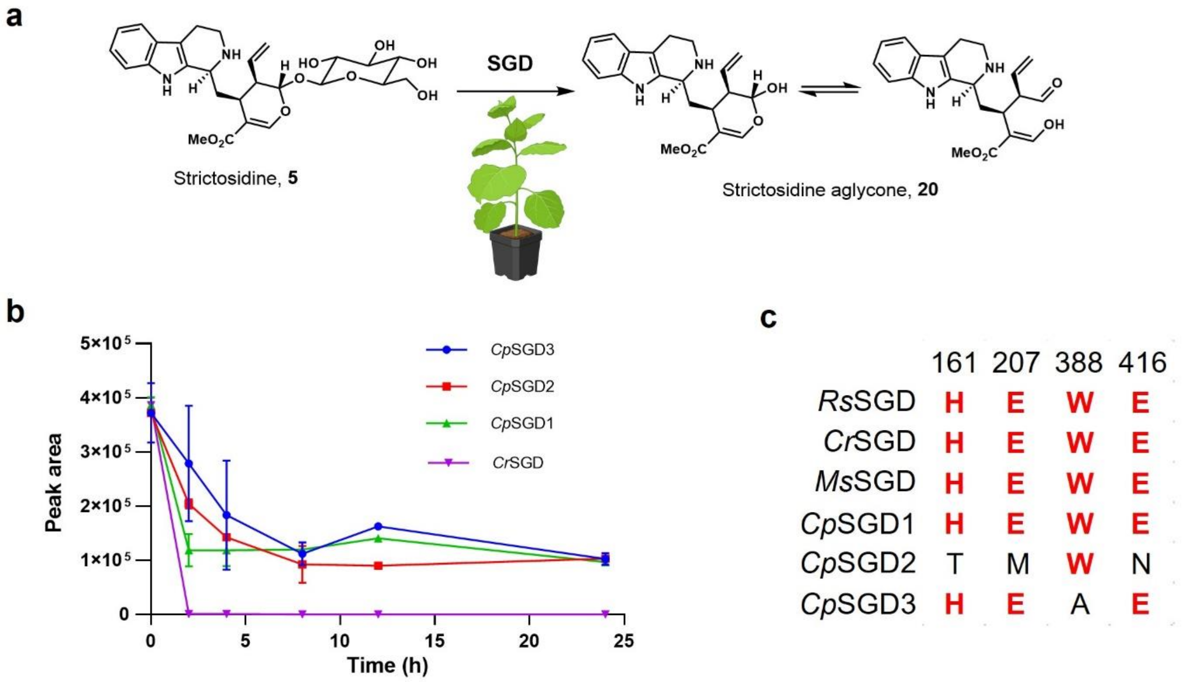

Supplementary Fig. 3. Identification of *C. pubescens* strictosidine *β*-glucosidases *Cp*SGD1-3. a. Reaction catalyzed by SGDs. **b.** Time-course analysis of strictosidine (**5**) deglycosylation by three identified *Cinchona* SGD, maned *Cp*SGD1-3, showing their low activity compared to the SGD from *Catharanthus roseus* (*Cr*SGD, GenBank ID: EU072423). Strictosidine mean peak areas ± s.d. measured at different time points are plotted ($n = 3$ replicates). **c.** Alignment of four key catalytic residues among *Cp*SGD1-3 candidates and three previously characterized SGDs: *Cr*SGD, *Rs*SGD (GenBank ID: AJ302044) from *Rauwolfia serpentina*, and *Ms*SGD (GenBank ID: OP800437) from *Mitragyna speciosa*.

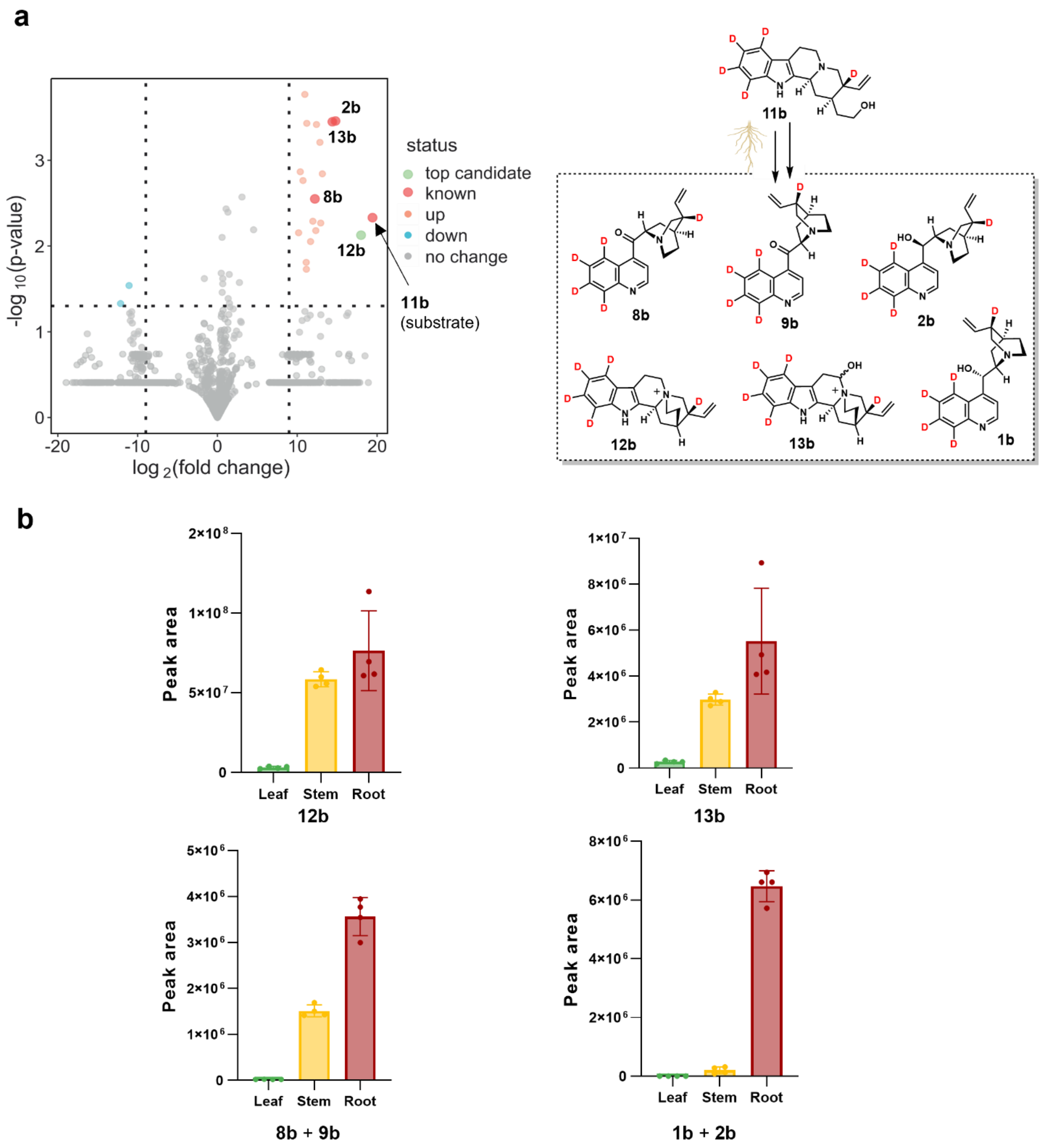

Supplementary Fig. 4. Untargeted metabolomics analysis of samples from feeding assays with d_5-corynantheol (11b). a. Volcano plot showing the accumulation of labeled metabolites in samples fed with **11b** compared to the water-fed controls (*n* = 4 per condition). The structures of some of the labeled compounds are shown, including the previously unknown compound **12** (herein described and named cinchonium) and cinchonaminal (herein shown to predominantly exist in the cyclized form **13**). **b.** Bar graphs showing the LC-MS peak areas of selected labeled products detected in different *C. pubescens* tissues after 24 h feeding, showing tissues incorporation performance. Root tissue was able to accumulate more labeled products compared to stem and leaf tissues. Data are presented as mean ± s.d. (*n* = 4 biological replicates).

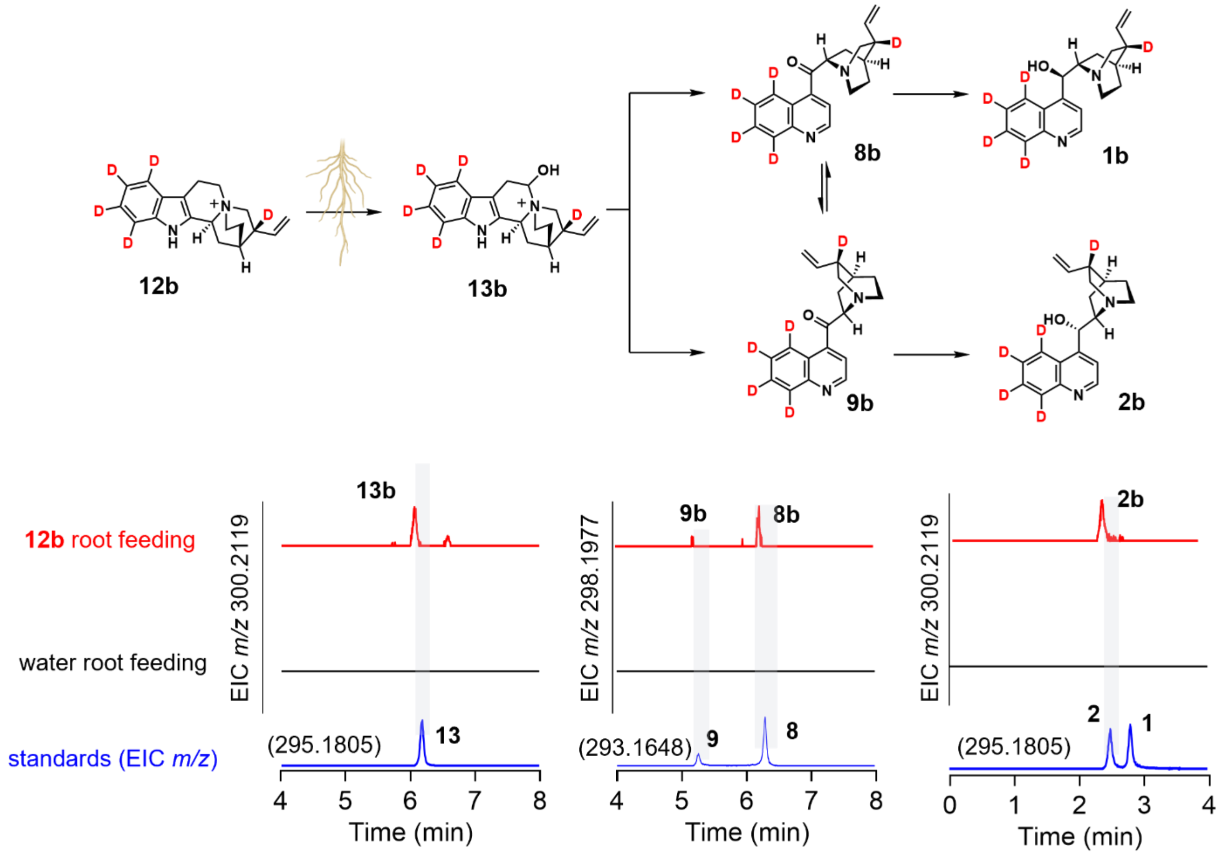

Supplementary Fig. 5. Incorporation of d_5-cinchonium (12b) into later-stage Cinchona alkaloids in root tissues after 48 h feeding. EICs of d_5-cyclocinchonaminal **13b**, d_5-cinchoni(di)none **8b**/**9b** and d_5-cinchoni(di)ne **1b**/**2b**, along with their unlabeled standards. This experiment was repeated three times with similar results.

a

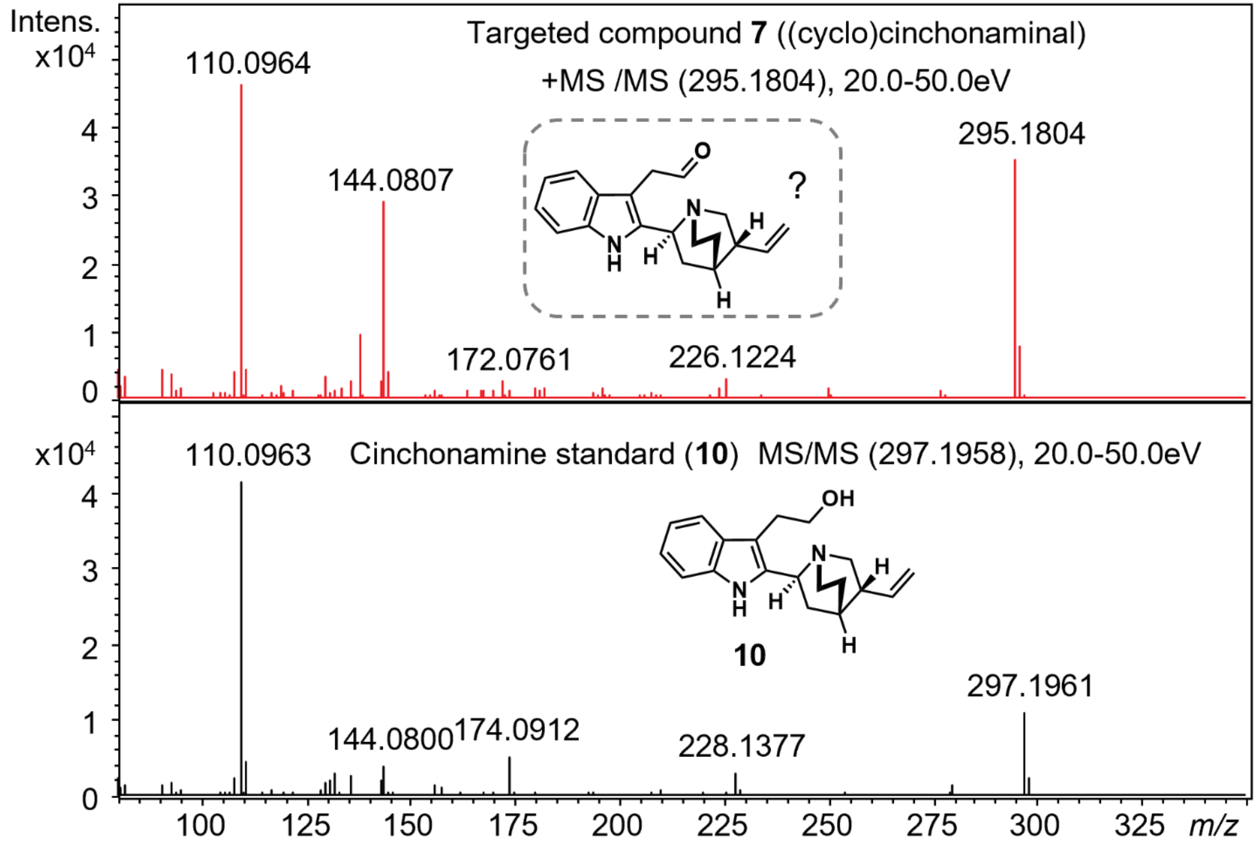

(continues on next page)

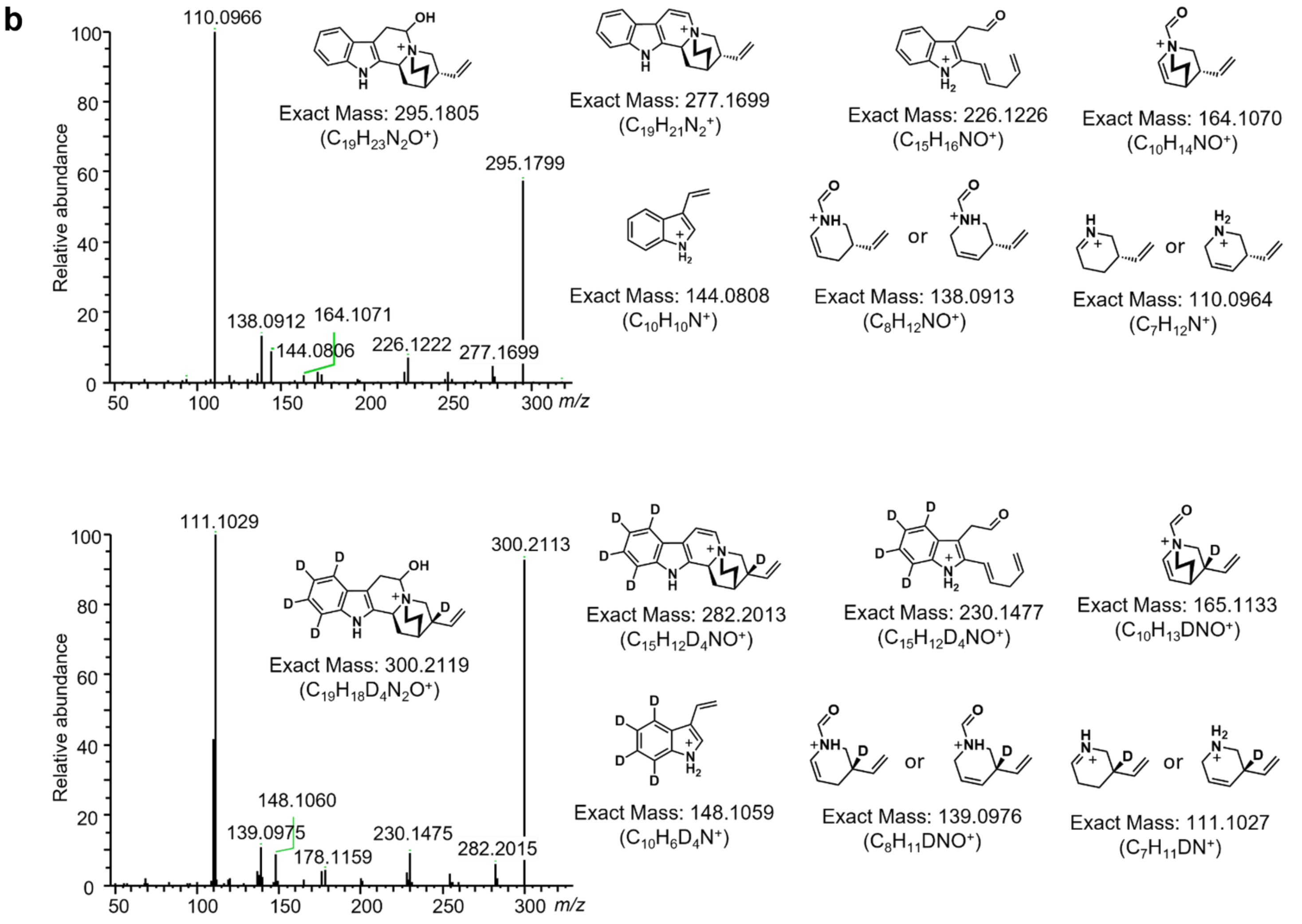

Supplementary Fig. 6. MS/MS spectra of cinchonamine and cinchonaminal. (a) Comparison of MS/MS spectra of the standard cinchonamine (**10**) and a target *Cinchona* metabolite, suggested it to be the putative intermediate cinchonaminal (**7**). **b)** MS/MS spectrum of purified cyclized cinchonaminal (cyclocinchonaminal) and the labeled analog d_5-cyclocinchonaminal (along with major putative MS/MS fragments).

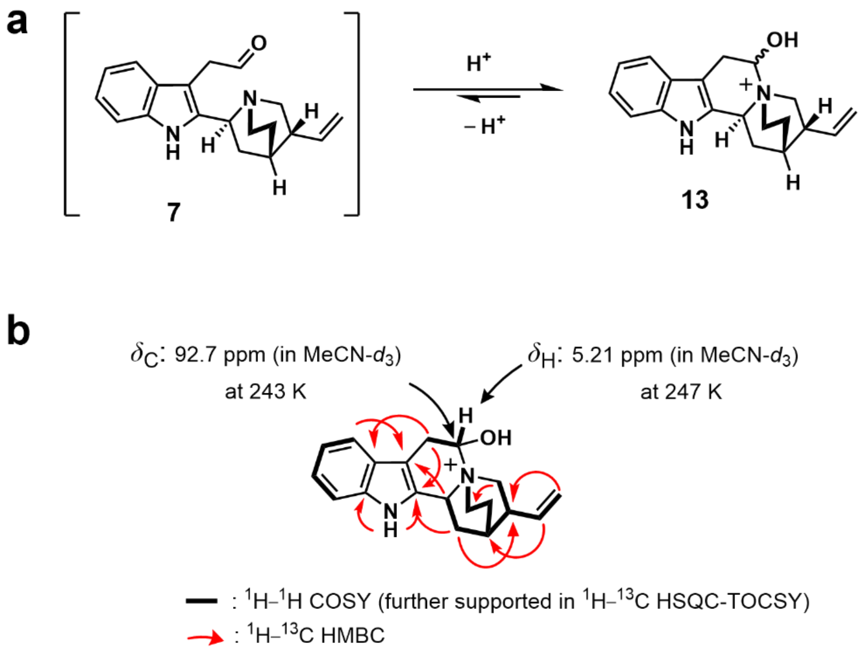

Supplementary Fig. 7. Structural characterization of cinchonaminal (7). a. In solution, cinchonaminal (**7**) exists in equilibrium between the open form (**7**) and the closed or cyclized form (**13**, herein named cyclocinchonaminal), with the latter being predominant. **b**. Selected NMR data supporting the structure of **13**. In NMR analyses of **7**, no aldehyde proton or carbonyl carbon could be detected. In contrast, hemiaminal proton and carbon signals could be detected (as indicated in **b**). The signal of the hemiaminal proton displayed no HSQC/HMBC, which is likely due to the rapid equilibrium between open and cyclized forms (see NMR spectra at 298 K in MeCN-d_3). Low-temperature measurements at 243 K in MeCN-d_3 revealed the HSQC correlation of the hemiaminal proton and carbon signals (Supplementary Fig. 52-59). The predominance of the cyclized form was further substantiated by the MS/MS fragmentation of **13** and the d_5-labeled analog **13b**, where putative fragments with amide functions were detected and which would not be generated if the compound was in the open form **7** (see *e.g.*, fragments with *m/z* 164.1070 and 138.0913, in Supplementary Fig. 6). Notably, the configuration of the hemiaminal stereocenter could not be determined due to the rapid equilibrium.

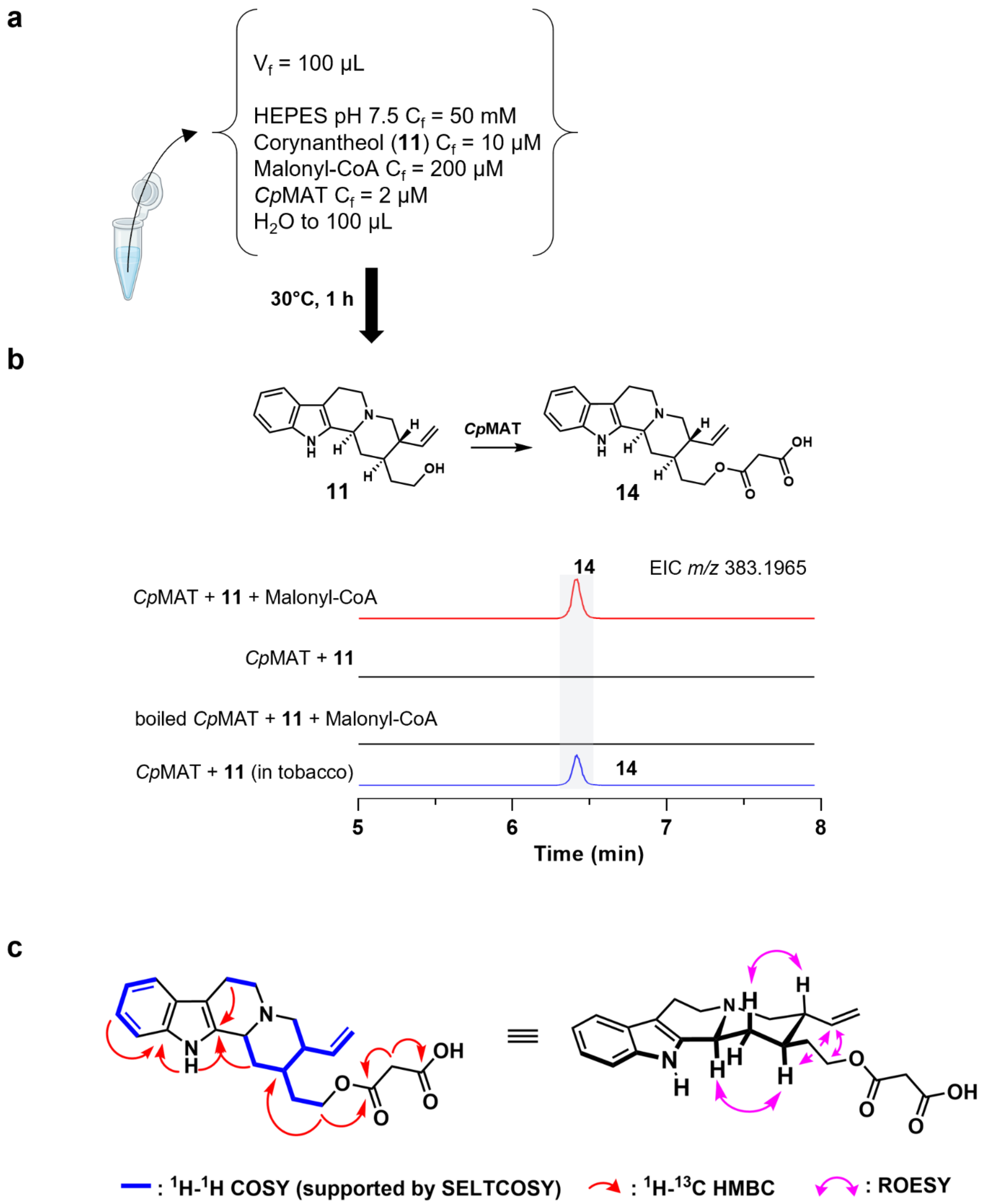

(continues on next page)

d

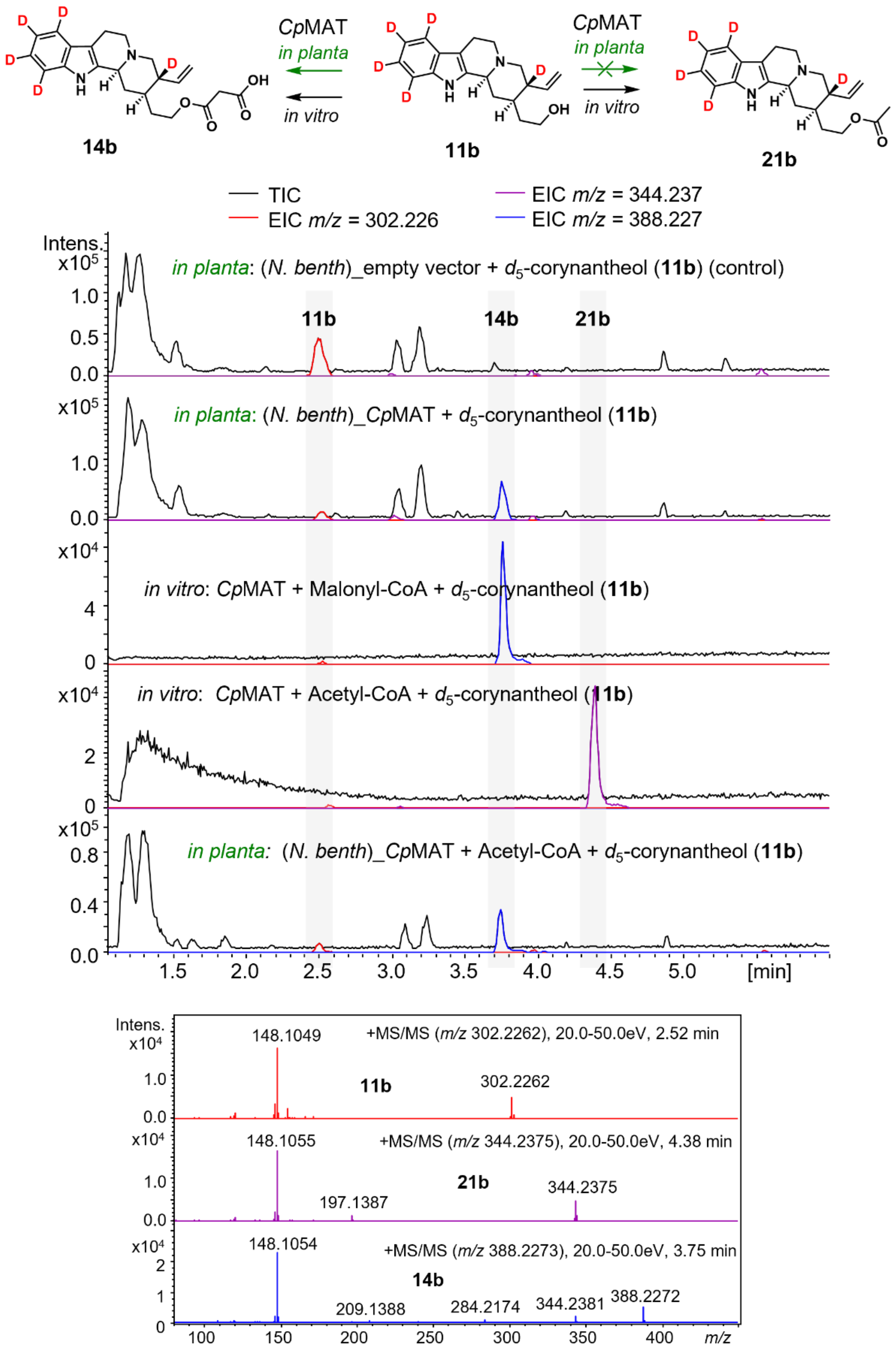

Supplementary Fig. 8. Additional data on *Cp*MAT activity and structural characterization of *Cp*MAT product. a. Schematic of the *in vitro* assay conditions for *Cp*MAT using corynantheol (**11**) as the substrate. **b.** Extracted ion chromatograms of malonyl-corynantheol (**14**) confirming the catalytic activity of *Cp*MAT *in vitro*. All assays were repeated independently three times with similar results. **c.** Selected NMR data demonstrating the *Cp*MAT product to be the *O*-malonylated derivative of corynantheol (herein referred to as malonyl-corynantheol, **14**). For NMR spectra, see Supplementary Fig. 60-70. **d)** Additional LC-MS data (TIC, EIC, and MS/MS) from assays *in vitro* and in *N. benthamiana* showing that *Cp*MAT malonylates d_5-corynantheol (**11b**) both *in vitro* and *in planta*, but it acetylates **11b** only *in vitro*, not *in planta*. These experiments were repeated three times with similar results.

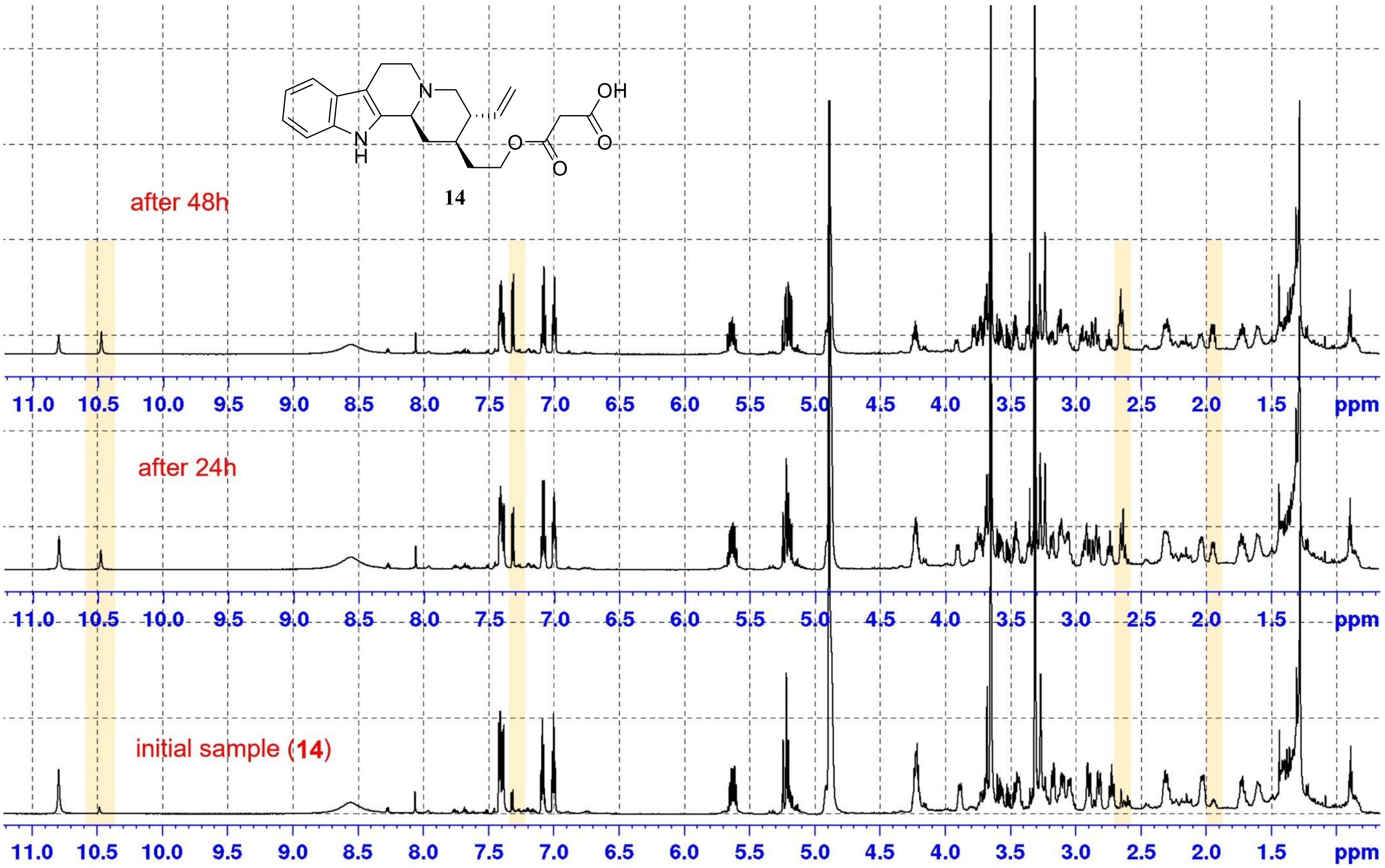

Supplementary Fig. 9. ^{1}H NMR spectra (700 MHz) of malonyl-corynantheol (14) in methanol-d_3 showing its degradation over time. Yellow-highlighted parts are selected regions showing an increase of peaks. For spectrum annotation or peaks assignment see Supplementary Fig. 67 or Table S11.

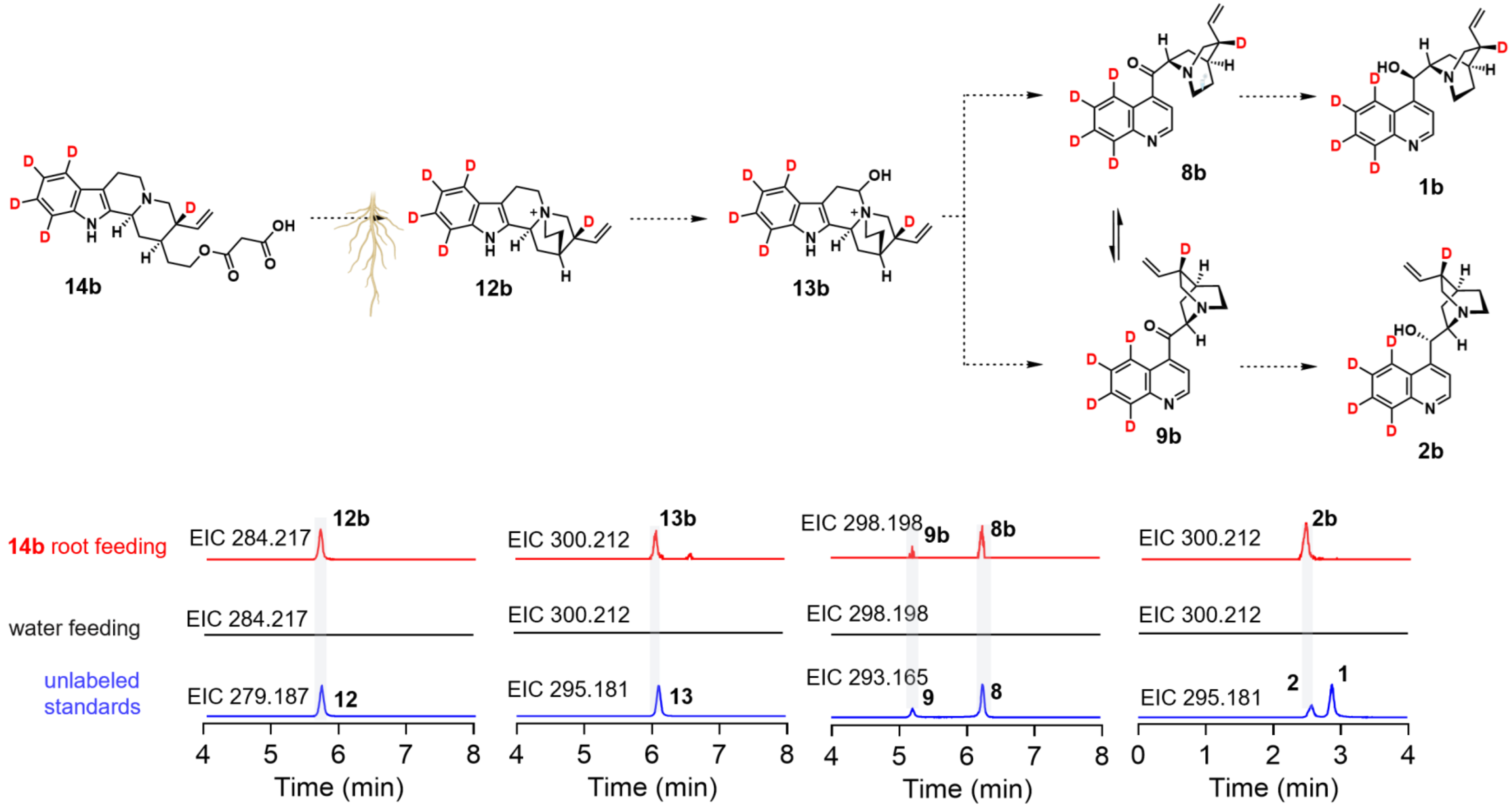

Supplementary Fig. 10. *C. pubescens* feeding with d_5-malonyl-corynantheol (14b). EICs showing the labelling pattern in downstream Cinchona alkaloids 48 h after feeding *C. pubescens* root tissues feeding with **14b**. This experiment was repeated three times with similar results.

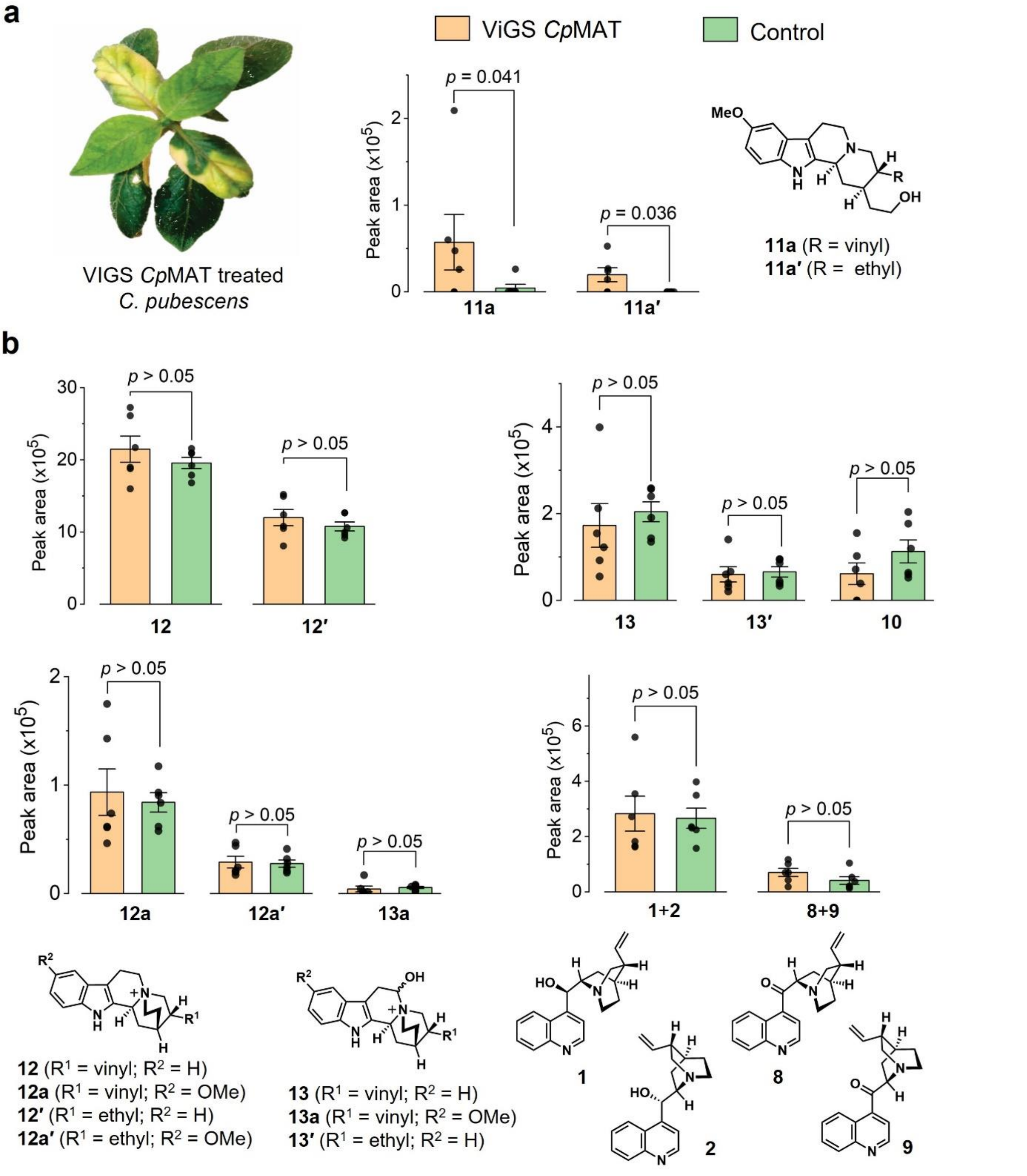

Supplementary Fig. 11. Additional results from *Cp*MAT silencing. **a**. Methoxylated corynantheol congeners **11a** and **11a′** accumulated in *Cp*MAT silenced tissues in comparison to controls, similarly to what observed for the non-methoxylated analogs (see Extended Data Fig. 1). **b.** Metabolites levels of selected downstream alkaloids did not change upon *CpMAT* silencing, likely due to the natural high abundance of the *Cp*MAT immediate downstream intermediate cinchonium (**12**) (and analogs). The values represent mean ± standard deviation (SD) ($n = 6$ biological replicates, p values present statistical analysis of means comparison using one-way ANOWA with post-hoc Tukey test).

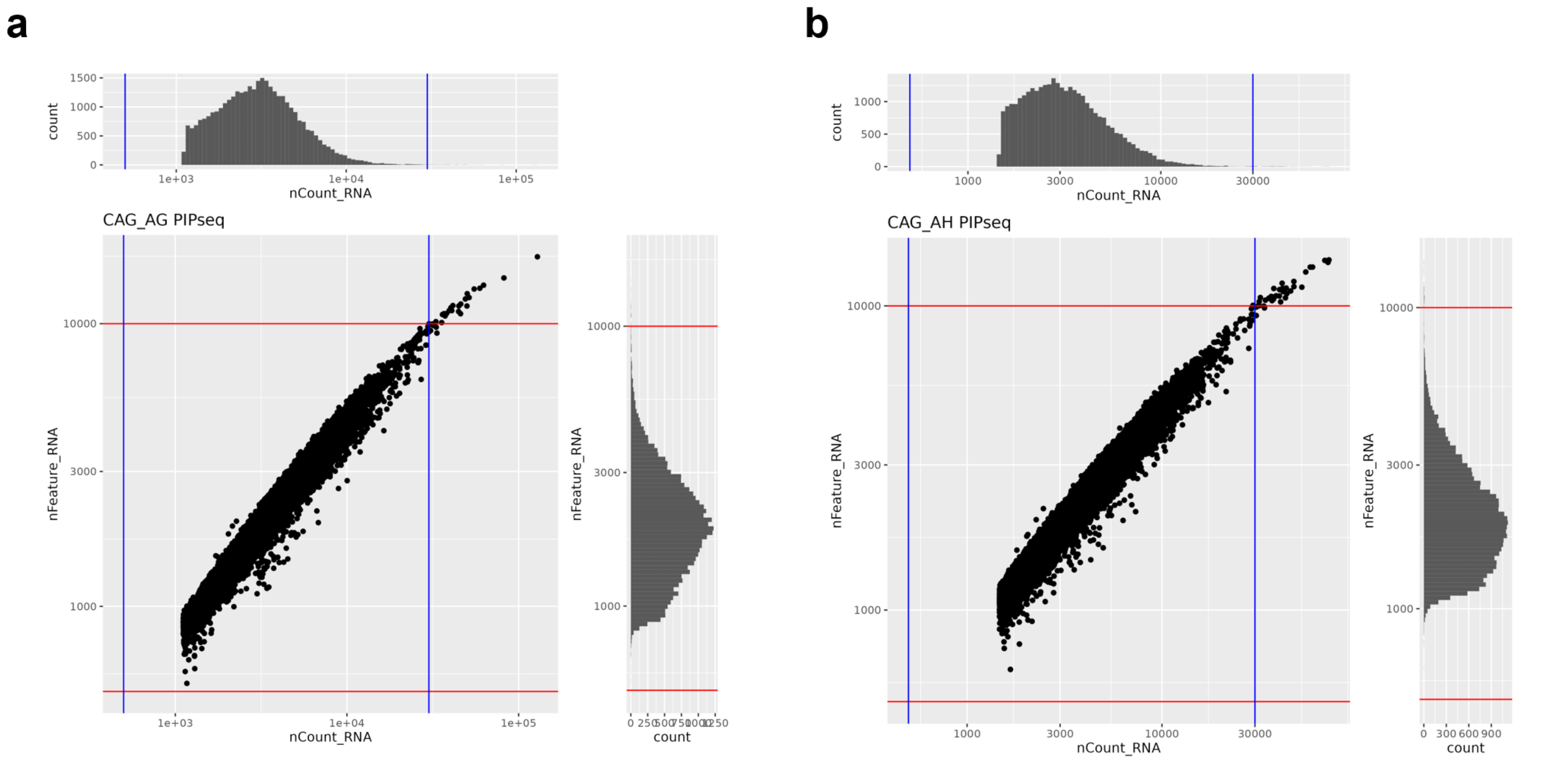

Supplementary Fig. 12. ***C. pubescens*** **leaf single nuclei RNA-seq libraries. a.** Scatter plots of nCount_RNA (UMI counts) and nFeature_RNA (genes detected) for replicate 1. **b.** Scatter plots of UMI counts and genes detected for replicate 2. Distributions of the UMI count and genes detected are shown above and to the right of the scatter plots. Nuclei between the red and blue lines passed quality filtering thresholds and were retained for downstream analysis.

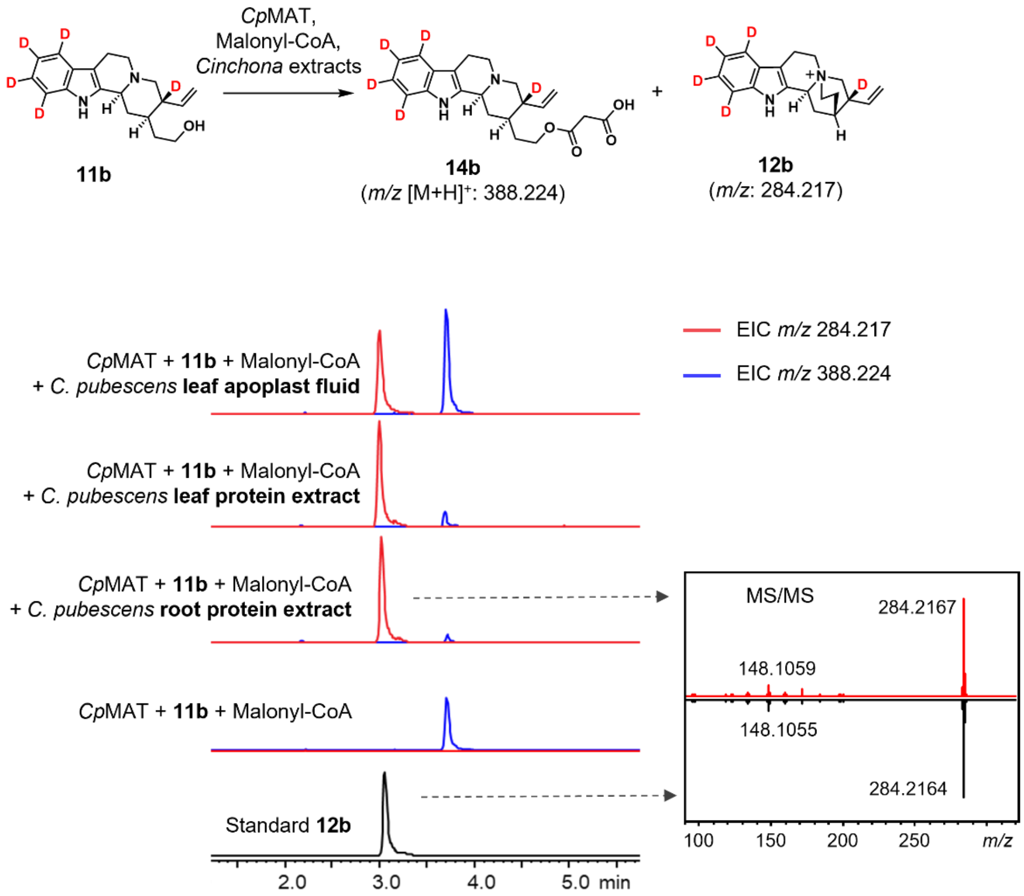

Supplementary Fig. 13. Malonyl-corynantheol cyclization activity of *Cinchona* protein extracts. Reaction scheme, extracted ion chromatograms (EIC), and MS/MS (20 to 50 eV) spectra showing that root and leaf crude protein extracts, as well as leaf apoplast fluid cyclize d_5-malonyl-corynantheol (**14b**) to d_5-cinchonium (**12b**). Assays were repeated three times with similar results.

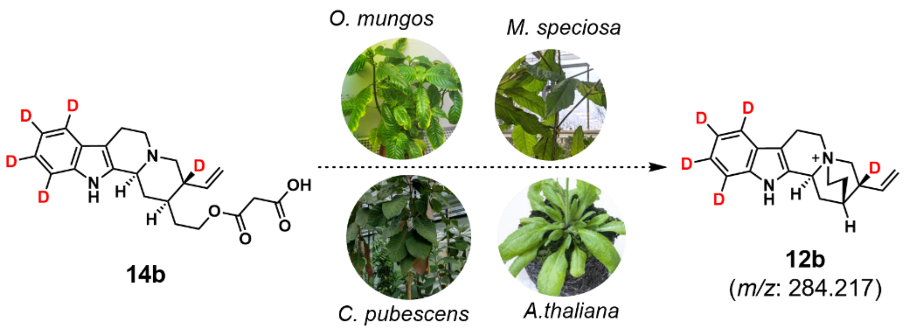

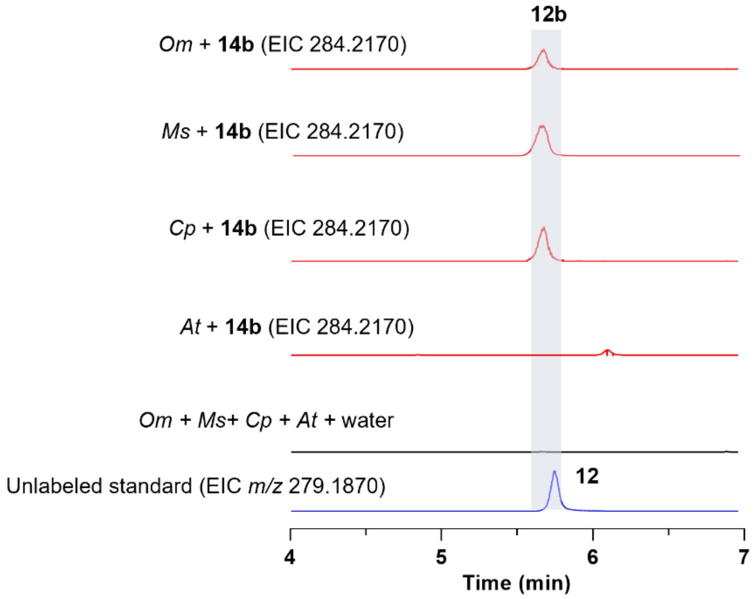

Supplementary Fig. 14. Conversion of d_5-malonyl-corynantheol into d_5-cinchonium by different plant species. EICs of d_5-cinchonium (**12b**) in d_5-malonyl-corynantheol (**14b**) feeding experiments with *C. pubescens* ("*Cp*"), *O. mungos* ("*Om*"), *M. speciosa* ("*Ms*"), and *A. thaliana* ("*At*"), along with the EIC of the unlabeled standard **12**. The slight change in retention time compared to the standard is due to the presence of the deuterium labels. This experiment was repeated three times with similar results.

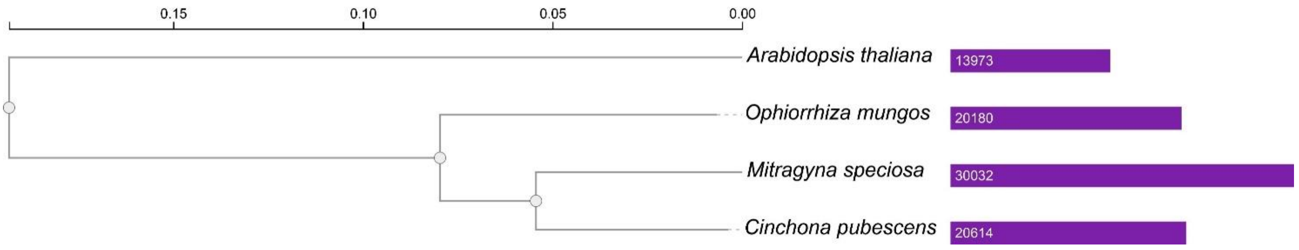

Supplementary Fig. 15. Phylogenetic tree of the four plants used in the cross-species comparison. The phylogenetic tree was generated using OrthoVenn 3 (https://orthovenn3.bioinfotoolkits.net) with the maximum likelihood method and the JTT + CAT model. The number of orthogroups identified in each species is shown to the right of the tree.

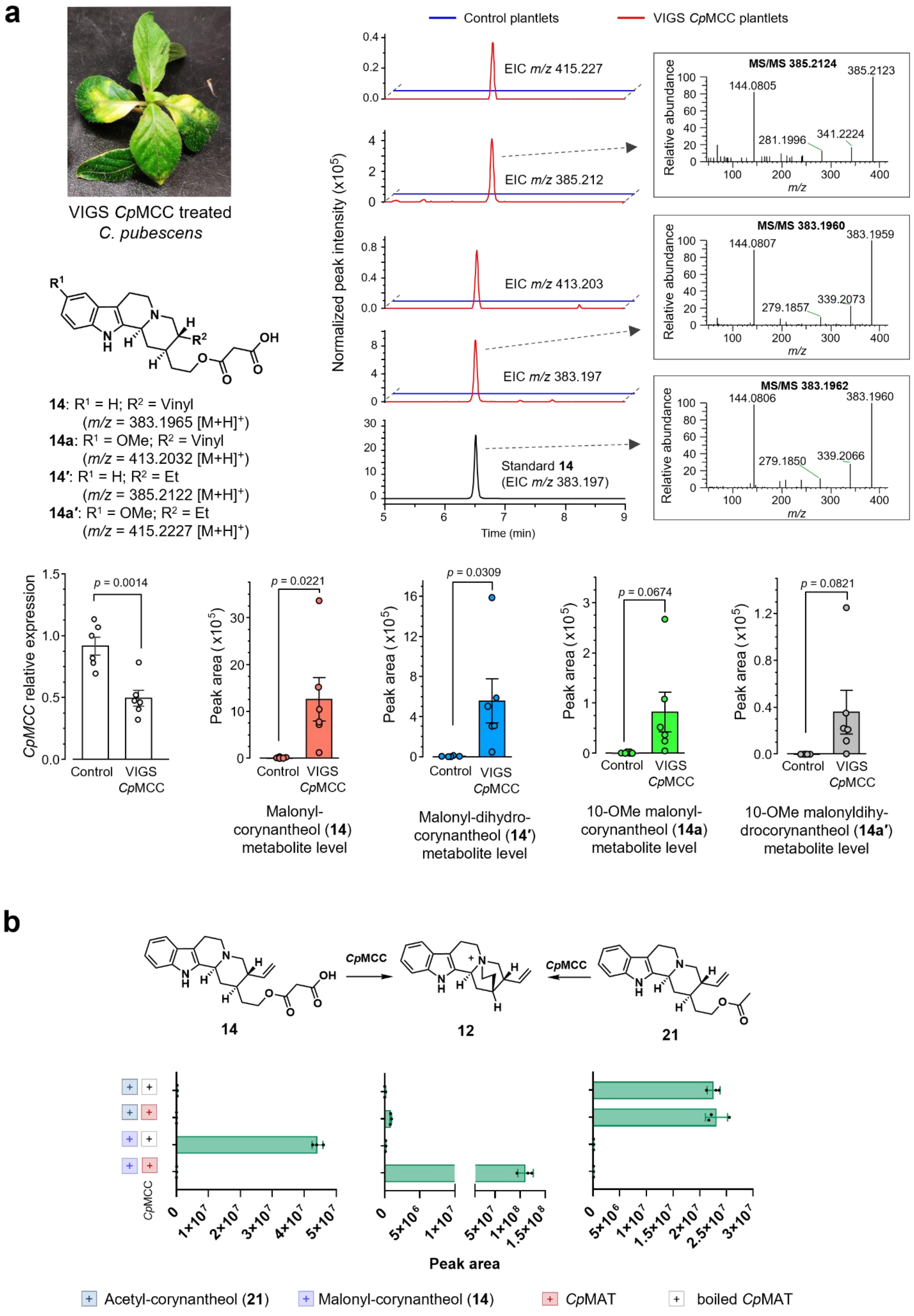

Supplementary Fig. 16. Additional results on *Cp*MCC functional characterization *in vivo* and *in vitro*. a. VIGS-*Cp*MCC-transformed *Cinchona* plantlet, extracted ion chromatograms (EIC), MS/MS spectra, relative

expression of *CpMCC,* and metabolites levels showing the accumulation of malonyl-corynantheol (**14**) and related dihydro and methoxylated congeners (**14′**, **14a** and **14a′**) in *Cp*MCC silenced tissues in comparison to controls. Bar graphs represent the values of the mean ± standard deviation (s.d.) (n = 6 biological replicates, p values present statistical analysis of means comparison using one-way ANOVA with post-hoc Tukey test). **b.** Assay to determine substrate preference of *Cp*MCC. LC-MS peak areas of cinchonium (**12**) from *in vitro* assays with either malonyl-corynantheol (**14**) or acetyl-corynantheol (**21**) as substrates. Data are presented as mean ± s.d. (n = 3 biological replicates).

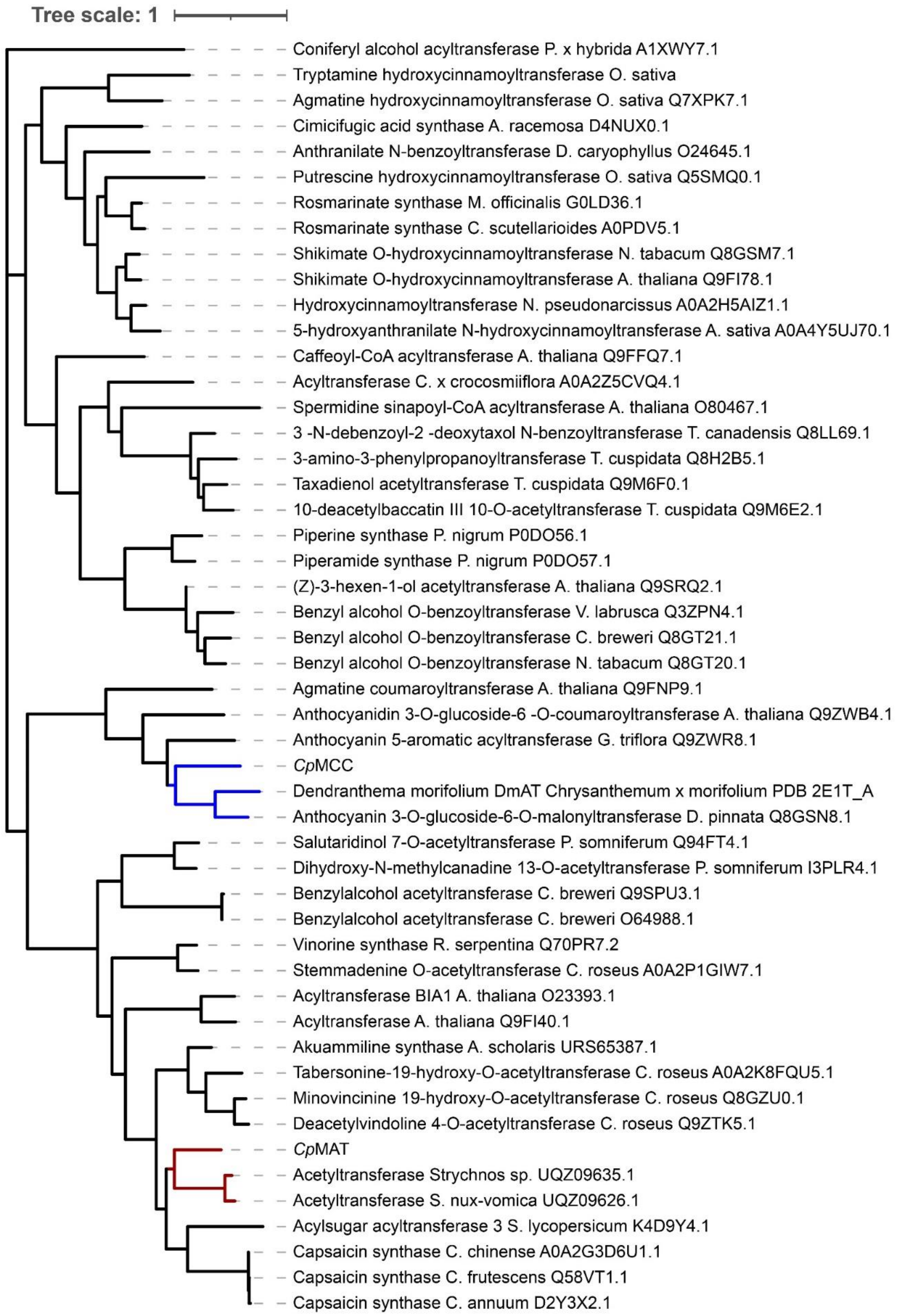

Supplementary Fig. 17. Phylogenetic tree of *Cp*MAT and *Cp*MCC with previously characterized BAHD acyltransferases. *Cp*MAT and its closest malonyltransferase homologs are highlighted in red, whilst *Cp*MCC and its closest malonyltransferase homologs are highlighted in blue.

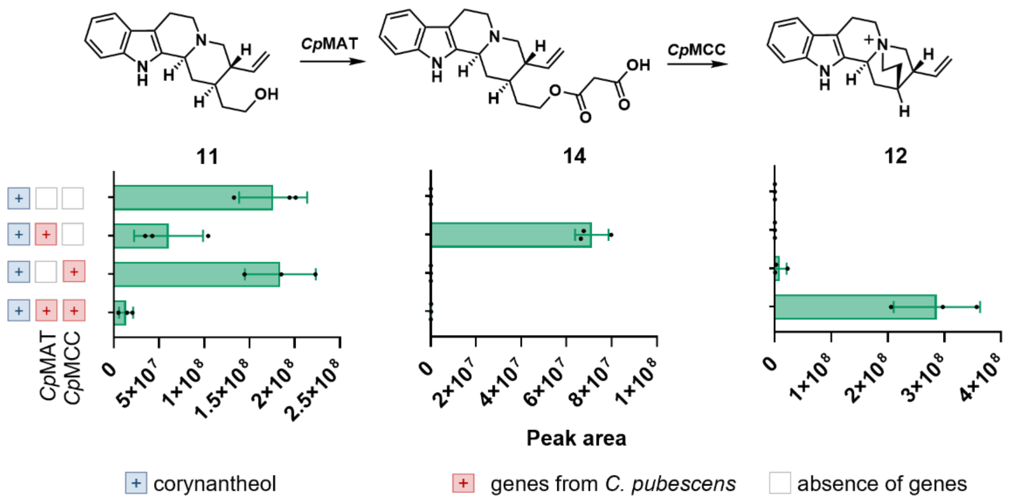

Supplementary Fig. 18. Enzymatic activity comparison between *Cp*MAT and *Cp*MCC. LC-MS peak areas of products detected in *N. benthamiana* following transient expression of the indicated biosynthetic enzymes and subsequent incubation with corynantheol (**11**) using a leaf-disc assay. Data are presented as mean ± s.d. (*n* = 3 biological replicates).

a

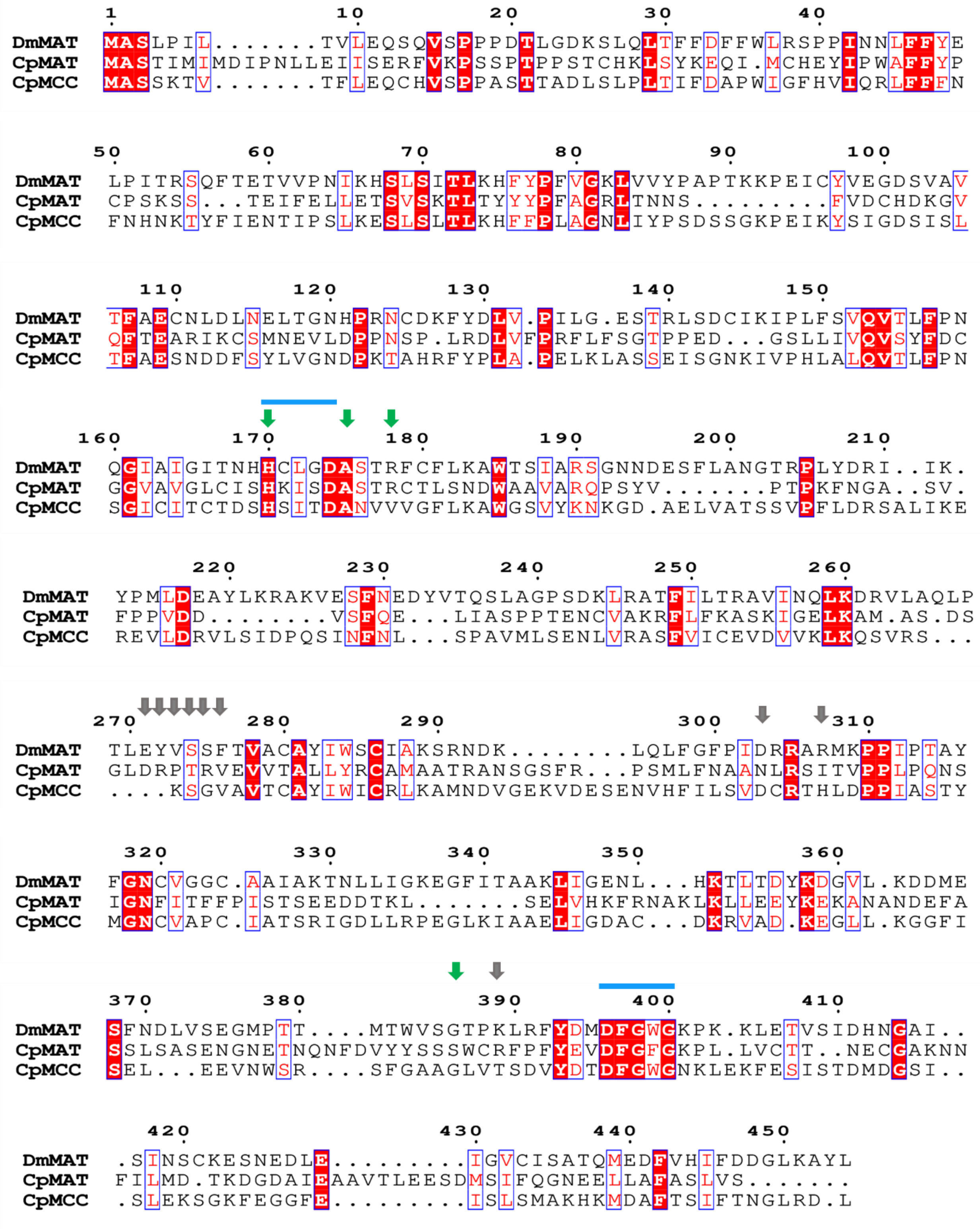

➡ : binding site to malonyl group of malonyl-CoA in *Dm*MAT

➡ : binding site to CoA moiety of malonyl-CoA in *Dm*MAT

▬ : canonical motifs in BAHD acyltransferases (HXXXD and DFGWG)

b

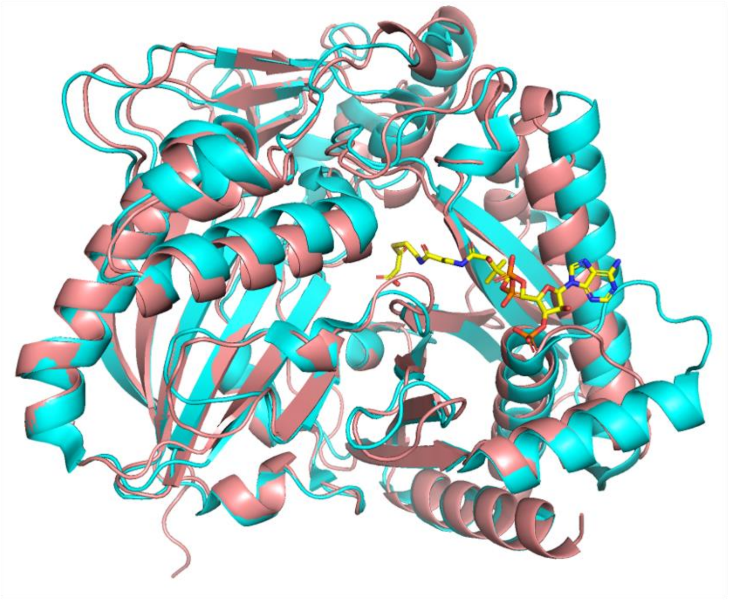

c

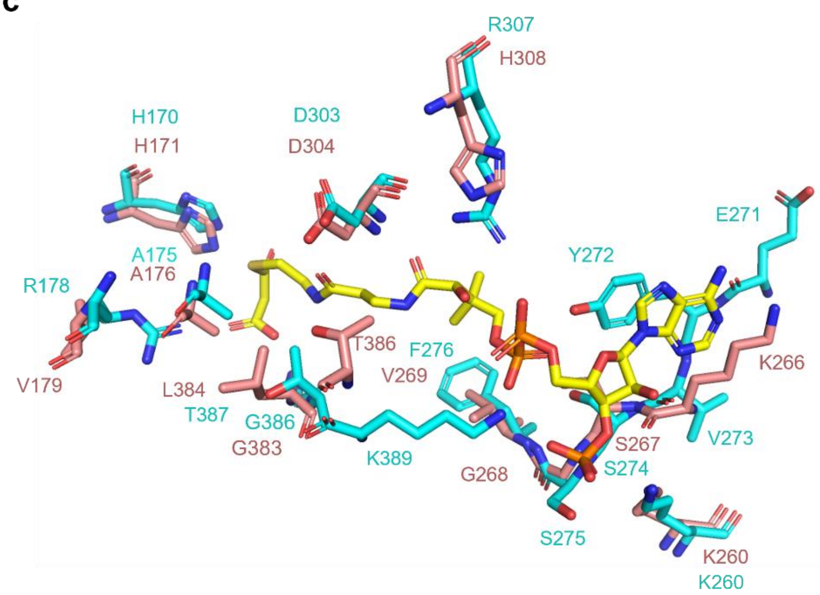

Supplementary Fig. 19. Comparison of *Cp*MAT and *Cp*MCC with a previously characterized BAHD malonyltransferase. a. Protein sequence alignment of *Cp*MAT, *Cp*MCC, and a well-known[70] malonyltransferase *Dm*MAT. *Dm*MAT is the closest homolog of *Cp*MCC (35.7 % Id.) for which a crystal structure is available (PDB: 2E1T). The binding sites to the cofactor malonyl-CoA in *Dm*MAT are indicated by green and gray arrows and the two canonical BAHD motifs are indicated with a blue line. Note in particular that the two important residues that fix the malonyl group via hydrogen bonds in *Dm*MAT (H170 and R178)[70] are conserved in *Cp*MAT, while a mutation is observed in *Cp*MCC. **b.** Structural overlay of *Cp*MCC model (in salmon) and the crystal model of *Dm*MAT (in cyan) in complex with the cofactor malonyl-CoA (in yellow). **c.** Residues responsible for the cofactor binding in *Dm*MAT (indicated in **a**) are highlighted (cyan), along with aligned residues in *Cp*MCC (in salmon). Muscle 5.1[71,72] was used for the protein sequences alignment, and ESPript V3 was used for alignment plotting[73], *Cp*MCC model was created with AlphaFold3[74].

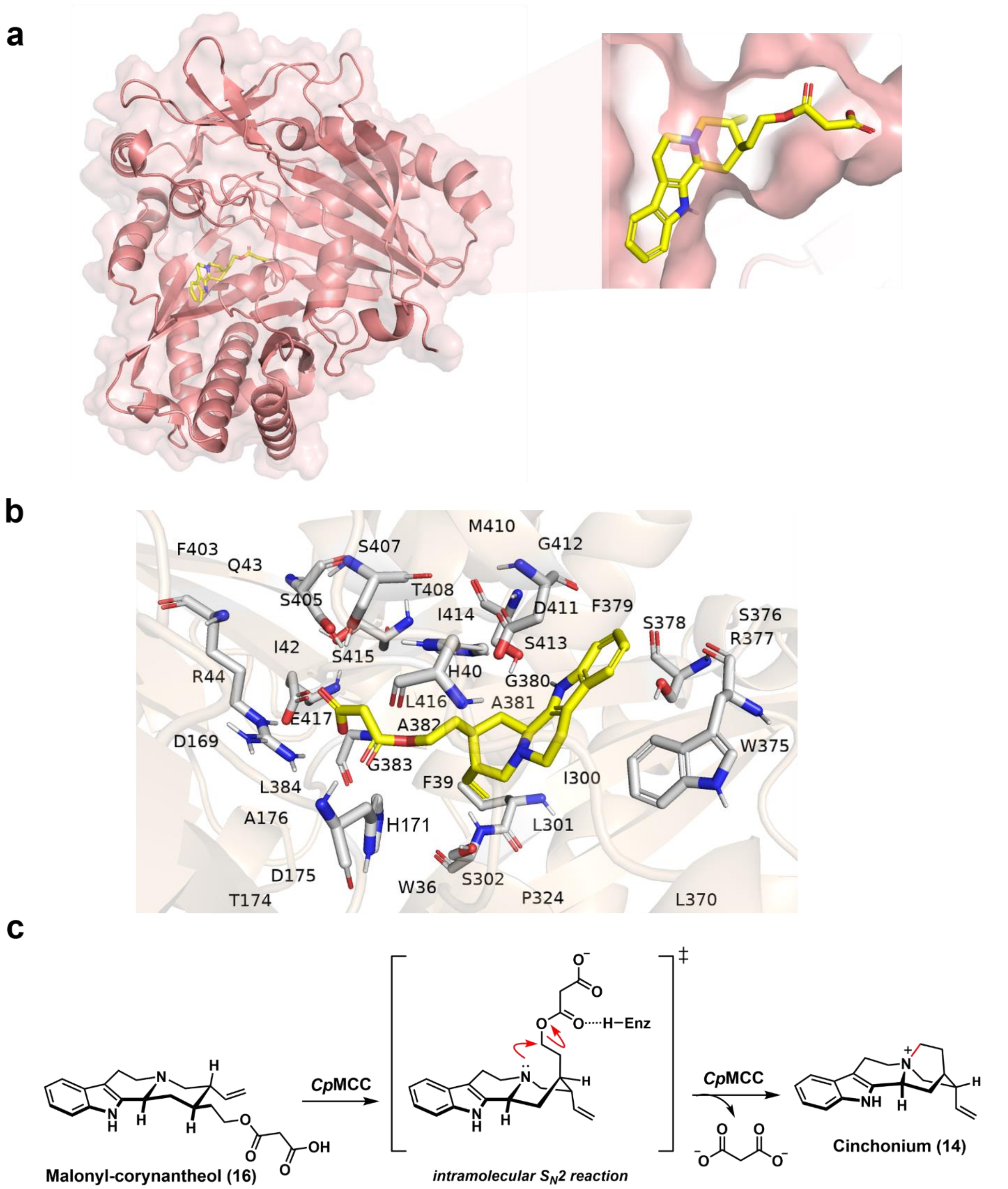

Supplementary Fig. 20. Docking model of *Cp*MCC with malonyl-corynantheol (16). a. AlphaFold3 model of *Cp*MCC in complex with malonyl-coryantheol (**16**, the scaffold with carbon atoms highlighted in yellow), showing the latter to be in a conformation where the bulky alkyl chain bearing malonyl group is in axial position. Docking was performed with AutoDock Vina,[75] implemented in SwissDock,[76] and visualized with PyMoL. **b.** *Cp*MCC residues within 5 Å to the ligand; putative key residues are highlighted. **c.** Proposed mechanism of *Cp*MCC.

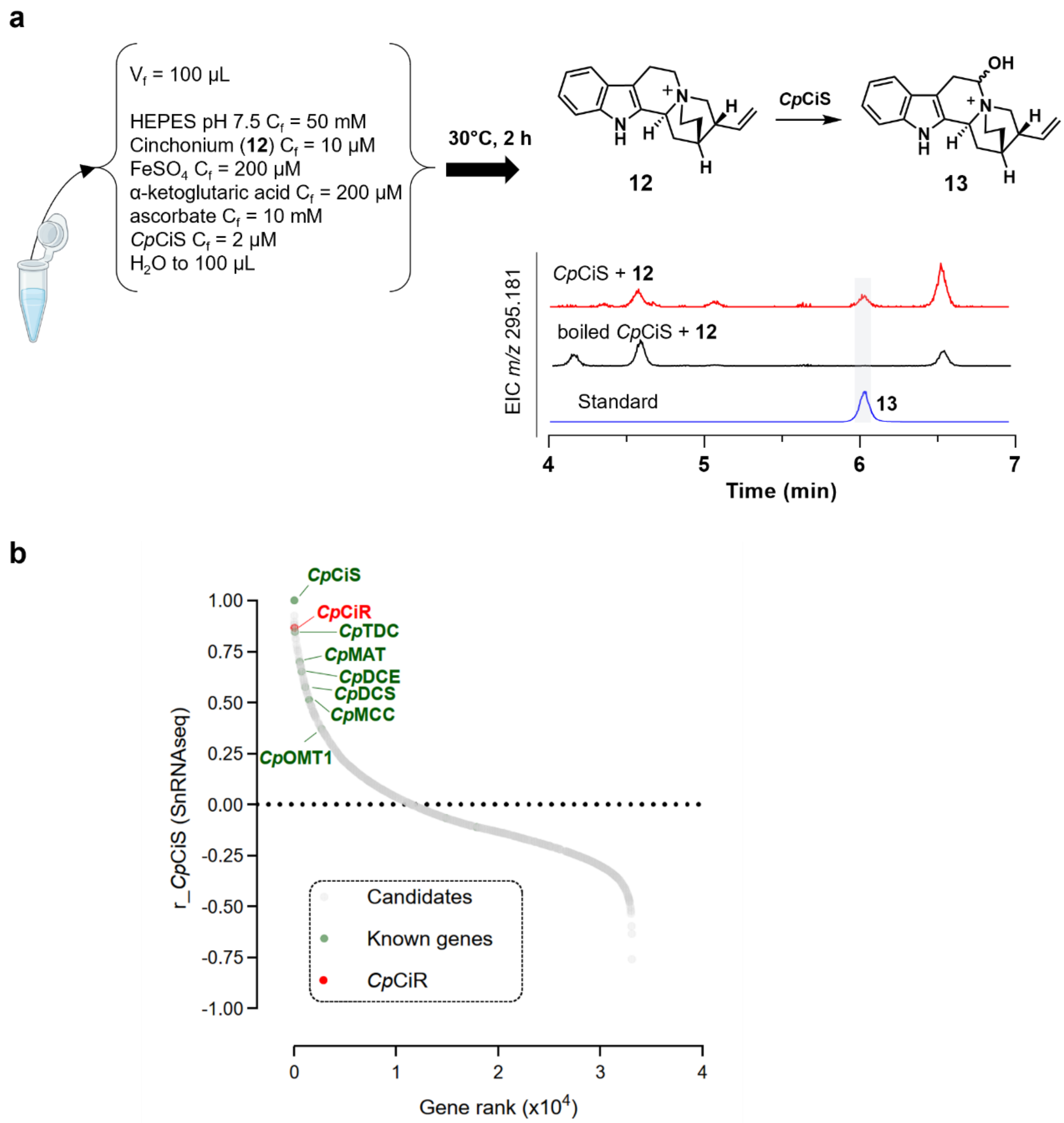

Supplementary Fig. 21. a. *In vitro* enzymatic assay of *Cp*CiS. Schematic of the *in vitro* assay conditions for *Cp*CiS using cinchonium (**12**) as the substrate and EICs of cinchonaminal (**13**) confirming the catalytic activity of *Cp*CiS *in vitro*. Assays were repeated independently three times with similar results. **b.** Ranking of reductase gene candidates based on co-expression with *Cp*CiS.

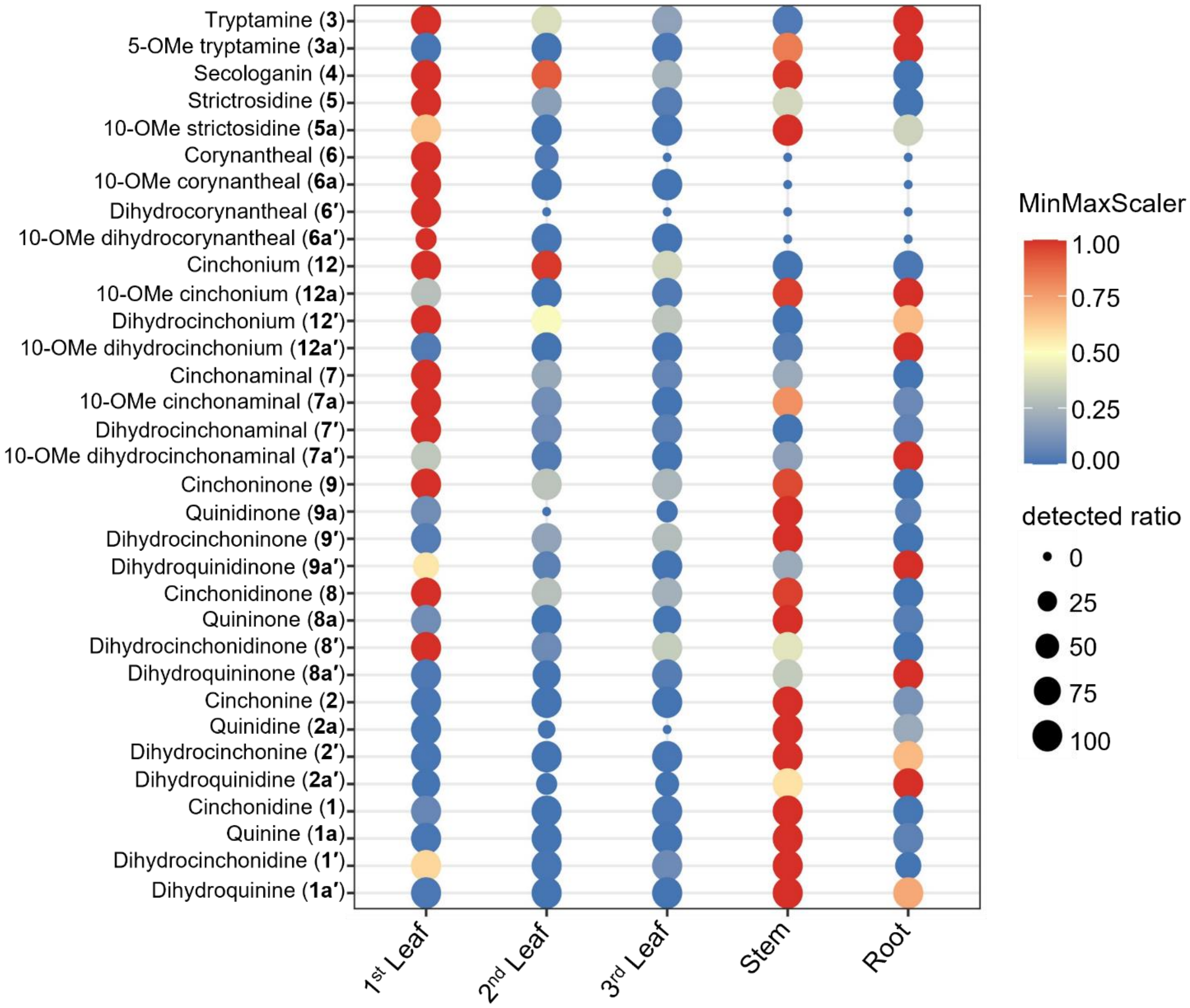

Supplementary Fig. 22. Metabolic profiling of *C. pubescens* tissues. Dot color indicates the relative abundance of each compound across tissues, and dot size represents the detection frequency among biological replicates ($n = 6$ per tissue). 1st, 2rd, and 3rd leaf refer to leaves at different developmental stage (1st leaf pair, 2nd leaf pair and 3rd leaf pair, from the apical bud).

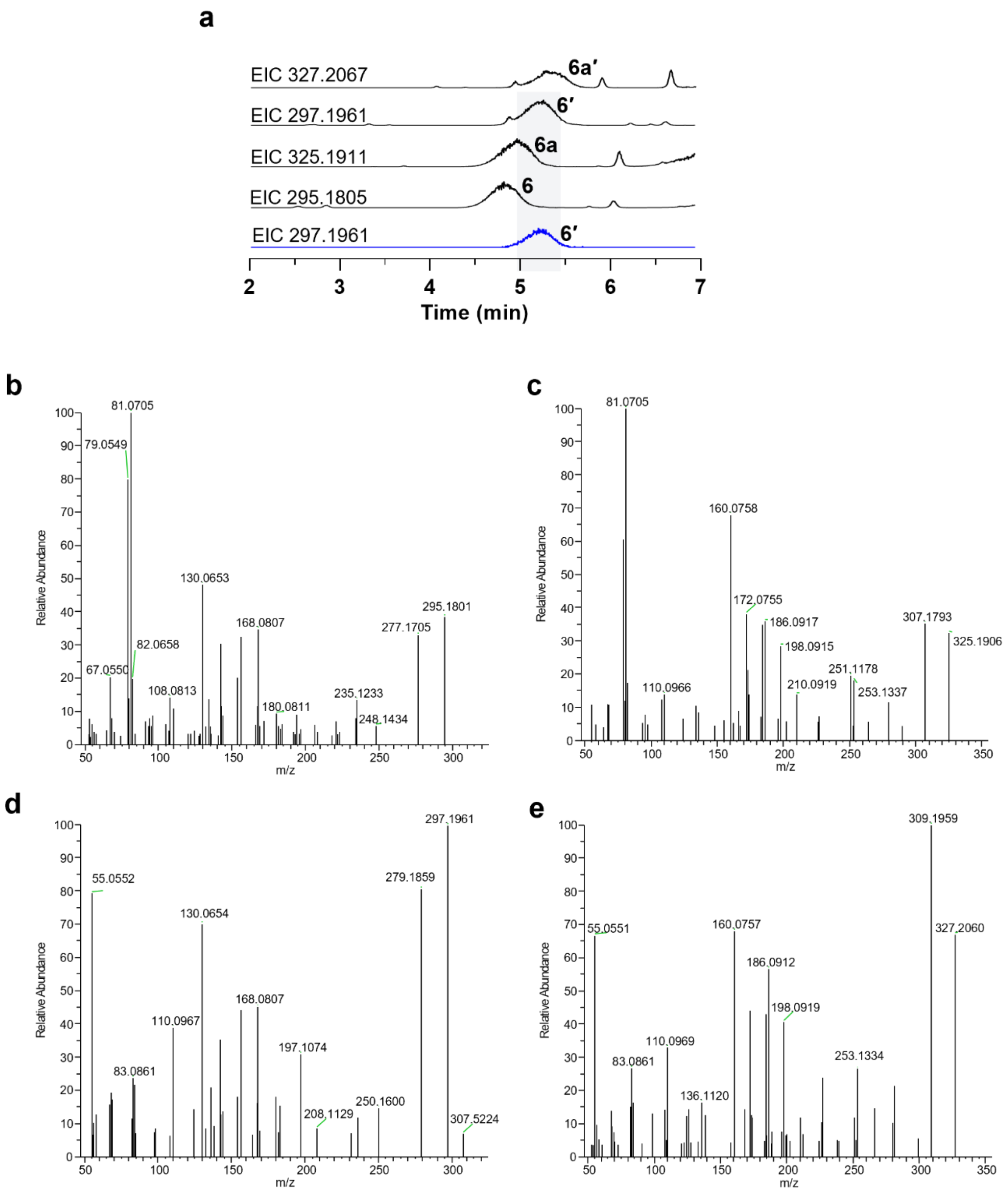

Supplementary Fig. 23. Identification of corynantheal and analogs in *C. pubescens*. a. EICs of corynantheal (**6**), 10-OMe corynantheal (**6a**), dihydrocorynantheal (**6′**) and 10-OMe dihydrocorynantheal (**6a′**) from plant extract (black). To distinguish among these structurally similar compounds, purified standard **8′** was used as reference (blue). The corresponding chromatographic peak is highlighted in blue. **b.** MS/MS fragmentation pattern of **6**. **c.** MS/MS fragmentation pattern of **6a**. **d.** MS/MS fragmentation pattern of **6′**. **e.** MS/MS fragmentation pattern of **6a′**. Compounds were reproducibly detected in biological replicates across four independent plants.

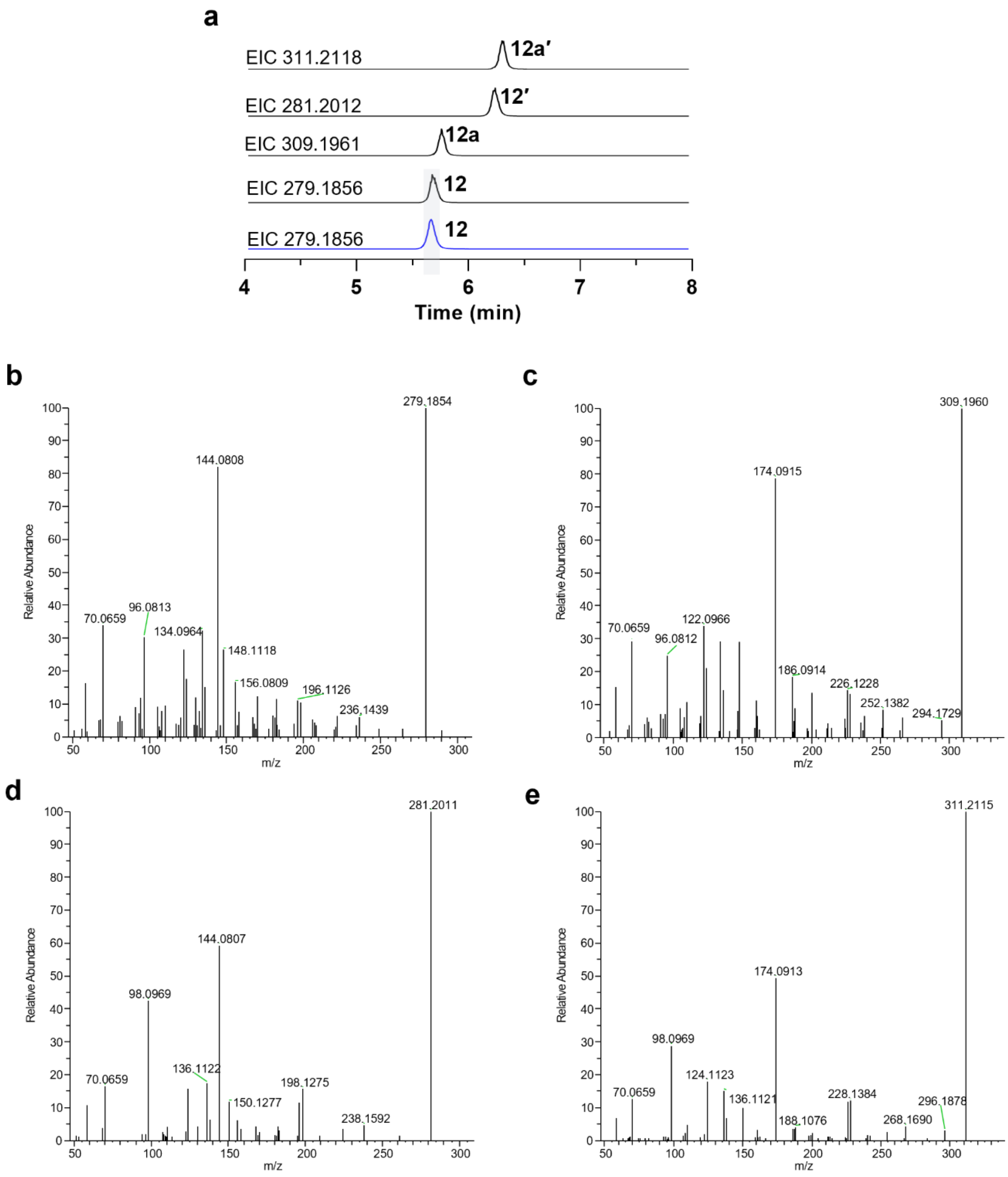

Supplementary Fig. 24. Identification of cinchonium and analogs in *C. pubescens*. a. EICs of cinchonium (**12**), 10-OMe cinchonium (**12a**), dihydrocinchonium (**12′**) and 10-OMe dihydrocinchonium (**12a′**) from plant extract (black). To distinguish among these structurally similar compounds, purified standard **12** was used as reference (blue). The corresponding chromatographic peak is highlighted in blue. **b.** MS/MS fragmentation pattern of **12**. **c.** MS/MS fragmentation pattern of **12a**. **d.** MS/MS fragmentation pattern of **12′**. **e.** MS/MS fragmentation pattern of **12a′**. Compounds were reproducibly detected in biological replicates across four independent plants.

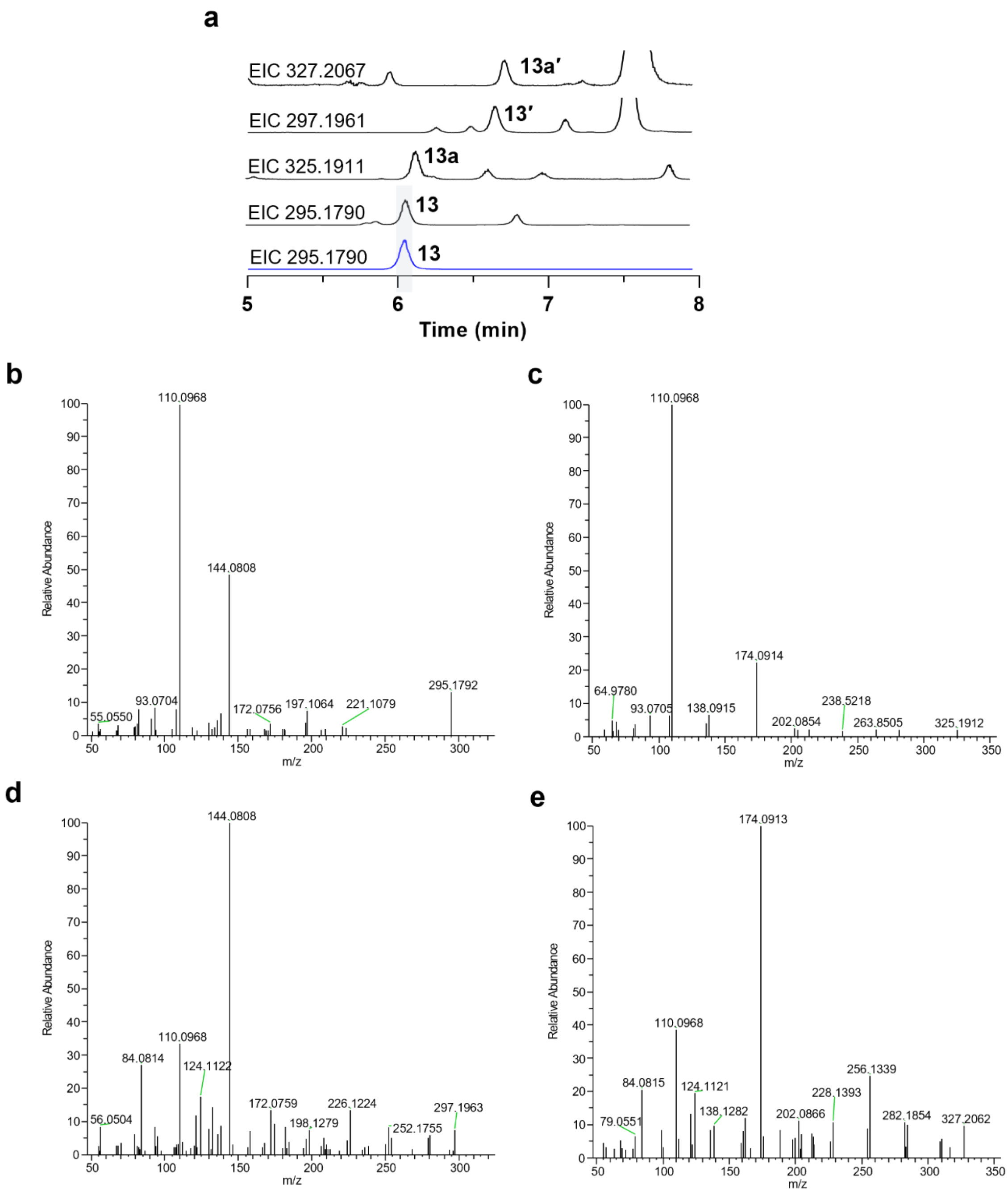

Supplementary Fig. 25. Identification of cinchonaminal and analogs in *C. pubescens*. a. EICs of cyclocinchonaminal (**13**), 10-OMe cyclocinchonaminal (**13a**), dihydrocinchonaminal (**13′**) and 10-OMe dihydrocinchonaminal (**13a′**) from plant extract (black). To distinguish among these structurally similar compounds, purified standard **13** was used as reference (blue). **b.** MS/MS fragmentation pattern of **13**. **c.** MS/MS fragmentation pattern of **13a**. **d.** MS/MS fragmentation pattern of **13′**. **e.** MS/MS fragmentation pattern of **13a′**. Compounds were reproducibly detected in biological replicates across four independent plants.

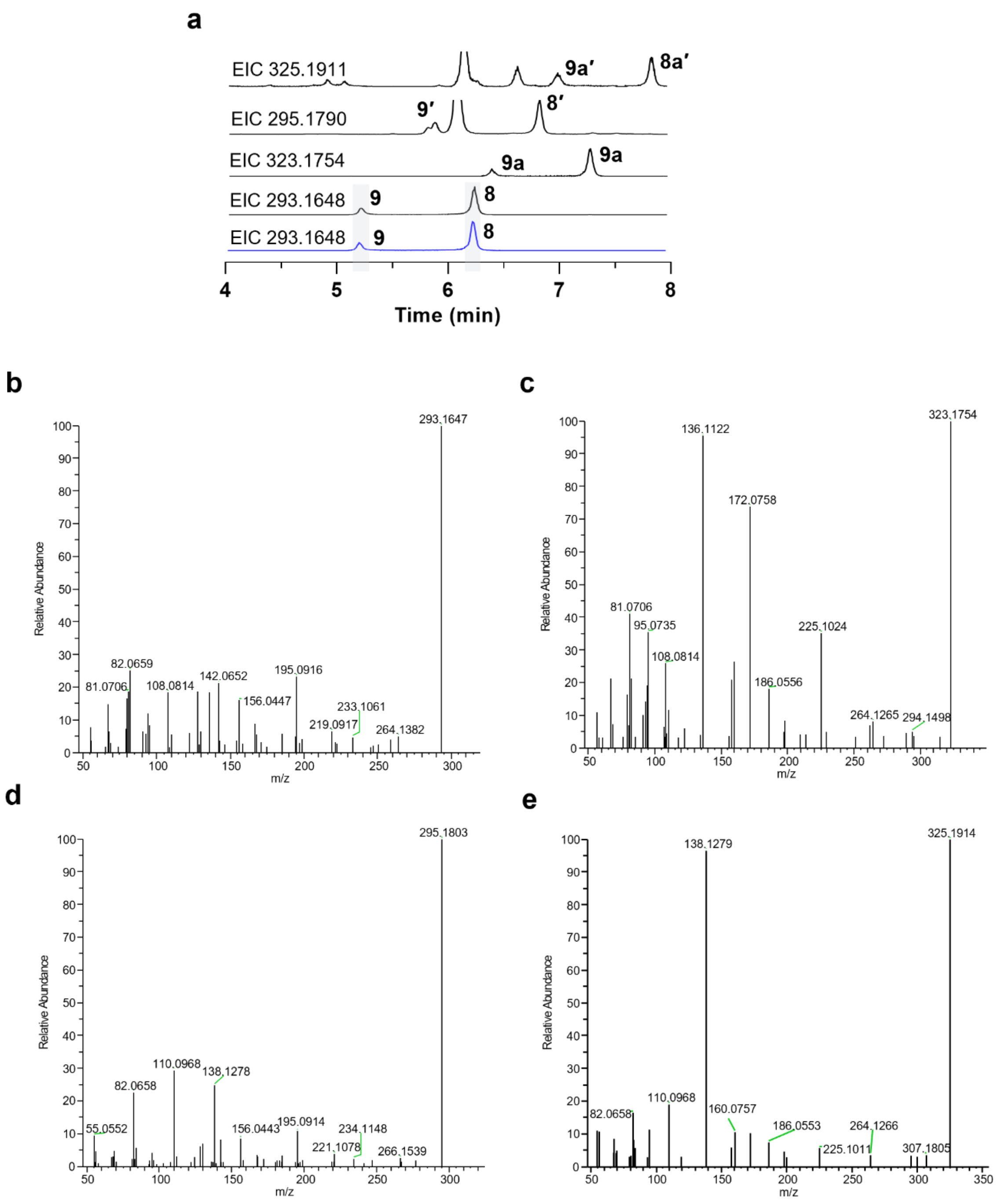

Supplementary Fig. 26. Identification of cinchoni(di)none and analogs in *C. pubescens*. a. EICs of cinchoni(di)none (**8**/**9**), quini(di)none (**8a**/**9a**), dihydro cinchoni(di)none (**8′**/**9′**) and dihydro quini(di)none (**8a′**/**9a′**) from plant extract (black). To distinguish among these structurally similar compounds, purified standards **8**/**9** were used as reference (blue). **b.** MS/MS fragmentation pattern of **8** and **9**. **c.** MS/MS fragmentation pattern of **8a** and **9a**. **d.** MS/MS fragmentation pattern of **8′** and **9′**. **e.** MS/MS fragmentation pattern of **8a′** and **9a′**. Compounds were reproducibly detected in biological replicates across four independent plants.

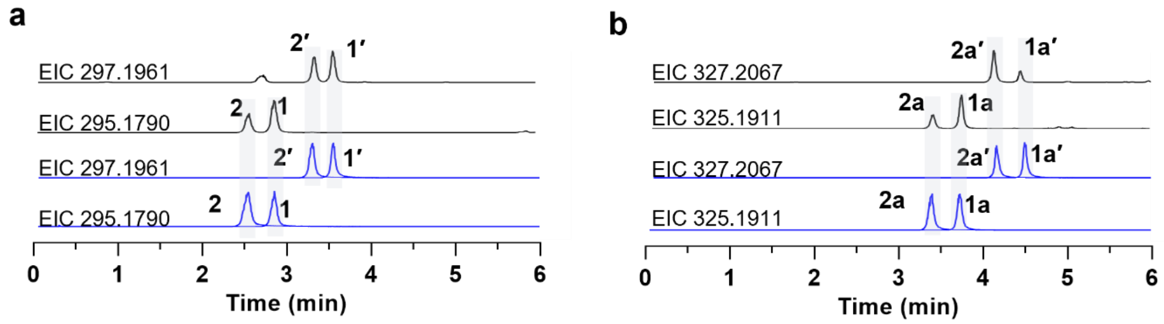

Supplementary Fig. 27. Identification of cinchoni(di)ne and analogs in *C. pubescens*. a. EICs of cinchoni(di)ne (**1**/**2**), dihydrocinchoni(di)ne (**1′**/**2′**) from plant extract (black), and their commercial standards (blue). **b.** EICs of quini(di)ne (**1a**/**2a**), dihydroquini(di)ne (**1a′**/**2a′**) from plant extract (black), and their commercial standards (blue). Compounds were reproducibly detected in biological replicates across four independent plants.

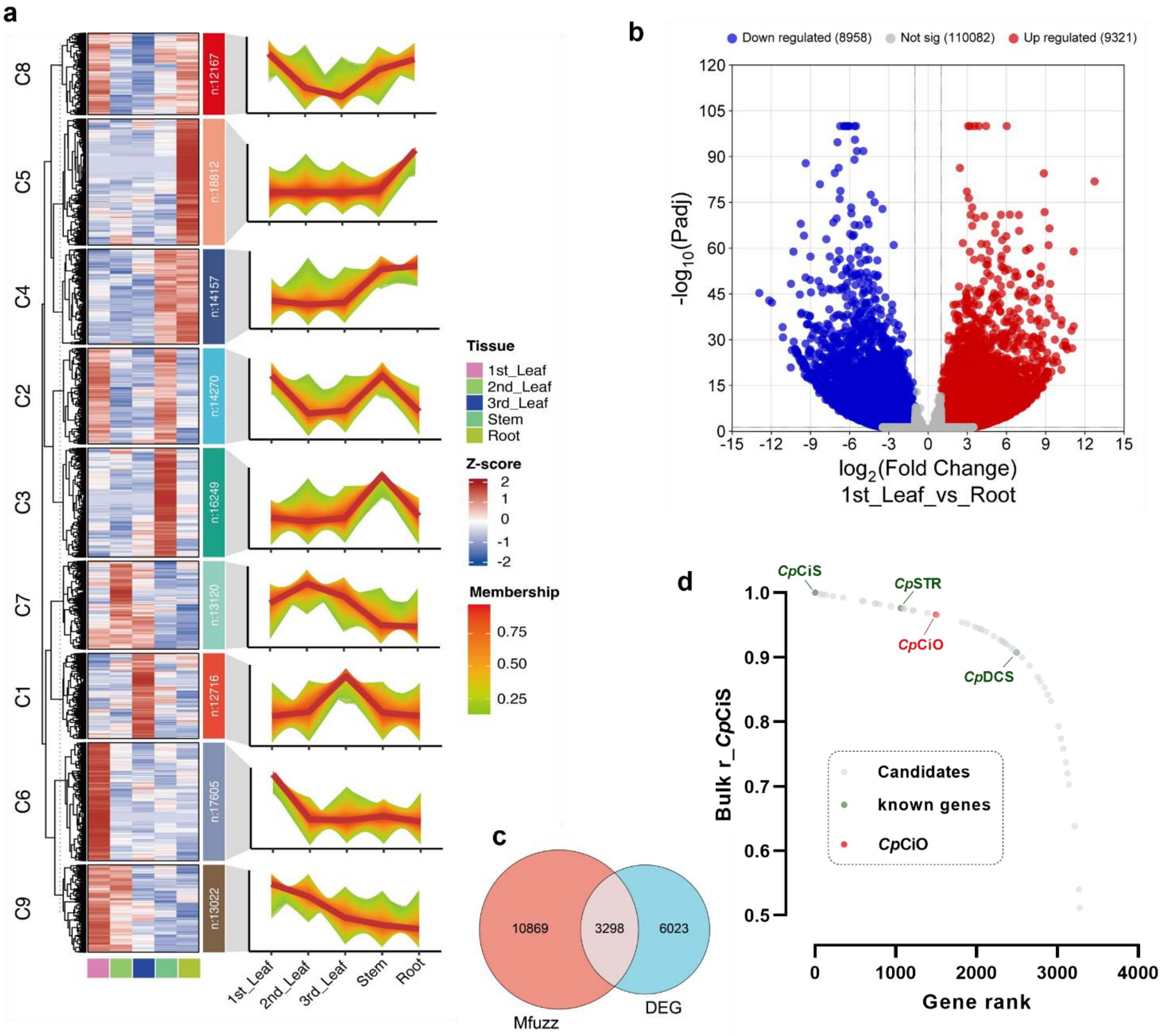

Supplementary Fig. 28. Bulk-tissue transcriptomic analysis for prioritizing gene candidates. a. Gene expression patterns of alkaloid biosynthetic candidates visualized using ClusterGVis. A complex heatmap combined with line plots represents the normalized expression (FPKM) of each gene across different *C. pubescens* tissues (1st leaf, 2nd leaf, 3rd leaf, stem, and root). Genes were clustered based on expression similarity to highlight potential pathway modules. **b.** Differentially expressed gene (DEG) analysis comparing root and 1st leaf samples using DESeq2. Fold change > 2 and P_{adj} < 0.05 were set as criteria. **c.** Integration of expression pattern clustering (**a**) and DEG data (**b**) to prioritize candidate genes involved in late-stage biosynthesis. **d.** Ranking of cytochrome P450 gene candidates based on Pearson correlation with *Cp*CiS using bulk tissue RNA-seq data.

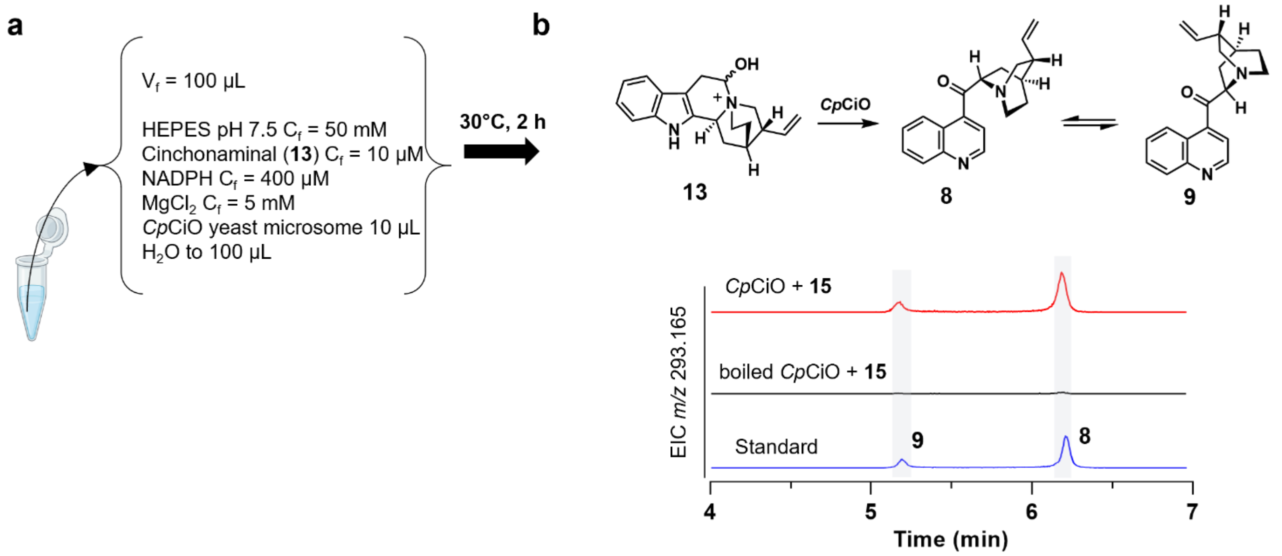

Supplementary Fig. 29. *In vitro* enzymatic assay of *Cp*CiO. a. Schematic of the *in vitro* assay used to test the activity of yeast microsomal preparations expressing *Cp*CiO using cinchonaminal **13** as the substrate. **b.** EICs of cinchoni(di)none **8**/**9** confirming the catalytic activity of *Cp*CiO *in vitro*. All assays were repeated independently three times with similar results.

a

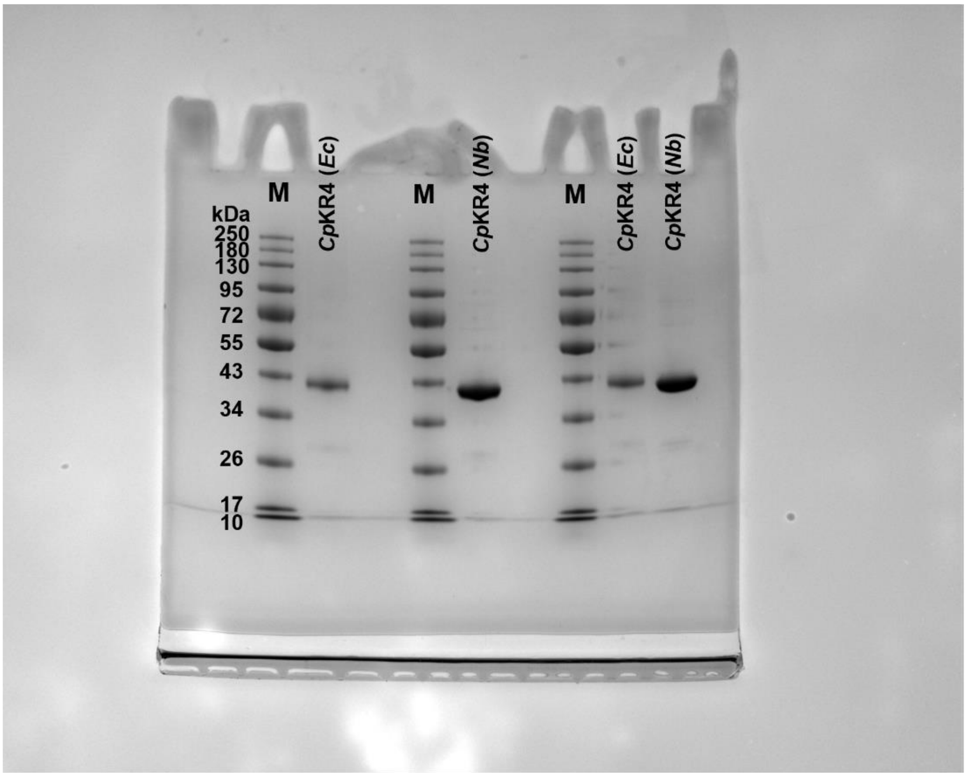

b

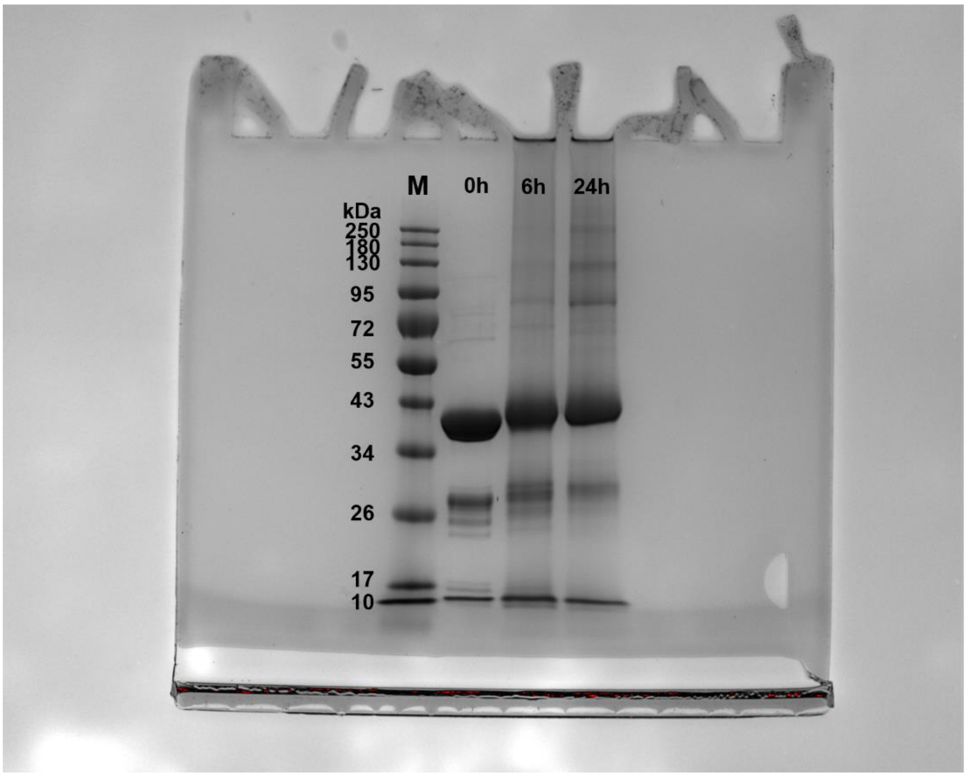

(continues on next page)

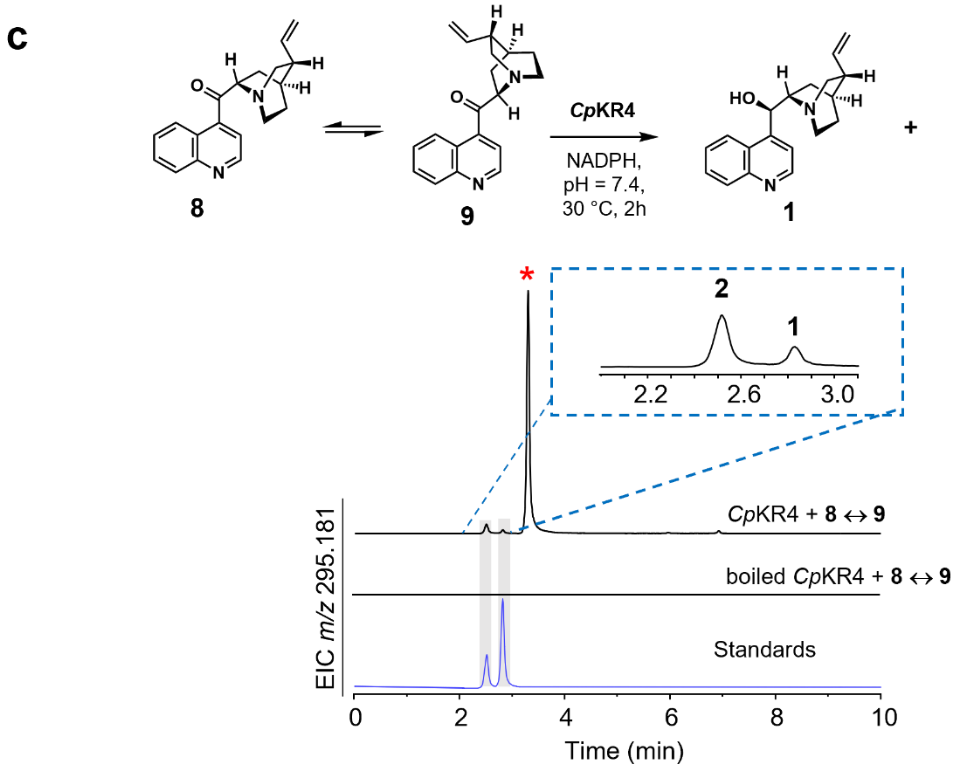

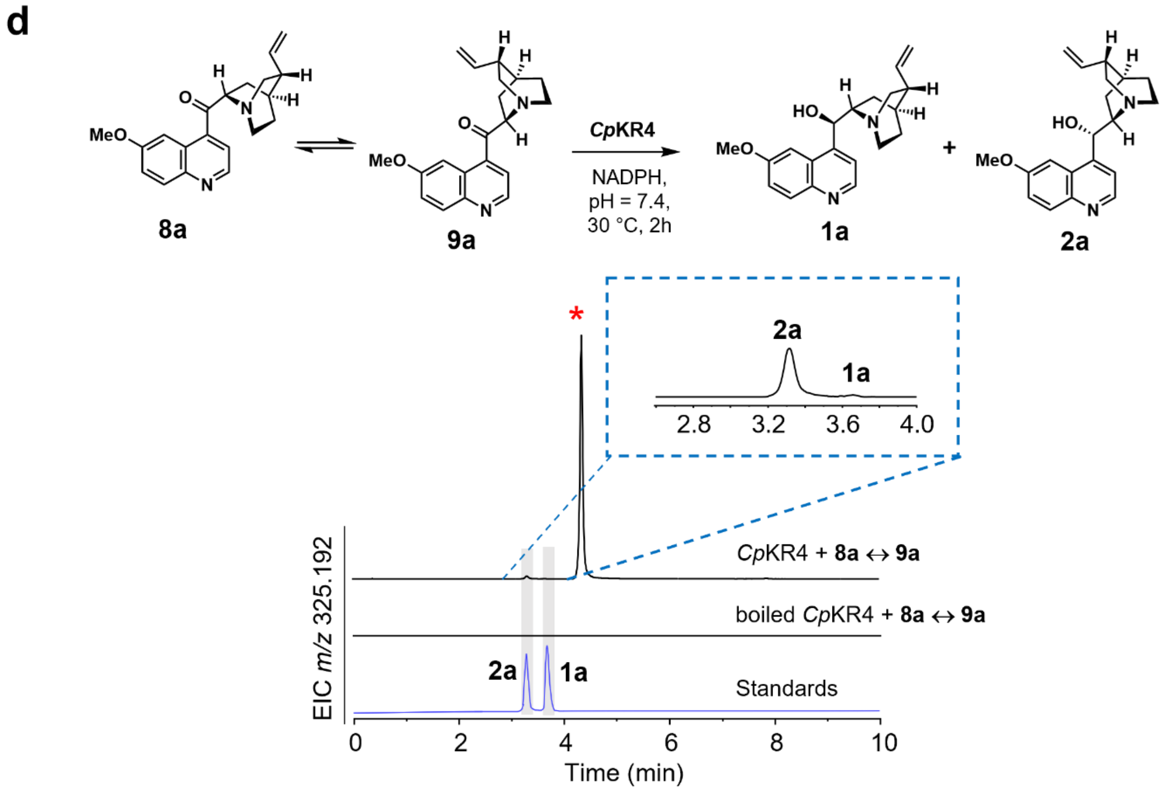

Supplementary Fig. 30. Additional data on the characterization of *Cp*KR4. a) SDS-PAGE analysis confirming the expression of *Cp*KR4 in both *E. coli* and *N. benthamiana*. Lane M: molecular weight marker (Color Prestained Protein Standard, P7719S, NEB). Lanes *Cp*KR4 (*Ec*): purified recombinant *Cp*KR4 (38.90 kDa) protein from *E. coli*. Lanes KR4 (*Nb*): purified recombinant *Cp*KR4 (38.90 kDa) protein from *N. benthamiana*. **b)** SDS-PAGE analysis confirming the stability of *Cp*KR4 with *N. benthamiana* extract. Lane M: molecular weight marker (Color Prestained Protein Standard, P7719S, NEB). Lanes 2: purified recombinant

*Cp*KR4 (38.90 kDa) protein from *E. coli*. Lanes 3-4: purified recombinant *Cp*KR4 (38.90 kDa) protein from *E. coli* incubated with *N. benthamiana* extract for 6 or 24 hours. **c.** *In vitro* assay of *Cp*AKR4 with cinchonidinone (**8**) and cinchoninone (**9**) along with EICs showing the formation of the cinchonidine (**1**) and cinchonine (**2**), along with a predominant unknown stereoisomer (indicated by an asterisk) which is not naturally produced by *Cinchona*. **a.** *In vitro* assay of *Cp*AKR4 with quininone (**8a**) and quinidinone (**9a**) along with EICs showing the formation of the quinine (**1a**) and quinidine (**2a**), along with a predominant unknown stereoisomer (indicated by an asterisk) which is not naturally produced by *Cinchona*.

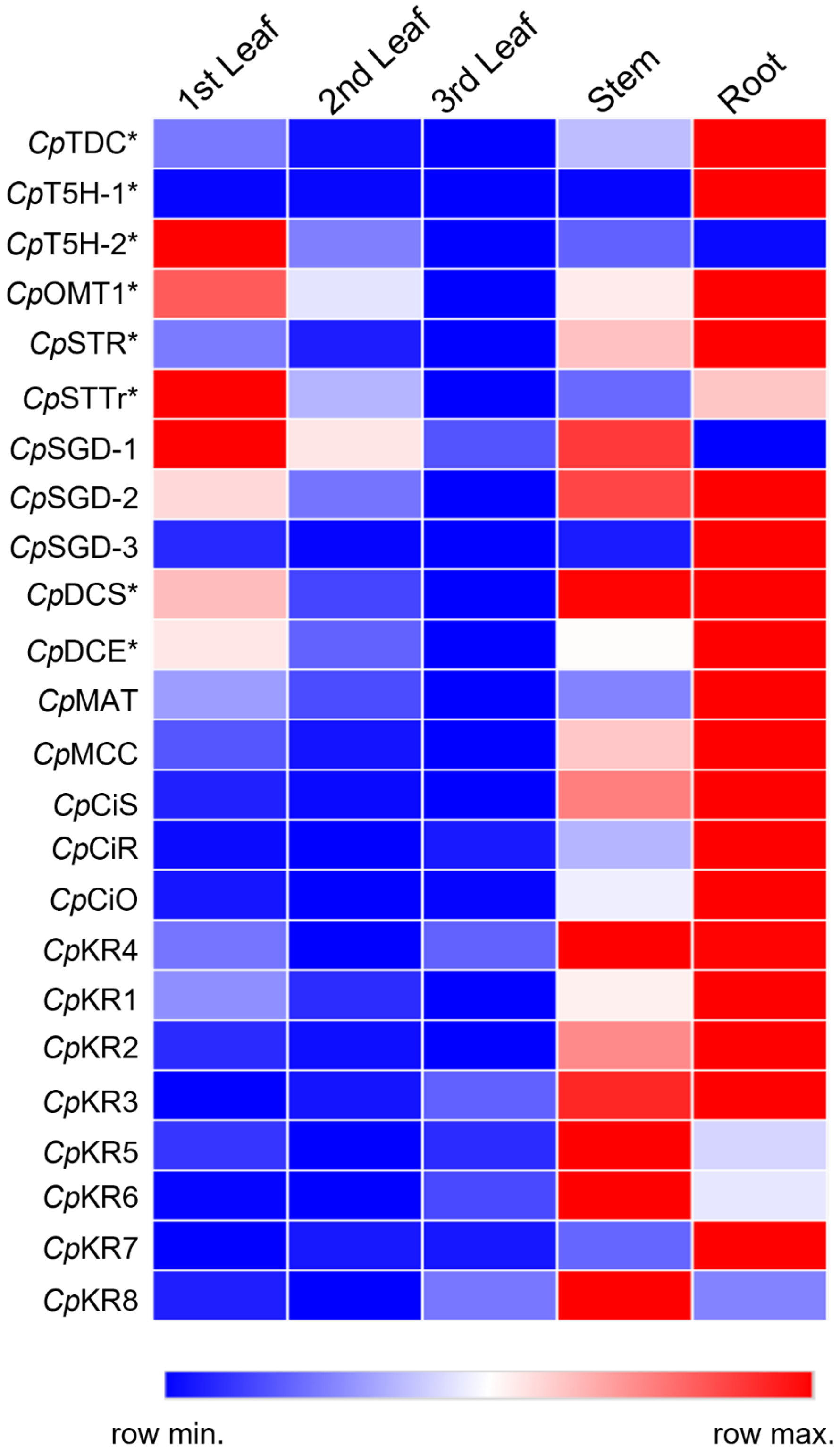

Supplementary Fig. 31. Expression profiles of herein reported Cinchona alkaloid genes (* indicates previously reported genes). Expression levels are represented as fragments per kilobase of transcript per million mapped reads (FPKM) values derived from *C. pubescens* transcriptomes. For each tissue, the plotted data represent an average of FPKM from three biological replicates. The heatmap was generated with Morpheus (https://software.broadinstitute.org/morpheus).

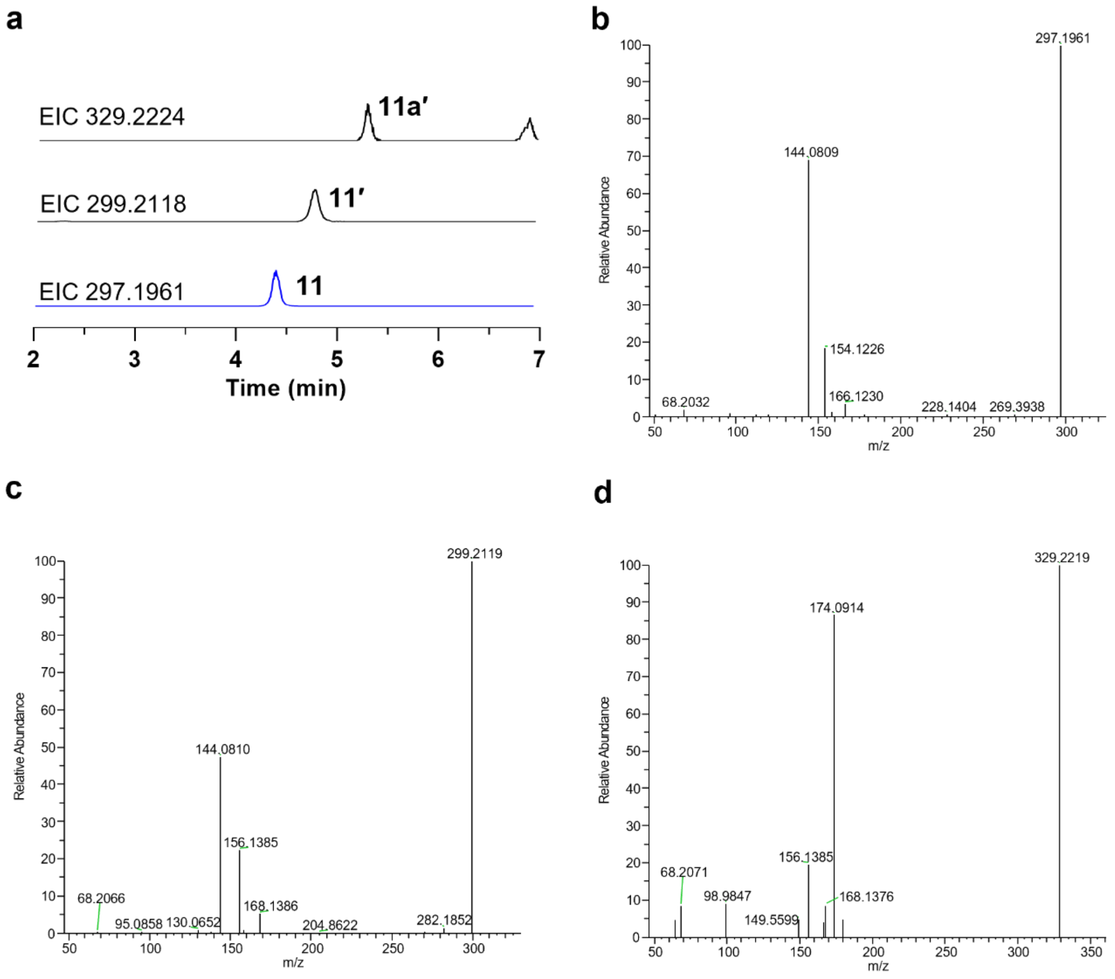

Supplementary Fig. 32. Identification of dihydro analogs of corynantheol in *N. benthamiana* after reconstructing the pathway. a. EICs of corynantheol (**11**, standard, highlighted in blue), dihydrocorynantheol (**11′**) and 10-OMe dihydrocorynantheol (**11a′**) from *N. benthamiana* extracts (black). **b.** MS/MS fragmentation pattern of standard **11**. **c.** MS/MS fragmentation pattern of **11′**. **d.** MS/MS fragmentation pattern of **11a′**. Compounds were reproducibly detected in biological replicates across four independent plants.

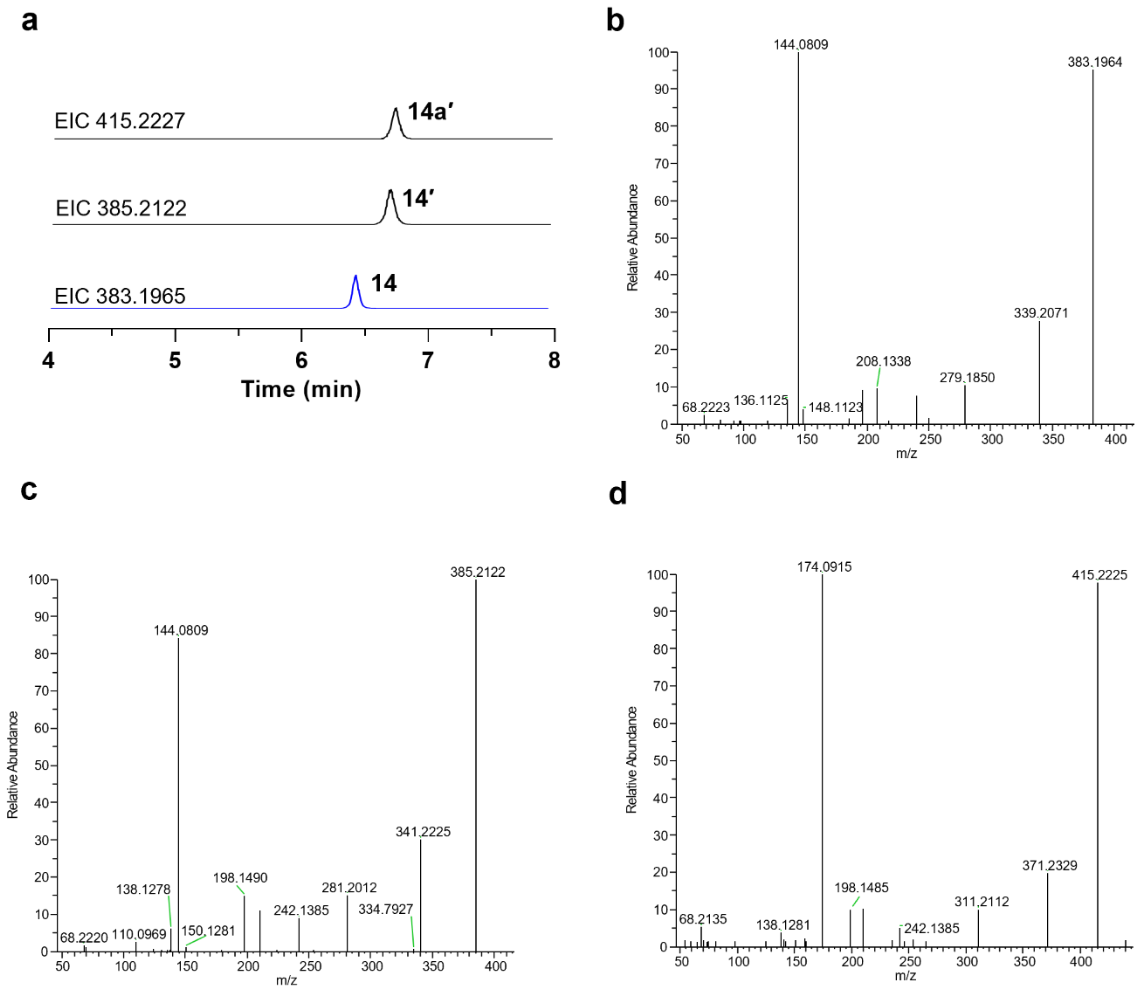

Supplementary Fig. 33. Identification of dihydro analogs of malonyl-corynantheol in *N. benthamiana* after reconstructing the pathway. a. EICs of malonyl-corynantheol (**14**, standard, highlighted in blue), malonyl-dihydrocorynantheol (**14′**) and 10-OMe malonyl-dihydrocorynantheol (**14a′**) from *N. benthamiana* extracts (black). **b.** MS/MS fragmentation pattern of standard **14**. **c.** MS/MS fragmentation pattern of **14′**. **d.** MS/MS fragmentation pattern of **14a′**. Compounds were reproducibly detected in biological replicates across four independent plants.

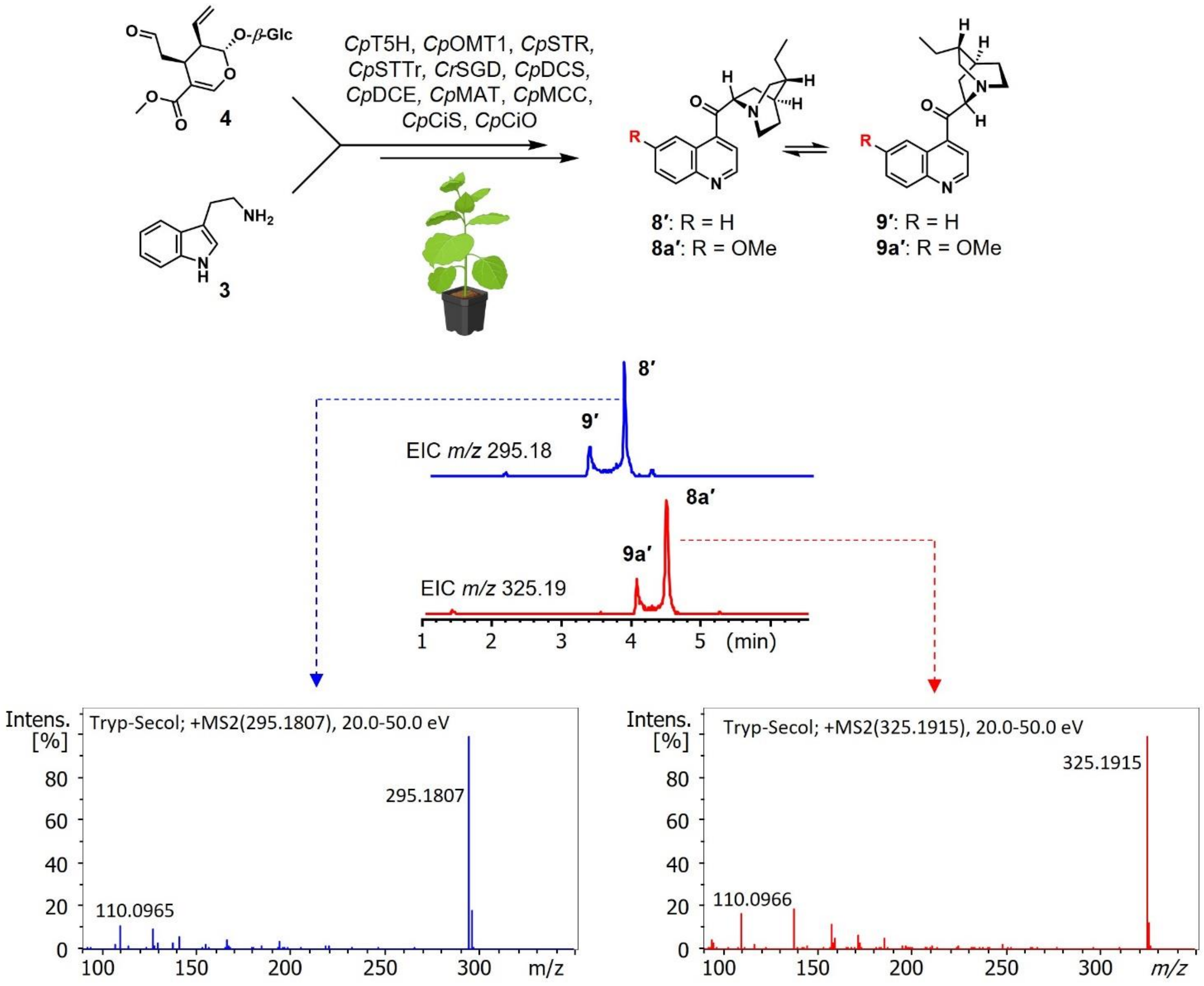

Supplementary Fig. 34. Biosynthesis of *Cinchona* quinoline alkaloid scaffolds from secologanin (4) and tryptamine (3). EICs and MS/MS spectra evidencing the concomitant formation of both non-methoxylated and methoxylated keto quinoline alkaloids **8′**, **9′**, **8a′** and **9a′** following transient expression of indicated genes in *N. benthamania* and subsequent infiltration of tryptamine and secologanin.

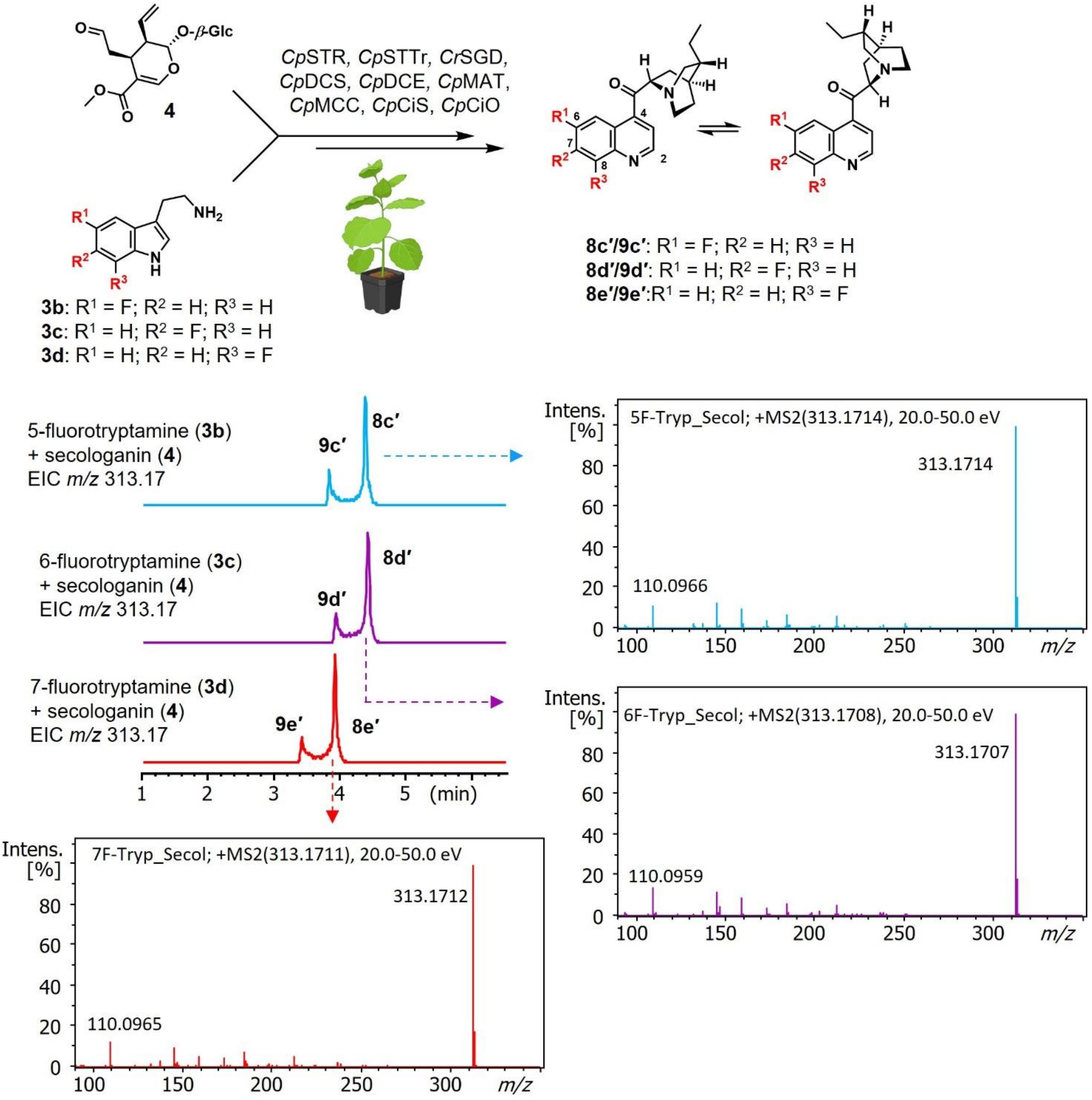

Supplementary Fig. 35. Directed biosynthesis of fluorinated quinoline compounds. EICs and MS/MS evidencing/displaying the formation of fluorinated analogs of *Cinchona* quinoline alkaloids in *N. benthamania* following transient expression of the indicated genes and subsequent co-infiltration of secologanin (**4**) with 5-, 6-, or 7-fluorotryptamine (**3b-d**).

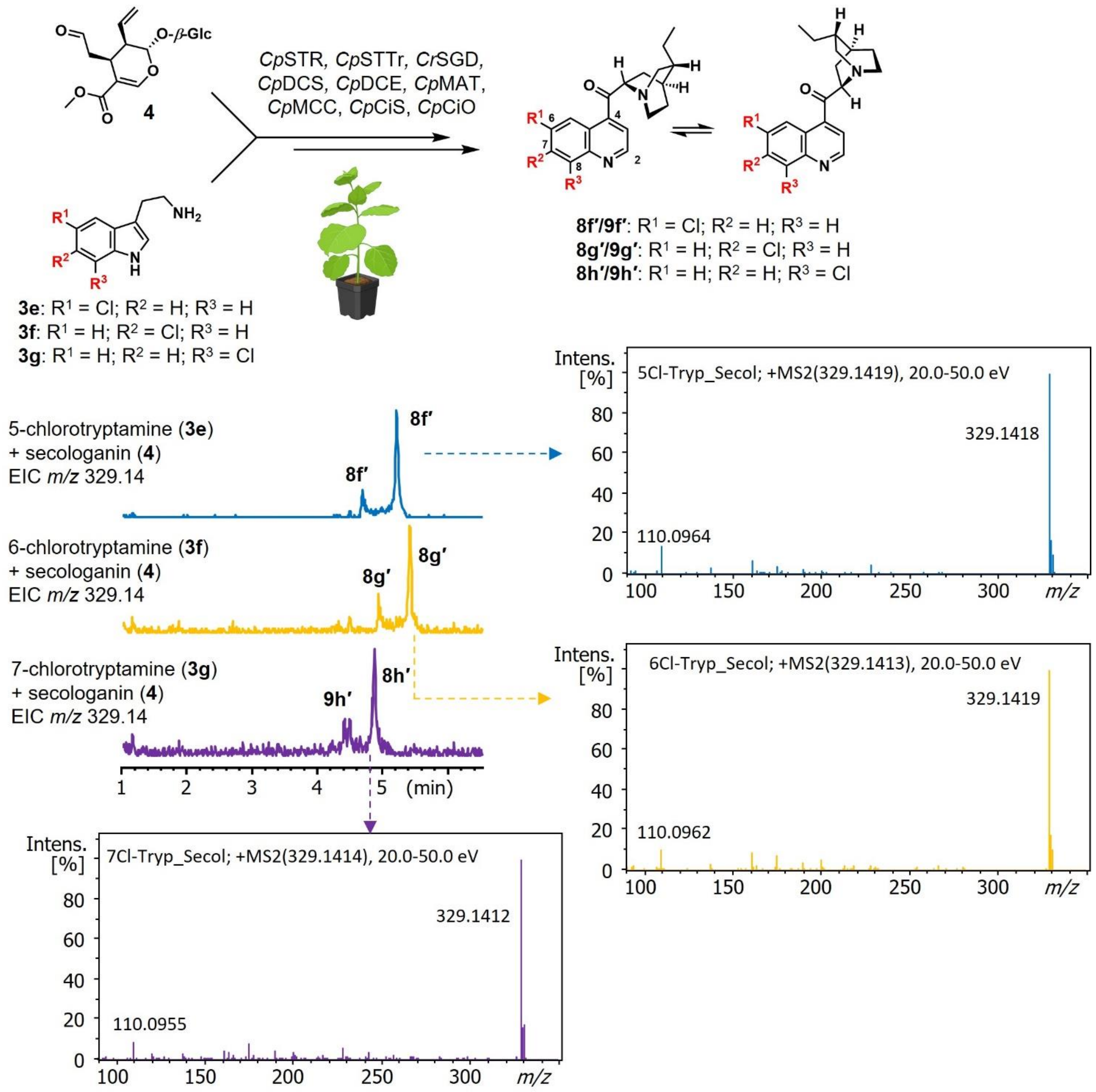

Supplementary Fig. 36. Directed biosynthesis of chlorinated quinoline compounds. EICs and MS/MS spectra displaying the formation of chlorinated analogs of *Cinchona* quinoline alkaloids in *N. benthamania* following transient expression of the indicated genes and subsequent co-infiltration of secologanin (**4**) with 5-, 6-, or 7-chlorotryptamine (**3e-g**).

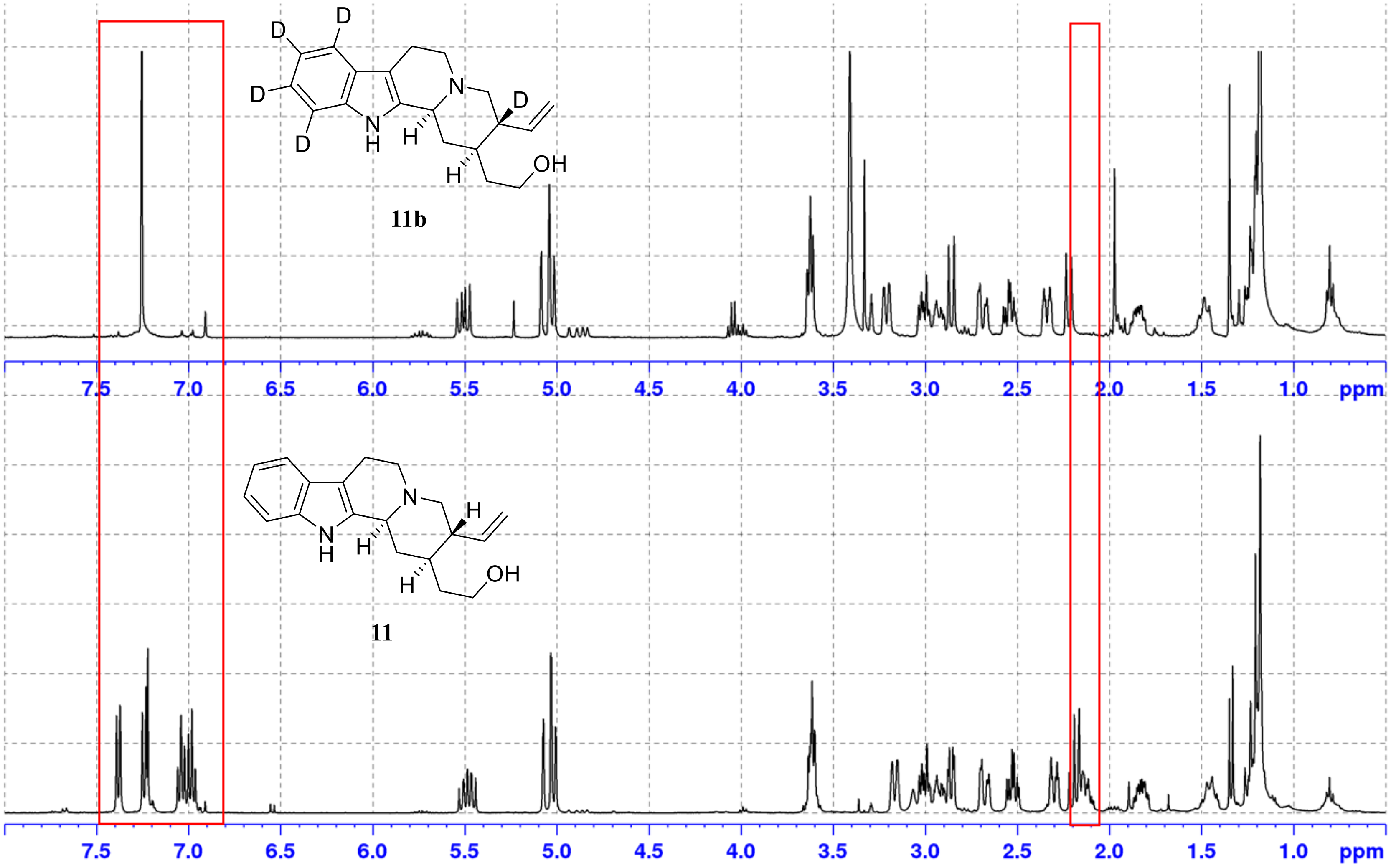

Supplementary Fig. 37. Comparison of ^{1}H NMR spectrum (400 MHz) of corynantheol (11, bottom) and the labeled analog *d*$_5$-corynantheol (11b, top) in chloroform-*d*$_3$: methanol-*d*$_4$ (95:5). Red highlighted regions evidence the isotopic labeling in **11b**.

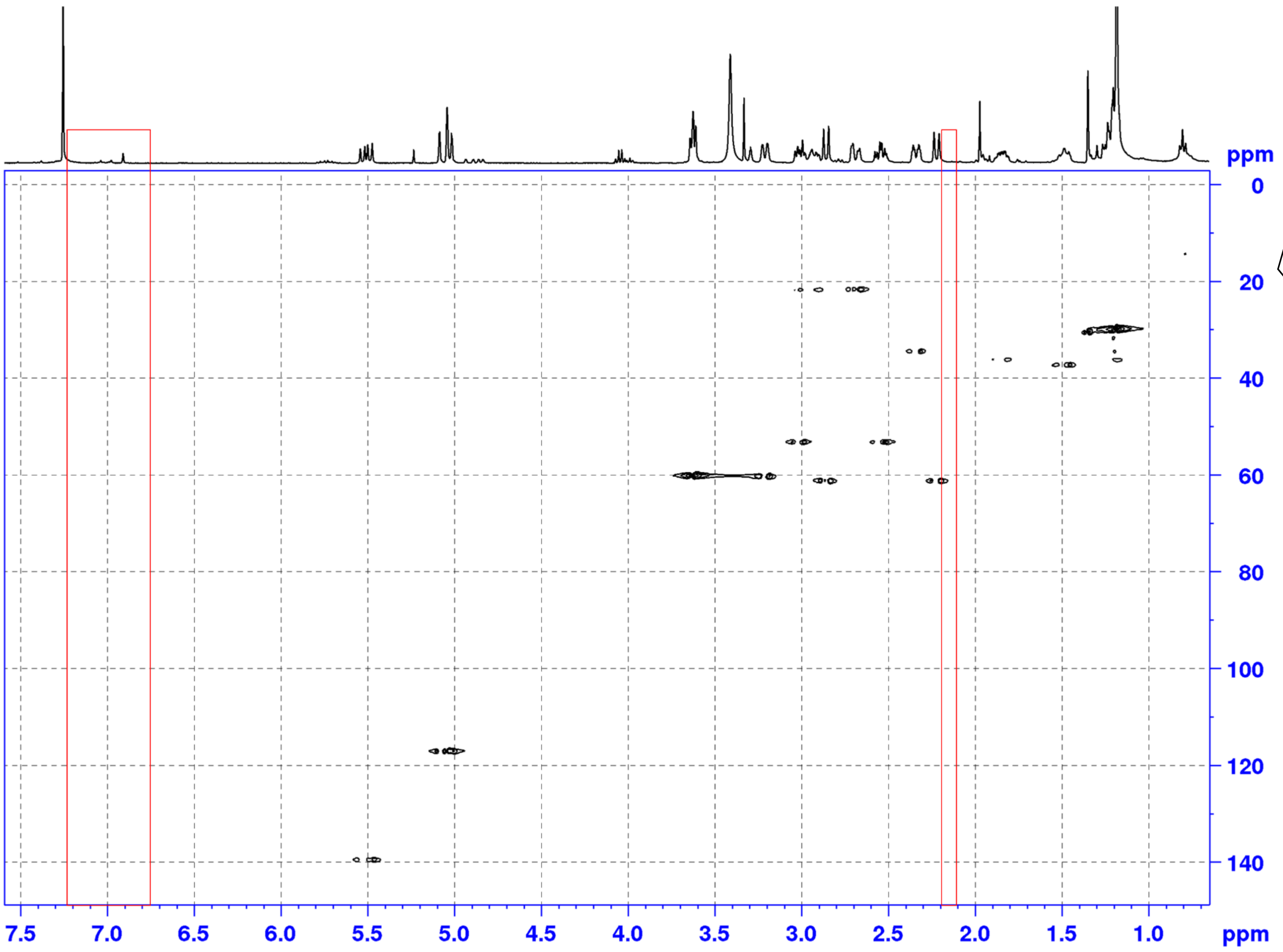

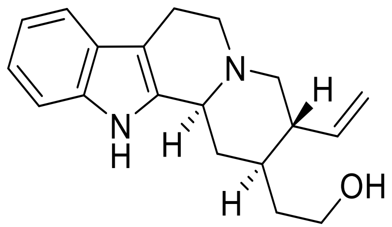

11b

Supplementary Fig. 38. ^{1}H-^{13}C HSQC NMR spectrum of *d*5-corynantheol (11b) in chloroform-*d*3: methanol-*d*4 (95:5). Red highlighted regions evidence the isotopic labeling in **11b**.

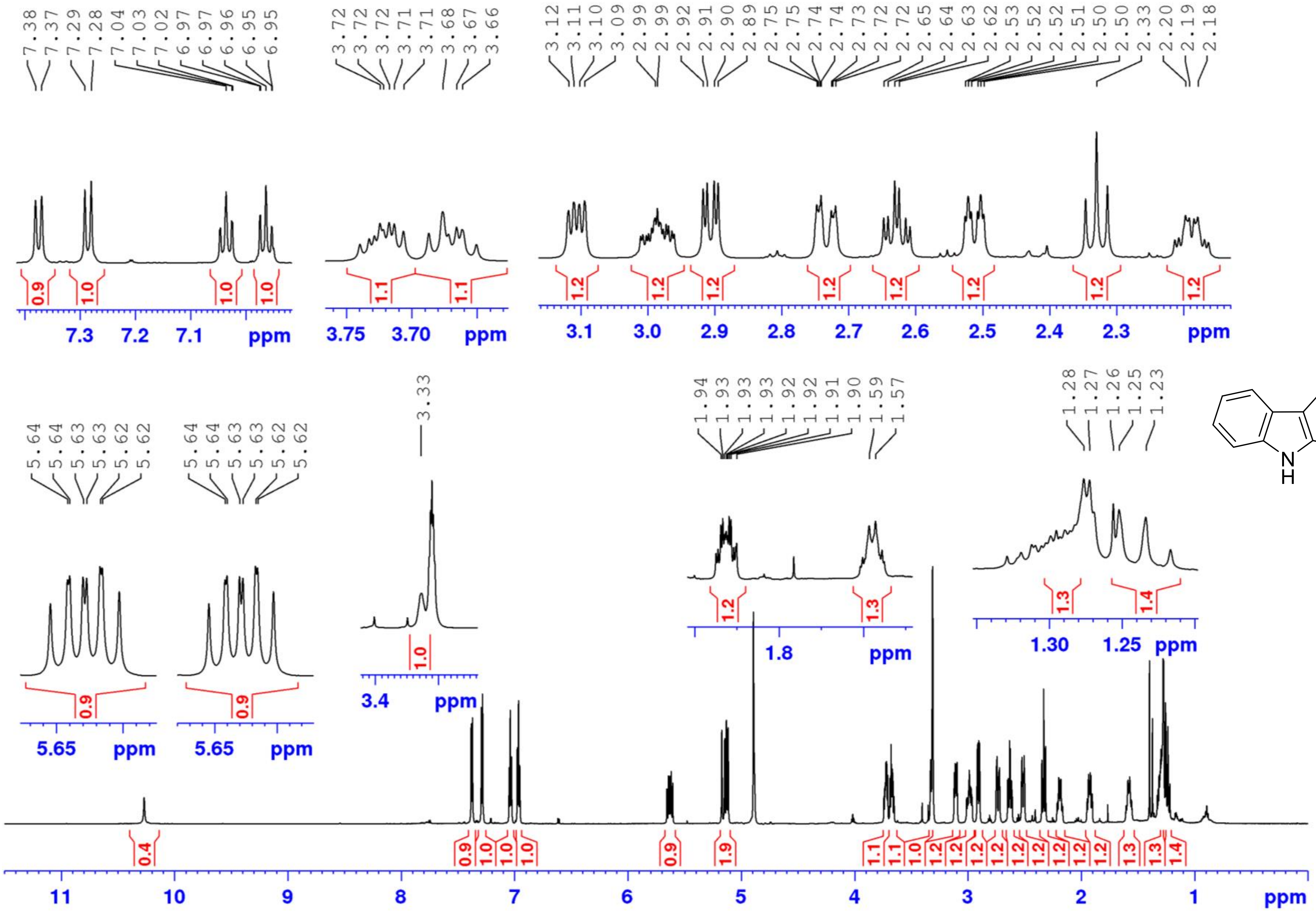

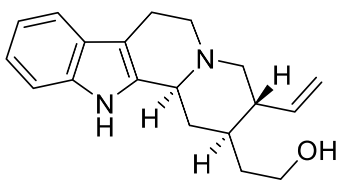

11

Supplementary Fig. 39. **^{1}H NMR spectrum (700 MHz) of corynantheol (11) in methanol-*d*$_3$.**

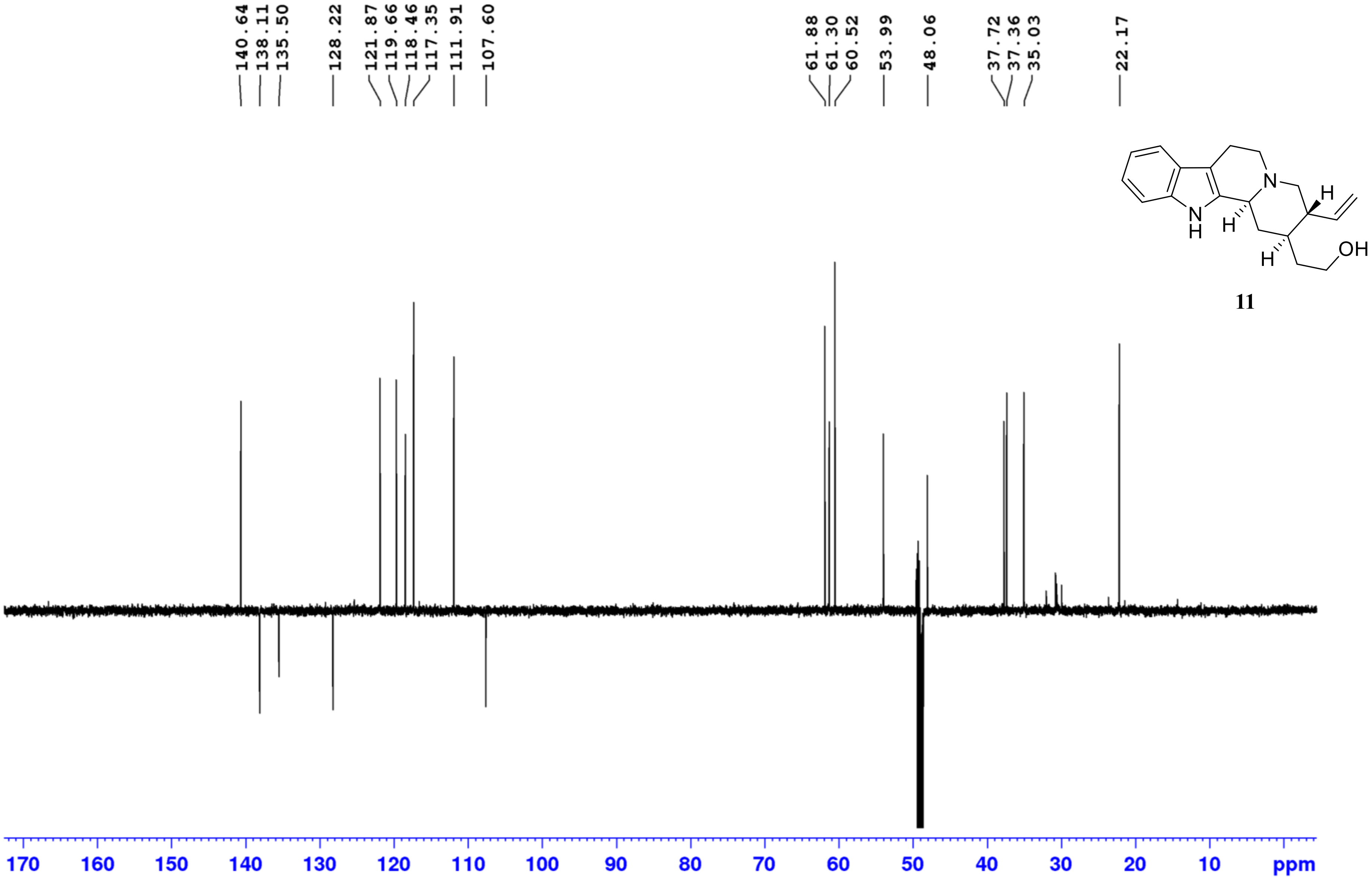

11

Supplementary Fig. 40. DEPTQ NMR spectrum of corynantheol (11) in methanol-*d*3.

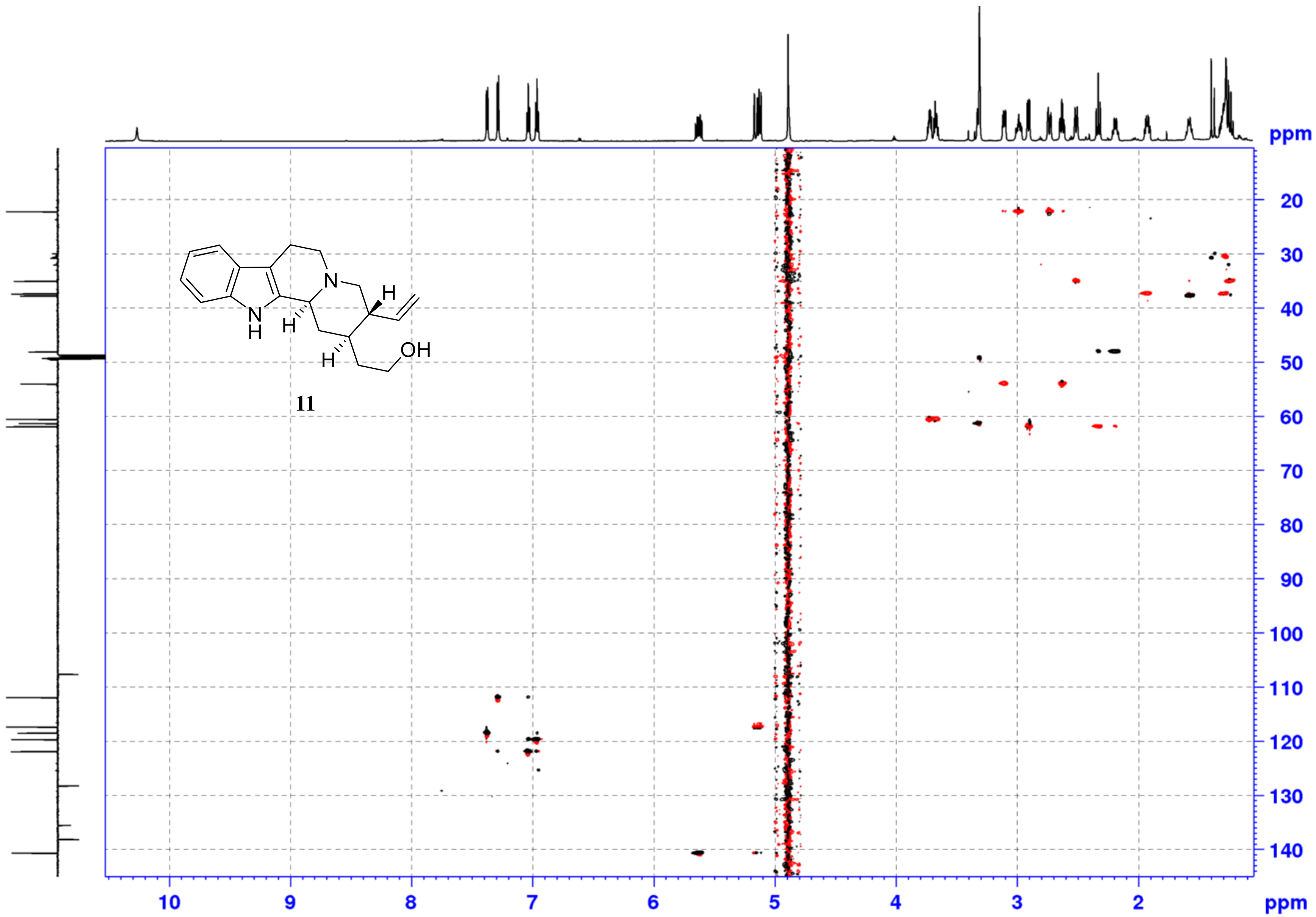

Supplementary Fig. 41. Phase-sensitive ^{1}H-^{13}C HSQC NMR spectrum of corynantheol (11) in methanol-d_3.

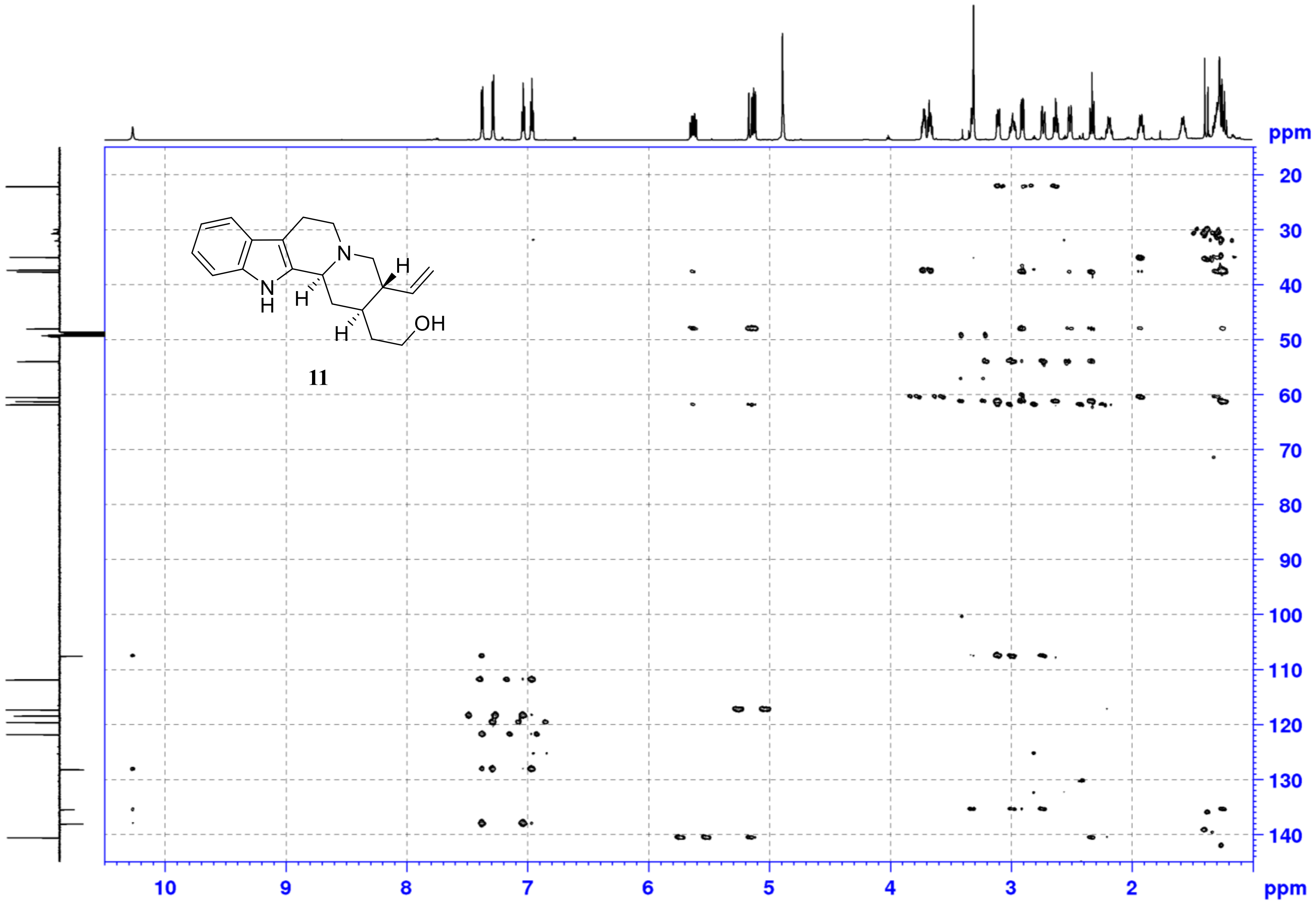

Supplementary Fig. 42. HMBC NMR spectrum of corynantheol (11) in methanol-d_3.

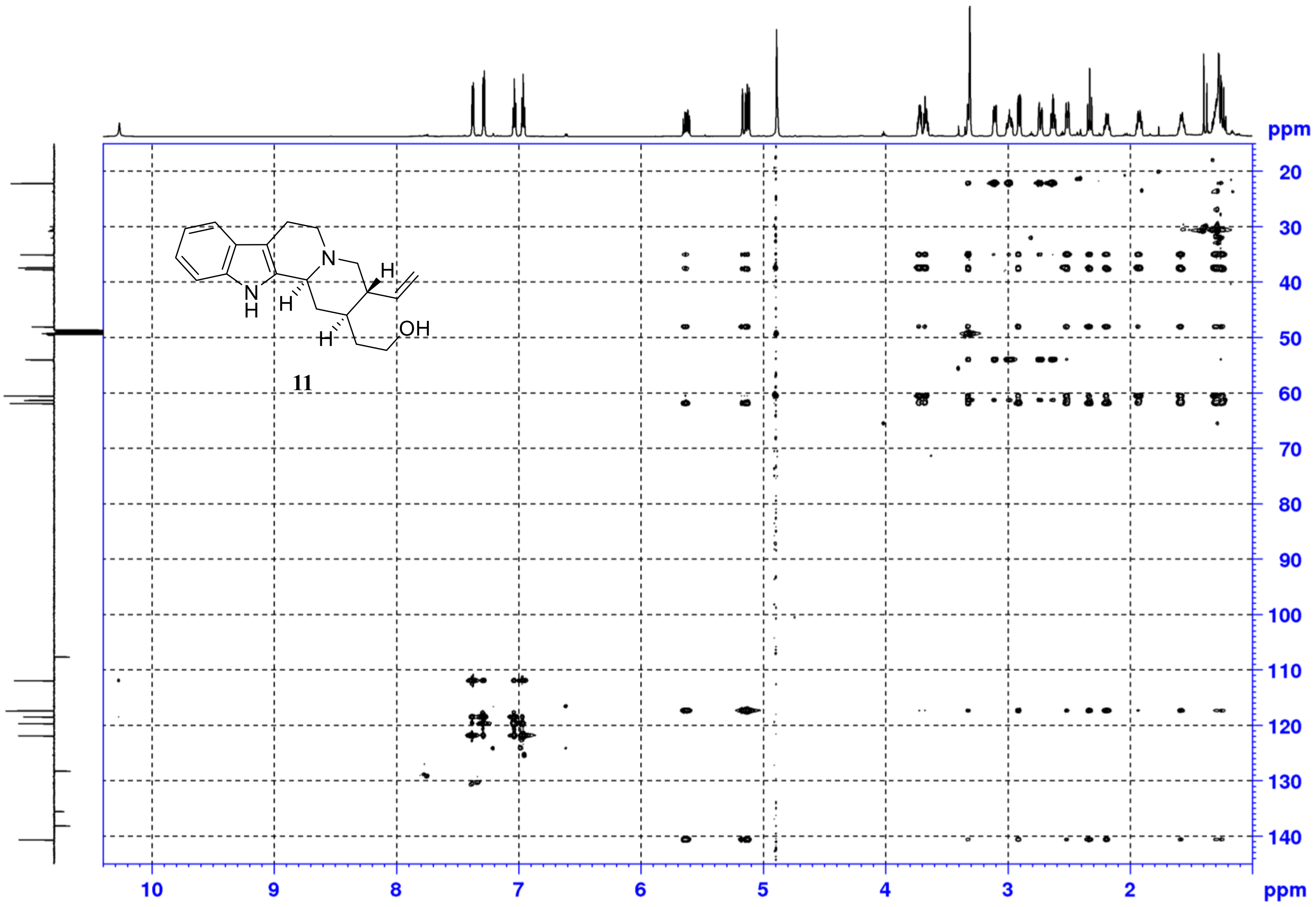

Supplementary Fig. 43. HSQC-TOCSY NMR spectrum of corynantheol (11) in methanol-*d*₃.

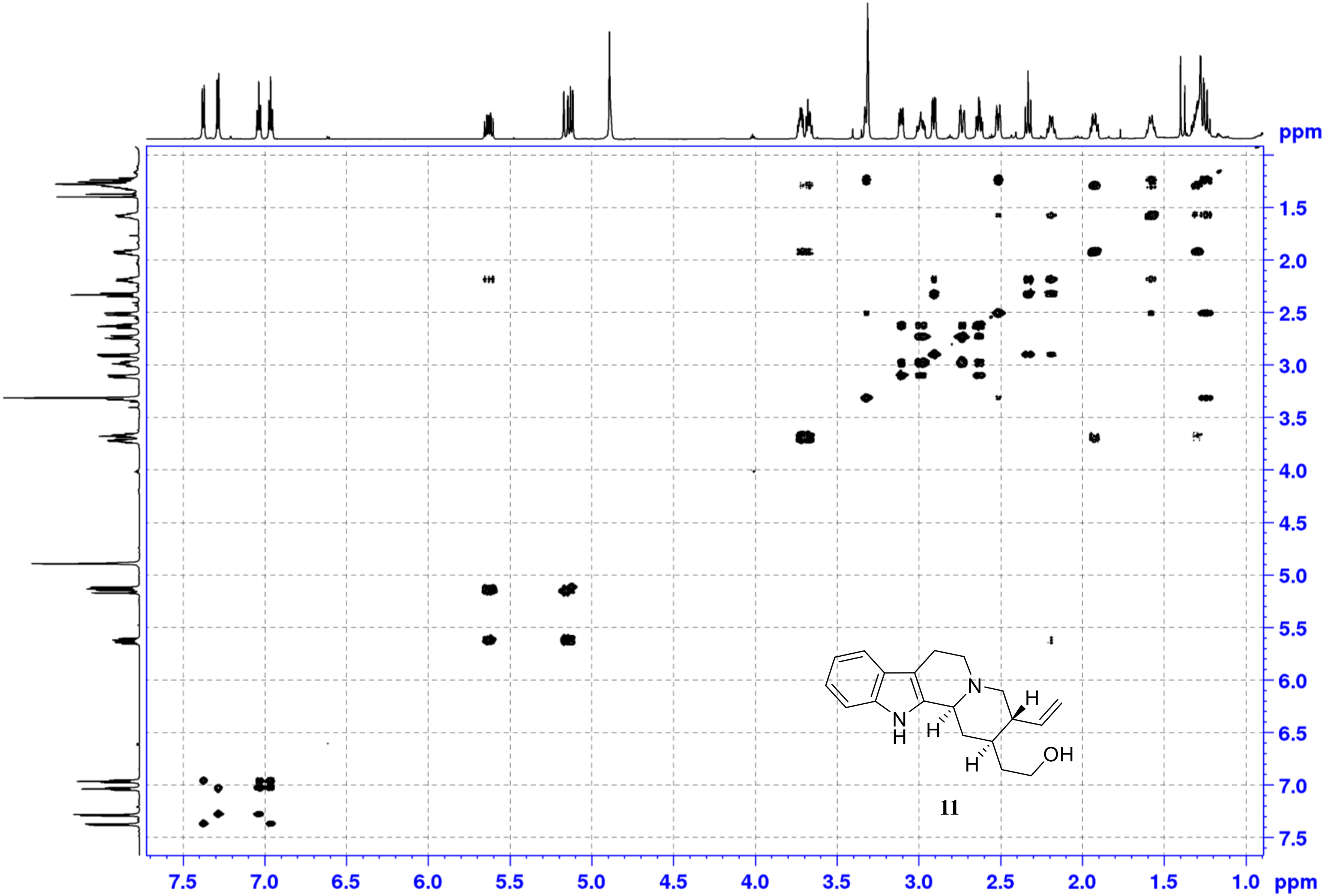

Supplementary Fig. 44. DQFCOSY NMR spectrum of corynantheol (11) in methanol-d_3.

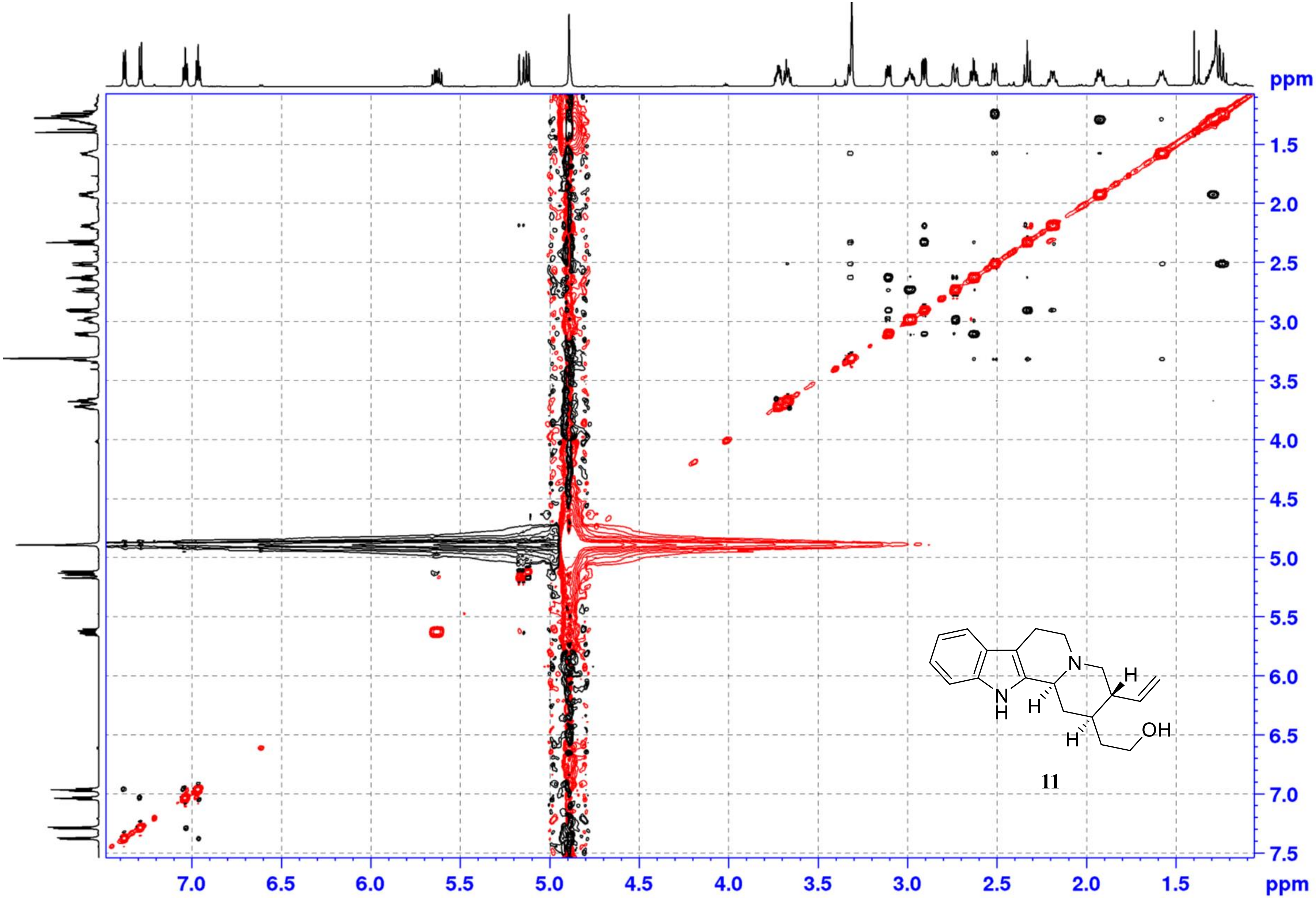

Supplementary Fig. 45. ROESY NMR spectrum of corynantheol (11) in methanol-d_3.

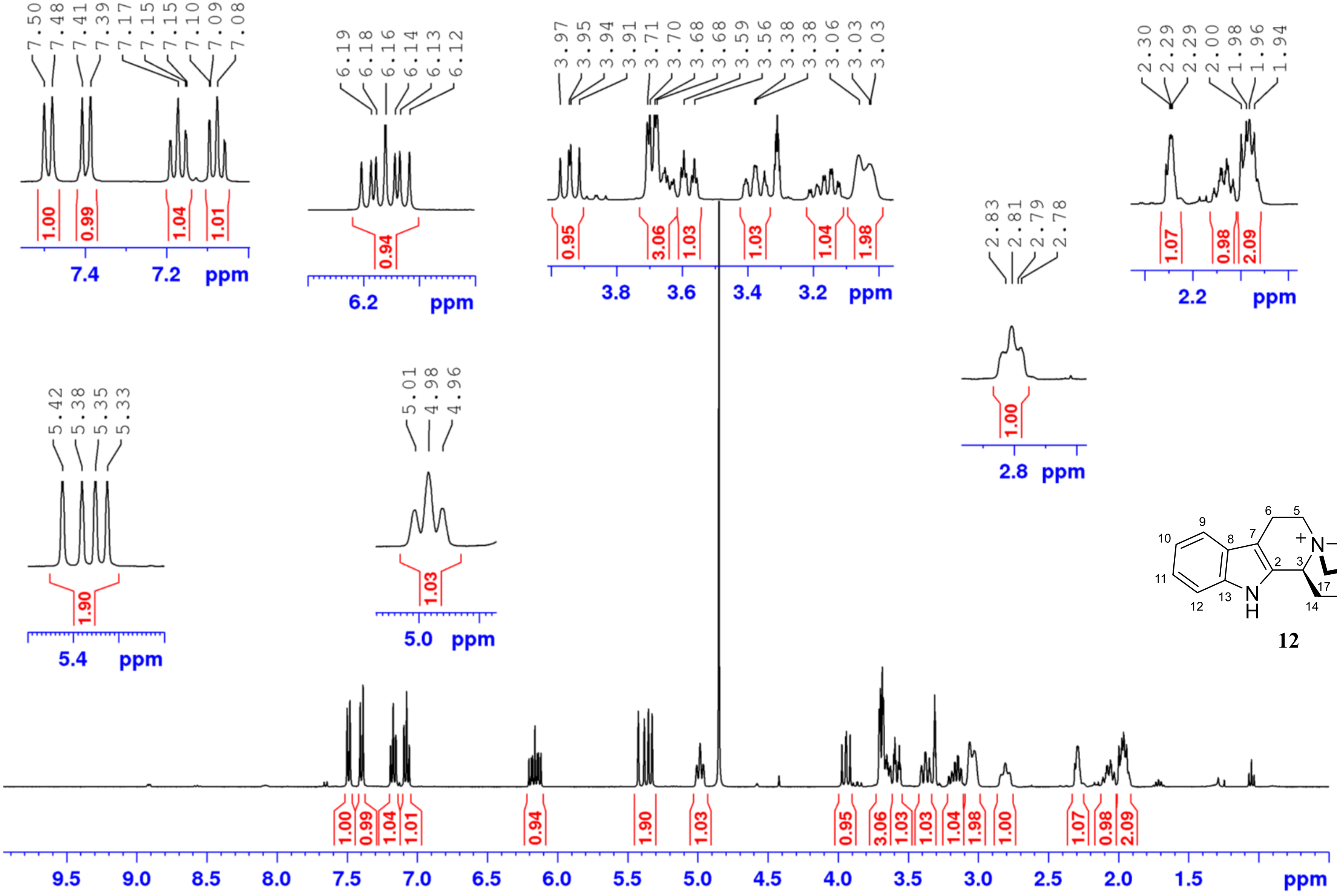

Supplementary Fig. 46. ^{1}H NMR spectrum (400 MHz) of cinchonium (12) in methanol-d_4.

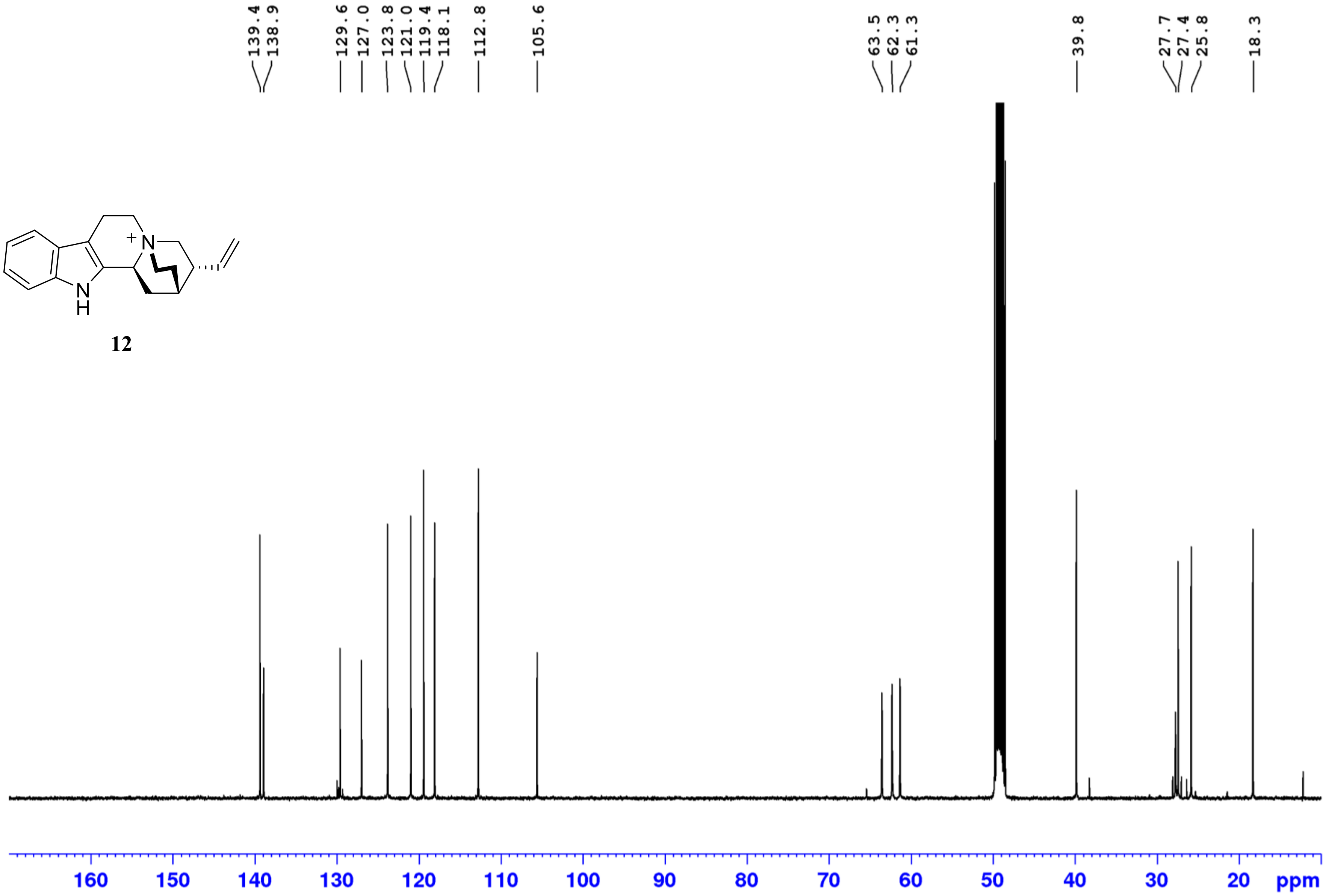

Supplementary Fig. 47. ^{13}C NMR spectrum (100 MHz) of cinchonium (12) in methanol-d_4.

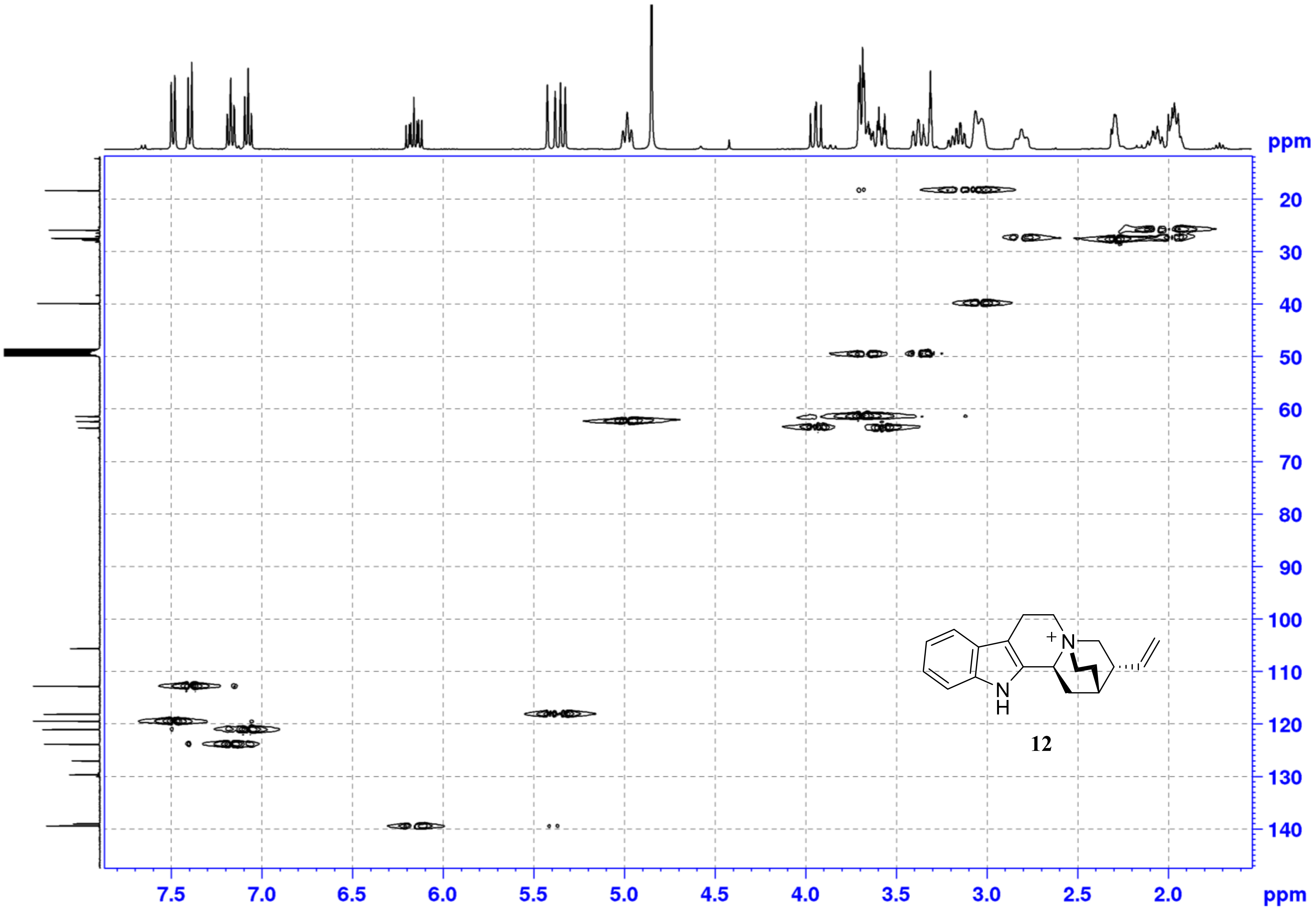

Supplementary Fig. 48. ^{1}H-^{13}C HSQC NMR spectrum of cinchonium (12) in methanol-d_4.

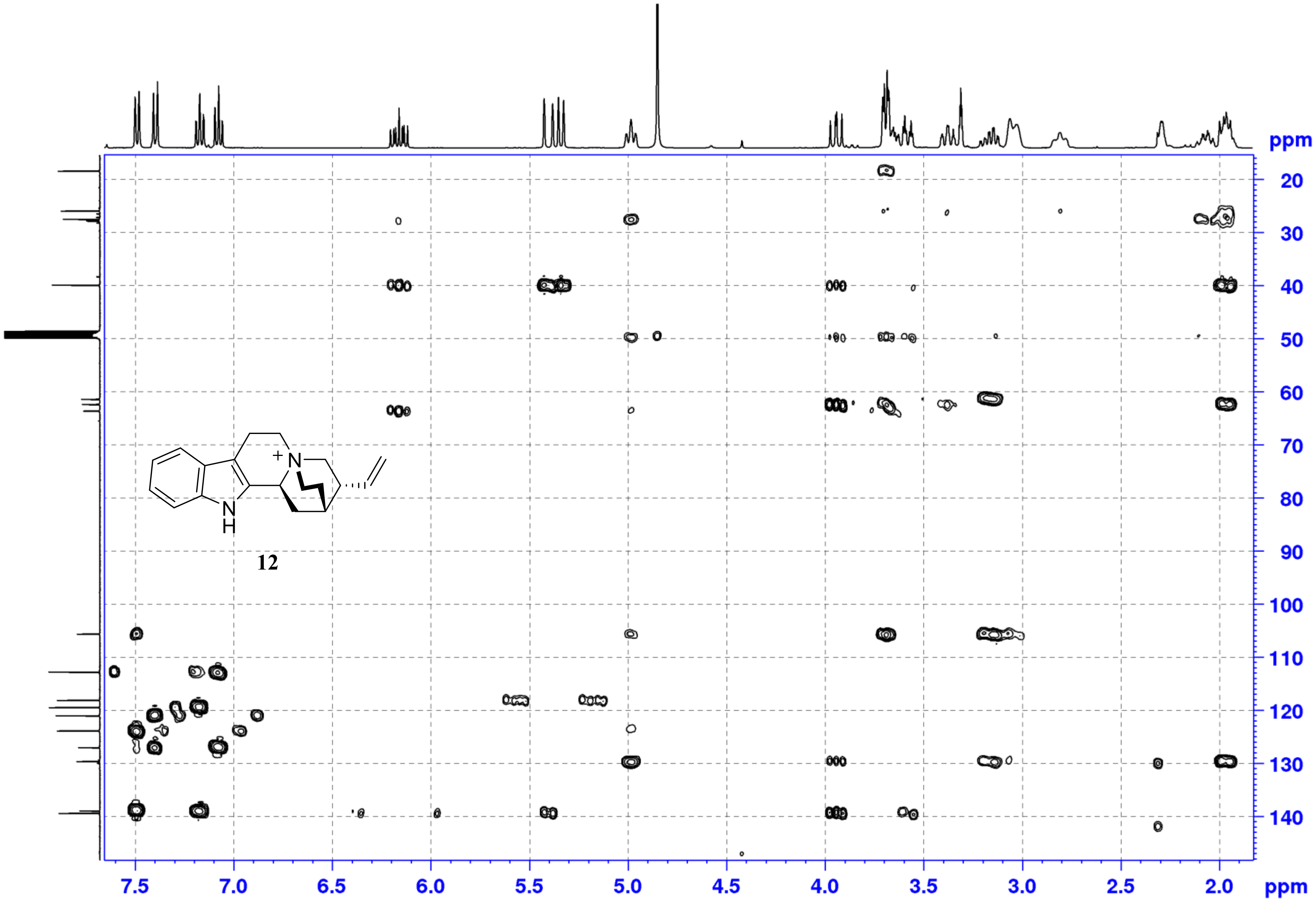

Supplementary Fig. 49. HMBC NMR spectrum of cinchonium (12) in methanol-d_4.

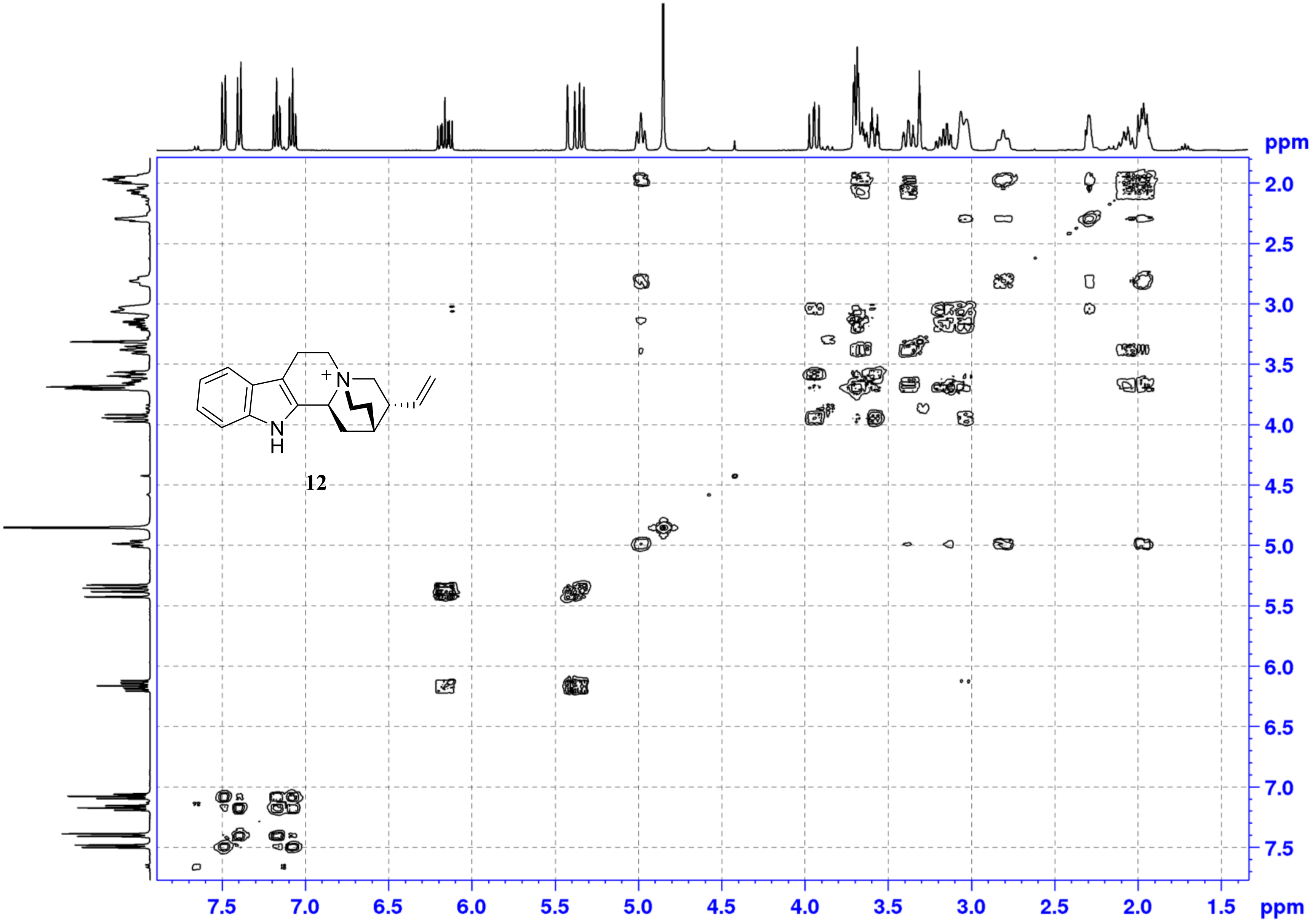

Supplementary Fig. 50. COSY NMR spectrum of cinchonium (12) in methanol-*d*4.

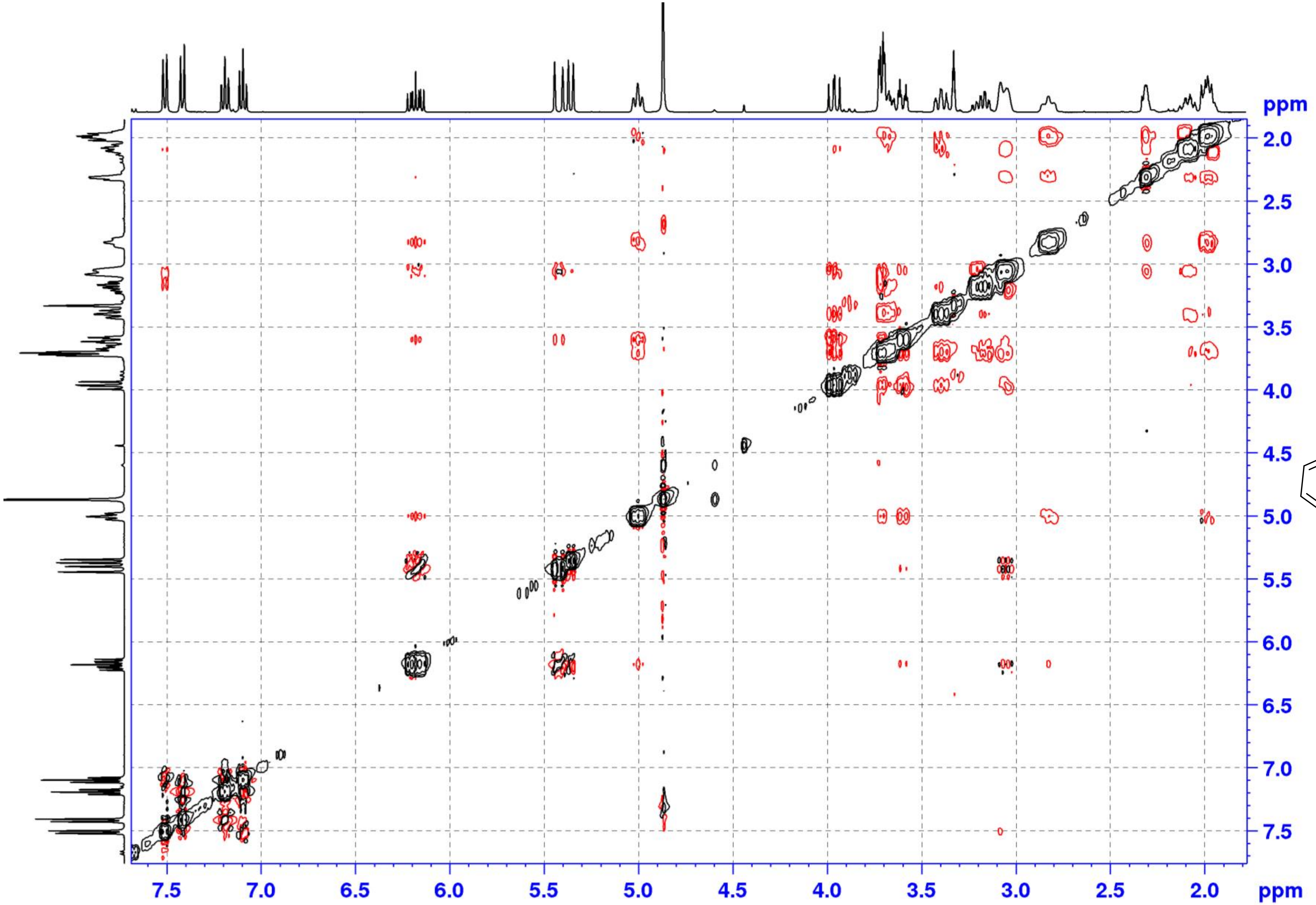

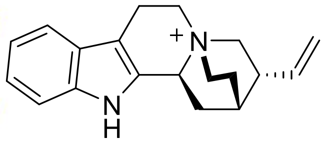

12

Supplementary Fig. 51. NOESY NMR spectrum of cinchonium (12) in methanol-d_4.

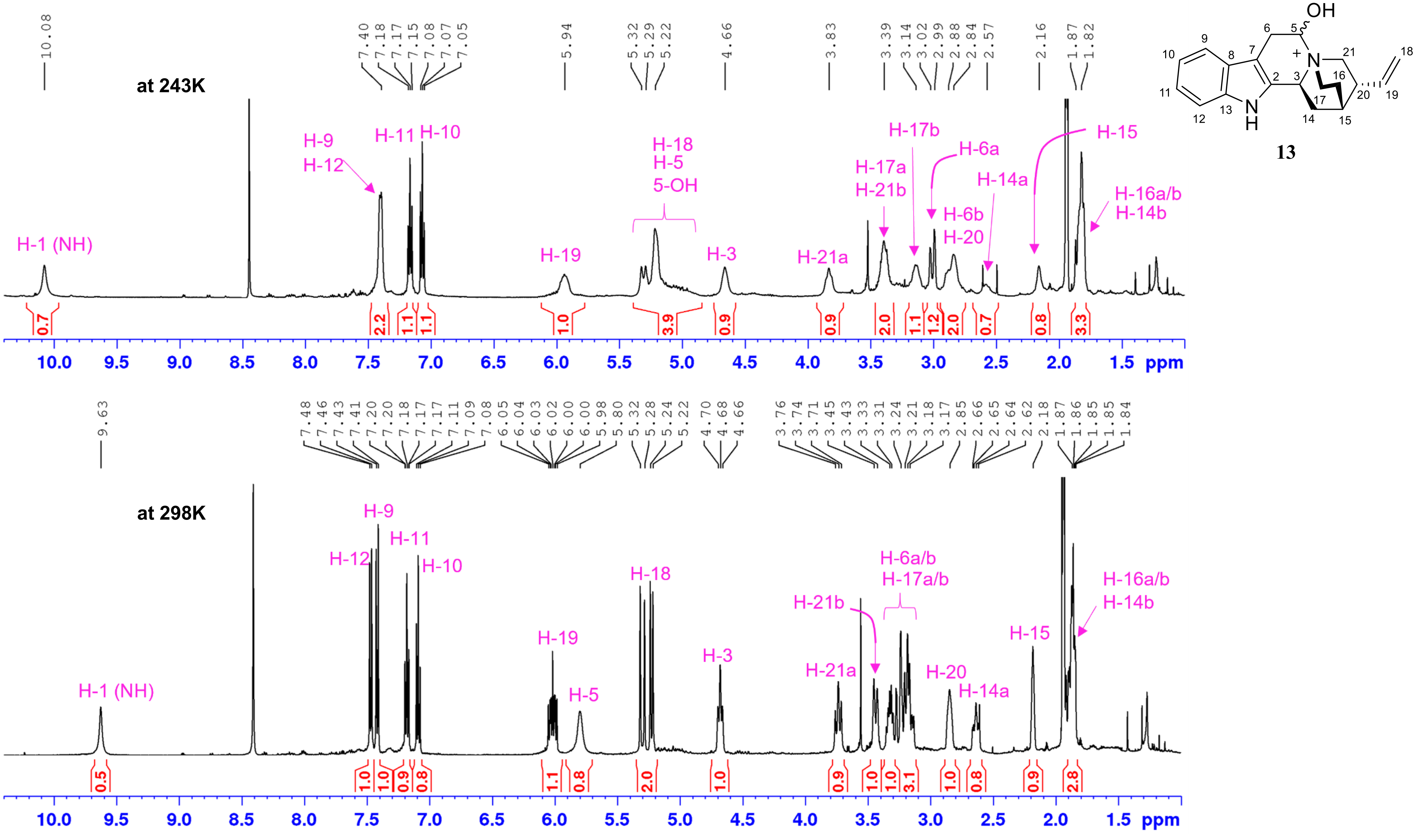

Supplementary Fig. 52. **^{1}H NMR spectra (500 MHz) of cyclocinchonaminal (13) at 243K (top) and 298K (bottom) in MeCN-d_3.**

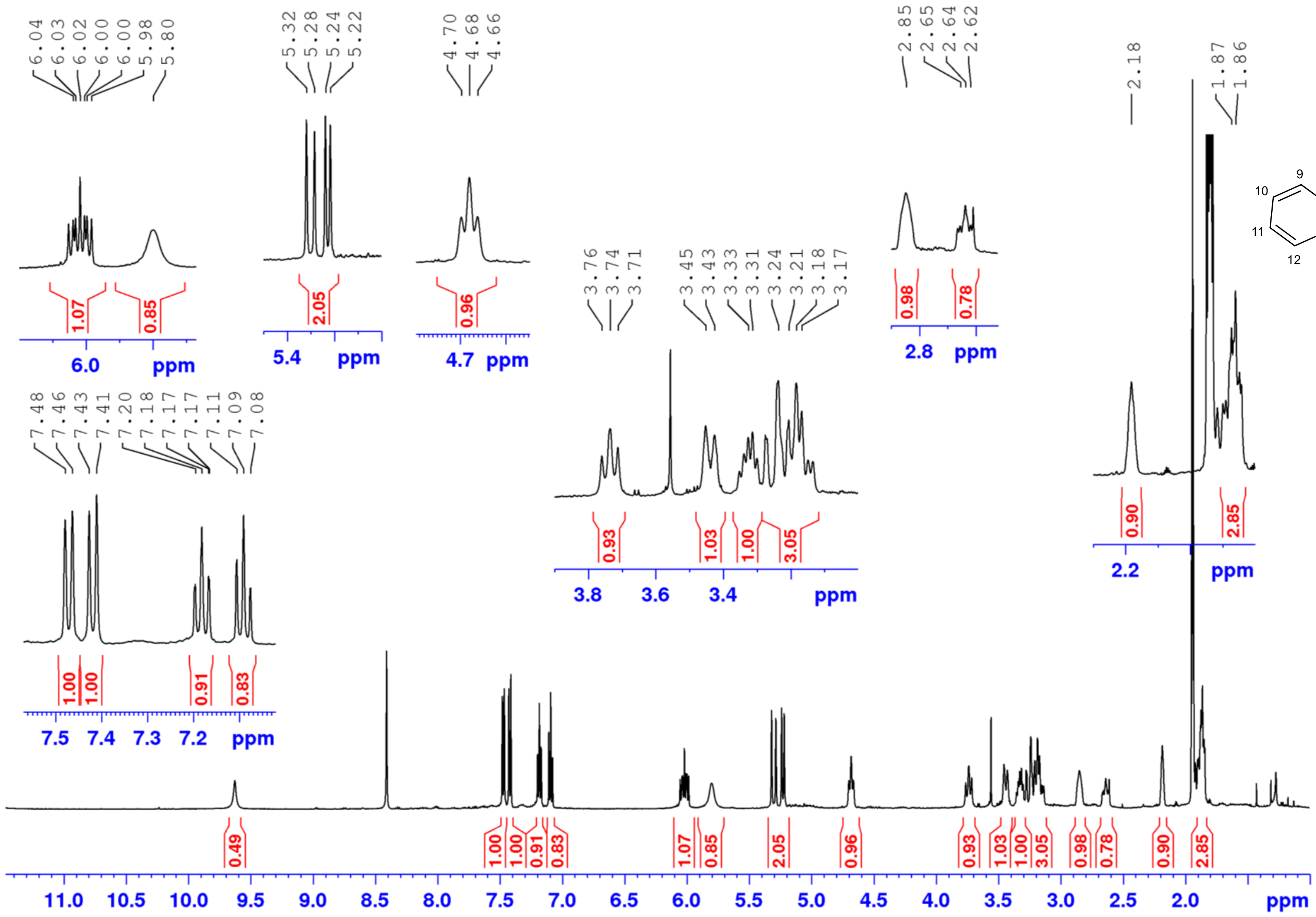

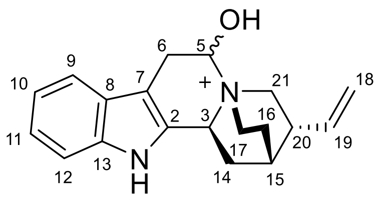

13

Supplementary Fig. 53. Enlarged ^{1}H NMR spectrum (500 MHz) of cyclocinchonaminal (13) at 298K in MeCN-d_3.

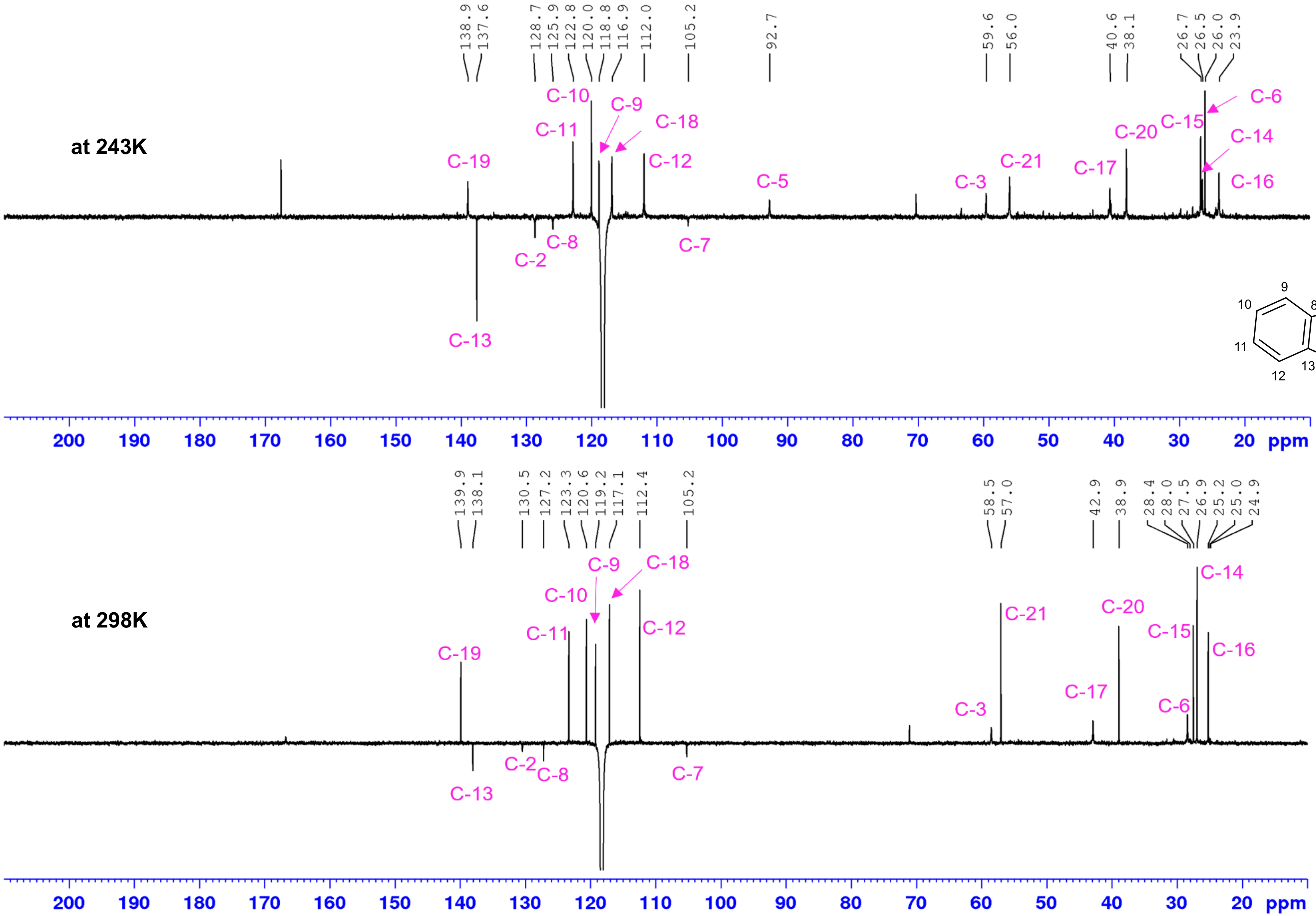

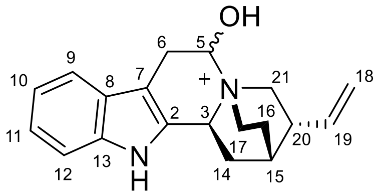

13

Supplementary Fig. 54. DEPTQ NMR spectra of cyclocinchonaminal (13) at 243K (top) and 298K (bottom) in MeCN-*d*3.

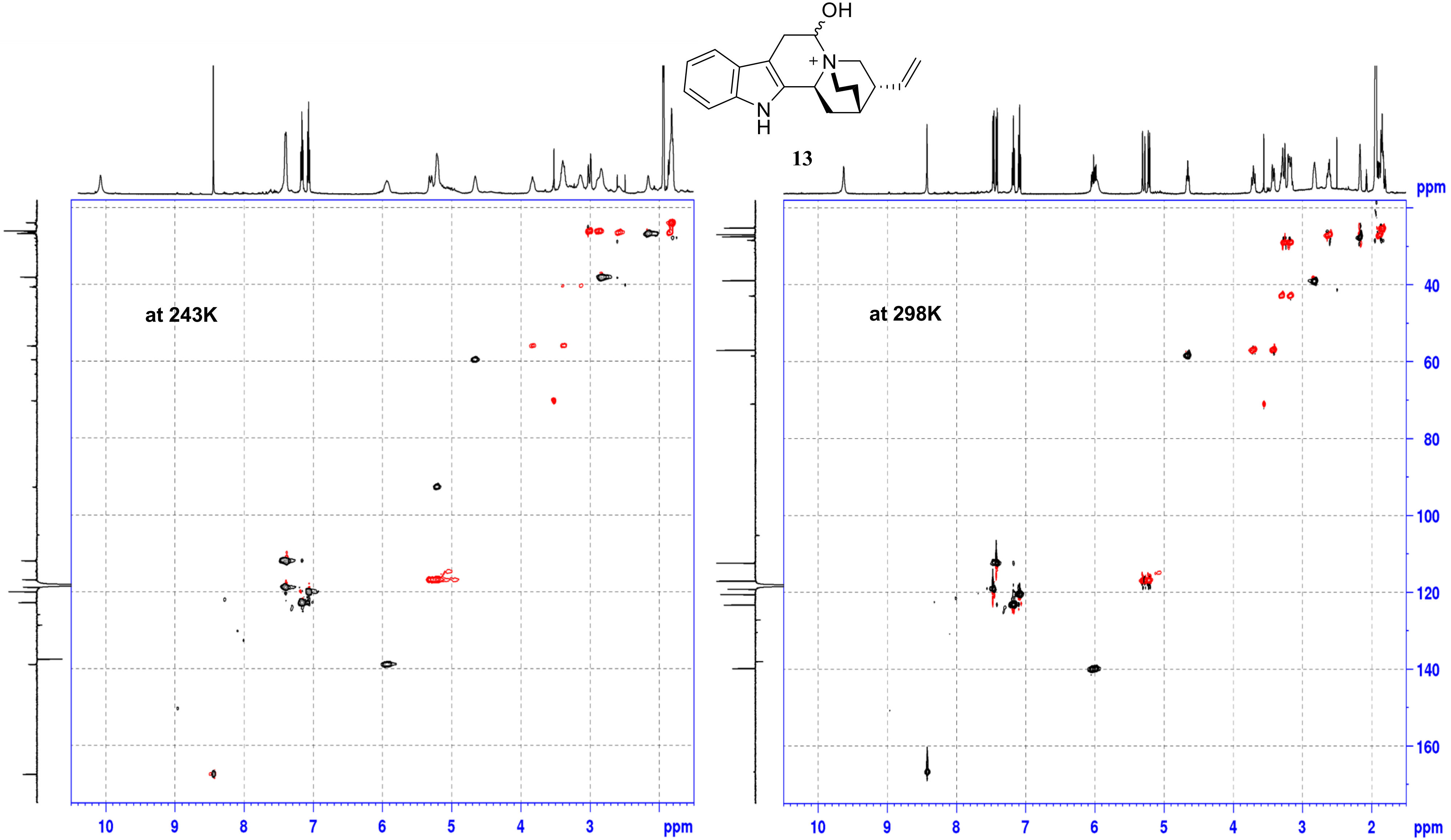

Supplementary Fig. 55. Phase-sensitive ^{1}H-^{13}C HSQC NMR spectra of cyclocinchonaminal (13) at 243K (left) and 298K (right) in MeCN-d_3.

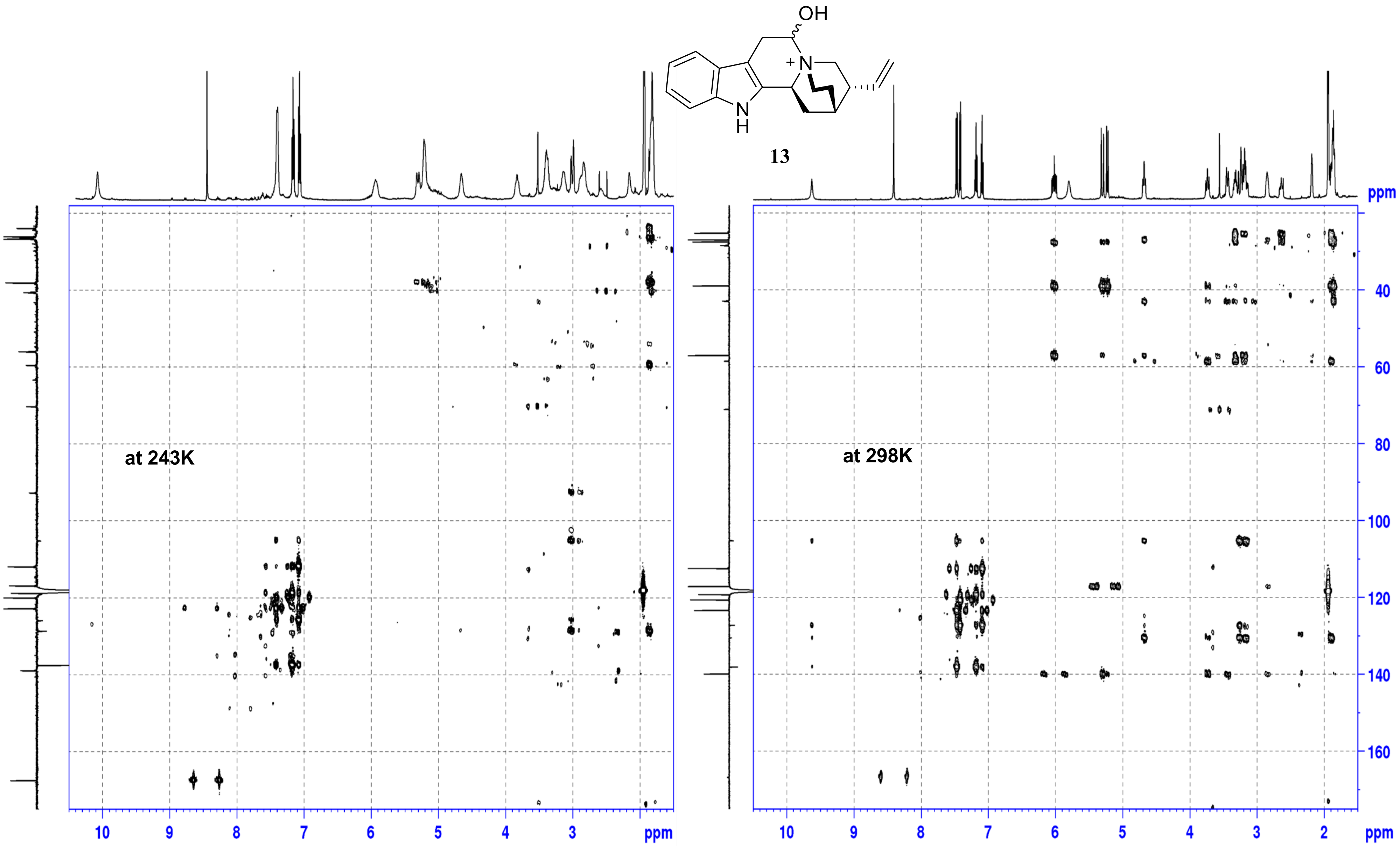

Supplementary Fig. 56. HMBC NMR spectra of cyclocinchonaminal (13) at 243K (left) and 298K (right) in MeCN-d_3.

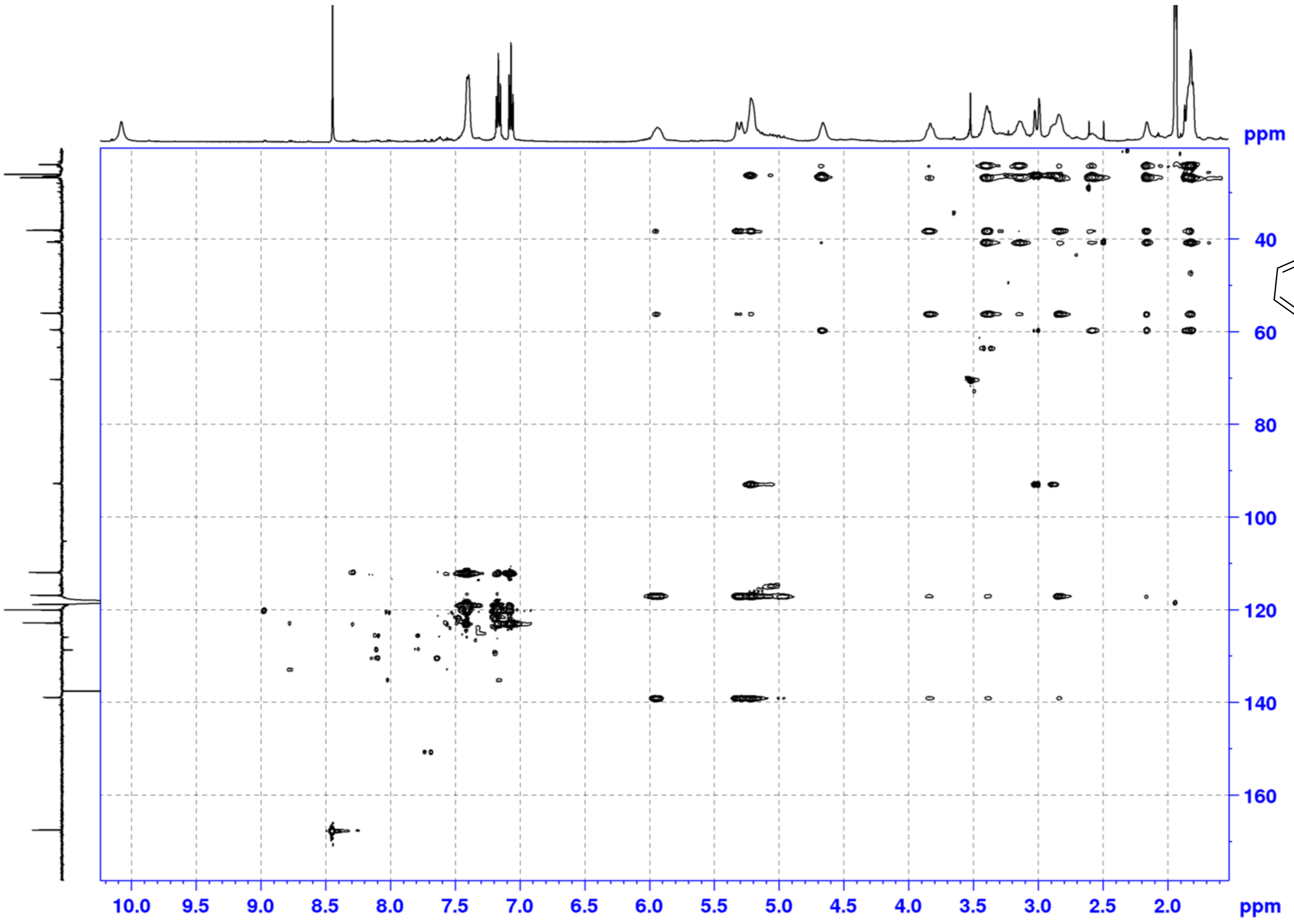

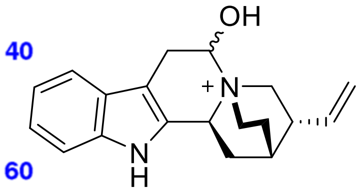

13

Supplementary Fig. 57. HSQC-TOCSY NMR spectrum of cyclocinchonaminal (13) at 243K in MeCN-*d*3.

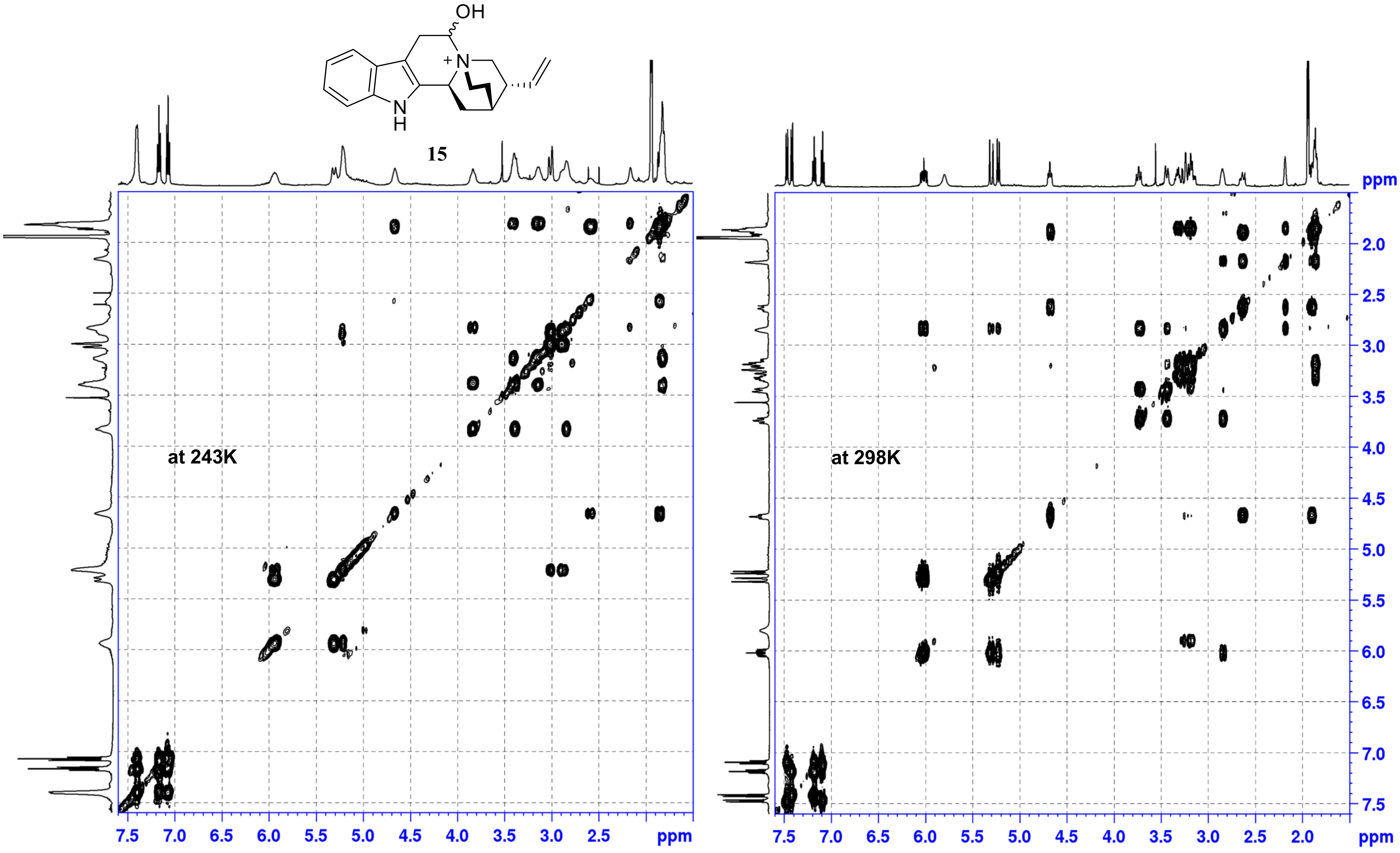

Supplementary Fig. 58. DQFCOSY NMR spectra of cyclocinchonaminal (13) at 243K (left) and 298K (right) in MeCN-*d*3.

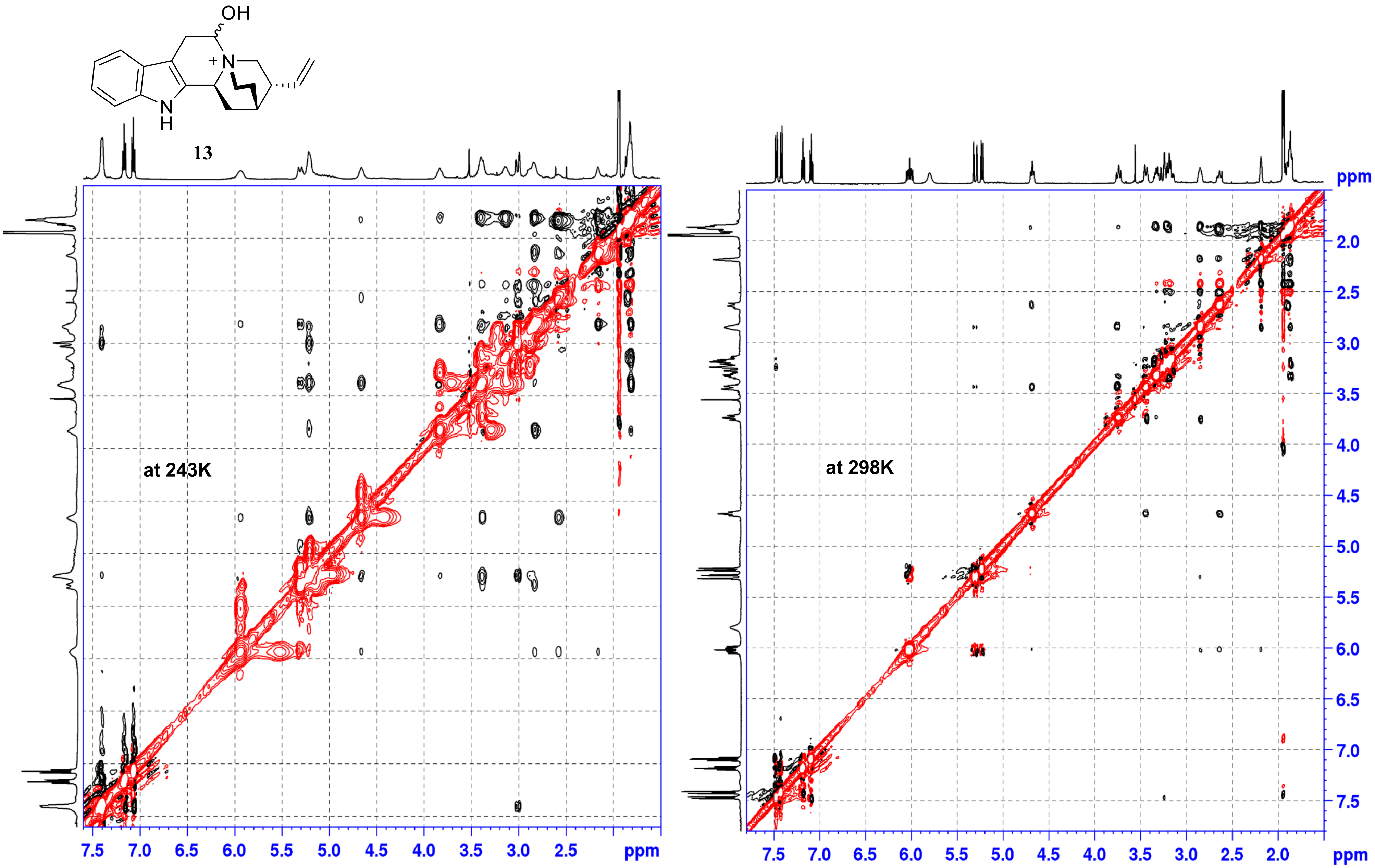

Supplementary Fig. 59. ROESY NMR spectra of cyclocinchonaminal (13) at 243K (left) and 298K (right) in MeCN-d_3.

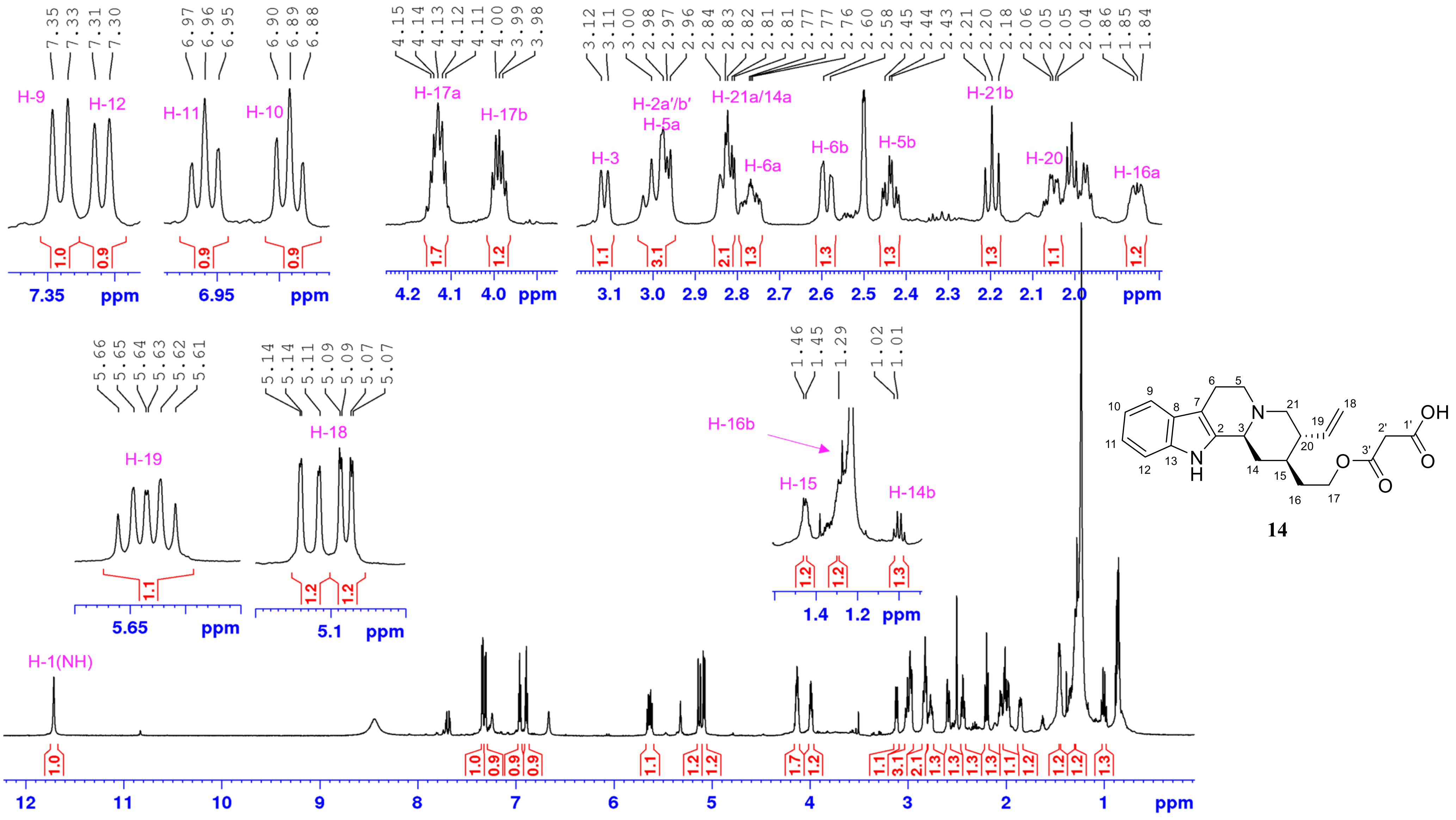

Supplementary Fig. 60. ^{1}H NMR spectrum of malonyl-corynantheol (14) in DMSO-d_6.

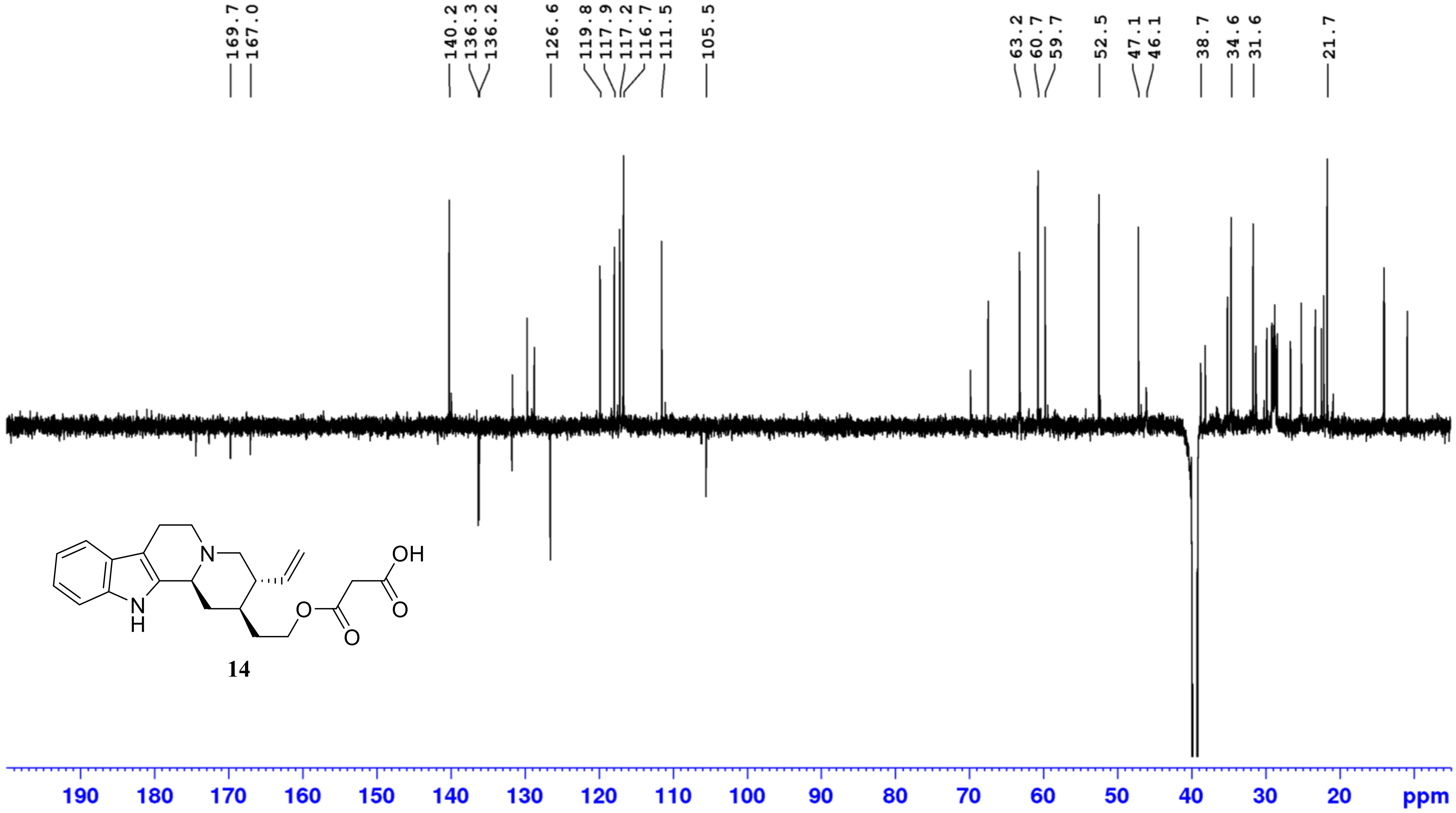

14

Supplementary Fig. 61. DEPTQ NMR spectrum of malonyl-corynantheol (14) in DMSO-d_6. Unlabeled peaks belong to unidentified impurities.

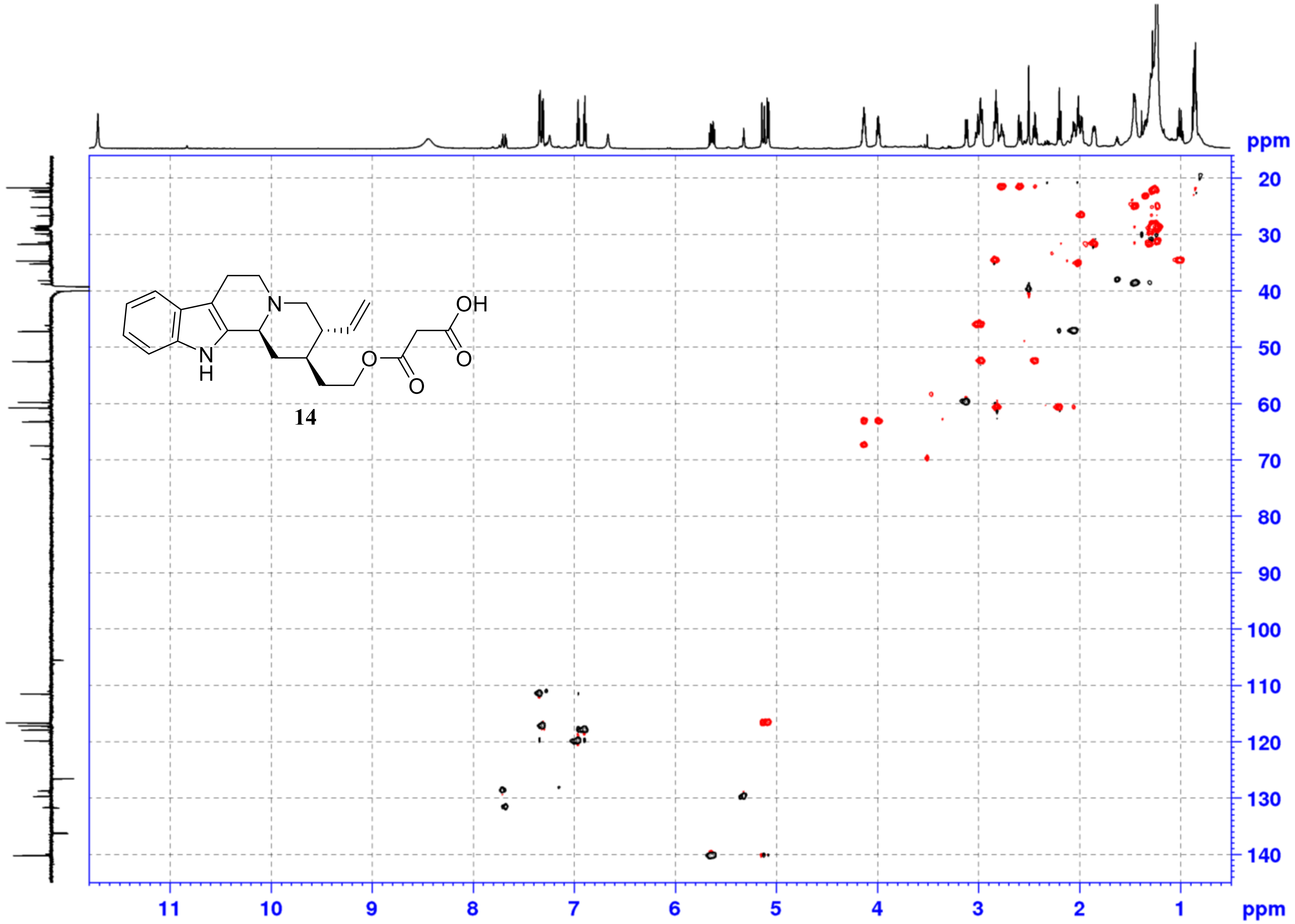

Supplementary Fig. 62. **^{1}H-^{13}C phase sensitive HSQC NMR spectrum of malonyl-corynantheol (14) in DMSO-*d*$_6$.**

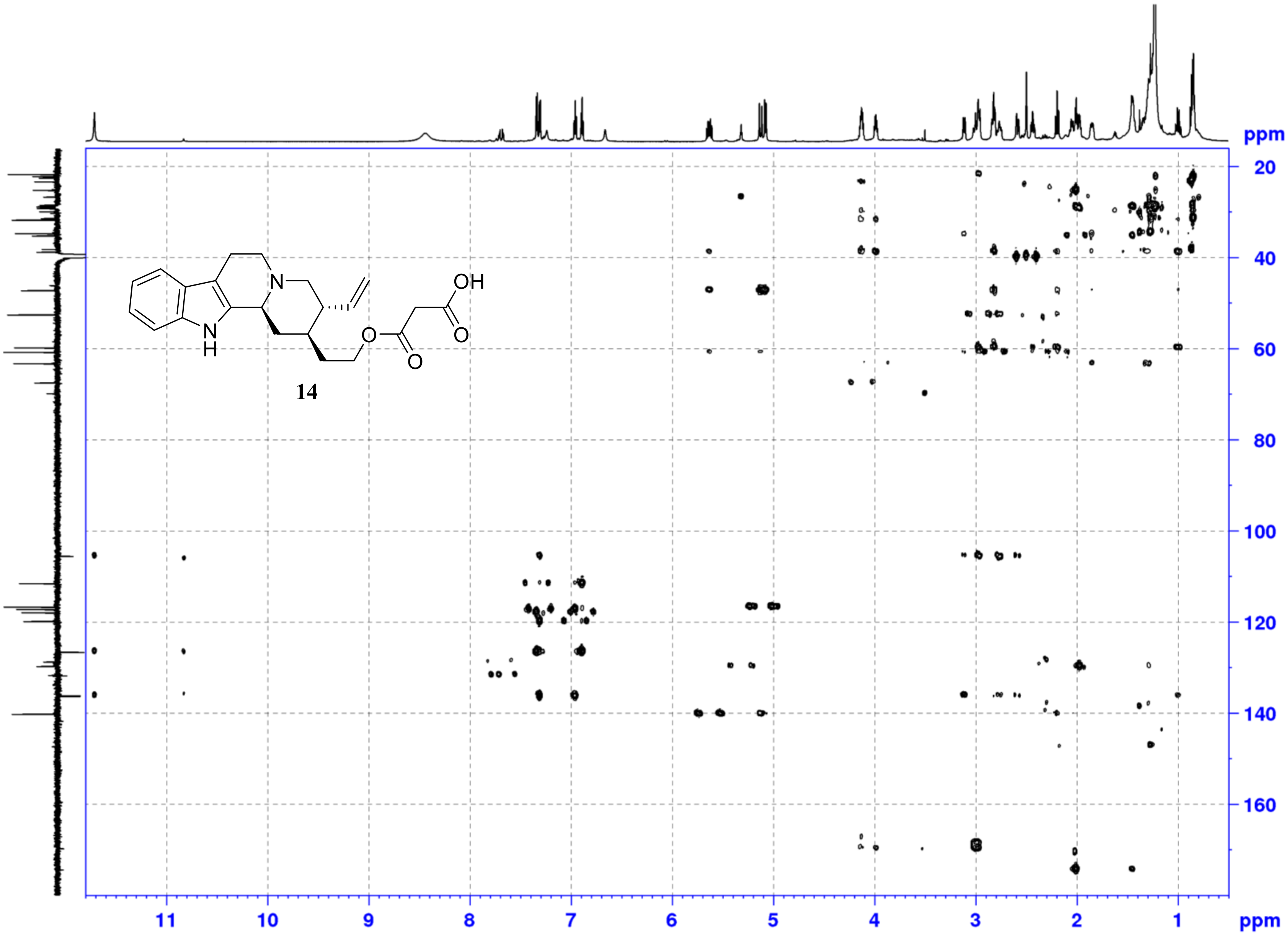

Supplementary Fig. 63. **^{1}H-^{13}C HMBC NMR spectrum of malonyl-corynantheol (14) in DMSO-d_6.**

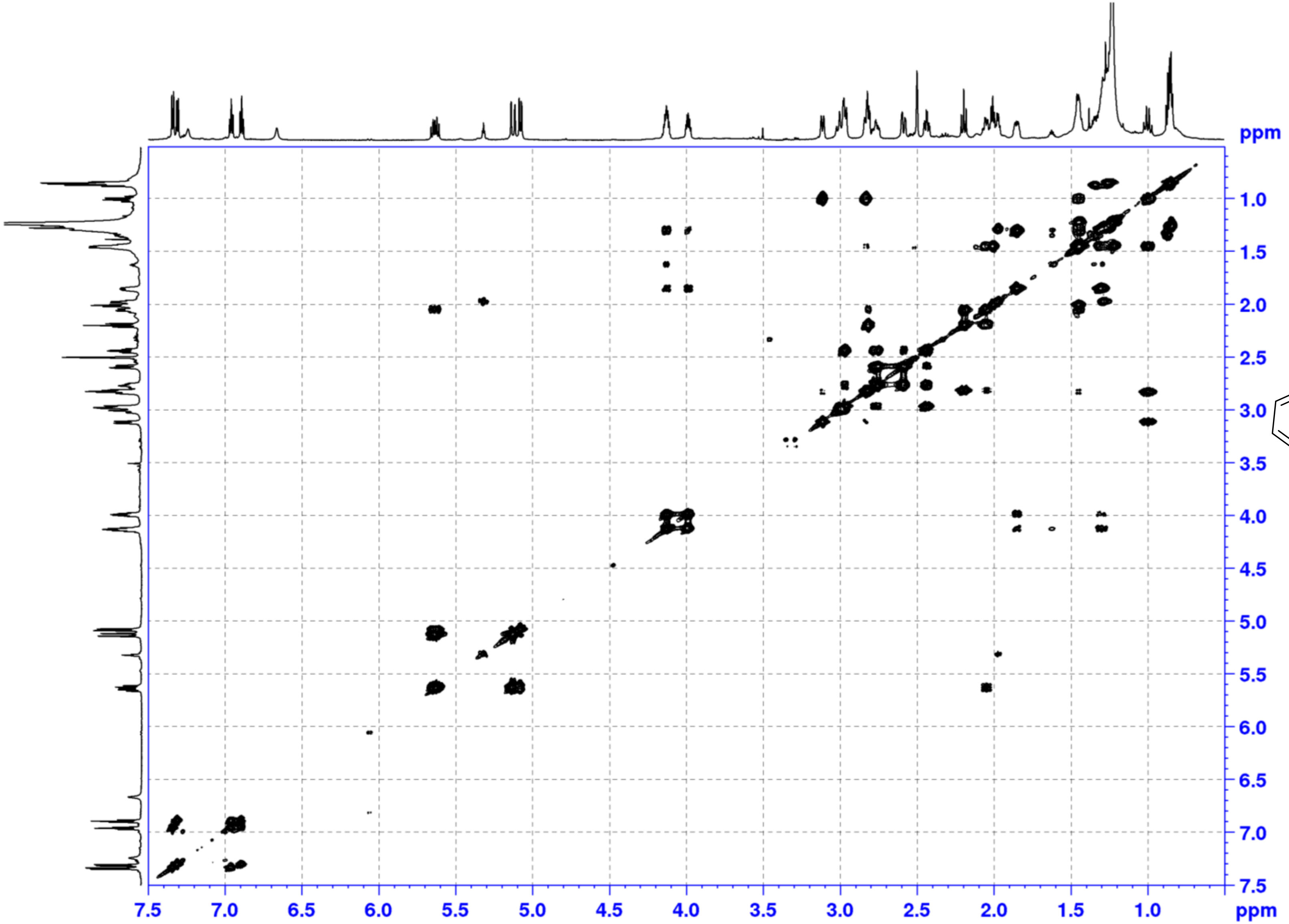

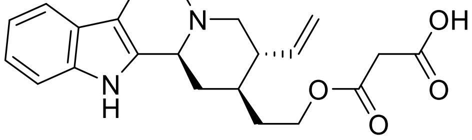

14

Supplementary Fig. 64. COSY NMR spectrum of malonyl-corynantheol (14) in DMSO-d_6.

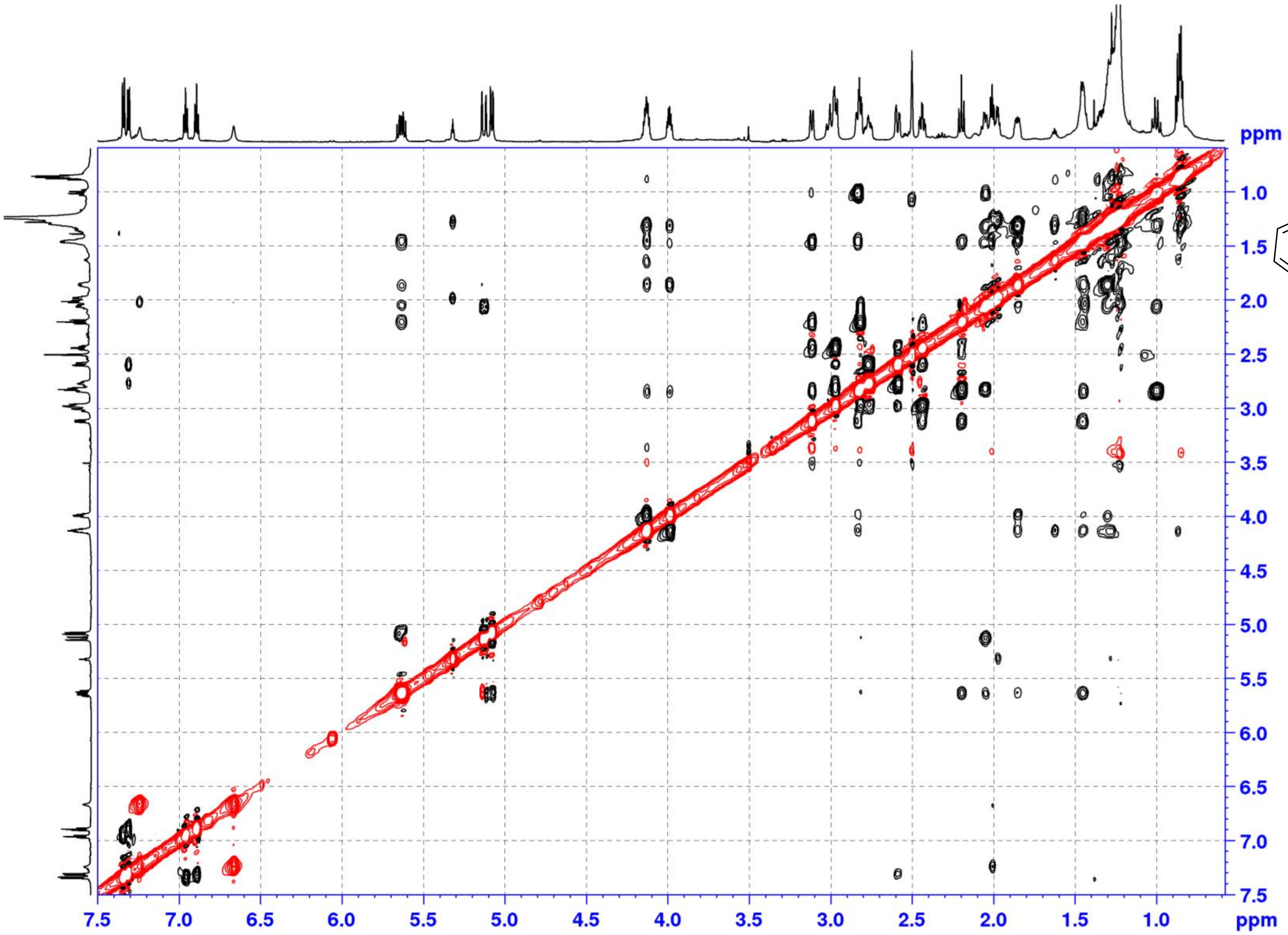

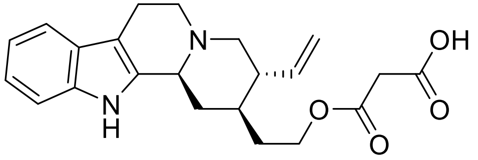

14

Supplementary Fig. 65. COSY NMR spectrum of malonyl-corynantheol (14) in DMSO-*d*6.

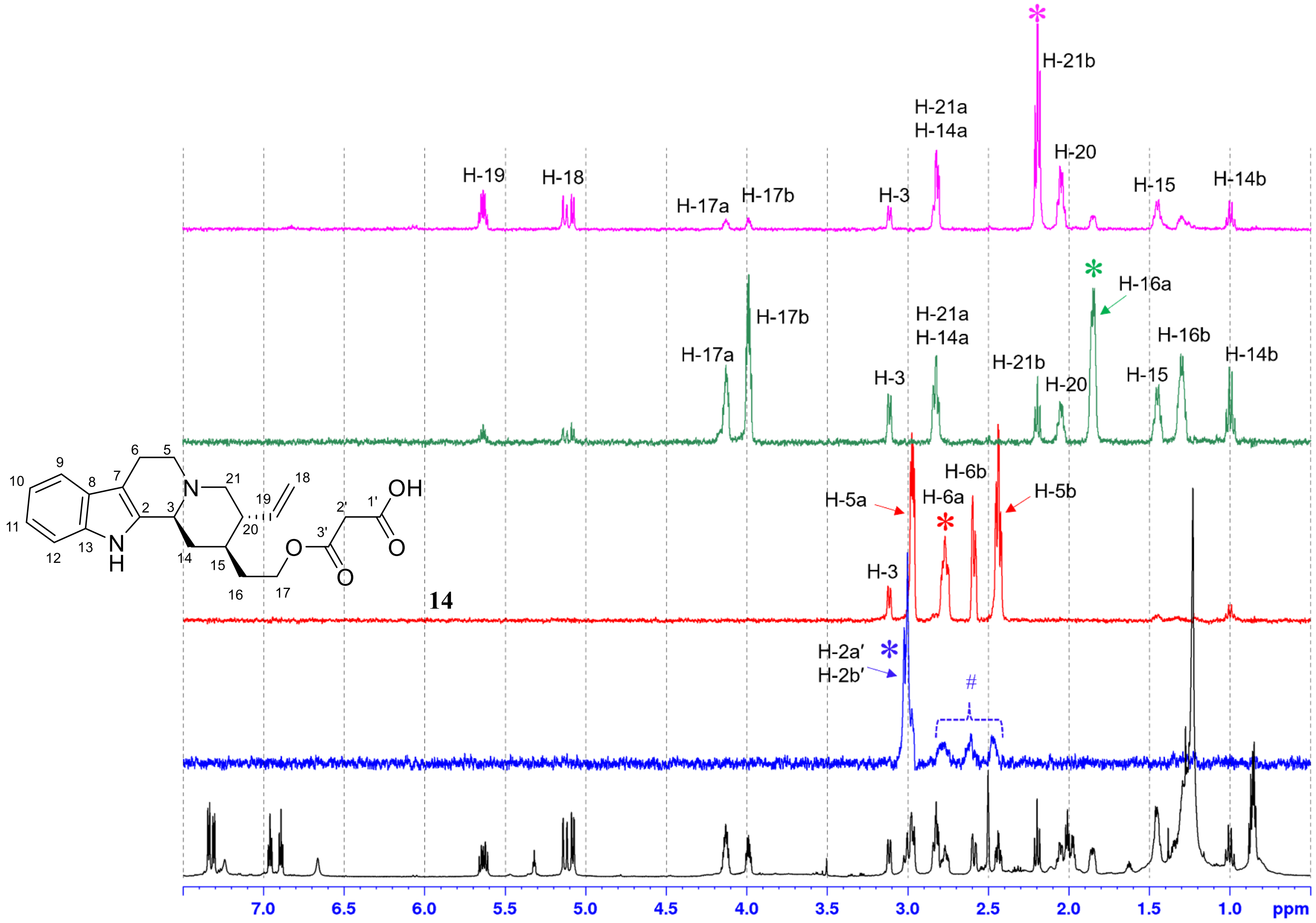

Supplementary Fig. 66. **^{1}H (black) and SELTOCSY (blue, red green, and pink) NMR spectra of malonyl-corynantheol (14) in DMSO-*d*$_6$.** In SELTOCSY spectra, the transmitter signals are indicated with an asterisk. # designates marginal enhancement of H-6a/b and H-5b, resulting from residual transmission from H-5a signal which partly overlaps with H-2′a/b.

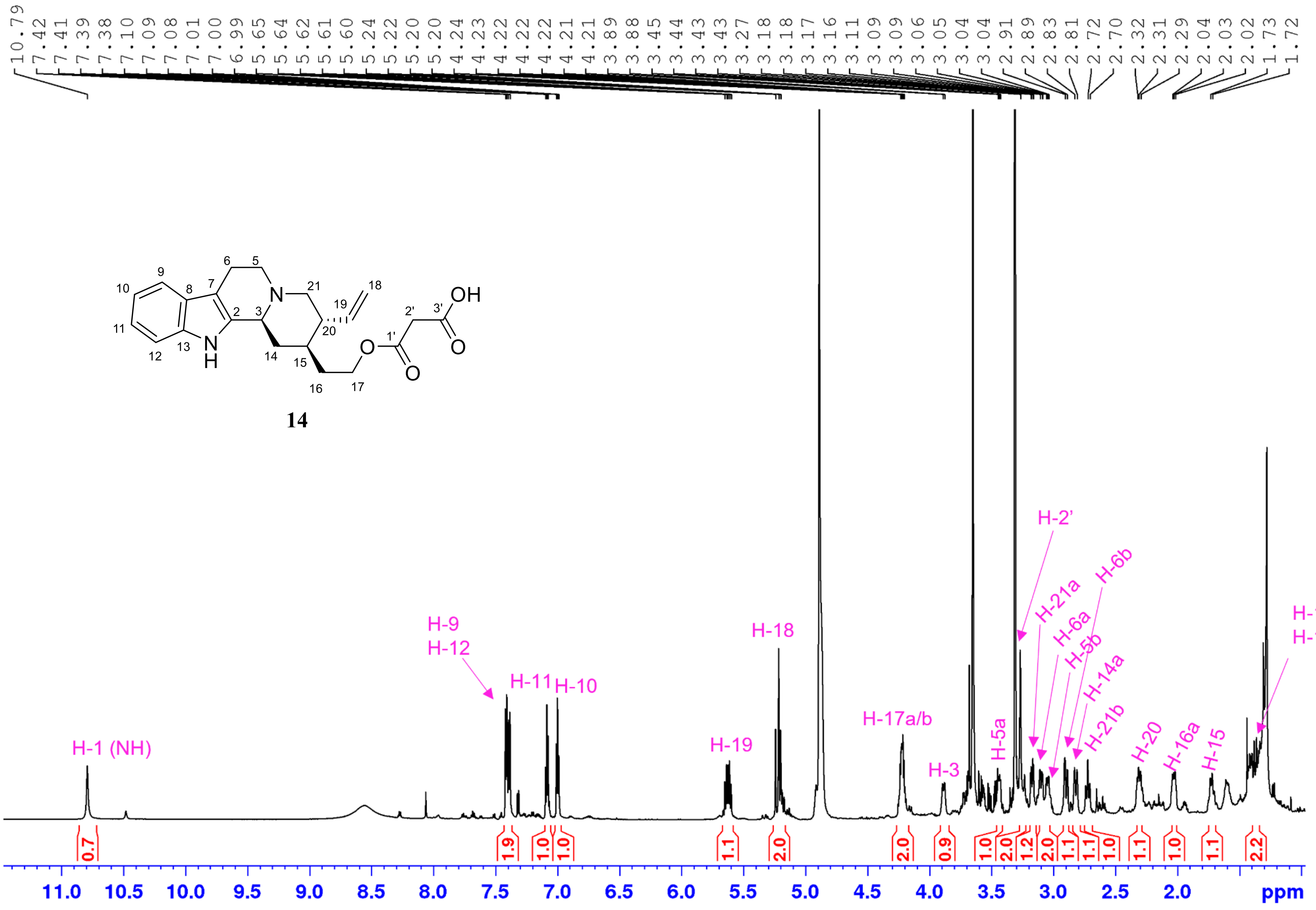

Supplementary Fig. 67. ^{1}H NMR spectrum (700 MHz) of malonyl-corynantheol (14) in methanol-*d*3.

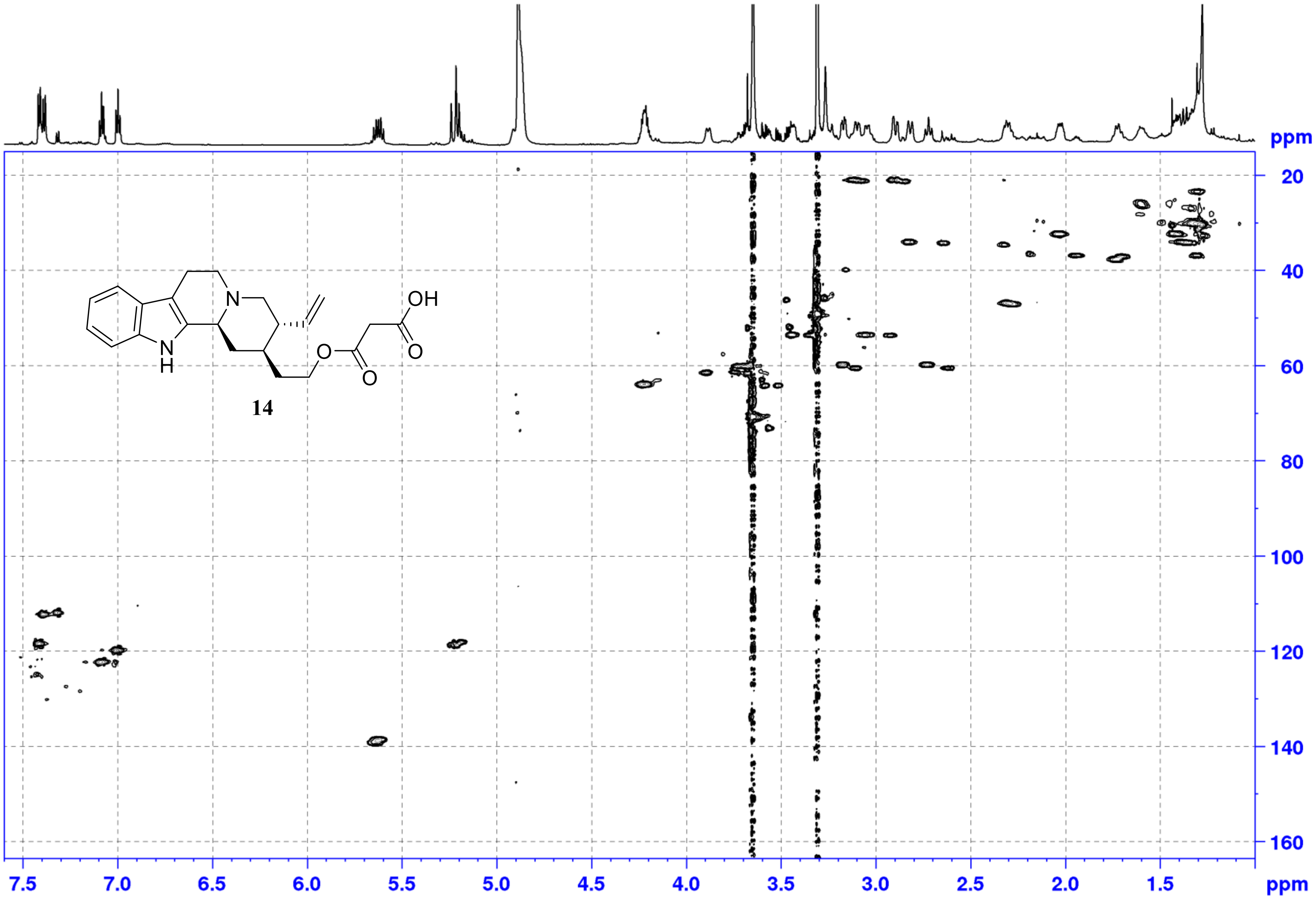

Supplementary Fig. 68. ^{1}**H-**^{13}**C HSQC NMR spectrum of malonyl-corynantheol (14) in methanol-*d*$_3$.**

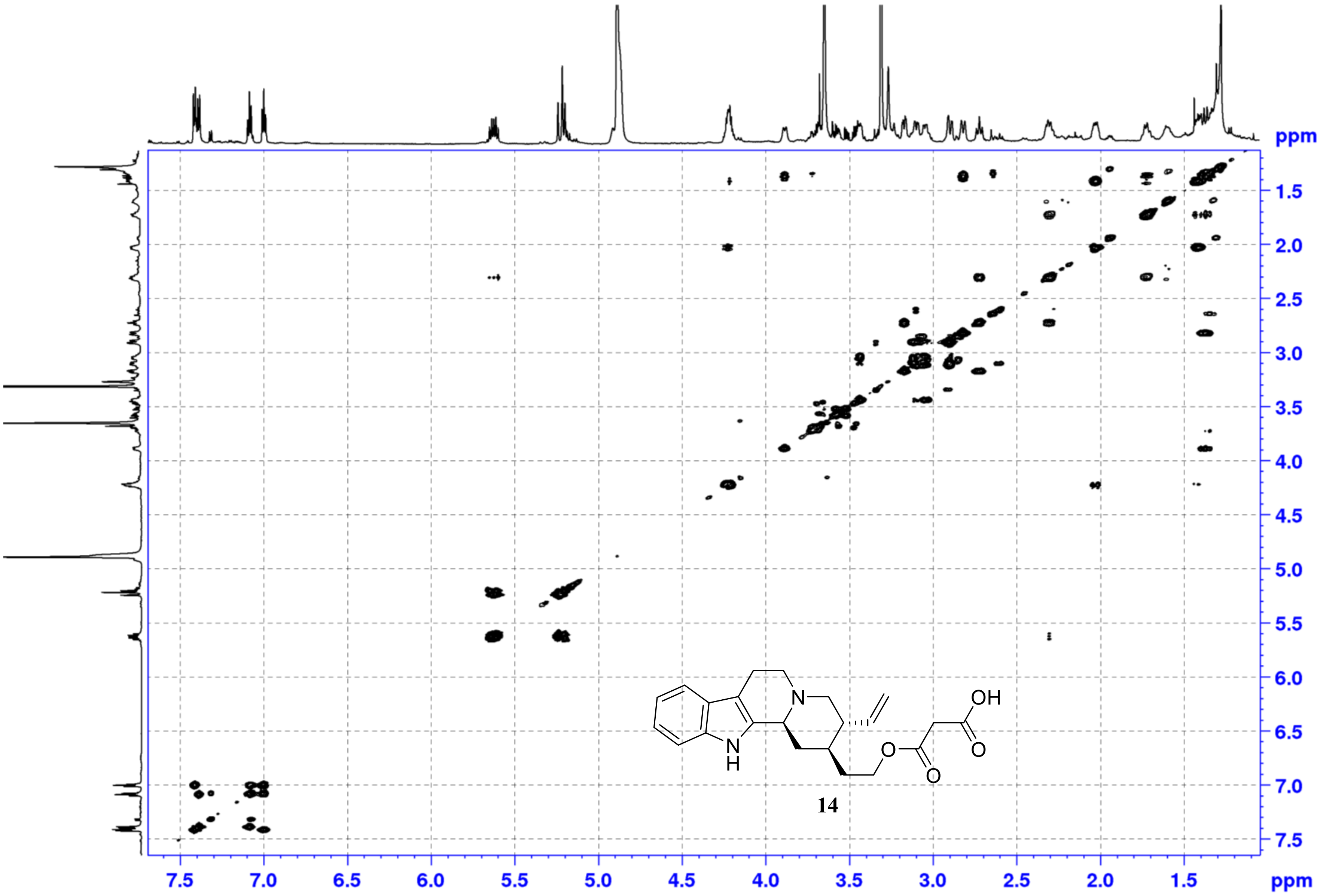

Supplementary Fig. 69. COSY NMR spectrum of malonyl-corynantheol (14) in methanol-d_3.

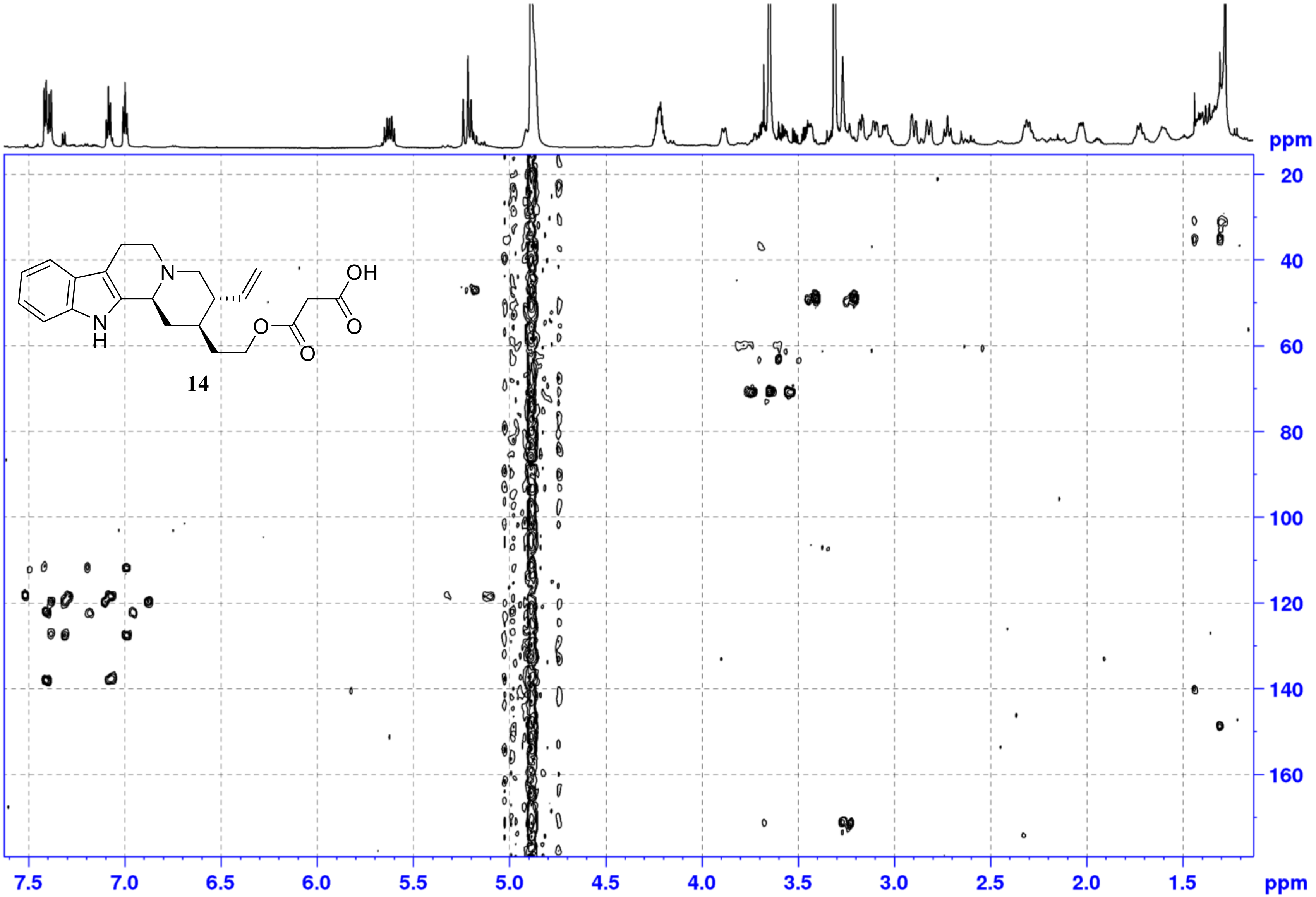

Supplementary Fig. 70. HMBC NMR spectrum of malonyl-corynantheol (14) in methanol-d_3.

Table S1: General assembly statistics for the *Cinchona pubescens* genome described and used in this study.

Genome Version	Metric
Number of scaffolds	5,158
Total size of scaffolds	917,026,882
Longest scaffold	9,773,304
Shortest scaffold	10,001
Number of scaffolds > 1K nt	5,158
Number of scaffolds > 10K nt	5,158
Number of scaffolds > 100K nt	1,280
Number of scaffolds > 1M nt	217
Number of scaffolds > 10M nt	0
Mean scaffold size	177,787
Median scaffold size	27,660
N50 scaffold length	986,242

Table S2: General statistics for *C. pubescens* leaf single-nuclei sequencing data described and used in this study.

Sample filtering criteria used for single-nuclei library preparation					
Library	Minimum Features	Maximum Features	Minimum UMIs	Maximum UMIs	Doublets Removed
CPU_AG	300	10,000	500	30,000	2,478
CPU_AH	300	10,000	500	30,000	2,274

Statistics of sequencing metrics and gene expression							
Library	Status	Number of nuclei	Total number of genes detected	Mean genes per nucleus	Median genes per nucleus	Mean UMIs per nucleus	Median UMIs per nucleus
CPU_AG	Doublet filtered	32929	36472	1909	1814	3178	2850
CPU_AH	Doublet filtered	30211	36273	1980	1856	3282	2893
CPU_Leaf	Integrated	63140	40513	1943	1835	3228	2871

Table S3. Nucleotide sequences for genes described and used in this study.

**Gene name (GenBank accession)**	**Nucleotide sequence**
*Cp*MAT<br>(PX842829)	>ATGGCGTCAACAATTATGATCATGGATATCCCCAACCTTCTTGAGATAATTTC<br>TGAAAGATTCGTCAAACCTTCCTCTCCTACCCCTCCTTCAACATGTCACAAACT<br>CTCCTACAAAGAACAAATAATGTGCCACGAATATATCCCCTGGGCCTTCTTCT<br>ACCCCTGCCCAAGCAAATCAAGTACTGAAATATTTGAACTTCTTGAAACTTCC<br>GTATCAAAAACTCTAACCTACTACTATCCCTTCGCCGGAAGGCTTACCAACAA<br>TTCCTTTGTGGATTGCCATGACAAGGGAGTTCAGTTCACAGAAGCCCGGATCA<br>AGTGTTCGATGAATGAAGTTCTCGATCCACCCAATTCTCCTCTTAGAGATCTTG<br>TTTTCCCTCGATTTCTATTTTCTGGCACGCCCCCGGAAGATGGCAGCCTGTTGA<br>TTGTTCAGGTCAGCTATTTTGACTGTGGAGGCGTGGCTGTAGGCCTCTGTATAT<br>CTCACAAGATCTCTGATGCGTCCACTAGATGCACTTTGAGCAATGATTGGGCT<br>GCCGTGGCACGCCAGCCGAGTTATGTCCCGACTCCAAAGTTTAACGGAGCTTC<br>TGTCTTTCCACCAGTCGATGACGTATCGTTTCAGGAACTCATCGCTTCGCCACC<br>AACTGAAAATTGCGTTGCTAAGAGATTCTTGTTTAAGGCCTCCAAAATTGGGG<br>AACTCAAGGCTATGGCTTCTGATTCAGGACTGGATCGTCCTACTCGAGTTGAA<br>GTCGTGACTGCACTACTTTATAGGTGTGCCATGGCTGCAACGAGGGCAAATTC<br>GGGTTCTTTTCGGCCTTCAATGTTGTTTAATGCTGCCAACTTGAGATCCATAAC<br>TGTCCCTCCACTACCACAGAATTCCATTGGCAATTTCATTACTTTTTTCCCCAT<br>ATCAACGTCTGAAGAAGACGACACAAAATTGTCCGAACTGGTTCATAAGTTTA<br>GGAATGCAAAATTGAAACTCCTTGAAGAGTACAAGGAGAAGGCCAATGCAAA<br>TGATGAATTTGCCTCGTCGCTATCTGCTTCTGAGAATGGAAATGAAACTAATC<br>AGAATTTTGATGTTTATTATTCCAGCAGTTGGTGTAGATTTCCGTTTTATGAAG<br>TTGATTTTGGCTTTGGAAAGCCTTTATTGGTATGTACGACCAATGAATGTGGA<br>GCCAAGAACAACTTCATTTTGATGGATACCAAAGATGGGGATGCAATAGAAG<br>CTGCAGTTACTCTAGAAGAATCAGACATGTCCATCTTCCAAGGTAATGAAGAG<br>CTCCTTGCATTTGCTTCTTTGGTTTCATAA
*Cp*MCC<br>(PX842830)	>ATGGCTTCAAGCAAAACAGTGACTTTTCTTGAGCAATGCCATGTTTCACCAC<br>CAGCATCCACCACTGCAGATTTATCACTCCCCTTAACGATTTTCGACGCGCCGT<br>GGATAGGCTTTCATGTAATTCAGCGTCTTTTCTTCTTCAATTTCAATCACAACA<br>AGACTTATTTCATTGAAAACACTATTCCGAGTCTCAAAGAATCCCTTTCCCTCA<br>CACTCAAACATTTCTTTCCTCTTGCTGGCAATCTGATATATCCTTCAGACTCTT<br>CTGGCAAGCCTGAAATAAAGTACTCTATTGGTGACTCCATTTCCTTGACTTTTG<br>CTGAATCCAATGATGATTTTAGCTACCTTGTCGGAAATGATCCGAAAACTGCC<br>CACAGATTCTACCCTTTAGCCCCTGAATTGAAACTAGCCTCCTCTGAAATATC<br>AGGCAACAAAATCGTTCCCCACTTAGCTTTGCAAGTGACATTGTTTCCAAATT<br>CTGGTATATGCATAACATGTACTGATAGTCATTCGATCACGGATGCAAATGTG<br>GTGGTAGGTTTCTTAAAAGCATGGGGTTCGGTGTATAAAAACAAGGGAGATG<br>CAGAGTTGGTAGCCACTTCTTCGGTACCATTTCTTGATAGATCAGCTCTGATCA<br>AAGAAAGGGAAGTTCTTGATAGAGTACTTTCGATAGATCCACAATCAATCAAC<br>TTTAACTTGAGCCCAGCTGTAATGCTGTCGGAAAACTTGGTCCGAGCCTCCTTT

	GTGATCTGTGAAGTTGATGTTGTGAAGCTCAAGCAATCAGTGAGGTCAAAATC AGGTGTTGCAGTAACCTGTGCATATATCTGGATATGCAGGCTGAAAGCAATGA ATGATGTTGGGGAGAAAGTGGACGAGAGCGAAAACGTTCACTTCATTTTGTCT GTTGATTGCAGGACACATCTGGATCCACCAATCGCTTCAACTTATATGGGAAA CTGCGTTGCTCCCTGCATTGCAACATCAAGAATTGGCGATTTACTCCGACCCG AAGGCCTCAAGATTGCTGCAGAGTTGATTGGAGATGCCTGTGATAAAAGGGTT GCCGACAAAGAGGGATTGTTGAAAGGTGGATTTATATCTGAACTTGAAGAAG TGAACTGGAGCAGGTCATTTGGAGCTGCTGGATTGGTGACTTCTGATGTTTAT GACACTGATTTTGGATGGGGAAATAAGCTGGAAAAATTTGAATCTATTTCCAC CGATATGGATGGATCCATATCTCTTGAGAAATCTGGCAAATTTGAAGGAGGAT TTGAGATTAGTTTGTCTATGGCTAAGCACAAGATGGATGCTTTTACATCTATTT TCACAAATGGTCTGAGGGATTTGTGA
*Cp*CiS (PX842831)	>ATGGCTTCCGAAATCATAAAGCTTCCCCTCATTAATTTCTCCGTCCCGAATTT GAAGCCCGGAACCGCAAAATGGAACTCCATAAAGATACAGGTGCGACAGGCC ATGGAAGAATTTGGATGCTTTGAAGCATCATTTGACAAAATCCCAGTTGAAAT TCGAAAAGCTATTTTTGATGCTTCAACACAGCTATTTGACCTGCCAGAAGAAA CCAAATTAAGATGCTCTTCTGAAAAGACCTCCATTGGCTACCTGGGAAGAAAC CATTCTGCTTTGTGCCCCCTCTATGAATCCATGGGAATTGCGGATGCAGATTTT TCAGAACAAGTTCACAGCTTCACTCAGATCCTTTGGCCCCAAGGCAACCCAAA CTTTAGCAATGCTGTGGTATCCTACACGCAACAGCTGTCTGAACTAGATCAAG TGATAAGAAGGATGCTGGCGGAAAGCTTGGGCCTGGCGAAACATTTTGATGA GCACCAGGGAAACACCAGCTATGTTGTTTTTCTCACCAAGTACAAAGCGCCCG AGAATGACGAGCCTCATCCTGGACTGAAATCCCACAAAGATCAGAACACCTT GACCATATTGTCCCAAAACCATGTAGATGGATTACAGGTCCTGACAAAAGATG GGCAGTGGTTTGATATTAAACCCGCACCAGACTCATTTGTAGTCATTTTAGGG GAGGCCTTTGCGGCGTGGATTAATGGCCGACTGGAGCCTCCGACTCATCGGGT AGTGGTGTCCGGCACTGAAGCAAGACACACGATTGGGTTATTTGCAGGTCCAA GACCAGGATATTTAGTGACAGCACCGGAGGAGCTGGTAGATGAGGAGCACCC CATGGCTTTCAAGCCCTATGATCATGCTGAGTACGATGAATATCTTCATGTGC ATCTGGCGAATGATGATCTCGACTTGAAGACCTTTTGCGGCACTAGCATCAGT GCTTGA
*Cp*CiR (PX842832)	>ATGGCTGGGCAGAATTTAAGGGATGTCCTTCGATTAGTAACTAGAAACAGCT CATCAGAGAAAGAGATATCGGCGGATAATCGCGTAAAGACCTTCGGCTGGGC AGCTAAAGATCCCTCCGGAATCTTTTACCCCTTCCAATTCACTAGAAGGGAAA CCGGTGAGGAGGATGTAAGATTTAAAGTTCTCTACTGTGGAATCTGTCACTCC GACCTTCATGCAGCTACGGATGAGTTCAGCTTTGGTTTATACCCGGTTGTACC AGGGCATGAAATAACTGGCATAGTCACCGAGGTAGGCAGCAAGGTAACCAAA TTCAAAGTTGGAGATAAAGTTGGGGTGGGCGGCTTAGTGGGATCATGCCGCTC ATGCGACAAATGTTCCAATGATCTAGAGCCTTACTGTTCCAAGATGATCATGA CCTATTTCTCCATTGATGAAGATGGAATCCCTACCCGCGGAGGATTTTCAAAT GAGATGGTTGTGAATGAGCATTTTGCAATTCGCTGGCCGGAAAATCTCCCTCT TGATGGAGGTGCTCCATTGCTATGTGCTGGTAGCGCTGTTTATAGTCCAATGA TTTACTATGGGTTGTCCAAACCAGGAACCCATCTGGGAGTAGTAGGCCTTGGT GGATTAGGTCATGTGGCTGTAAAATTTGCTAAGGCATGGGGGCTCAAAGTGAC

	TGTGATCAGCACTTCACCTGGCAAAAAAGAGGAAGCCATCAATCATCTTGGA<br>GCTGACTCTTTTTTGGTTAGCACTGATCAAGAGCAAATGCAGGCTGCCATAGG<br>CACATTAGATGGTATTTATGACACAGTATCTGCTGTTCACCCGCTCATGCCATT<br>GTTTGGTCTACTCAAACCTGATGGAAAACTTATTGTTGTTGGTGTACCAAGTA<br>AGCCACTGGAAGTAGACATCCCTGTCCTCATTTTTGGGAGGAAAATGCTTGGG<br>ACTTCTGCAGCTGGAGGGATTAAGGAGACTCAAGAAATGATTGATTTTGCAGC<br>AAAGCGCAATATAACTGCAGATATTGAGGTTGTTTCAACGGACTACATTAACA<br>AAGCAATGGAACGTCTTGAGAAAGGAGATGTTAGGTACCGATTTGTCATCGA<br>CATTGGGAATACTCTTGTGGCTACCCCAAGCACTTGA
*Cp*CiO<br>(PX842833)	>ATGGCTACTTTCCCATTCCAAAAAGAAGCAACGTGGTTCTTGAAGCACTATT<br>ATTTAGAACTTTTTCTGCTTCTTCCATCCTACATTTTCCTTGTATTTCTTTTCAA<br>ATGGTGGTTTGGTACTTATTTTTCAAATAACAAAAAGAATCCTCCACCTTCACC<br>ACCAAAGCTCCCAATAATCGGAAACCTTCATCAGATCGGTCCTCTCGCCCACC<br>GCTCTTTCAAATCCTTGGCGGAAAAATATGGTCCAGTTATGCTGTTTCACTTAG<br>GCAGCCGACCACTTTTAGTAGTCTCCTCTGCTGAGGCAGCTCACCAAGTCCTC<br>GTAGCAAAAGATGTGGACTTCGCGGATAAATCTAATACGATGGTTGGCAGAC<br>GGCTTTACTACGAAAGCAAGGCTATAGCTTTTGCTCCTTATGGTGAATATTGG<br>AAGCAAATAAGGGGAATCAGTGTACATCTGATTCTAAGTCAAACCAGAGTTC<br>AATCATTACGAAATGTAAGAGAAGAAGAGATGGAATTACTTATAAAAAACAT<br>TAGAGAGCATATTGATTCGTCTTCGGTGATACGTATGGATAAGGTTTTGAGAG<br>CCTTTACATACGAAATTTCATCGAGGGCTGTGATTGGGAGGAGATTCTCAGAT<br>GAAGGTGGTAAAAAATTTATGGAGTTGCTTAAGGAAGCTCATGATTTGTTGGG<br>TGTTGTTAACATTGGGGACTACATCCCATGGCTTGGATGGGTTAATCGTTTCA<br>ATGGGCTGGAAGCAAAGATCAAGAAAGTAGAAAAAGAATTGGATGAATATTT<br>GGCGAATTTAATTCAAGGTGAAATTATCAGACAGAGAAGTACTGGAAGCATT<br>GATGTTGATCAACAACGTAATAATCAGAGTTTCCTCGAGCTTTTGATTGAATC<br>CCAGAAAACCAACCCGACTGCTGATTTTTTGGATGTAGACACCATGAAAGTCA<br>TCACCTTGGAAATGGTTGCAAGTATAGCAGATACATATACGGTGATTGAATGG<br>GCACTAGCAGCAGTCATACGACATCCAAATGTGATGGCAAAATTGCAAACTG<br>AGGTGAAAAAAGCAGCTGGAGAAAAATCATTCATAACGGAGGATAATTTAAA<br>CAACCTGCAATATTTGAAAGCTGTAATAAAAGAGTGTTTCAGATATAGTCCTG<br>GAAATACTTTAGTTGCACGAGCTGCGGCCAAGAATGTCAAGTTAATGGGCTAC<br>GACATTGAAGCAGGTACAGAAGTGCTTATCAATTCTTGGGCTATTGGAAAAGA<br>TCCATTATTGTGGAAAGATCCAGAGGAATTCCAGCCAGAGAGGTTCTTAAATA<br>GTTCAGTAGATCTTAAAGGTCATCATTTCGAATTCGTTCCATTTGGTTCAGGAA<br>GAAGGGGCTGCCCTGGAATTGCATTTGCTTTAGCCATAGCTGAACTTACGCTG<br>GCAAATCTCGTCTGCAACTTCAATTTTGCACTACCTAATGGATTAAGAGTAGA<br>GGAACTGGATATGACCGAAGCTCCTGGGACGGTAACTCGCATGAATACCCCTC<br>TACAAGTTGTTGCATCAGCATGTTAA
*Cp*KR4<br>(PX842840)	>ATGGAAGAAGGCAATCAAATCCAAATTCCAAGAGTGAAACTGGGTTCTCAA<br>GGTCTAGAGGTATCAAAACTGGCCTATGGATCTGCAGGACTCTCTGGTTATTA<br>CAGTGGACCTGTCTCATATGAGGAGGGATGTTTAATGATAAAGGAAGTATTCG<br>AAAGAGGTATCACTTTCTTGGACACAGCAGAAATCTATGGTGGAGATCATCAT<br>AATGAGATCATGGTCGGAAAGGCTTTAAAGAACCTTCCTCGAGAAAAGGTTC

	AATTAGCAACAAAATTTGGCATTTCAGAGAGGGATGGTCAATTGATGGTCAAC<br>GGTAATCCTGAATATGTTCGGAAGAGTTGTGAAGCTAGCCTTAAGAGGCTTGA<br>TGTGGACTACATTGATCTGTACTATCAGCACCGGATAGATGTTTCAGTGCCAA<br>TAGAAGAGACTATGGAGGAGCTCAAAAAACTTGTTGAGGAGGGAAAGATAAA<br>GTACATTGGATTGTCTGAAGCAAGCATCGATACAATAAGAAGAGCGCATGCT<br>GTTCATCCTATTACTGCTATACAGATGGAGTATTCTTTGTTGACTCGTGAAATT<br>GAGTCTGAGATTATTCCACTTTGCCGAGAGCTCGGTATTGGAATTGTGGCATA<br>CAGCCCTCTTGGTCATGGCTTCTTTGGAGGGAAGGCAACGGAGGAGAATGTGC<br>CTTCAGAGAGTATATTGAATTCAATTCCTCGGTTTAATGGAGAGAATTTAGAG<br>AGGAACAAACTTCATTATGCTCGTGTAGCAGATTTGGCGGCAAGGCATGCCTG<br>TGCACCTTCTCAATTAGCTTTGGCATGGCTGCTTCATCAGGGAGATGATATTAT<br>ACCACTTTTTGGGACAACAAAGGTTAAGAACCTTGAGAATAACATAAGATCCT<br>TGACTGTGAAGCTTACAGAAGAGGATTTGAAAGCGATTTCTGAAGCAGTGCC<br>AGCTGATGAAGTAGCTGGTGAGAGGGAAGGGCCTATCTTGTACAAACACAGT<br>TGGAAATTCGCAACTACACCTCCCAAAAATAAGAATCCACCAGCTACATAA
*Cp*SGD1<br>(PX842834)	>ATGGAGATCCATCGTTCGGATTTTCACACTGATTTCATCTTTGGAGCTGGAAC<br>TGCTGCTTATCAGATTGAAGGTGCTGTAGCTGAAGGAGGTCGAGGCCCTAGTT<br>CTTGGGATACTCTTATCCAAAGGACACCAGGGAAGGTCAATGAGGGTCAAAA<br>TGCAAATATTGCATGTAACTCGTATTATCTTTACAAGGAAGATGTGAAATTAG<br>CGAAGCATATTGGTTTAGACTCCTACAGATTCTCAATCTCATGGACAAGAGTA<br>TTGCCTGGTGGTAGATTAAATGCTGGTGTCAACAAAGAAGGGATCCGGTACTA<br>CAACGATCTCATTAACGAGCTCCTAGCAAATGGCATCGAACCATTTGTGACCT<br>TGCACCACTTTGAAGTTCCTCAACCACTAGAAGAAGAGTACGGTGGTTTTTTG<br>AGTGACCGAATTGTGAAGGACTTTTGTGAATTTGCGGAATTGTGCTTCTGGGA<br>ATTTGGTGATCGTGTGAAACATTGGACGACATTCAATGAGCCATGGAGTTTTA<br>TATATTATGGATATGTTGCTGGCTCAATGCCCCCTTGTCGAGGTTCATCATCAG<br>CAGAACACGCAGAACACCCTCTTATTCGACATAGATGTCATCATCACTGCGCA<br>TTCATTTGTGAAAAGGGAGATCCCGGCGTAGAGCCATACATGGCATCACGCCA<br>TTTGCTCCTTGCTCATGCAGAAGCTGTTCAAATTTATAGAAATAAATTCAAGG<br>TTCAAGGGGGAAAAATTGGACTTACACTTGTGACCCATTGGTATGAGCCACTC<br>ACCCAATCAGTGAGCGATATAGAGGCAGCTCAACGAGCAATGGATTTTGAAT<br>TTGGCTGGTTTATGGATCCAGTAACCTATGGTCAGTATCCACTGAGTATGATT<br>GAACTCGTTCCACCAAACCGTCTTCAACGATTTTCACCAGAAGAATCTGAAAA<br>ATTGAAAGGATCATATGATTTCCTTGGGCTCAACTATTACACATCTCTGTATGC<br>AACTGATTATTCGGGTCGCAATTATGGACCTCCTAGTTACATAACAGATTCGC<br>AAACCAAGACTCAATGTACGGCTGCTGATGGAAAGACATTGATTGGTCCAGA<br>AACTGATTCTGGCTGGATATACATTTGTCCTAAAGGAATATACAAGCTTTTGT<br>GTTATATAAAGAAGCAGTACAACGATCCACCAATCTATATCACAGAGAATGG<br>TGTCGCTGAATCGGAAGATTTCAATAAGACGGTTTGTGAGGCTCGTGCAGACG<br>AGACCAGGATTAACTATCATCGTGAGCATTTAAAAGAAATAAAACATGCTAT<br>GGTTGAAAATCGGGTTAATGTGAAAGGTTATTTTATATGGTCATTGATTGACA<br>ATTTTGAGTGGACATCAGGTTACAAAGTTCGCTTTGGCCTTGTATATGTCAACT<br>TTAAAGAGGATCAGCTGTCGAGGTACCCGAAAGACTCGGCTCTATGGTTTATG<br>AATTTTCTTGATAAGAAGAACAAACCTGTTGAATATCCACCACGAGGAGGTTT

	ACTTGCCAATCATGCCATGGAGATTACTACCATTCCACCACCTTCCTTTCAGAA ATTAGCAACAAATTCTGTGGAAATTGTTGTCCGCGAGAGTCCTATTAAGAGGC TTCGTGCAAATTGA
*Cp*SGD2 (PX842835)	>ATGGTGGAGTTGACAATGCTCAAACTACGGCTTGGAATCATTTTACTGTTCAT CACCTTTTTCTCCAATGCAGCAGGAGCATTTATTGGTATTAACATTGGCACTGA CCTTTCAAACCTACCATCAGCAACGGATGTGGTTGCAATCCTAAAAGCTCATC AAATAACTCATGTCCGCCTGTTTGATGCCGATGCTCACATGCTGACCGCTCTTT CAGATACTGGTATTGAAGTCATGGTCAGTGTCACAAATGAGGAGATTCTAAGG ATTGGTCAATCACCATCTGTTGCAGCAGCCTGGATTAATAAAAATGTTGCAGC TTTCTTGCCTTCAACTAATATTACAGCTATTGCTGTTGGCAGTGAAGTTCTCAC TAATATTCCTAATGCTGCACCTATTTTGGTTCCTGCCATGAATTACCTCCACAA AGCCCTGGTTGCTTCTAACATAAATGATCAGGTTAAAGTTTCAACCCCACAAT CAATGGATGTGATCCCTAGGCCCTTTCCTCCATCCACTGCAGCATTCAATGCC ACATGGAACTCCACAATTTTTCAGATGCTTCAGTTTTTAAAAAACACTAATTCC TTTTACATGTTAAATGCTTATCCTTATTATGGATATGTTGATAGTGATGGCATT TTTCCAATAGAATATGCTCTTTTCCGACCACTCTCCTCAGTCAAACAAATTGTT GATCCAAACACCCTTTTTCACTATGACAGTATGTTTGATGCTATGGTGGATGCT ACCTATAACTCCATTGCAGCTTTCAATCTTTCAGGCATCCCAATTGTGGTGACA GAAACAGGGTGGCCATGGTTTGGCGGCACCAACGAACAAGATGCTTCTATAG AAAATGCTGAAACTTTTAATAATAATTTAATCCGGCGTGTATCAAATGATTCT GGTCCTCCTAGTCAGCCTGACATTCCCATAAATGCATACATCTATGAATTGTTC AACGAGGACAAGAGAGGTGGGCCTGTTTCAGAAAGGAACTGGGGAATCATTT TTACCAATGGAAGCACTGTCTATCCAATTAGTTTAAGTAATTCAGATAGTGTT AGTAAAAATTCTTCAGGTGTGTTTTGCGTAGCAAGAAAAGATGTTGACGATAA AAAGCTGCAAAGTGGACTTAATTGGGCTTGTGGACAAGGCCAGGCTAATTGC ACAGCCATTCAAACAGGGCAGCCTTGTTATTTTCCTGATACACTGCAAAATCA TGCTTCTTATGCTTATAATGATTATTATCAGAAAATGCATTCCATTGGTGGAAC CTGTGACTTTGATGATACAGCAATGACTACTTCTCAAGATCCCAGTTACGGAT CCTGTAAATTTACTGGAAGGTCAATTTCAAGTACTGGTGGTTTATTTCCCTCTC CAGCATTTGGACCTGTAGGTCCATCATCACAAGGTCCAATAGTTAGAGCGTTT AGTGTTGTCTTTGTGTTGGTTATTGTTTTGTCTTTGGTCATTGTTGATGTTAATA TCTACAACACTTTGTAG
*Cp*SGD3 (PX842836)	>ATGGCTCTAATTATGAACTTTTGCATACCAAGTATGAATGCATCAAGGGGCC GTCCTTCAGAAAACCCATATGTTCATTTGAGTTCTTTGGATAAAGAAAGCCGC ATTCTGAAAAGAAATGTGGAATTAGTTTCTTCATCTCGTGCAAGGGACAACTC CTATTCCAGAAGCGTAACAAAATGTCTCAAGGGGGTTGCTTTTCCGGGTCCTG ATGTACTTCAAAATCTCGTAAATGGAACAATCTTCCCAGATCCACTCATCCCT CAAACTCAACAAATCTCGAGGAAAGATTTTCCTACTGATTTTAAGTTTGGCTG CTCAACTTCTGCTCTTCAGACTGAGAGCTCACCAACTGAAGGTGGGAGAGGAC CGAGTACGTGGGATTTTATGATTGGCGACAACCAAATAGCAGTTGATTCCTAC CATAGATACAAGGAAGATGTGCAACTGCTCAAGAAAATGGGGGCCGACACTT ACAGATTCTCCATTTCTTGGTCGAGGATCTTACCTGATGGGACAGTGAGGGGA GGAATAAATCAAGAGGGCATCGACTTCTACAACAATTTAATTAATGAATTGAT TAAAAACGGGATTACTCCATTTGTGACGCTTTTCCATTTTGACTTACCAACGGC

	ATTGCAAAACAAATACAACGGATTCTTGGACAGCCGGATCGCGGATGATTTTA<br>AAGCATACGCAGATATTTGCTTCAAAAATTTCGGCGATCGAGTAAAATATTGG<br>TTAACAATTAATGAACCACAAGTGTTTGCTCAGTATGGATATGTTCAAGGACT<br>AAAAATACCGGATGATCGAGTCAAGTATCCTTTCCTTGCTATGCACAATATCA<br>TATTAACTCATGCTACTGCTTTTAAACACTACAAGGAGAATTATCAGGCAACC<br>CAAAAGGGAGAGATTGGAATGTCATTGGTAACAGAGTGGTTTTTGCCGAGTG<br>ATGAATCGAGACCAAACAGAGCTGCAGCAGAGAGGGCGTTCGACTTTTTAAC<br>CGGATGGATATTGGAGCCACTGGTATATGGTGATTATCCATTCATCATGAAGG<br>CTTTGGTGAGAGATGTGCTTCCAACATTCACAGACGCGCAAAAGAAATTAATT<br>AAAGGATCGCACGATTTCATTGGCGTCAACTATTATACTTCAAGATATGCAGC<br>TGATATTCCAATTGTGCCGAATGAAACCTACACCAGTTACGATCAGTACCAAC<br>ACGTTCTGGAGAACGTTGACAATAACGGAAAGCCCATAGGATTACAGTCCCC<br>AGGAAACACTGCTATCTATGTGTACCCTCAAGGTTTGGGAGATATTCTGGAGT<br>ACATAAACAGAGAATATGGCCAGCCCAAGATATACATCACTGAGAATGGCTA<br>CCCGGAGAAAAGAGACGATTCGATTCCAGTGGAAACTGCACTCAAGGACGAT<br>GCTAGAATTCAACATATTATTACTCATCTCCAGTCAGTTTCAAGAGCCCTCAA<br>AAATGGAGCAAACGTTAAAGGTTATTTCATGTGGGCACTGATGGATTGCATGG<br>AAGTGGGCTCGAAGTATGCTGTCCGTTACGGTCTCAACTACACGGATTACCTC<br>AACAATTTGAACCGTATTCCAAAGAAGTCTTCCCAATGGTTCCAATCTACTTTC<br>TCGAAAGGCTCCAACTGA
*Cp*KR1<br>(PX842837)	>ATGTCTGACCAAGTTGAGATGCGAAGAGTGAAATTGGGAAGCCAAGGACTT<br>GAGGTTTCAAGATTAGGTTTTGGTTGTATGGGTCTTAGTGGAGTTTACAATGCT<br>CCAATACCTGAGGAGGATGGAATTGCAATACTGAAGGAGGCTTTTAGCAAAG<br>GAGTTACGTTTTGGGATACATCAGATATATATGGCATAGAGCATGCTAATGAA<br>TACTTGGTTGGGAAGGCACTAAAGCAACTGCCTCGTGAAAAGGTCCAGTTAGC<br>CACAAAGTTTGGTGTGTTTAACATTGAGCCTACAAAAGTCACAGTAAAAGGCA<br>CTCCTGAATATGTTCGCTCTTGCTGTGAGGGTAGCTTGAAGTGCCTTCAAGTG<br>GACTACATAGATCTGTATTATATACATCGAGTAGACACTTCAGTACCTATTGA<br>AGAAACTATGGGTGAGCTTAAAAAATTAGTTGAAGAAGGTAAAATTAAGTAC<br>ATTGGTCTATCTGAAGCTAGCCCAGATACAATAAGGAGGGCACATGCTGTTCA<br>TCCTATCACTGCTATACAACAGGAATATTCCCTATGGACTCGTGACATTGAAG<br>CAGAGTTGCTACCTCTTTGCAGGGAACTTGGAATCGGGATAGTTCCATATAGC<br>CCTGTTGGTCGTGGTCTTTTTGCTGGAAAGGCTGTTGTTGAAAGCTTGCCTCAG<br>AATAGTAGCTTGGAGTATCATCCAAGGTTTACAGGAGAGAACTTTGAAAAGA<br>ACAAGGTCATCTTTTTCCGCTTAGATAAGTTAGCTAAAAAGCATGGATGCACT<br>CCTGCCCAACTTGCAATTGCTTGGGTTCTTCATCAGGGTGATGACATTGTACCC<br>ATCCCTGGAACAACTAAGATAAAAAATCTTTACGACAATGTTGGTTCTCTGAA<br>AGTCAAGCTCGCTGAGGAGGATTTGAAAGAGCTTTCTGATGCAGTCCCAGTCA<br>ATGATGTTGCAGGTCAGAGGATTGGAGATGCTCTTTTCCGTACTTCATTTTACT<br>TTGCTAATACACCACCACTCAAGAAGTGA
*Cp*KR2<br>(PX842838)	>ATGGCCACTGGGATAAAGTACTTCGTGTTGAACACCGGAGCAAAGATGCCGT<br>CGGTTGGTCTAGGGACCTGGCAGTCGGAACCTGGTCTCGTCGCTCAAGGGGTC<br>GATGTTGCTATTAGGCATGGGTATCGCCATGTTGATTGTGCTGCCTCTTACTCC<br>AATGAGAAAGAGATTGGCGATATTTTGAAGAAACTGTTTCAGGAGGGTGTTGT

	GAAGCGTGAGGATTTGTGGATTACTTCGAAACTCTGGCATTCAGAACATGCAC<br>CTGAAGATGTACCCGTGGCATTTGAGAAAACCCTGCAAAACTTGCAGCTTGAT<br>TATCTTGACTTATACCTTATCCACTGGCCTGTCCGCTTGAAGAAGGGGCTTCCT<br>AGAGGGGCACCTGGTAGTGTTATGCCAACAGATATACCTAGCACGTGGAAAG<br>CAATGGAAGCACTTTATGATTCTGGCAAGGCTCGAGCTATTGGAGTTTGCAAT<br>TTCTCATCAAAGAAGCTGGGAGATTTGTTGGATATTGCACGGGTTCCCCCTGC<br>TGTTGACCAGGTAGAGTGCCACCCTGTATGGCAGCAGCACAAGTTGCGGGAA<br>TTCTGTAAATCTAAGGGTGTTCACCTATCTGCATGGTCACCTTTAGGTTCACCT<br>GGGACATCATGGATGAAAGGAAATGTCCTCACACATCCAGTTATTACCAGTAT<br>TGCAGAGAAATTGGGGAAAACTCCTGCACAGGTTTCTTTACGCTGGGGTTTGC<br>AAGTTGGTAATAGTGTCCTTCCCAAGAGTACAAGAGAAGAAAGAATTAAGGA<br>GAATTTTGACATCTTTGACTGGTCCATACCTGAAGACTTGCTTGCCAAGTTCTC<br>TGAAATTAAGCAGGATCGATTAGTGAAAGGGGAATTCTATGTTTCCGAAAATG<br>GACCATACAAGTCAGTTGAGGAAATCTGGGATGGTGAGGTCTAA
*Cp*KR3<br>(PX842839)	>ATGGCAACATCAGTGAGGAAGATCAAACTGGGGTCCCAAGGCCTAGAAGTC<br>TCAGTTCAAGGACTTGGATGCATGGGCATGTCTGCTTTCTATGGCCCTCCTAA<br>GCCTGAATCTGAGATGATCCCACTCATCCACCAAGTTATTGATTGTGGCATCA<br>CCCATCTCGACACTTCTGACATCTATGGTCCTCATACCAATGAAATTCTCGTTG<br>GAAAGGCGTTGAAGGGAGGTTACAGAGAGAAAGTGCAATTGGCTACCAAGTT<br>TGGGGCAAAACCATCAGGAGGCATCTGTGGTGAACCAGCATATGTGAGGGAA<br>GCATGTGAGGCTAGTTTGAAGCGCTTGGGTGTTGATTATATTGACCTCTATTAT<br>GTTCATCGTATCGACACTAGTGTGCCTATTGAAGTCACAGTGGGAGAACTGAA<br>AAAGCTGGTTGAAGAGGGTAAAATAAAATACATAGGCCTCTCTGAGGCCTCA<br>CCTGACACTATCAGGAGGGCGCATGCAGTTCATCCAATAACAGCAGTTCAGCT<br>AGAATGGTCTTTGTGGACACGAGATGCTGAAGAGAGTGTTATTCCTACTTGCA<br>GGGAGCTTGGGATCGGGATTGTATCTTTCAGTCCCCTAGGAGGCGGATTCTTT<br>GCTTCTGGTCCAAAATTGGTTGAGAATCTGACCAGTGATGACTTCCGAAAGTA<br>TAACCCGAGAATTCAGCCAGAAAATGTTGAGCACAACCTGCAATTGTTTAACC<br>GGGTAAATGAAGTAGCAGCTAGGAAGGGGTGTATGACATCACAGCTAGCATT<br>GGCATGGGTTCATCACCAGGGTGATGATGTGTGTCCAATTCCAGGAACCACCA<br>AGTTGGAAAACCTCAAAAACAACATCAAGGCTGTATCTGTTAAGTTAACCCCA<br>GAGGAAATGGATGAACTAGAGTCCATTGCTATTGCAATTAAGGGTGAAAGAT<br>TGCCATCTGGAGCCATGGCTCATACATGGAGACATGCCAATACTCCGCCCTTG<br>TCCTCGTGGAAAGCTACATAA
*Cp*KR5<br>(PX842841)	>ATGGCATCAGAAGAGGTGAAAGTGCCAAGAATCAAGTTGGGCTCACAAGGC<br>CTTGAAGTTTCAGCTCAAGGGCTGGGCTGTATGGGTATGTCAGCCTTCTACGG<br>CCCGCCCAAACCCGAGCCCGACATGATCAAACTCATCCACTATGCTATTGACA<br>GAGGGATCACCCATCTCGACACCGCCGAGGGGTATGGGCCTTACACCAACGA<br>ACTACTCCTCGGCAAGGCTTTGAATGGAGGGATAAGAGAGAAAGTGGAGTTA<br>GCAACTAAATTTGGAGCCAAATTAGTGGATGGGAAACACGAGATTTGTGGTG<br>ATCCGGCTTATGTACGGGCTGCATGTGAGGGCAGCTTGAAGCGGCTTGGTGTT<br>GACTGCATTGATCTGTACCATCAACATCGCATTGATACCCGCCTGCCCATTGA<br>AGTCACGATTGGAGAACTTAAGAAGCTGGCTGAAGAGGGTAAGATAAAGTAT<br>ATAGGTCTATCTGAGGCCTCCGCTCCAACAATCAGAAGAGCACATGCTGTTCA

	TCCAATAACAGCTATACAGTTGGAGTGGTCATTGTGGTCCAGAGATGTGGAGG AAGAGATAATTCCCACGTGCAGAGAACTTGGGATAGGGATTGTGGCATACAG TCCACTTGGACGAGGATTCTTCTCATCAGGTCCAAAGCTGATGGAGAATTTGA CTGAAGGTGACTTCCGAAAGTACTTGCCGAGACTACAACCAGAGAATATGGA GCATAACAAGAGCTTGTATGAGCGGGTCAATGACATCGCTTCAAGGAAAGGT TGTACCCCATCACAGCTAGCATTGGCCTGGGTCCATCACCAGGGAAATGATGT TTGTCCCATACCTGGCACTACCAAGATTGAGAACCTCAAAGAGAATATAGGA GCGTTGTCTGTAAAATTGTCAGCAGAAGATATGTCTGAGCTTGAATCCATTGC TTCAACTGGAGTTAAGGGTGACAGATACGGGCCTGAGATTCCTACTTGGCAGA CTTCTAATACTCCACCTTTGTCAACATGGAAAGGTATATGA
*Cp*KR6 (PX842842)	>ATGGCAACAGTGGTGAGGAAGATAAAGCTAGGATCCCAAGGCCTAGAAGTC TCGGCTCAAGGACTTGGATGCATGGGCATGTCTGCTTTCTATGGCAATCCTAA GCCTGAATCTGAGATGATCCCTCTCATCCACCACGCTATTAACAGTGGCATCA CTCATCTCGACACTTCTAACGGCTATGGTCCTTACACCAATGAAATTCTCATTG GAAAGGCTTTGAAGGGAGGGTACAGAGAGAAGGTGCAACTAGCCACCAAGTT TGGGCACGTAATACGTGAAGATGGGAGCAGGGCAGGCGTCTGTGGTGAACCA GCATTCGTGAGAGCATCATGTGAGGCTAGTTTGAAGCGTCTGGATGTTGATTA CATTGATCTCTACTATGTTCATCGCATCGACACTCGTGTTCCTATTGAAGTCAC AGTTGGGGAACTGAAGAAGCTGGTTGAGGAGGGTAAAATAAAATACATTGGC CTATCAGAGGCCTCACCTGAAACTATCAGAAGGGCACATGCAGTTCACCCAAT AACAGCAGTTCAGCTGGAGTGGTCTTTGTGGACAAGAGATGCTGAAGAAAGT GTTATTCCTACTTGCAGGGAACTTGGTATTGGGATTGTTCCTTTCAGTCCCCTT GGGAGCGGATTCTTTGCATCTGGTCCAAAATTGGTTGAGAACCTGAACCAGAG CAGTAATGACTTCCGAAAGTACAGCCCAAGATTTCAGCCAGAAAATGTTGAG CACAATCTGAAATTGTTTGAGCGCGTTAACGAAATTGCCTCAAGGAAGGGGTG TACACCATCACAGCTAGCATTGGCATGGGTTCATCACCAAGGGGATGATGTCT CTCCGATTCCAGGAACCACCAAGATAGAAAACCTCAACAACAACATTAAGGC TTTATCTGTTAAGTTAACACCAGAGGAAATGGCTGAACTTGAGTCCATTGCTA ATGAAATTAAGGGTGAAAGATTGCCAGCTGCTGTCTTAGCTCTTACCTGGAGA CATGCCAATACTCCGCCATTGTCCTCGTGGAAAGCTACTTAA
*Cp*KR7 (PX842843)	>ATGGCGAGTCTACCAAAAGTGCCGAGGATGAAGCTGGGCAAACAGGGCCTT GAGGTATCAAAACAAGGTCTGGGCTGTATGGGTATGTCCTCCAATTACGGCCC GCCCAAGCCCGAGCCCGATATGATCGAACTCATCCGTCACGCTATTAACTCCG GAGTCACATTTCTTGATACCTCCGACGTCTATGGACCCCACACCAACGAACTC CTCATCGGCAAGGCTTTGAAAGGAGGGCTGAGAGAGAAAGTGCAAATAGCTA CAAAGTTTGGGATTAGTTGGACGGATGGAAAAATGACAGTTCGTGGAGACCC TGAACACATCCGAGCTTGCTGTGAGGCCACCTTGAAGCGGCTTGATGTCGATT ACATTGATCTTTATTATGCTCATCGCATTGATACACGAGTTCCCATTGAAGTAA CGATTGGTGAGCTCAAGAAACTTGTTGAAGAGGGTAAAGTAAAACACATTGG TCTATCGGAGGCCAATGCTTCAACAATTAGAAGGGCTCACGCAATACATCCGT TAACAGCTATTCAAGCTGAATGGTCTTTGTGGTCAAGAGATTTGGAGGAAGAG ATTATTCCAACATGCAGAGAGCTTGGCATCGCCATTGTACCTTATAGCCCTCTT GGACGTGGATTCTTTTCGGCAGGACCCAAAATGTTTGAGAACTTGTCTGAGGG TGATTCACGCAAGAATTTCCCAAGGCTGAAGGCAGAAAACCTTGATCAGAAC

	AAGCAAATATTTGAAAGGGTTAGTGAAATGGCAGCAAAGATGGGATGCAGCA<br>CAGCACAGCTTGCACTGGCTTGGGTACATCATCAGGGAGATGATGTATGTCCG<br>ATTCCTGGTACTACCAAGTTGGAGAACTTCAACCAGAATGTTGGAGCTTTGTC<br>AGTGAAACTATCACCAGAGGAGATGACTGAACTCGAATCCTACGCTTCCTTTG<br>ATGTGGTTAAGGGTGACAGACATGCCTACATGGGGAATACATGGATTAACTCC<br>AATACCCCTCCTTTGTCTTCCTGGAAAGCTGAGTAA
*Cp*KR8<br>(PX842844)	>ATGGCAACCGAAGAGGTGAAAGTGCCGAGAATCAAGTTGGGCTCACAAGGC<br>CTTGAAGTTTCAGCTCAAGGGCTGGGCTGTATGGGTATGTCAGTCTTCTACGG<br>CCCGCCCAAACCCGAGCCCGACATGATCAAACTCATCCACTATGCTATTGACA<br>GAGGGATCACCCATCTCGACACCGCCGATATATATGGGCCTTACACCAACGAA<br>CTACTCCTCGGCAAGGCTTTGAATGGAGGGATAAGAGAGAAAGTGGAGTTAG<br>CAACTAAATTTGGAGCCAAATTAGTGGATGGGAAACACGAGATTTGTGGTGA<br>TCCGGCTTATGTACGGGCTGCATGTGAGGGCAGCTTGAAGCGGCTTGGTGTTG<br>ACTGCATTGATCTGTACCATCAACATCGCATTGATACCCGCCTGCCCATTGAA<br>GTCACGATTGGAGAACTTAAGAAACTGGCTGAAGCGGGTAAGATAAAGTATA<br>TAGGTCTATCTGAGGCCTCCGCTCCAACAATCAGAAGAGCACATGCTGTTCAT<br>CCAATAACAGCTATACAGTTGGAGTGGTCATTGTGGTCCAGAGATGTGGAGG<br>AAGAGATAATTCCCACGTGCAGAGAACTTGGGATAGGGATTGTGGCATACAG<br>TCCACTTGGACGAGGATTCTTCTCATCAGGTCCAAAGCTGATGGAGAATTTGA<br>CTGAAGGTGACTTCCGAAAGTACTTGCCGAGACTACAACCAGAGAATATGGA<br>GCATAACAAGAGCTTGTATGAGCGGGTCAATGACATCGCTTCAAGGAAAGGT<br>TGTACCCCGTCACAGCTAGCATTGGCCTGGGTCCATCACCAGGGAAATGATGT<br>TTGTCCCATACCTGGCACTACCAAGATTGAGAACCTCAAAGAGAATATAGGA<br>GCGTTGTCTGTAAAATTGTCAGCAGAAGATATGTCTGAGCTTGAATCCATTGC<br>TTCAACTGGAGTTAAGGGTGACAGATACGGGCCTGAGATTCCTACTTGGCAGA<br>CTTCTAATACTCCACCTTTGTCAACATGGAAAGGTATATGA
*Cp*T5H<br>(PQ568389)	>ATGGAGAATTTTACAGTTGAAATCCTCTGCATGTTTTTCTCTCTTTTGCCACC<br>TTTTGTTACTACTATTGTTGCAAAATTTATGCACGGACTCGGTCACAGCCTGTG<br>ACTCTACCACCGTCACCCTCAAAACTCCCCATCATCGGTCACCTACACCTCCTC<br>ACCGACATGCCCCACGTTGACATGGCTCAACTCGCTGAAAAACTCGGCCCAAT<br>AATCTACCTCCAACTCGGTCAAGTCCCCACTGTGGTTATCTCGTCGGCTAAACT<br>CGCCGAACTCGTTCTCAAAACTCACGATCACATAATGGCGAATCGGCCCCAAC<br>TCATTGCCGCTCAGTACCTCTCATTCGGCTGCTCCGACGTCACTTTCTCCCCTT<br>ACGGCCCTTACTGGCGGCAAGCGAGAAAGGTCTGCGTCACCGAGTTACTGAA<br>TTCGAAACGAGTCAACTCGTTCCAACTCGTTCGAGATGAGGAAGTGAATCGCA<br>CGTTACGCACCGTGTCCGAGTCAGCTCATACAAACTCTGAAATCGACGTGAGC<br>GAGTTGTTCTTCAAACTCGCCAACGACATCCTCTGCCGCGTAGCATTCGGGAA<br>GAGGTTTATGGACGAGACGAGTCATGAAGGAGGGAAGAGGAACGAGTTGGTC<br>CGAGTCTTGACGGAAACGCAGGCTTTGCTAGCTGGGTTTTGCATCGGAGACTT<br>TTTTCCGAGTTGGAAGTGGGTTAATTCAGTGAGTGGGATGAAGAGAAGGTTGA<br>TGAATAATTTGAAGGATTTGAGAATGGTTTGTGATGAAATAATTAATGAGCAT<br>TTGAGAAAGACAGAGCATACAGGTTCAAGCGGCGTCGTTTCGGAGAAAGAAG<br>ATTTTGTGGATGTTTTGCTCAGAGTTCAAAAGCAAGATGATCTTGAAGTTCCT<br>ATTACTGATGATAATCTCAAAGCTCTTGTTATGGACATGTTTGTAGCTGGGAC

	AGATACAACATCAGCAACATTAGAGTGGACAATGACAGAACTGGCAAGGCAT<br>CCTAATATAATGAAAAGAGCACAAGAAGAAGTAAGGCAAATTGCAGCAAGCA<br>AAGGAAGAGTAGAAGAAACTGACCTTCAGCATCTTCATTACTTGAGAGCTGTG<br>ATCAAAGAAACCATGAGACTTCATCCACCAGTCCCTCTTCTCGTTCCACGCGA<br>ATCTCTTGCCAAATGTACGATTGATAAGTATGAAATACCAGCGAATACTCGGG<br>TTTTGATCAACACTTACGCTATTGGGAGGGATGCTGATTCATGGGAGAACCCT<br>TTGGAATACAATCCTGAAAGGTTTGTAGGGAAAGATGGTATTGATTTTAAGGG<br>TCAAGATTTCAGGTTTTTGCCATTTGGAGGTGGAAGAAGAGGTTGCCCTGGTT<br>TCTCCTTTGGGTTGGCAAGTGTTGAGATTTCGCTAGCCCGTTTACTGTATCATT<br>TTGACTGGAAATTGCCTCAAGGGGTTGGAGCAGATGATGTTGATCTTACAGAG<br>ATTTTCGGACTTGCTACTAGGAAAAGATCAGCACTAAAATTGGTTCCTACAAT<br>GATCAAGAATTAA
*Cp*OMT1<br>(MW456557)	>ATGGCAACATCTGAGAATTCTGCTGAGCTTATGAGAGCTCACAATCTTATTT<br>GGAACCAAACATTCAACATCAAGAATTCAGCCTGTCTAAAATGTGCAATTCAA<br>CTAGGCATACCAGACGTCATCCACAAGCATGGAAAGCCCATCGCTCTCGCTGA<br>CCTGACTTCTGCCCTTCCAATTAACCCTTCTAAAGCTCCGTACATCAAACGCTT<br>AATGCAAATTCTAAAAGATGCTGGGTTTTTTGCTCAAGAAAAAGAGGGTTATT<br>ATGCTCTTACTTTTGCGGGCCGCCTTCTTGTCGAAAATGAGCCAATGAATGGA<br>AGAGAGTTCGTTCTTATGAATCTTGATGCTGCTATGATGAAGCCTTGGATTGT<br>GTTGAGTGAGTGGTTTCAGAATGATGATCATGCTCCATTTGACACTGCTCATG<br>GGAAGAATTTTTGGGATCATAATGCCGATGAACCAAAAGTTGGCAAACTATTT<br>AATGAAGCTATGGCTAGTGATTCTCAGATGGTTACGACGGTGCTGACTAAAGA<br>ATGTGGATATGTGTTTGAAGGGTTGACATCTTTGGTGGATGTTGGTGGTGGCA<br>CAGGCACAGTTGCTAGGTCCATTGCTAAAATGTTCCCGAATTTAAAATGCGCT<br>GTGTTTGATCTCCCACATGTGATTGCCAATCAAGAAGGAACTCAGAACTTGGA<br>TTTTGTTGCAGGAGATATGTTCGAGAAGGTGCCCCCAGCTAATGCAATCTTAC<br>TTAAGTGGATTCTCCATGACTGGGGTGATGAGGAATGCATAAAGATTCTCAAG<br>AACTGCAAAAAGGCAATTCCAGGAAGAGACGAAGGGGGAAAATTGATCATCA<br>TAGAGATGGTTATGGAAAGCCAGATAGAGGATGAGGATTCAGTTGAAGAGCA<br>AATTTGCTCGGACTTGCAAATGTTTGTTTTATTCCGTACTAAAGAAAGAACAG<br>AGAAAGAATGGGCAACACTTTTCTGGAATGCTGGATTCAGCGACTATAAAGT<br>ATTTCCAGTATTGGGTGCAAGGAGTATCATTGAGGTTTATCCTTAA
*Cp*STR<br>(PQ568387)	>ATGCACATTTCTGAAAATATGTTCGTCGTCACCATTTCCTTCATCCTTTTCTTG<br>TCATCTCCTTCACTCGTTCTCTCTTCTCCATATTTCCAATTTATTCAAGCACCAT<br>CCTACGGCCCCAACGCCTATGCTTTTGATTCAGCTGGTGGACTCTATGCTGTCG<br>TAGAGGATGGTAGAATTGTGAAGTATGAAGGATCAAGCAATGCATTCTTGGA<br>CCATGCTGTTGCCTCTCCATTCTGGACTAAAAAACTGTGTGAGAACAACACTA<br>AACCTCAGCTAAAACCCCTGTGTGGGAGGGCATATGACCTCGGATTCCACTAT<br>GAAACTCAGCAATTATACATTGCTGATTGCTATTTTGGTCTTGGTGTTGTTGGA<br>CCTGAAGGAGGGCTTGCAAAAAAGCTTGCCAAAAGTGGAGATGGTGTGGAAT<br>TCAAGTGGCTTTATGCCTTGGTTGTGGACCAGCAAACTGGCTTTGTTTACGTCA<br>CCGATGTTAGCACAAAATATGATGACAGGGGTGTTCAAGATATCCTAAGGAC<br>AAATGATACAACAGGAAGATTAATCAAATATGATCCCACAACCAGAGAAGTT<br>ACAGTTTTGATGAAAGGCCTAAATGTACCAGGTGGTGCGGAAATTAGCAAAG

	ATGGCTCTTTTATTCTTATAGGTGAATTCTTAAGCAACCAAATTCTCAAGTATT<br>GGCTAAAGGGTCCCAAAGCAAATACTTTAGAATTCTTGTTACATGTTAAGGGT<br>CCAGGTAGTATTAGGCGGACTAAGGCTGGAGATTTTTGGGTGGCTTCAAGTGA<br>TAATAATGGAATTACGGTTACTCCTAGAGGAATAAGGTTTGATGAATCTGGCA<br>ACATTTTGGAAGTTGTGCCTATTCCTCTACCATACAAAGGTGAACATATTGAA<br>CAAGTTCAAGAACACAATGGTGCACTCTACATTGGATCTTTGTTCCATGGTTTC<br>ATAGGTATATTGTACAATTACAAGGGTTTATCAGAGGAAAATAATCTAGGTGG<br>GGTCGTTGAATCATTGAAAGGAGAGTCGTTTTCTTTCTGA
*Cp*STTr<br>(PQ568386)	>ATGGAAGCTGCAGTGATGTCTTCTTTGGACGACGACGCTGAAACTCAACTGT<br>TACAACAACCCAGTCCCAAGACTAAGAAAGGCGGTTGGATCACCTTCCCATTT<br>CTTATAGCAACCAGGGCTGGCATGACGGTTGCAGCATTAGGATGGAGCGCCA<br>ATCTTATTGTCTACCTCATTGACAAGTACAATATCGAGAGTATTGATGCTGCA<br>CAAATCTTCAATGTAGTCAATGGCTGCATGGCACTTTTTCCTATTATCGTAGCT<br>ATAATTGCAGATAGTTTTCTTGGCTGCTTTGCTGTCATCTGGATTTCTTCAATC<br>ATCTCTTTGCTGGGAATGGTTCTGTTGACTCTAACTGCAACGATTAGTTCACTA<br>AGACCTGCACCATGTAATGAAGGGTCGAGCTTTTGCACAACTCCATCACCATT<br>GGAATATACAAACCTATTTTTGGCTGTGGCTCTGGCATCTATAGGCTGTGCAG<br>GTACTAGTTTCACAGTCGGAACAATGGGAGCAGATCAACTGGATAATCCTGA<br>GCATCAAGAGAATTTCTTTAACTGGTTTCTCTTTGTTTGGAATGCTGCTTCAAT<br>AATTAGTGCTACTGTGATTGTCTATGTTCAAGATAATGTGAGTTGGGGACTGG<br>GGTATGGACTATGTGCTGCAGCAAATTTGTTAGGATTAATTAGTTTTTTGCTGG<br>GAAAGCATTACTATCGCTATGTTCAGCCACAAGGGAGTCCATTCAAGGATATA<br>GCTCGTGTACTATTTGCGGCCTTCTCTAAGAGGAAGGTCTTTTTGTCAACAAG<br>AAATGAAGATTATTTTAGTGAATTACATCTTGAAGTCGATGGCCAACATGATG<br>GTATCAAAGAATTGGCAGCATCAGCAACACCACATAAAGAAACATTCAAGTT<br>TCTGAACCATGCGGCCTTAATAAGCCAAGCAGACATCCAATCAGACGGATCA<br>ATCAGGCAATCGTGGAAGTTGTGCACAGTACAACAAGTGGAAGATCTTAAAA<br>CCTTACTTAGAATTGCCCCAATTTGGGCAACCGGTATTTTCTTAACCACACCAA<br>TGGGTATGCTATCTACCTTAACAGTCCTTCAGGCTCTAACAATGGACACTTGT<br>ATTGTATCCAATTTCAAATTCCCGGTAGGTTCCCTGGTAGTCTTTTCACTACTT<br>TCTGGTGCCATCTCTCTCACCATAGTAGACCGATTGATATTCCCTTTATGGCAA<br>AAAACATTTGGAAAAACTCCAACACCCCTCCAACGACTAGGCACGGGTCATG<br>TCTTGAATGTACTGAGCATAGTCATTGCAGCCCTGGTGGAATCAAAGCGGCTC<br>CAAATAGCTCGAGCCAGCTACATTGTCCAAGAATTGACCAGTTCCACCGTGCC<br>AATGTCTGTTTTCTGGTTAGTTCCGCAGCTTGCACTTTCGGGAATGGGAGAAG<br>CATTTCATTTTCCAGGACAAGCTTCATTATACTATCAAGAATTCCCTGCATCCC<br>TTAAAAGCACGTCGACTGCAATGGTTGCACTGCTTATAGCAATTGGATACTAT<br>TTGAGCACGGCCTTGACAGATTTTGTGCGGAAGGTAACGAATTGGTTGCCAGA<br>TGATTTGAATCATGGAAGGCTCAACTATTTGTATTGGGTGCTGGCTATGATTG<br>GTGCATTGAATTTTGGCCTTTATTTAACATCTGCTGGGGTCTACAAGTATAGAA<br>ATGATCAGGATGACAAGACGGTCGATAATAGTTCAAACCAAGGGGATGCAAC<br>GTTGTACTACTAG
*Cp*DCS<br>(MW456554)	>ATGGCCGGAAAATCTCAAGAAGATGGGCAGACGGTAAAGGCTCTAGGATGG<br>GCCGCTAGGGAAGTTTCTGGGGCGATCTCTCCTTTCGATTTCTCAAGAAGGGC

	CCCAGGAGAGCGCGATGTGCAGGTTAAAATACTATATTGTGGAATCTGTAGTT<br>TTGACACAGAAATGATCAATAACAAGTTTGGCTTTACCAGATATCCCTTTGTA<br>CTCGGGCATGAGATTGTGGGAGTGGTATCTGAAGTTGGTAGAAAGGTGCAAA<br>AATTCAAGATTGGGGATAAAGTTGGTGTAGGAACCATGATTGGATCTTGTCGC<br>ACTTGTTATAGCTGCACTCACAATCTCGAAAATTACTGCCCAAAAGTTACATT<br>AACAGAAGCAACTTCTGGTGGTTGTTCTAATCTTGTGATAGCAGATGAAGACT<br>TTGTGTTCCATTGGCCGGTGAATTTGCCTCTTGATCTTGGAGCTCCTCTCCTTT<br>GTGCTGGGATTACTGTTTATAGCCCTTTGAAAAATTTTGAACTTGATAAGCCTG<br>GATTGCGTATTGGTGTGGTTGGTCTTGGTGGTATTGGCCATATAGCTGTAAAA<br>TTTGCCAAGGCTTTTGGGGCTAAGGTGACAGTGATTAGTTCATCAGAAAGTAA<br>AAAGGTTGAAGCCATTGAAAAATATGGTGCAGATTCCTTTTTGGTTAGCAGTG<br>ATCCAGGGCAGATGCTGGCAGCTGCCGGAACCTTGGATGGTGTCATTGATACC<br>GTCCCAGCACCTCACTCTATTTTGCCATTCCTTGATTTACTCTTGCCTCGTGGA<br>AAGCTAATTATATTAGGTGCACCAATGGAGCCATTTGTACTGCCAATCTATCC<br>CCTGCTTCAAGGTGGGAGAGTAGTTGCTGGGAGTGCCACTGGAGGATTGAAA<br>CAAATCCAAGAAATGCTTCATTTTGCAGCAGAGCACAACATAGTAGCAGATG<br>GCGAGGTTATCCCAATCGACGACATTAACACTGCGATAAAGCGCATTGAGAA<br>AGGCGATGTCAAATATCGATTTGTGGTTGACATTGGCAATACCTTAAAATCTG<br>CTTGA
*Cp*DCE<br>(MW456556)	>ATGGAGAAACATTTTGTGCTAATCCATGGAGGTTGTTTCGGGGCATGGGCAT<br>GGTACAAAGTGGTGACAATCTTGGAATCCAACGGCTACAAAGCCACTGCCCTT<br>GATATGGCTTCTTCTGGGATCAATCCCAGACGTACAGACGAGGTGAAATCCTA<br>CTCCGATTATTCTGAGCCGTTGATCAAGTTCATGGAGGATTTACCATCAAATG<br>AGAGAGTGGTTTTGGTTGGGCACAGTTTGGCTGGAGTTATTGTTTCTTTGGCTA<br>TGGAAAAATTCCCTCAGAAAATTGCTGCTGGCGTTTTCCTCACTGCTGTCATGC<br>CTGGTCCTGAGATCACTATGGCAACGCTTCATGAGGAGCACAAGGAACAAAT<br>TGATACTTTCATGGACTGCCAAATGATCCACGGCAATGGCGATGATAATCCTC<br>CGACTGCTTTCCTCTTTGGTCTCGAGTACTTAAAATCCAAAGTGTTTCAACTCT<br>GTCCTCCTGAGGACATGACACTCGCATCCCTTTTGGTAAGGCCAATATCTCTG<br>GCAATCGAGCAAGGAGAACTAGACATCCCACTCACCCAGATTAACTACGGTT<br>CGGTTCCTCGTATTTACCTCATATCTGAAAATGACAACGTGACAAAGGTCGAA<br>GTTCAGAGATGGATGGTCGACAAAAATCCACCCGAAGAAGTCTTCGTGATCCC<br>TAGTTGCGATCATATGGTCATGTTATCCAATCCCAAAGATCTAAGCTCTCGTTT<br>GCTGGAAATTGCCCAGAAATATGACTAA
*Cr*SGD<br>(AF112888)	>ATGGGATCTAAAGATGATCAGTCCCTTGTTGTTGCCATTTCTCCAGCTGCTGA<br>ACCAAATGGAAATCATTCTGTCCCCATCCCATTCGCCTACCCCAGTATCCCCAT<br>TCAACCTAGAAAGCACAACAAGCCCATCGTTCATCGTCGAGATTTCCCCTCAG<br>ATTTCATCTTGGGTGCCGGAGGATCTGCTTATCAGTGTGAGGGTGCATATAAT<br>GAAGGCAACCGCGGTCCCAGTATATGGGATACTTTCACAAACCGATATCCAGC<br>CAAAATAGCTGATGGATCTAATGGCAATCAAGCCATCAATTCTTACAATTTGT<br>ACAAGGAAGATATCAAGATTATGAAGCAAACAGGCTTGGAATCATATAGGTT<br>TTCAATTTCATGGTCAAGAGTATTGCCAGGTGGGAATCTATCCGGTGGAGTGA<br>ATAAAGATGGTGTCAAGTTCTATCATGACTTTATAGATGAGCTTCTAGCCAAT<br>GGCATCAAACCCTTTGCAACTCTCTTCCACTGGGATCTTCCCCAAGCTCTTGAA

	GACGAGTATGGAGGCTTCTTGAGTGATCGAATTGTGGAAGATTTTACGGAGTA<br>TGCAGAATTTTGCTTTTGGGAATTCGGTGACAAAGTAAAATTTTGGACGACTT<br>TCAATGAACCACATACTTATGTTGCAAGTGGATATGCCACTGGTGAATTTGCA<br>CCAGGAAGAGGTGGTGCAGATGGCAAGGGGGAACCTGGCAAAGAACCCTATA<br>TAGCGACACATAATTTACTTCTTTCTCACAAAGCTGCTGTGGAAGTATATAGG<br>AAAAATTTTCAGAAATGTCAAGGAGGTGAAATTGGAATTGTACTTAATTCAAT<br>GTGGATGGAGCCTCTCAATGAAACCAAAGAAGATATTGATGCTCGGGAAAGG<br>GGTCTTGATTTCATGCTCGGATGGTTCATAGAGCCATTAACAACGGGTGAATA<br>CCCAAAATCCATGAGAGCTCTTGTAGGAAGCCGTCTTCCAGAATTTTCAACAG<br>AAGTTTCCGAAAAATTAACAGGATGCTATGATTTTATCGGAATGAATTATTAT<br>ACAACTACTTATGTTTCTAATGCAGACAAAATTCCCGATACTCCGGGTTACGA<br>AACAGATGCTCGAATTAATAAGAATATTTTTGTCAAAAAAGTTGATGGGAAG<br>GAAGTGCGCATTGGTGAACCGTGCTATGGGGGATGGCAGCATGTTGTTCCATC<br>TGGACTCTACAATCTCTTGGTTTACACTAAGGAGAAATACCATGTTCCAGTGA<br>TTTATGTCTCAGAATGTGGTGTGGTTGAGGAAAATAGAACCAACATATTACTT<br>ACAGAAGGTAAAACCAACATATTACTTACAGAAGCTCGTCACGATAAACTCA<br>GGGTTGATTTTCTACAAAGTCATCTCGCTAGCGTGCGAGATGCTATTGATGAT<br>GGTGTGAATGTAAAAGGATTCTTTGTTTGGTCATTCTTCGACAACTTCGAATG<br>GAATTTGGGATATATATGCCGTTATGGAATTATCCATGTTGATTATAAAACTTT<br>TCAAAGATATCCAAAGGATTCTGCCATATGGTACAAGAATTTCATTAGTGAAG<br>GATTTGTTACGAATACAGCTAAAAAGAGATTCCGAGAAGAAGATAAACTAGT<br>TGAGTTAGTCAAGAAGCAAAAATACTAA

Table S4. Primers used for genes cloning in this study.

Gene	Plasmid	Primer direction	Sequence (5'-3')
N. benthamiana single transcriptional unit constructs (In-Fusion cloning)			
*Cp*MAT	p3Ω1	Forward	TTTATGAATTTTGCAGCTCGATGGCGTCAACAATTATGATCAT
		Reverse	GACAACCACAACAAGCACCGCTATGAAACCAAAGAAGCAAATGCAA
*Cp*MCC	p3Ω1	Forward	TTTATGAATTTTGCAGCTCGATGGCTTCAAGCAAAACAGTG
		Reverse	GACAACCACAACAAGCACCGCTACAAATCCCTCAGACCAT
*Cp*CiS	p3Ω1	Forward	TTTATGAATTTTGCAGCTCGATGGCTTCCGAAATCATAAAGCTT
		Reverse	GACAACCACAACAAGCACCGCTAAGCACTGATGCTAGTGC
*Cp*CiR	p3Ω1	Forward	TTTATGAATTTTGCAGCTCGATGGCTGGGCAGAATTTAAGG
		Reverse	GACAACCACAACAAGCACCGCTAAGTGCTTGGGGTAGCCACAA
*Cp*CiO	p3Ω1	Forward	ACCCTCACTAAAGGGCGGCCGCAACCATGGCTACTTTCCCATTCC
		Reverse	GTCATCCTTGTAATCCATCGATACACATGCTGATGCAACAACTT
*Cp*KR4	p3Ω1	Forward	TTTATGAATTTTGCAGCTCGATGGAAGAAGGCAATCAAATCC
		Reverse	GACAACCACAACAAGCACCGCTATGTAGCTGGTGGATTCTTA
*Cp*KR4 (with His tag)	p3Ω1	Forward	TTTATGAATTTTGCAGCTCGATGGCACACCATCACC
		Reverse	GACAACCACAACAAGCACCGCTAATCACAAACTGGTCTAGATGTA
*Cp*SGD1	p3Ω1	Forward	TTTATGAATTTTGCAGCTCGATGGAGATCCATCGTTCGGATT
		Reverse	GACAACCACAACAAGCACCGCTAATTTGCACGAAGCCTCTTA
*Cp*SGD2	p3Ω1	Forward	TTTATGAATTTTGCAGCTCGATGGTGGAGTTGACAATGCTCAA
		Reverse	GACAACCACAACAAGCACCGCTACAAAGTGTTGTAGATATTAACATCA
*Cp*SGD3	p3Ω1	Forward	TTTATGAATTTTGCAGCTCGATGGCTCTAATTATGAACTTTTGCATA
		Reverse	GACAACCACAACAAGCACCGCTAGTTGGAGCCTTTCGAGAA
Sequencing primers for p3Ω1		Forward	GATGAAAAAGCCCTAAAATTGGAG
		Reverse	ATTATTCACAAATGAGAAACAGAATGG
E. coli BL21 (DE3) expression constructs (In-Fusion cloning)			
*Cp*MAT	pOPINF	Forward	AAGTTCTGTTTCAGGGCCCGGCGTCAACAATTATGATCAT
		Reverse	ATGGTCTAGAAAGCTTTATGAAACCAAAGAAGCAAATGCAA
*Cp*MCC	pOPINF	Forward	AAGTTCTGTTTCAGGGCCCGGCTTCAAGCAAAACAGTG
		Reverse	ATGGTCTAGAAAGCTTTACAAATCCCTCAGACCAT
*Cp*CiS	pOPINF	Forward	AAGTTCTGTTTCAGGGCCCGGCTTCCGAAATCATAAAGC
		Reverse	ATGGTCTAGAAAGCTTTAAGCACTGATGCTAGTGC
*Cp*KR4	pOPINF	Forward	AAGTTCTGTTTCAGGGCCCGGAAGAAGGCAATCAAATCCAAATTCCA AGAG
		Reverse	ATGGTCTAGAAAGCTTTATGTAGCTGGTGGATTCTTATTTTTGGGAG
*Cp*KR1	pOPINF	Forward	AAGTTCTGTTTCAGGGCCCGTCTGACCAAGTTGAGATGCG
		Reverse	ATGGTCTAGAAAGCTTTACTTCTTGAGTGGTGGTGTATTA
*Cp*KR2	pOPINF	Forward	AAGTTCTGTTTCAGGGCCCGGCCACTGGGATAAAGTACTT
		Reverse	ATGGTCTAGAAAGCTTTAGACCTCACCATCCCAGATTTC

*Cp*KR3	pOPINF	Forward	AAGTTCTGTTTCAGGGCCCGGCAACATCAGTGAGGAAGATCAAACTG G
		Reverse	ATGGTCTAGAAAGCTTTATGTAGCTTTCCACGAGGACAAGG
*Cp*KR5	pOPINF	Forward	AAGTTCTGTTTCAGGGCCCGGCATCAGAAGAGGTGAAAGTG
		Reverse	ATGGTCTAGAAAGCTTTATATACCTTTCCATGTTGACAAAGG
*Cp*KR6	pOPINF	Forward	AAGTTCTGTTTCAGGGCCCGGCAACAGTGGTGAGGAAGAT
		Reverse	ATGGTCTAGAAAGCTTTAAGTAGCTTTCCACGAGGACA
*Cp*KR7	pOPINF	Forward	AAGTTCTGTTTCAGGGCCCGGCGAGTCTACCAAAAGTGC
		Reverse	ATGGTCTAGAAAGCTTTACTCAGCTTTCCAGGAAGACA
*Cp*KR8	pOPINF	Forward	AAGTTCTGTTTCAGGGCCCGGCAACCGAAGAGGTGAAAGTGC
		Reverse	ATGGTCTAGAAAGCTTTATATACCTTTCCATGTTGACAAAGGTGGAGT
Sequencing primers for pOPINF		Forward	TAATACGACTCACTATAGGG
		Reverse	TAGCCAGAAGTCAGATGCT
S. cerevisiae WAT11 expression constructs (In-Fusion cloning)			
*Cp*CiO	pESC-His	Forward	GAGAAAAAACCCCGGATCCATGGCTACTTTCCCATTCC
		Reverse	ACTTCTGTTCCATGTCGACTTAACATGCTGATGCAACAACTT
Sequencing primers for pESC-His		Forward	ATGATTTTTGATCTATTAACAGATA
		Reverse	GTATAATGTTACATGCGTACAC

Table S5. Primers for VIGS gene fragments amplification

Gene	Primer sequence	
*Cp*MAT	forward	ACATTGCTGCGGATCCGCACGCCCCCGGAAGATG
	reverse	ATGCCCGGGCCTCGAGGTTCCCCAATTTTGGAGGCC
*Cp*MCC	forward	ACATTGCTGCGGATCCCGATCACGGATGCAAATGTGG
	reverse	ATGCCCGGGCCTCGAGGTTACTGCAACACCTGATTTTGACC
*Cp*CiO	forward	ACATTGCTGCGGATCCGCTCCCAATAATCGGAAACCTTC
	reverse	ATGCCCGGGCCTCGAGGTTTGACTTAGAATCAGATGTACAC
*Cp*MgChl	forward	TAAGGTTACCGAATTCCAAGGGGCTCAAGCAGCTCA
	reverse	GCTCGGTACCGGATCCGCAGCAATGTTAACTAGTGTGGCA

Table S6. VIGS gene fragments

Genes	Fragment sequence
*Cp*MgChl fragment (300 bp)	CAAGGGGCTCAAGCAGCTCAGTGAGCTTATTTCCTCTTACCAATCCCTAAAAGA CACTGGTCGTGGCTCACAAATTGTAAGCTCTATTATCAGCACAGCTAGACAATG TAATCTTGACAAGGATGTAGATCTTCCTGAAGAAGGGCAAGAAATATCAGCCAG AGAGCATGACCTTGTGGTTGGAAAAGTATATTCCAAAATTATGGAAATTGAATC CAGACTTTTACCCTGTGGGCTTCATGTCATTGGTGAGCCTCCATCTGCAATGGAG GCAGTTGCCACACTAGTTAACATTGCTGC
*Cp*MAT fragment (300 bp)	GCACGCCCCCGGAAGATGGCAGCCTGTTGATTGTTCAGGTCAGCTATTTTGACT GTGGAGGCGTGGCTGTAGGCCTCTGTATATCTCACAAGATCTCTGATGCGTCCA CTAGATGCACTTTGAGCAATGATTGGGCTGCCGTGGCACGCCAGCCGAGTTATG

	TCCCGACTCCAAAGTTTAACGGAGCTTCTGTCTTTCCACCAGTtGATGACGTATC GTTTCAGGAACTCATCGCTTCGCCACCAACTGAAAATTGCGTTGCTAAGAGATT CTTGTTTAAGGCCTCCAAAATTGGGGAAC
*Cp*MCC fragment (298 bp)	CGATCACGGATGCAAATGTGGTGGTAGGTTTCTTAAAAGCATGGGGTTCGGTGT ATAAAAACAAGGGAGATGCAGAGTTGGTAGCCACTTCTTCGGTACCATTTCTTG ATAGATCAGCTCTGATCAAAGAAAGGGAAGTTCTTGATAGAGTACTTTCGATAG ATCCACAATCAATCAACTTTAACTTGAGCCCAGCTGTAATGCTGTCGGAAAACT TGGTCCGAGCCTCCTTTGTGATCTGTGAAGTTGATGTTGTGAAGCTCAAGCAATC AGTGAGGTCAAAATCAGGTGTTGCAGT
*Cp*CiO fragment (303 bp)	GCTCCCAATAATCGGAAACCTTCATCAGATCGGTCCGCTCGCCCACCGCTCTTTC AAATCCTTGGCGGAAAAATATGGTCCAGTTATGCTGTTTCACTTAGGCAGCCGA CCACTTTTAGTAGTCTCCTCTGCTGAGGCAGCTCACCAAGTCCTCGTAGCAAAAG ATGTGGACTTCGCGGATAAATCTAATACGATGGTTGGCAGACGGCTTTACTACG AAAGCAAGGCTATAGCTTTTGCTCCTTATGGTGAATATTGGAAGCAAATAAGGG GAATCAGTGTACATCTGATTCTAAGTCAAAC

Table S7. Primers for qPCR

**Genes**	**qPCR Primer sequence**		**Amplicon length (bp)**
*Cp*MgChl	Forward	CATGGATTCGCTGCTTACTACTC	118 bp
	Reverse	CACTCATTCCCACCTGTTTCC	
*Cp*MAT	Forward	GATCCATAACTGTCCCTCCAC	119 bp
	Reverse	CCTAAACTTATGAACCAGTTCGG	
*Cp*MCC	Forward	GAATTGGCGATTTACTCCGAC	106 bp
	Reverse	CCACCTTTCAACAATCCCTC	
*Cp*CiO	Forward	GAGAGCATATTGATTCGTCTTCGG	102 bp
	Reverse	CTGAGAATCTCCTCCCAATCAC	
*Cp*2227	Forward	GGAGAACAAGGGAGATGGAG	104 bp
	Reverse	GGTCTAACCAATCAGGAGGAG	
*Cp*Actin	Forward	GTAGAGAAGAGCTATGAATTGCC	103 bp
	Reverse	CCATTCCAATCATTGAAGGCTG	

Table S8. Tabulated NMR data of corynantheol (11) in methanol-d_3

pos.	δ_C[a], type	δ_H[b], *mult* (*J* in Hz)
1	-	10.27, *s*
2	135.5, C	-
3	61.3, CH	α 3.32, *m*
5	54.0, CH_2	β 3.11, *dd* (11.4, 59)
		α 2.63, *ddd* (11.8, 11.4, 4.6)
6	22.2, CH_2	β 2.99, *dddd* (14.9, 11.8, 5.9, 2.3)
		α 2.73, p*dd* (14.9, 4.6)
7	107.6, C	-
8	128.2, C	-
9	118.5, CH	7.38, *d* (7.9)
10	119.7, CH	6.96 *ddd* (7.9, 7.7, 0.8)
11	121.9, CH	7.04, *ddd* (8.1, 7.3, 1.0)
12	119.9, CH	7.29, *d* (8.1)
13	138.1, C	-
14	35.0, CH_2	α 2.51, *ddd* (13.0, 3.1, 2.5)
		β 1.24, *ddd* (13.0, 12.0, 11.2)
15	37.7, CH	α 1.58, *m*
16	37.4, CH_2	1.93, *m*
		1.29, *m*[§]
17	60.5, CH_2	3.72, *m*
		3.67, *m*
18	117.3, CH_2	5.16, *dd* (17.3, 1.6)<br>5.12, *dd* (10.3, 1.8)
19	140.6, CH	5.63, *ddd* (17.3, 10.3, 9.1)
20	48.1, CH	β 2.19, *m*
21	61.9, CH_2	2.90, *dd* (11.8, 4.8)
		2.33, *dd* (11.8, 11.3)

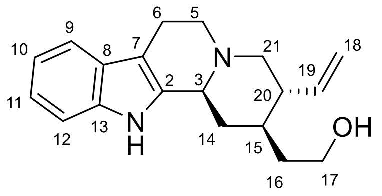

11

[a] measured at 176 MHz; [b] measured at 700 MHz; [§] unresolved, overlapping signals

Table S9. Tabulated NMR data of cinchonium (12) in methanol-d_4

pos.	δ_C[a], type	δ_H[b], *mult* (*J* in Hz)
2	129.6, C	-
3	62.3, CH	4.98, *brdd* (9.3, 8.9)
5	61.3, CH_2	3.71-3.68, *m*
6	18.3, CH_2	β 3.16, *td* (8.8, 2.4)
		α 3.04, *m**
7	105.6, C	-
8	127.0, C	-
9	119.4, CH	7.49, *brd* (8.1)
10	121.0, CH	7.08, *ddd* (8.1, 7.1, 1.0)
11	123.8, CH	7.17, *ddd* (8.2, 7.0, 1.2)
12	112.8, CH	7.40, *brd* (8.2)
13	138.9, CH	-
14	27.4, CH_2	α 2.81, *m*
		β 2.00-1.94, *m**
15	27.7, CH	α 2.29, *m*
16	25.8, CH_2	2.07, *m*
		2.00-1.94, *m**
17	49.5, CH_2	3.38, *dddd* (12.6, 10.8, 3.2, 1.8)
		3.67, *m*
18	118.1, CH_2	5.39, *brd* (17.2); 5.34, *brd* (10.5)
19	139.4, CH	6.16, *ddd* (17.3, 10.5, 6.9)
20	39.8, CH	β 3.04, *m**
21	63.5, CH_2	β 3.95, *dd* (12.8, 10.4)
		α 3.58, *dt* (12.9, 3.3)

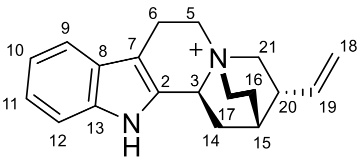

12

[a] measured at 100 MHz; [b] measured at 400 MHz; * overlapping signals

Table S10. Tabulated NMR data of cyclized cinchonaminal (cyclocinchonaminal, 13) in acetonitrile-d_3.

pos.	δ_C[a], type		δ_H[b], *mult* (*J* in Hz)	
	at 243K	at 298K	at 243K	at 298K
1	-	-	10.09, *s*	9.63, *s*
2	128.7, C	130.5, C	-	-
3	59.6, CH	58.5, CH	4.66, *brs*	4.69, *brdd* (9.2, 8.9)
5	92.7, CH	-**	5.21, *m*[§]	5.80*
6	26.5, CH_2	28.4, CH_2	α 3.01, *brd* (16.5)	α 3.26, *m*[§]
			β 2.85, *m*[§]	β 3.18, *m*[§]
7	105.2, C	105.2, C	-	-
8	125.9, C	127.2, C	-	-
9	118.8, CH	119.2, CH	7.41, *m*[§]	7.47, *d* (7.8)
10	120.0, CH	120.6, CH	7.06, *dd* (7.8, 7.5)	7.09, *dd* (7.9, 7.6)
11	122.8, CH	123.3, CH	7.17 *dd* (7.7, 7.5)	7.18, *dd* (8.1, 7.6)
12	112.0, CH	112.4, CH	7.41, *m*[§]	7.42, *d* (8.1)
13	137.6, C	138.1, C	-	-
14	26.7, CH_2	26.9, CH_2	α 2.59, *m*	α 2.64, *m*
			β 1.83, *m*[§]	β 1.87, *m*[§]
15	27.0, CH	27.5, CH	2.16, *m*	2.18, m
16	23.9, CH_2	25.2, CH_2	1.82 - 1.85, *m*[§]	1.82-1.88, *m*[§]
17	40.6, CH_2	42.9, CH_2	3.39, *m*[§]	3.33, *m*[§]
			3.14, *m*	3.20, *m*[§]
18	116.9, CH_2	117.1, CH_2	5.31, *m*<br>5.21, *m*[§]	5.30, *d* (17.2); 5.23, *d* (10.4)
19	138.9, CH	139.9, CH	5.95, *m*	6.03, *ddd* (17.1, 10.6, 6.7)
20	38.1, CH	38.9, CH	β 2.85, *m*[§]	β 2.85, *m*
21	56.0, CH_2	57.0, CH_2	β 3.83, *m*	β 3.74, *brdd* (13.2, 11.2)
			α 3.39, *m*[§]	α 3.44, *m*

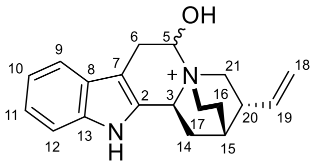

13

[a] measured at 125 MHz; [b] measured at 500 MHz; * The chemical shift was not stable during the measurements; [§] overlapping signals; **the signal was not detected at 298K.

Table S11. Tabulated NMR data of malonyl-corynantheol (14).

	in DMSO-d_6		in methanol-d_3**	
pos.	δ_C[a], type	δ_H[b], *mult* (*J* in Hz)	δ_C[a], type	δ_H[b], *mult* (*J* in Hz)
1	-	11.71	-	10.79, *s*
2	136.2, C	-	133.0, C	-
3	59.7, CH	3.10, *d* (11.2)	61.1, CH	3.88, *d* (11.3)
5	52.5, CH_2	β 2.95, *dd* (11.2, 5.7)	53.4, CH	3.43, *m*
		α 2.45, *ddd* (11.3, 11.2, 4.1)		3.05, *m*
6	21.7, CH_2	β 2.77, *m*	21.0, CH_2	3.10, *m*
		α 2.58, *m*		2.89, *brd* (12.3)
7	105.5, C	-	-***	-
8	126.6, C	-	127.7, C	-
9	117.2, CH	7.31, *d* (7.9)	118.5, CH	7.41, *d* (7.7)
10	117.9, CH	6.89, *dd* (7.9, 7.3)	120.0, CH	7.00, *dd* (7.7, 7.4)
11	119.8, CH	6.96, *dd* (7.8, 7.3)	122.3, CH	7.09, *dd* (8.0, 7.4)
12	111.5, CH	7.34, *d* (7.8)	112.3, CH	7.38, *d* (8.0)
13	136.3, C	-	138.3, C	-
14	34.6, CH_2	α 2.84, *m**	33.9, CH_2	2.81, *brd* (14.3)
		β 1.00, *ddd* (12.0, 11.4, 10.8)		1.36-1.50, *m*§
15	38.5, CH	α 1.45, *m*	37.6, CH	1.72, *m*
16	31.6, CH_2	1.85, *m*	32.3, CH_2	2.03, *m*
		1.29, *m*		1.36-1.50, *m*§
17	63.2, CH_2	4.13, *m*	63.9, CH_2	4.22, *m*
		3.99, *m*		
18	116.7, CH_2	5.13, *dd* (17.5, 1.6)	118.3, CH_2	5.23, *d*(17.5)<br>5.21, *dd* (10.5, 1.1)
		5.08, *dd* (10.6, 1.9)		
19	140.2, CH	5.62, *ddd* (17.5, 10.6, 9.3)	138.8, CH	5.62, *ddd* (17.5, 10.5, 9.1)
20	47.1, CH	β 2.05, *m*	46.9, CH	2.29, *m*
21	60.7, CH_2	β 2.82, *m**	59.8, CH_2	3.17, *dd* (12.0, 4.0)

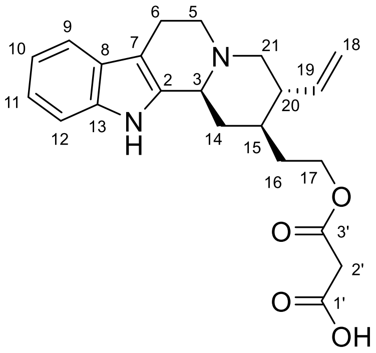

14

		α 2.20, *dd* (11.3, 10.9)		2.72, *dd* (12.0, 11.5)
1′	168.6, C	-	171.1, C	-
2′	46.1, CH_2	3.01, *d* (13.9); 2.97, *d* (13.9)	45.7, CH_2	3.27, *brs*
3′	169.7, C	-	173.5, C	-

[a] deduced from HSQC, HMBC and/or COSY correlations; [b] measured at 700 MHz; [§] or [*] overlapping signals; [**]NOTE: this compound was slowly hydrolyzed during the long-term measurements in MeOH-d_3, thus measuring it in MeOH-d_3 is not appropriate; [***]sufficient HMBC was not obtained to identify the chemical shift.

References

1 Trenti, F. *et al.* Early and late steps of quinine biosynthesis. *Organic Letters* **23**, 1793-1797 (2021). https://doi.org/10.1021/acs.orglett.1c00206

2 Lombe, B. K., Zhou, T., Caputi, L., Ploss, K. & O'Connor, S. E. Biosynthetic origin of the methoxy group in quinine and related alkaloids. *Angewandte Chemie International Edition* **64**, e202418306 (2025). https://doi.org/10.1002/anie.202418306

3 Schotte, C. *et al.* Directed biosynthesis of mitragynine stereoisomers. *Journal of the American Chemical Society* **145**, 4957-4963 (2023). https://doi.org/10.1021/jacs.2c13644

4 https://nanoporetech.com/software/other/guppy/history?version=6-3-2

5 Shen, W., Le, S., Li, Y. & Hu, F. SeqKit: A cross-platform and ultrafast toolkit for FASTA/Q file manipulation. *PLOS ONE* **11**, e0163962 (2016). https://doi.org/10.1371/journal.pone.0163962

6 Kolmogorov, M., Yuan, J., Lin, Y. & Pevzner, P. A. Assembly of long, error-prone reads using repeat graphs. *Nature Biotechnology* **37**, 540-546 (2019). https://doi.org/10.1038/s41587-019-0072-8

7 https://github.com/nanoporetech/medaka

8 Nataly Allasi, C. *et al.* A highly contiguous, scaffold-level nuclear genome assembly for the fever tree *Cinchona pubescens* as a novel resource for *Rubiaceae* research. *Gigabyte* **2022**, 0 (2022). https://doi.org/10.46471/gigabyte.71

9 Aury, J.-M. & Istace, B. Hapo-G, haplotype-aware polishing of genome assemblies with accurate reads. *NAR Genomics and Bioinformatics* **3** (2021). https://doi.org/10.1093/nargab/lqab034

10 Manni, M., Berkeley, M. R., Seppey, M., Simão, F. A. & Zdobnov, E. M. BUSCO Update: Novel and streamlined workflows along with broader and deeper phylogenetic coverage for scoring of eukaryotic, prokaryotic, and viral genomes. *Molecular Biology and Evolution* **38**, 4647-4654 (2021). https://doi.org/10.1093/molbev/msab199

11 Chen, N. Using RepeatMasker to identify repetitive elements in genomic sequences. *Current Protocols in Bioinformatics* **5**, 4.10.11-14.10.14 (2004). https://doi.org/10.1002/0471250953.bi0410s05

12 Flynn, J. M. *et al.* RepeatModeler2 for automated genomic discovery of transposable element families. *Proceedings of the National Academy of Sciences* **117**, 9451-9457 (2020). https://doi.org/doi:10.1073/pnas.1921046117

13 Campbell, M. S., Holt, C., Moore, B. & Yandell, M. Genome annotation and curation using MAKER and MAKER-P. *Current Protocols in Bioinformatics* **48**, 4.11.11-14.11.39 (2014). https://doi.org/10.1002/0471250953.bi0411s48

14 Bao, W., Kojima, K. K. & Kohany, O. Repbase Update, a database of repetitive elements in eukaryotic genomes. *Mobile DNA* **6**, 11 (2015). https://doi.org/10.1186/s13100-015-0041-9

15 Martin, M. Cutadapt removes adapter sequences from high-throughput sequencing reads. *2011* **17**, 3 (2011). https://doi.org/10.14806/ej.17.1.200

16 Kim, D., Paggi, J. M., Park, C., Bennett, C. & Salzberg, S. L. Graph-based genome alignment and genotyping with HISAT2 and HISAT-genotype. *Nature Biotechnology* **37**, 907-915 (2019). https://doi.org/10.1038/s41587-019-0201-4

17 https://github.com/nanoporetech/pychopper

18 Li, H. New strategies to improve minimap2 alignment accuracy. *Bioinformatics* **37**, 4572-4574

(2021). https://doi.org/10.1093/bioinformatics/btab705

19 Kovaka, S. *et al.* Transcriptome assembly from long-read RNA-seq alignments with StringTie2. *Genome Biology* **20**, 278 (2019). https://doi.org/10.1186/s13059-019-1910-1

20 Brůna, T., Hoff, K. J., Lomsadze, A., Stanke, M. & Borodovsky, M. BRAKER2: automatic eukaryotic genome annotation with GeneMark-EP+ and AUGUSTUS supported by a protein database. *NAR Genomics and Bioinformatics* **3** (2021). https://doi.org/10.1093/nargab/lqaa108

21 Campbell, M. A., Haas, B. J., Hamilton, J. P., Mount, S. M. & Buell, C. R. Comprehensive analysis of alternative splicing in rice and comparative analyses with Arabidopsis. *BMC Genomics* **7**, 327 (2006). https://doi.org/10.1186/1471-2164-7-327

22 Altschul, S. F., Gish, W., Miller, W., Myers, E. W. & Lipman, D. J. Basic local alignment search tool. *Journal of Molecular Biology* **215**, 403-410 (1990). https://doi.org/10.1016/S0022-2836(05)80360-2

23 Lamesch, P. *et al.* The Arabidopsis information resource (TAIR): improved gene annotation and new tools. *Nucleic Acids Research* **40**, D1202-D1210 (2011). https://doi.org/10.1093/nar/gkr1090

24 Boutet, E. *et al.* in *Plant Bioinformatics: Methods and Protocols* (ed David Edwards) 23-54 (Springer New York, 2016).

25 Li, W. *et al.* The EMBL-EBI bioinformatics web and programmatic tools framework. *Nucleic Acids Research* **43**, W580-W584 (2015). https://doi.org/10.1093/nar/gkv279

26 Mistry, J. *et al.* Pfam: The protein families database in 2021. *Nucleic Acids Research* **49**, D412-D419 (2020). https://doi.org/10.1093/nar/gkaa913

27 Li, C. *et al.* Nuclei isolation protocol from diverse angiosperm species. *bioRxiv*, 2022.2011.2003.515090 (2022). https://doi.org/10.1101/2022.11.03.515090

28 Hao, Y. *et al.* Integrated analysis of multimodal single-cell data. *Cell* **184**, 3573-3587.e3529 (2021). https://doi.org/10.1016/j.cell.2021.04.048

29 McGinnis, C. S., Murrow, L. M. & Gartner, Z. J. DoubletFinder: doublet detection in single-cell RNA sequencing data using artificial nearest neighbors. *Cell Systems* **8**, 329-337.e324 (2019). https://doi.org/10.1016/j.cels.2019.03.003

30 https://github.com/junjunlab/ClusterGVis

31 Love, M. I., Huber, W. & Anders, S. Moderated estimation of fold change and dispersion for RNA-seq data with DESeq2. *Genome Biology* **15**, 550 (2014). https://doi.org/10.1186/s13059-014-0550-8

32 Tang, D. *et al.* SRplot: A free online platform for data visualization and graphing. *PLOS ONE* **18**, e0294236 (2023). https://doi.org/10.1371/journal.pone.0294236

33 https://orthovenn3.bioinfotoolkits.net

34 Cárdenas, P. D. *et al.* Pathways to defense metabolites and evading fruit bitterness in genus Solanum evolved through 2-oxoglutarate-dependent dioxygenases. *Nature Communications* **10**, 5169 (2019). https://doi.org/10.1038/s41467-019-13211-4

35 Berrow, N. S. *et al.* A versatile ligation-independent cloning method suitable for high-throughput expression screening applications. *Nucleic Acids Res* **35**, e45 (2007). https://doi.org/10.1093/nar/gkm047

36 Kamileen, M. O. *et al.* Streamlined screening platforms lead to the discovery of pachysiphine synthase from Tabernanthe iboga. *New Phytologist* **244**, 1437-1449 (2024). https://doi.org/10.1111/nph.20133

37 Urban, P., Mignotte, C., Kazmaier, M., Delorme, F. & Pompon, D. Cloning, Yeast expression, and characterization of the coupling of two distantly related *Arabidopsis thaliana* NADPH-cytochrome P450 reductases with P450 CYP73A5. *Journal of Biological Chemistry* **272**, 19176-19186 (1997). https://doi.org/10.1074/jbc.272.31.19176

38 Sparkes, I. A., Runions, J., Kearns, A. & Hawes, C. Rapid, transient expression of fluorescent fusion proteins in tobacco plants and generation of stably transformed plants. *Nature Protocols* **1**, 2019-2025 (2006). https://doi.org/10.1038/nprot.2006.286

39 Pompon, D., Louerat, B., Bronine, A. & Urban, P. Yeast expression of animal and plant P450s in optimized redox environments. *Methods Enzymol* **272**, 51-64 (1996). https://doi.org/10.1016/s0076-6879(96)72008-6

40 http://www.bioinformatics.com.cn/srplot

41 O'Leary, B. M., Rico, A., McCraw, S., Fones, H. N. & Preston, G. M. *The Infiltration-centrifugation Technique for Extraction of Apoplastic Fluid from Plant Leaves Using Phaseolus vulgaris as an Example*. Vol. 94 (1940-087X, 2014).

42 Hughes, C. S. *et al.* Ultrasensitive proteome analysis using paramagnetic bead technology. *Molecular Systems Biology* **10**, 757 (2014). https://doi.org/10.15252/msb.20145625

43 Cox, J. & Mann, M. MaxQuant enables high peptide identification rates, individualized p.p.b.-range mass accuracies and proteome-wide protein quantification. *Nature Biotechnology* **26**, 1367-1372 (2008). https://doi.org/10.1038/nbt.1511

44 Perez-Riverol, Y. *et al.* The PRIDE database at 20 years: 2025 update. *Nucleic Acids Research* **53**, D543-D553 (2024). https://doi.org/10.1093/nar/gkae1011

45 Liu, Y., Schiff, M., Marathe, R. & Dinesh-Kumar, S. P. Tobacco Rar1, EDS1 and NPR1/NIM1 like genes are required for *N*-mediated resistance to tobacco mosaic virus. *The Plant Journal* **30**, 415-429 (2002). https://doi.org/10.1046/j.1365-313X.2002.01297.x

46 Liscombe, D. K. & O'Connor, S. E. A virus-induced gene silencing approach to understanding alkaloid metabolism in Catharanthus roseus. *Phytochemistry* **72**, 1969-1977 (2011). https://doi.org/10.1016/j.phytochem.2011.07.001

47 Payne, R. M. E. *et al.* An NPF transporter exports a central monoterpene indole alkaloid intermediate from the vacuole. *Nature Plants* **3**, 16208 (2017). https://doi.org/10.1038/nplants.2016.208

48 Li, C. *et al.* Single-cell multi-omics in the medicinal plant *Catharanthus roseus*. *Nature Chemical Biology* **19**, 1031-1041 (2023). https://doi.org/10.1038/s41589-023-01327-0

49 Palmer, L. *et al.* In vivo characterization of key iridoid biosynthesis pathway genes in catnip (*Nepeta cataria*). *Planta* **256**, 99 (2022). https://doi.org/10.1007/s00425-022-04012-z

50 https://datadryad.org/dataset/doi:10.5061/dryad.d2547d851, therein https://datadryad.org/downloads/file_stream/2121644

51 https://ngdc.cncb.ac.cn/bioproject/browse/PRJCA004217

52 Tamura, K., Stecher, G. & Kumar, S. MEGA11: Molecular evolutionary genetics analysis version 11. *Molecular Biology and Evolution* **38**, 3022-3027 (2021). https://doi.org/10.1093/molbev/msab120

53 Zuckerkandl, E. & Pauling, L. in *Evolving Genes and Proteins* (eds Vernon Bryson & Henry J. Vogel) 97-166 (Academic Press, 1965).

54 Sievers, F. & Higgins, D. G. Clustal Omega for making accurate alignments of many protein sequences. *Protein Science* **27**, 135-145 (2018). https://doi.org/10.1002/pro.3290

55 Raisanen, M. & Karkkainen, J. Deuterium labeling of tryptamine, serotonin and their *N*-methylated metabolites using solvent exchange-reactions *Acta Chemica Scandinavica Series B-Organic Chemistry and Biochemistry* **33**, 11-14 (1979). https://doi.org/10.3891/acta.chem.scand.33b-0011

56 Zhang, W., Bah, J., Wohlfarth, A. & Franzén, J. A stereodivergent strategy for the preparation of corynantheine and ipecac alkaloids, their epimers, and analogues: efficient total synthesis of (−)-dihydrocorynantheol, (−)-dorynantheol, (−)-protoemetinol, (−)-corynantheal, (−)-protoemetine, and related natural and nonnatural compounds. *Chemistry – A European Journal* **17**, 13814-13824 (2011). https://doi.org/10.1002/chem.201102012

57 Liu, T. *et al.* Collective syntheses of corynanthe alkaloids based on a chirality-tunable structure unit. *Organic Letters* **26**, 8803-8809 (2024). https://doi.org/10.1021/acs.orglett.4c03177

58 Isaac, J. E., Robins, R. J. & Rhodes, M. J. C. Cinchoninone: NADPH oxidoreductases I and II - novel enzymes in the biosynthesis of quinoline alkaloids in *Cinchona ledgeriana*. *Phytochemistry* **26**, 393-399 (1987). https://doi.org/10.1016/S0031-9422(00)81420-X

59 Kowanko, N. & Leete, E. Biosynthesis of the cinchona alkaloids. I. The incorporation of tryptophan into quinine. *Journal of the American Chemical Society* **84**, 4919-4921 (1962). https://doi.org/10.1021/ja00883a058

60 Leete, E. & Wemple, J. N. Biosynthesis of the cinchona alkaloids. II. Incorporation of tryptophan-1-^{15}N,2-^{14}C and geraniol-3-^{14}C into quinine. *Journal of the American Chemical Society* **91**, 2698-2702 (1969). https://doi.org/10.1021/ja01038a049

61 Hay, C. A., Anderson, L. A., Phillipson, J. D., Curless, D. & Brown, R. T. In vitro cultures of *Cinchona* species. Part II. *Plant Cell, Tissue and Organ Culture* **9**, 197-206 (1987). https://doi.org/10.1007/BF00040805

62 Battersby, A. R. & Parry, R. J. Biosynthesis of the cinchona alkaloids: late stages of the pathway. *Journal of the Chemical Society D: Chemical Communications*, 31-32 (1971). https://doi.org/10.1039/C29710000031

63 Battersby, A. R. & Parry, R. J. Biosynthesis of the chinchona alkaloids: middle stages of the pathway. *Journal of the Chemical Society D: Chemical Communications*, 30-31 (1971). https://doi.org/10.1039/C29710000030

64 Battersby, A. R. *et al.* Further evidence concerning the biosynthesis of indole alkaloids and quinine. *Chemical Communications (London)*, 810-812 (1966). https://doi.org/10.1039/C19660000810

65 Battersby, A. R. *et al.* Further evidence concerning the biosynthesis of indole alkaloids and quinine. *Chemical Communications (London)*, 888-890 (1966). https://doi.org/10.1039/C19660000888

66 Leete, E. & Wemple, J. N. Biosynthesis of the cinchona alkaloids. The incorporation of geraniol-3-^{14}C into quinine. *Journal of the American Chemical Society* **88**, 4743-4744 (1966). https://doi.org/10.1021/ja00972a050

67 Inouye, H., Ueda, S. & Takeda, Y. Zur biosynthese der vinca- sowie der cinchonaalkaloide: Inkorporation des swerosids in reserpinin und chinin. *Tetrahedron Letters* **10**, 407-408 (1969). https://doi.org/10.1016/S0040-4039(01)87704-2

68 Inouye, H., Ueda, S. & Takeda, Y. Studies on monoterpene glucosides and related natural products. XIII. Incorporation of [10-^{14}C]-sweroside into gentiopicroside and the alkaloids in *Vinca* and *Cincona* plants. *Chemical & Pharmaceutical Bulletin* **19**, 587-594 (1971).

https://doi.org/10.1248/cpb.19.587

69 Battersby, A. R. & Hall, E. S. Biosynthesis of quinine from loganin. *Journal of the Chemical Society D: Chemical Communications*, 194-195 (1970). https://doi.org/10.1039/C29700000194

70 Unno, H. *et al.* Structural and mutational studies of anthocyanin malonyltransferases establish the features of BAHD enzyme catalysis. *Journal of Biological Chemistry* **282**, 15812-15822 (2007). https://doi.org/10.1074/jbc.M700638200

71 Edgar, R. C. MUSCLE: multiple sequence alignment with high accuracy and high throughput. *Nucleic Acids Research* **32**, 1792-1797 (2004). https://doi.org/10.1093/nar/gkh340

72 Edgar, R. C. Muscle5: High-accuracy alignment ensembles enable unbiased assessments of sequence homology and phylogeny. *Nature Communications* **13**, 6968 (2022). https://doi.org/10.1038/s41467-022-34630-w

73 Robert, X. & Gouet, P. Deciphering key features in protein structures with the new ENDscript server. *Nucleic Acids Research* **42**, W320-W324 (2014). https://doi.org/10.1093/nar/gku316

74 Abramson, J. *et al.* Accurate structure prediction of biomolecular interactions with AlphaFold 3. *Nature* **630**, 493-500 (2024). https://doi.org/10.1038/s41586-024-07487-w

75 Eberhardt, J., Santos-Martins, D., Tillack, A. F. & Forli, S. AutoDock Vina 1.2.0: New docking methods, expanded force field, and python bindings. *Journal of Chemical Information and Modeling* **61**, 3891-3898 (2021). https://doi.org/10.1021/acs.jcim.1c00203

76 Bugnon, M. *et al.* SwissDock 2024: major enhancements for small-molecule docking with attracting cavities and AutoDock Vina. *Nucleic Acids Research* **52**, W324-W332 (2024). https://doi.org/10.1093/nar/gkae300
