# Supplementary material for: Biosynthesis of cinchona alkaloids
Source: Nature. 2026 Mar 18;653(8113):306–14. doi: 10.1038/s41586-026-10227-x (PMC13149305; doi:10.1038/s41586-026-10227-x)
Supplement: Supplementary file 2 — Reporting Summary [file 41586_2026_10227_MOESM2_ESM.pdf]

nature portfolio

Corresponding author(s): C. Robin Buell
Sarah E. O'Connor

Last updated by author(s): Jan 27, 2026

Reporting Summary

Nature Portfolio wishes to improve the reproducibility of the work that we publish. This form provides structure for consistency and transparency in reporting. For further information on Nature Portfolio policies, see our Editorial Policies and the Editorial Policy Checklist.

Statistics

For all statistical analyses, confirm that the following items are present in the figure legend, table legend, main text, or Methods section.

n/a	Confirmed	
☐	☒	The exact sample size (n) for each experimental group/condition, given as a discrete number and unit of measurement
☐	☒	A statement on whether measurements were taken from distinct samples or whether the same sample was measured repeatedly
☐	☒	The statistical test(s) used AND whether they are one- or two-sided<br>*Only common tests should be described solely by name; describe more complex techniques in the Methods section.*
☒	☐	A description of all covariates tested
☐	☒	A description of any assumptions or corrections, such as tests of normality and adjustment for multiple comparisons
☐	☒	A full description of the statistical parameters including central tendency (e.g. means) or other basic estimates (e.g. regression coefficient) AND variation (e.g. standard deviation) or associated estimates of uncertainty (e.g. confidence intervals)
☐	☒	For null hypothesis testing, the test statistic (e.g. F, t, r) with confidence intervals, effect sizes, degrees of freedom and P value noted<br>*Give P values as exact values whenever suitable.*
☒	☐	For Bayesian analysis, information on the choice of priors and Markov chain Monte Carlo settings
☒	☐	For hierarchical and complex designs, identification of the appropriate level for tests and full reporting of outcomes
☐	☒	Estimates of effect sizes (e.g. Cohen's d, Pearson's r), indicating how they were calculated

Our web collection on statistics for biologists contains articles on many of the points above.

Software and code

Policy information about availability of computer code

Data collection	All presented data have been acquired using existing and routinely used softwares. These are mentionned in the respective parts in Methods. LC-MS data was collected with Bruker Compass qtofControl 5.2.109 / Hystar 5.1.5.1 or Thermo Scientific Xcalibur 4.7.69.37. NMR data were collected with Bruker TopSpin ver. 3.6.1. HPLC-UV data were acquired using Agilent OpenLAB CDS ChemStation 35900.
Data analysis	All data analysis was done using routinely used softwares and online available bioinformatic plateforms, as mentioned in the respective parts of the manuscript. LC-MS data were analysed using Bruker Compass DataAnalysis 6.1 and MetaboScape 2024b. LC-MS related graphs were prepared using OriginPro 9.6.0, GraphPad Prism 10.4.1, or SRplot v2025 (https://www.bioinformatics.com.cn), and were assembled in Microsoft PowerPoint 2024. Geneious Prime 2024 was used for primers design, sanger sequencing analysis, and translation of transcriptomes to proteomes. Normalization, visualization and clustering of RNA transcripts abundance were done using ClusterGVis (v0.1) and DESeq2 (v1.51.6). Phylogenetic trees were generated with MEGA11. Comparative transciptomics analysis were done with OrthoVenn3(https://orthovenn3.bioinfotoolkits.net). Protein structural models were predicted using AlphaFold 3 (https://alphafoldserver.com) and docking was done using AutoDock Vina on the SwissDock webserver (https://www.swissdock.ch) and visualised with PyMOL 2.5.3. Chemical structures were generated in ChemDraw Professional 23.1.12. Transcript reads were basecalled using Guppy (v6.3.2) and filtered with SeqKit (v0.16.1). The genome assembly was generated using Flye v2.9.1, Medaka v1.7.1 and Hapo-G v1.0. Genome annotation used RepeatMasker (v4.1.2-p1), RepeatModeler (v2.0.3), ProtExcluder (v1.2), Cutadapt (v2.10), HISAT2 (v2.1.0), Pychopper (v2.5.0), minimap2 (v2.17-r941), StringTie (v2.2.1), BRAKER2 (v2.1.6), PASA2 (v2.5.2), BLASTP (v2.12.0) and PfamScan (v1.6). Single nuclei transcriptomics data was processed with pipseeker 3.1.3, STARsolo 2.7.10b, Seurat 4.3.0.1. Microsoft Excel 2014 and OriginPro 9.6.0 were used for statistical analysis.

For manuscripts utilizing custom algorithms or software that are central to the research but not yet described in published literature, software must be made available to editors and reviewers. We strongly encourage code deposition in a community repository (e.g. GitHub). See the Nature Portfolio guidelines for submitting code & software for further information.

Data

Policy information about availability of data

All manuscripts must include a data availability statement. This statement should provide the following information, where applicable:

- Accession codes, unique identifiers, or web links for publicly available datasets
- A description of any restrictions on data availability
- For clinical datasets or third party data, please ensure that the statement adheres to our policy

Data supporting the findings of this work are available within this manuscript and its Supplementary Information files. Sequences of reported genes are included in Supplementary Information and are deposited in the National Center for Biotechnology (NCBI) GenBank under the following accession numbers: CpMAT (PX842829), CpMCC (PX842830), CpCiS (PX842831), CpCiR (PX842832), CpCiO (PX842833), CpSGD1 (PX842834), CpSGD2 (PX842835), CpSGD3 (PX842836), CpKR1 (PX842837), CpKR2 (PX842838), CpKR3 (PX842839), CpKR4 (PX842840), CpKR5 (PX842841), CpKR6 (PX842842), CpKR7 (PX842843), and CpKR8 (PX842844). C. pubescensis bulk and single nuclei RNA-seq, ONT full length cDNA, and ONT genomic DNA reads are deposited in NCBI under BioProject PRJNA1347772. Raw reads from RNA-sequencing of O. mungos leaves have been deposited in NCBI (accession: PRJNA1413194). Bulk RNA-seq of M. speciosa from a previous study was downloaded from NCBI (accession PRJNA1244102) and the transcriptome of A. thaliana used is implemented and available in OrthoVenn3 (at https://orthovenn3.bioinfotoolkits.net). Proteomics data have been deposited to the ProteomeXchange Consortium (dataset identifier PXD068683). Source data are provided with this paper. The following databases were used for gene functional annotations TAIR (v10), Swiss-Prot Plant (2015_08) and PFAM (v35.0).

Research involving human participants, their data, or biological material

Policy information about studies with human participants or human data. See also policy information about sex, gender (identity/presentation), and sexual orientation and race, ethnicity and racism.

Reporting on sex and gender	Not applicable
Reporting on race, ethnicity, or other socially relevant groupings	Not applicable
Population characteristics	Not applicable
Recruitment	Not applicable
Ethics oversight	Not applicable

Note that full information on the approval of the study protocol must also be provided in the manuscript.

Field-specific reporting

Please select the one below that is the best fit for your research. If you are not sure, read the appropriate sections before making your selection.

☒ Life sciences ☐ Behavioural & social sciences ☐ Ecological, evolutionary & environmental sciences

For a reference copy of the document with all sections, see nature.com/documents/nr-reporting-summary-flat.pdf

Life sciences study design

All studies must disclose on these points even when the disclosure is negative.

Sample size	All experiments designed to probe the function of distinct enzymes were conducted with a sample size of at least three to ensure minimal statistical power analysis: Nicotiana benthamiana pathway reconstitution experiments were done on four independent biological replicates. In vitro assays were performed in three replicates. For VIGS studies, 5 to 6 biological replicates were used. Bulk RNA-seq of Cinchona pubescens was generated from five different plant tissues (three biological replicates each) for genes discovery, and from one replicate for O. mungos which is sufficient for cross-species transcriptomics comparison as no statistics were applied. Single-nuclei RNA-seq was performed on two biological replicates, which is sufficient for minimizing technical noise and identifying consistent cell-type-specific and gene-expression profiles, as also performed in several studies (e.g., Nat. Commun. 2025, 16, 3169; Nat. 2025, 643, 582; Nat. Chem. Biol. 2023, 19, 1031).
Data exclusions	No data was excluded from the analyses.
Replication	All experiments were repeated at least once and details about biological replicates are provided in the figure legends. Pathway reconstitution experiments were conducted on four biological replicates that correspond to four independent individual plants. All attempts of replication were successful. In vitro assays were performed as three technical replicates from the same enzyme purifications. Replications with different enzyme purifications were successful. VIGS experiments were successfully replicated twice, once with sterile plantlets and also using non-sterile 4-month-old plants grown on sand.
Randomization	For all LC-MS analyses, samples were loaded randomly on the mass spectrometer. To reduce variance, the order of injections into the LC-MS system was randomized, using the function RAND in Microsoft Excel. Plants and plantlets for gene expression profiling, gene heterologous expression, and VIGS assays were grown randomly in the growth chamber. For all the other experiments, randomization was not relevant.

Blinding	Blinding was not relevant for this study; characterization of pathway genes and enzymes requires insight into the experimental conditions and characteristics of the samples.

Reporting for specific materials, systems and methods

We require information from authors about some types of materials, experimental systems and methods used in many studies. Here, indicate whether each material, system or method listed is relevant to your study. If you are not sure if a list item applies to your research, read the appropriate section before selecting a response.

Materials & experimental systems

n/a	Involved in the study	
☒	☐	Antibodies
☒	☐	Eukaryotic cell lines
☒	☐	Palaeontology and archaeology
☒	☐	Animals and other organisms
☒	☐	Clinical data
☒	☐	Dual use research of concern
☐	☒	Plants

Methods

n/a	Involved in the study	
☒	☐	ChIP-seq
☒	☐	Flow cytometry
☒	☐	MRI-based neuroimaging

Dual use research of concern

Policy information about dual use research of concern

Hazards

Could the accidental, deliberate or reckless misuse of agents or technologies generated in the work, or the application of information presented in the manuscript, pose a threat to:

No	Yes	
☒	☐	Public health
☒	☐	National security
☒	☐	Crops and/or livestock
☒	☐	Ecosystems
☒	☐	Any other significant area

Experiments of concern

Does the work involve any of these experiments of concern:

No	Yes	
☒	☐	Demonstrate how to render a vaccine ineffective
☒	☐	Confer resistance to therapeutically useful antibiotics or antiviral agents
☒	☐	Enhance the virulence of a pathogen or render a nonpathogen virulent
☒	☐	Increase transmissibility of a pathogen
☒	☐	Alter the host range of a pathogen
☒	☐	Enable evasion of diagnostic/detection modalities
☒	☐	Enable the weaponization of a biological agent or toxin
☒	☐	Any other potentially harmful combination of experiments and agents

Plants

Seed stocks	N. benthamiana and A. thaliana seeds were obtained from seed stocks maintained by the greenhouse team at Max Planck Institute for Chemical Ecology, Jena. C. pubescens and Kratom seeds were obtained from Edinburgh botanical garden and the University of Florida, respectively. O. mungos was obtained as a plantlet from the Palmengarten botanical garden, as described in Plant Materials.
Novel plant genotypes	No plant stable transformation was performed in this study. Genes heterelogous expression in N. benthamiana was carried out transiently through agroinfiltration of young leafs, as described in the methods paragraph "Transient gene expression in N. benthamiana". Virus-induced gene silencing in C. pubescens was also transient, as described in the methods section "Virus induced gene silencing (VIGS) in C. pubescens".
Authentication	Transient transformation of N. benthamiana through leaf agroinfiltration was done as described in the methods paragraph "Transient gene expression in N. benthamiana". Virus-induced gene silencing in C. pubescenas was also transient as described in the Methods paragraph "Virus induced gene silencing (VIGS) in C. pubescens". No plant stable transformation was carried out.
