# Supplementary material for: Biosynthesis of cinchona alkaloids
Source: Nature. 2026 Mar 18;653(8113):306–14. doi: 10.1038/s41586-026-10227-x (PMC13149305; doi:10.1038/s41586-026-10227-x)
Supplement: Supplementary file 6 — Peer Review File [file 41586_2026_10227_MOESM6_ESM.pdf]

nature portfolio

Peer Review File

Biosynthesis of Cinchona Alkaloids

Corresponding Author: Professor Sarah O'Connor

Any redactions in this file are there to maintain patient confidentiality, the confidentiality of unpublished data, or to remove third-party material.

This file contains all reviewer reports in order by version, followed by all author rebuttals in order by version.

Version 1:

Reviewer comments:

Referee #1

(Remarks to the Author)
This study uncovered the biosynthesis of the quinoline-quinuclidine scaffold, the characteristic core of the Cinchona alkaloids, using combination methods, such as isotopic labeling, gene silencing, single nuclei RNA sequencing, and comparative transcriptomics. It’s a very solid, inspiring, and interesting story.
Several parts not entirely clear and needs certain clarification:
1. For Fig. 2, it’s unclear what “mock” refers to. Is it the one without feeding, or the one fed with 11 instead of 11b? This distinction is important because both serve as critical negative controls for comparison. Please label these conditions more clearly in the figure or clarify their definitions in the caption. Additionally, from the current figure, it is also unclear whether both 11 and 11b were fed to C. pubescens, or if only 11b was used. Please clarify this as well to avoid confusions.
For example, in Figure 2e, was compound 12 fed only to C. pubescens roots and was converted to 1b, 2b, 10b, and 13b? Does this indicate that these conversions were not detected in leaf or stem tissues? Please clarify whether this transformation was tissue-specific or whether other tissues were not tested.
Similarly, in the sentence “we fractionated the isotopically labeled form of this compound from C. pubescens leaf that had been fed with 11b, which we then re-fed to fresh C. pubescens tissues,” does this mean that the conversion from 11b to 12b was detected only in leaf tissue, or more efficient in leaves? If so, does this suggest that the conversion efficiencies—from 11b → 12b and from 12b → (1b, 2b, 10b, 13b)—vary among different plant tissues?
2. The paragraph starting with “Feeding of d5-corynantheol (11b) and d5-cinchonium (12b) to C. pubescens tissues also led to the formation of a compound with a molecular mass (m/z 295.1805) and MS/MS fragmentation pattern similar to commercially available cinchonamine (10) (an alcohol, m/z 297.1961), consistent with the structure of the long-predicted aldehyde precursor cinchonaminal (7).” is confusing. It is not clear how compound 7 becomes involved in this context. A comparison of the MS/MS data and a small biosynthetic schematic would be very helpful to clarify the logic behind identifying 7 here.
Additionally, the sentence referencing “MS/MS analyses of purified 7” is ambiguous. Is this referring to the newly observed compound with m/z 295.1805? Or is it referring to an independently purified standard of 7? Please clarify.
Overall, the paragraph would benefit from rewriting to improve clarity, particularly in explaining the flow from the feeding experiments to the detection of the m/z 295.1805 species, how this connects to compound 7, and how isolating and characterizing this putative intermediate (7 or 13?) is important.
3. In the section “Conversion of corynantheal to malonyl-corynantheol,” the evidence supporting malonyl-corynantheol as an intermediate in the biosynthetic pathway is not entirely convincing.
In the VIGS experiment, the levels of downstream alkaloids were not reduced, suggesting that the malonylation step may not be essential for flux through the pathway. While the data clearly show that CpMAT can catalyze the malonylation of corynantheal, they do not definitively demonstrate that this reaction is functionally required in the native pathway.
If possible, including a control without CpMAT in Fig. 4 or Extended Data Fig. 4 for the Nicotiana benthamiana in vitro assay, where all other pathway enzymes are present but CpMAT is omitted, leading to accumulation of compound 11 but not downstream products such as 14 or 12, would make it much clearer that CpMAT is essential for the biosynthesis. This addition would substantially strengthen the argument that malonyl-corynantheol is an essential pathway intermediate.
Minor cosmetic issues:
1. Fig. 2, panel d and f, “Incorporation of 11b into a previously unknown compound (12)” in panel d and “Incorporation” in panel f are boxed, which should be removed.
2. It’ll be nice if the author can include the relationship between 6 and 11 in fig. 2a, to help the audience follow the logic.

3. Extended Data Fig. 1, please remove the borderline of the plant pictures in panel e.
4. Extended Data Fig. 3, please remove the borderline of the labels in panel f and g.
5. fig 3, panel b has an unnecessary line that should be removed.

Referee #2

(Remarks to the Author)
I co-reviewed this manuscript with one of the reviewers who provided the listed reports.

Referee #3

(Remarks to the Author)
This manuscript represents a huge effort and presents massive amounts of data to demonstrate delineation of the metabolic network in plants that produces cinchona alkaloids, a very important group of medicinal compounds that includes well-known antimalarial quinine. The latter compound has been known, as indicated in the manuscript, for well over 100 years, yet its biosynthesis has remained elusive, until now. Dr. O'Connor's team has done an excellent job of identifying the genes, and providing conclusive evidence to that fact, that are involved in the biosynthesis of this and related compounds. This manuscript is a tour de force that could easily have been broken out into 5 - 6 separate, smaller manuscripts. Indeed, the 120+ page "supplementary information" document/data section was quite an undertaking to read through as it was so comprehensive and complete. It was really like reading 5 separate manuscripts, all placed together one after the other. I understand why the authors did this, to provide clear demonstration, in one single publication, of the elucidation of this biochemical pathway/network. This will surely lead to a impact for the article. It might be easier to ready as a series of articles, but it works well as is because the whole story is presented in one complete narrative, and this seems to follow the pattern of many articles published in Nature.
To answer the questions requested in the review process:
A. Summary: This team clearly outlined the metabolic pathway/network involved in cinchona alkaloid biosynthesis, and did so using all appropriate tools available today. The summary and conclusion sections of the manuscript outline this well.
B. The question of originality/novelty is perhaps a tricky one for something like this manuscript. The approach used to elucidate the pathway is the same approach that everyone working on plant metabolism (particularly medicinal plant metabolism) follows, using a combination of modern multi-omic analyses and more traditional basic biochemistry and organic synthesis and medicinal chemistry and analytical chemistry to verify structures and then transgenic plant production to generate and validate steps in the biochemical network using other species (specifically, N. benthamiana), with specific experiments as appropriate for the particular pathway in question. So, the general approach is pretty standard now. What makes this article particularly strong is that this approach was performed very well – and also extensively – in this case. Three things stand out to me as particularly novel about this work. The first is the identification of two members of the BAHD family of enzymes, CpMAT and CpMCC, as key enzymes mediating malonylation and subsequent demalonylative cyclization reactions – opening up new functions for members of the BAHD family of enzymes in plants. The second is the use of just the right comparative species, particularly Mitragyna speciosa, to aid in novel gene function identification. This team had some novel insights into which specific experiments should be performed utilizing these other species, which other researchers have missed, that allowed them to conclusively demonstrate how the pathway/network functions and how it can be utilized for generation of novel compounds when engineered and/or introduced into other systems. Picking the right comparisons to make, which is not a trivial task, is a strength of this work and this team. The third thing that stood out to me as particularly novel, more so than the other two listed just above, was the demonstration of the ability of the enzymes in this network to utilize various halogenated precursors with relatively high efficiency, which leads to the ability to generate novel analogs of the natural cinchona alkaloids, opening the door for novel drug development. This was clearly emphasized in the paper and may be viewed down the road as the most important part of the work presented.
C. Data & methodology: I had no concern with the approach or the results generated/data presented, except that some of the figures in the supplementary information document, particularly some of the chemical structures, appeared to be rather poor resolution, at least in the version that I had access to. This is not a problem for the work and conclusions and is potentially a result of the document generation process generating the pdf for review. I bring this up so that the authors are aware of this issue, so they can make sure that a final publication has the highest quality figures. I looked at the chemical structures presented and all appeared to be drawn correctly, and all data supporting the structures identified appeared correct to me. I didn’t notice anything that appeared to be amiss.
D. Statistics were used appropriately for this type of investigation (e.g., “$p < 0.0001$, Tukey test”). No concerns here.
E. The conclusions appeared to me to be clearly justified. I have to say that this is one of the most complete and convincing pieces of work I have read in a long time. I have been impressed by Dr. O’Connor’s work in general (having known her work for a long time now), but this paper really raised the bar. I looked for errors, as a reviewer is supposed to do, and maybe there are some copy editing issues that I missed (again because the work is so long), but there were no large or even small errors that I noticed.
F. I would not suggest adding more data to this work. What is presented is sufficient and clearly justified as being important and relevant to the conclusions. I didn’t see a need for a major revision.
G. Regarding references cited: There could be a large number of additional references added to the list, but I understand space limitations and those that were included seem to be well suited.
H. The text of the manuscript was very well written and was clear to me. I think that anyone in a field closely aligned to the work presented will find the article to be highly approachable. I think that although there is a lot of technical language specific to biochemistry/metabolism studies used throughout, it should still be understandable to a broad audience. The figures are very nice to look at. I think that good looking figures with the right use of color, which this article mostly does, really help convey the message. I mentioned above the potential issue of figure resolution in the supplementary information

document. I also found something to consider for potential minor revision. The authors use, as a way to highlight important points in the figures, the colors red and green. Since about 1 in 12 males are red-green color blind, such potential readers of the article may miss the points emphasized. The information is mostly there in other ways – use of specific words – but not always. For example, in Fig. 1 "previously reported catalytic activity" is pointed out as being colored red in the figure. For a color blind person, there would be no way to identify what this activity is. This should be addressed/made more accessible.
In summary, the manuscript was a pleasure read, even if taking so long to do so as it required. It was nice to see how this type of effort can be carried out in a manner leading to clear delineation of a novel metabolic pathway in such a short time (from what I could tell). This paper should have a significant impact on several fields, from metabolism studies, to drug discovery and novel treatment development.
Signed: David Gang, Institute of Biological Chemistry, Washington State University

Referee #4

(Remarks to the Author)
Lombe et al., conducted an impressive series of experiments that integrated multiple approaches and discovered several novel enzymes involved in biosynthesis of Cinchona alkaloids, which include quinine, an anti-malaria drug and a key ingredient of tonic water. The biosynthetic pathway and responsible enzymes of Cinchona alkaloids have been enigmatic for many decades, and this study finally revealed their nature and identity. The authors found that the distinctive scaffold of Cinchona alkaloids is formed via a novel quaternary amine intermediate. The discovered enzymes now enable biotechnological production of diverse Cinchona alkaloids, including unnatural halogenated derivatives, which may process new biological activity or properties. Therefore, this study provides exciting new discoveries for those working on plant natural product chemistry and biochemistry as well as broader biotechnology and pharmaceutical sciences.

Major comments:

The overall conclusion of the study is generally supported by a large body of experimental data. However, a few areas of data presentation can be further improved.

1. Malonyl-corynantheol (14) was found to be a novel functional intermediate of the pathway, which is supported by multiple data, such as the conversion of labeled malonyl-corynantheol (14) into cinchonium (12) in the feeding experiment (Supp Fig 14) and in vitro substrate specificity testing of CpMCC (Supp Fig 16b). However, it remains unclear if other intermediates are potentially involved. The Extended Fig. 1b shows the detection of a malonylated derivative metabolized from corynantheol (11) upon expression of CpMAT in Nicotiana leaves. However, it is unclear how this was found and if there were other new peaks produced, such as in the TIC trace. Also, does CpMAT have acetyltransferase activity, given that CpMAT is closely related to other acetyltransferases (Supp Fig 17)? Similarly, when CpMCC was downregulated by VIGS in Cinchona plantlet (Supplementary Fig. 16), were 14 and its derivatives (14', 14a, 14a') the major metabolites accumulated upon VIGS (upon inspection of TIC trace)? As malonyl-corynantheol (14) could not be detected in Cinchona plant tissues, additional descriptions will be helpful to evaluate if the major activity of CpMAT is indeed malonylation.

2. Fig 3f-g: it is interesting that N. benthamiana extract inhibits CpKR4 activity, which unfortunately prevents the production of the final products, dihydrocinchonidine and dihydroquinine, through heterologous pathway reconstruction in N. benthamiana. Does it inhibit the reaction or protein stability (and degrade CpKR4)? If it affects protein stability, adding a tag (e.g., YFP) could help stabilize the CpKR4 protein. Does the tissue extract of Cinchona or other plants (e.g., Arabidopsis) lack this inhibitory effect? These information will guide future metabolic engineering strategies, including plant chassis selection, for Cinchona alkaloid production.

3. While I acknowledge that a huge amount of data is already presented, some key information were still missing. In Page 11, how were the CpCiR candidates obtained for the screening of alcohol dehydrogenase that convers cinchonaminal to cinchonamine? In page 13: what were the 60 reductase candidates that were tested for the NADPH-dependent oxidoreductase, which resulted in the discovery of CpKR4? Seven additional CpKR candidates are also strongly expressed in roots and/or stem (Supp Fig. 31); were they tested for activity in vitro? Please provide these descriptions, so that readers know what exactly have been tested in this study.

4. Fig 4c: It is exciting that a series of halogenated tryptamine substrates can be incorporated into the corresponding dihydrocinchoninone and dihydrocinchonidinone analogs. It will be helpful if the graph of Fig 4c includes the results of Fig 4a with the natural tryptamine substrate, so that relatively efficiency can be compared and discussed.

5. The conclusion of the main text should discuss remaining knowledge and technical gaps, such as inactivity of CpKR4 in N. benthamiana and the missing enzyme responsible for the formation of dehydro congeners, corynantheal (6) and methoxylated analog (6a).

Minor comments

6. Page 2: Cite Supplemental Figure 1 in addition to Fig. 1.

7. Page 4: The structural determination of the novel intermediate cinchonium (12) is crucial data (Supplementary Fig. 46-51) supporting one of the major findings of the study. However, they are difficult to find them. I would be best if these data are

directly cited in the main text (e.g., line 9-11 of page 4).

8. Fig. 3f: Please provide the list of 20 candidates as an associated table. (Also, the main text in page 9 is citing Fig. 3e, instead of 3f).

9. Table S3: It will be best to provide in a fasta format with more detailed gene names, so that people can easily copy the entire table to carry out various analyses.

10. Supplementary Fig. 8: Please cite figure numbers of the underlying NMR spectra in the figure legend.

11. Fig 4b: Malonyl-corynantheol (14) was detected while the main text says otherwise (page 16, bottom). Please clarify.

Version 2:

Reviewer comments:

Referee #4

(Remarks to the Author)
The revised manuscript fully addressed all comments from the previous review by providing additional data and supplementary figures and revising texts. This will be a landmark work that opens future explorations to uncover diverse specialized metabolic pathways of Cinchona alkaloids having critical societal uses and values.

Open Access This Peer Review File is licensed under a Creative Commons Attribution 4.0 International License, which permits use, sharing, adaptation, distribution and reproduction in any medium or format, as long as you give appropriate credit to the original author(s) and the source, provide a link to the Creative Commons license, and indicate if changes were made.
In cases where reviewers are anonymous, credit should be given to 'Anonymous Referee' and the source.
The images or other third party material in this Peer Review File are included in the article’s Creative Commons license, unless indicated otherwise in a credit line to the material. If material is not included in the article’s Creative Commons license and your intended use is not permitted by statutory regulation or exceeds the permitted use, you will need to obtain permission directly from the copyright holder.
To view a copy of this license, visit https://creativecommons.org/licenses/by/4.0/

We are very grateful for the constructive and positive review of our manuscript (2025-10-26998A). Please find below our point-by-point responses.

Please note that we have removed the embedded figures in the main text file as requested by the Editor. We have included a version of the manuscript with figures embedded as a related article file in case it makes reviewing easier.

Referee #1

This study uncovered the biosynthesis of the quinoline-quinuclidine scaffold, the characteristic core of the Cinchona alkaloids, using combination methods, such as isotopic labeling, gene silencing, single nuclei RNA sequencing, and comparative transcriptomics. It's a very solid, inspiring, and interesting story. Several parts not entirely clear and needs certain clarification.

Response. We thank the referee for the positive review.

*1. For Fig. 2, it's unclear what "mock" refers to. Is it the one without feeding, or the one fed with **11** instead of **11b**? This distinction is important because both serve as critical negative controls for comparison. Please label these conditions more clearly in the figure or clarify their definitions in the caption. Additionally, from the current figure, it is also unclear whether both **11** and **11b** were fed to C. pubescens, or if only **11b** was used. Please clarify this as well to avoid confusions. For example, in Figure 2e, was compound **12** fed only to C. pubescens roots and was converted to **1b**, **2b**, **10b**, and **13b**? Does this indicate that these conversions were not detected in leaf or stem tissues? Please clarify whether this transformation was tissue-specific or whether other tissues were not tested. Similarly, in the sentence "we fractionated the isotopically labeled form of this compound from C. pubescens leaf that had been fed with **11b**, which we then re-fed to fresh C. pubescens tissues," does this mean that the conversion from **11b** to **12b** was detected only in leaf tissue, or more efficient in leaves? If so, does this suggest that the conversion efficiencies—from **11b** → **12b** and from **12b** → (**1b**, **2b**, **10b**, **13b**)—vary among different plant tissues?*

Response. The "mock" was defined in the Figure caption, as "samples fed with water instead of the labeled compound (control group)". We have now highlighted this definition in our revised version. We fed only isotopically labeled substrates which is advantageous as the isotopic incorporation into downstream products can reliably and efficiently be tracked even in complex mixtures by LC-MS. We have made the following modification in Figure 2a and corresponding caption, to make it more clear on what exactly was fed to *C. pubescens* tissues: In Figure 2a, the text "Feeding *C. pubescens* tissues (root, stem and leaf)" was changed to "Feeding **11b to** *C. pubescens* tissues (root, stem and leaf)". The caption for this panel has been changed to "Schema illustrating the natural conversion of corynantheal (**6**) into the corresponding alcohol congener corynantheol (**11**), along with the structure of the synthetic isotopically labeled analog d_5-

corynantheol (**11b**) that was used to feed tissues from *C. pubescens* plantlets". Please note that we have also modified slightly this Figure panel and caption according to another request/suggestion from the same referee (i.e., "*It'll be nice if the author can include the relationship between **6** and **11** in fig. 2a, to help the audience follow the logic*").

All tissues were tested and were all biosynthetically active, which matches with the detection of the alkaloids in these tissues. The tissues showed varied conversion efficiency, with roots being most efficient in feeding assays (see Supplementary Figure 4b), which is why we use roots (image or illustration) in the Figure 2. We have changed the sentence "*we fractionated the isotopically labeled form of this compound from C. pubescens leaf that had been fed with **11b**, which we then re-fed to fresh C. pubescens tissues*" to "we fractionated the isotopically labeled form of this compound from *C. pubescens* tissues that had been fed with **11b**, which we then re-fed to fresh *C. pubescens* tissues".

*2. The paragraph starting with "Feeding of d_5-*corynantheol *(**11b**) and d_5-*cinchonium *(**12b**) to C. pubescens tissues also led to the formation of a compound with a molecular mass (m/z 295.1805) and MS/MS fragmentation pattern similar to commercially available cinchonamine (**10**) (an alcohol, m/z 297.1961), consistent with the structure of the long-predicted aldehyde precursor cinchonaminal (**7**)." is confusing. It is not clear how compound **7** becomes involved in this context. A comparison of the MS/MS data and a small biosynthetic schematic would be very helpful to clarify the logic behind identifying **7** here. Additionally, the sentence referencing "MS/MS analyses of purified **7**" is ambiguous. Is this referring to the newly observed compound with m/z 295.1805? Or is it referring to an independently purified standard of **7**? Please clarify. Overall, the paragraph would benefit from rewriting to improve clarity, particularly in explaining the flow from the feeding experiments to the detection of the m/z 295.1805 species, how this connects to compound **7**, and how isolating and characterizing this putative intermediate (**7** or **13**?) is important.*

Response. We have modified this paragraph to make it clearer. **Early version**: "Feeding of d_5-corynantheol (**11b**) and d_5-cinchonium (**12b**) to *C. pubescens* tissues also led to the formation of a compound with a molecular mass (*m/z* 295.1805) and MS/MS fragmentation pattern similar to commercially available cinchonamine (**10**) (an alcohol, *m/z* 297.1961), consistent with the structure of the long-predicted[25] aldehyde precursor cinchonaminal (**7**) (Fig. 2f, Supplementary Fig. 6). As with cinchonium, an isotope-labeled derivative of this target was partially purified from fed plant tissues, tested as an intermediate by re-feeding to *C. pubescens*, and confirmed by LC-MS analysis to be incorporated into **1** and **2** (Fig. 2g). NMR and MS/MS analyses of purified **7** established that this compound predominantly exists in the hemiaminal cyclized form **13** (herein referred to as cyclocinchonaminal, Fig. 2f), marking the first time that this putative intermediate has actually been isolated and structurally characterized (Supplementary Fig. 7)."

New version: "Feeding of d_5-corynantheol (**11b**) and d_5-cinchonium (**12b**) to *C. pubescens* tissues also led to the detection of a native compound with a molecular mass (*m/z* 295.1805) and MS/MS fragmentation pattern similar to commercially available cinchonamine (**10**) (an alcohol,

m/z 297.1961), suggesting that the detected metabolite was the long-predicted[25] aldehyde intermediate cinchonaminal (**7**) (Fig 1, Fig. 2f, Supplementary Fig. 6). As with cinchonium (**12**), an isotope-labeled derivative of this target (putative cinchonaminal, **7**) was partially purified from fed plant tissues, tested as an intermediate by re-feeding to *C. pubescens*, and confirmed by LC-MS analysis to be incorporated into **1** and **2** (Fig. 2g). Isolation, semi-synthesis, and NMR and MS/MS analyses confirmed the target compound to be the predicted cinchonaminal an showed that it predominantly exists in the hemiaminal cyclized form **13** (herein referred to as cyclocinchonaminal, Fig. 2f). This marks the first time that this intermediate has actually been isolated and structurally characterized (Supplementary Fig. 7)."

Moreover, we have modified Supplementary Fig. 6, by adding a panel showing MS/MS spectra of the two compounds (cinchonamine and cinchonaminal), as suggested.

3. *In the section "Conversion of corynantheal to malonyl-corynantheol," the evidence supporting malonyl-corynantheol as an intermediate in the biosynthetic pathway is not entirely convincing. In the VIGS experiment, the levels of downstream alkaloids were not reduced, suggesting that the malonylation step may not be essential for flux through the pathway. While the data clearly show that CpMAT can catalyze the malonylation of corynantheal, they do not definitively demonstrate that this reaction is functionally required in the native pathway. If possible, including a control without CpMAT in Fig. 4 or Extended Data Fig. 4 for the Nicotiana benthamiana in vitro assay, where all other pathway enzymes are present but CpMAT is omitted, leading to accumulation of compound* ***11*** *but not downstream products such as* ***14*** *or* ***12****, would make it much clearer that CpMAT is essential for the biosynthesis. This addition would substantially strengthen the argument that malonyl-corynantheol is an essential pathway intermediate.*

Response. We appreciate this important point, but, however, we believe that it was already addressed. As shown in Supplementary Fig. 18, omitting *Cp*MAT during pathway reconstruction in *N. benthamiana* leads to the accumulation of corynantheol (**11**) and virtually no downstream products, a result that clearly indicates both that the malonylation is essential for the cyclization and that *Cp*MCC lacks the transferase activity. We believe that this is the key result that reviewer was asking for. Notably, in *Cinchona* plants, corynantheol and analogs (dihydro and methoxylated) only occur at trace levels (which might be a reason why these compounds had not been considered or hypothesized as pathway intermediates). Thus, the accumulation of **11** both in *Cp*MAT VIGS assays and in reconstruction experiments when *Cp*MAT is omitted, together with the feeding assays with isotopically labelled substrates, strongly show that *Cp*MAT is an on-pathway enzyme. The fact that levels of downstream alkaloids are not decreased in *Cp*MAT VIGS experiments is most probably due to the fact the immediate pathway downstream intermediate cinchonium (**12**) is the most dominant metabolite in the Cinchona leaves (10- to 30-time more abundant than other downstream alkaloids, see Supplementary Fig. 11, or in Extended Data Fig. 1g see the dominant peak at ca. 3.1 min for **12** and the peak at 3.5 min for the dihydro analog **12′**). As already mentioned in the main text, the huge accumulation of **12** and analogs would compensate for the transient interruption of the metabolic flux from VIGS assays.

Minor cosmetic issues:
1. Fig. 2, panel d and f, "Incorporation of ***11b*** *into a previously unknown compound (****12****)" in panel d and "Incorporation" in panel f are boxed, which should be removed.*
2. It'll be nice if the author can include the relationship between ***6*** *and* ***11*** *in fig. 2a, to help the audience follow the logic.*
3. Extended Data Fig. 1, please remove the borderline of the plant pictures in panel e.
4. Extended Data Fig. 3, please remove the borderline of the labels in panel f and g.
5. fig 3, panel b has an unnecessary line that should be removed.

Response. We thank the referee for the remarks and suggestions. In Figure 2a, we have added a scheme illustrating the relationship between **6** and **11**, as already mentioned above. During the conversion of our files to pdf format, unwanted frame lines unfortunately appeared and the quality of some figures dropped. We have made all the changes and will pay more attention to figures prior to the final publication.

Referee #2:

I co-reviewed this manuscript with one of the reviewers who provided the listed reports.

Response. We are grateful for the effort in reviewing our work.

Referee #3:

This manuscript represents a huge effort and presents massive amounts of data to demonstrate delineation of the metabolic network in plants that produces cinchona alkaloids, a very important group of medicinal compounds that includes well-known antimalarial quinine. The latter compound has been known, as indicated in the manuscript, for well over 100 years, yet its biosynthesis has remained elusive, until now. Dr. O'Connor's team has done an excellent job of identifying the genes, and providing conclusive evidence to that fact, that are involved in the biosynthesis of this and related compounds. This manuscript is a tour de force that could easily have been broken out into 5 - 6 separate, smaller manuscripts. Indeed, the 120+ page "supplementary information" document/data section was quite an undertaking to read through as it was so comprehensive and complete. It was really like reading 5 separate manuscripts, all placed together one after the other. I understand why the authors did this, to provide clear demonstration, in one single publication, of the elucidation of this biochemical pathway/network. This will surely lead to a impact for the article. It might be easier to ready as a series of articles, but it works well as is because the whole story is presented in one complete narrative, and this seems to follow the pattern of many articles published in Nature.

To answer the questions requested in the review process:

A. Summary: This team clearly outlined the metabolic pathway/network involved in cinchona alkaloid biosynthesis, and did so using all appropriate tools available today. The summary and conclusion sections of the manuscript outline this well.

B. The question of originality/novelty is perhaps a tricky one for something like this manuscript. The approach used to elucidate the pathway is the same approach that everyone working on plant metabolism (particularly medicinal plant metabolism) follows, using a combination of modern multi-omic analyses and more traditional basic biochemistry and organic synthesis and medicinal chemistry and analytical chemistry to verify structures and then transgenic plant production to generate and validate steps in the biochemical network using other species (specifically, N. benthamiana), with specific experiments as appropriate for the particular pathway in question. So, the general approach is pretty standard now. What makes this article particularly strong is that this approach was performed very well – and also extensively – in this case. Three things stand out to me as particularly novel about this work. The first is the identification of two members of the BAHD family of enzymes, CpMAT and CpMCC, as key enzymes mediating malonylation and subsequent demalonylative cyclization reactions – opening up new functions for members of the BAHD family of enzymes in plants. The second is the use of just the right comparative species, particularly Mitragyna speciosa, to aid in novel gene function identification. This team had some novel insights into which specific experiments should be performed utilizing these other species, which other researchers have missed, that allowed them to conclusively demonstrate how the pathway/network functions and how it can be utilized for generation of novel compounds when engineered and/or introduced into other systems. Picking the right comparisons to make, which is not a trivial task, is a strength of this work and this team. The third thing that stood out to me as particularly novel, more so than the other two listed just above, was the demonstration of the ability of the enzymes in this network to utilize various halogenated precursors with relatively high efficiency, which leads to the ability to generate novel analogs of the natural cinchona alkaloids, opening the door for novel drug development. This was clearly emphasized in the paper and may be viewed down the road as the most important part of the work presented.

C. Data & methodology: I had no concern with the approach or the results generated/data presented, except that some of the figures in the supplementary information document, particularly some of the chemical structures, appeared to be rather poor resolution, at least in the version that I had access to. This is not a problem for the work and conclusions and is potentially a result of the document generation process generating the pdf for review. I bring this up so that the authors are aware of this issue, so they can make sure that a final publication has the highest quality figures. I looked at the chemical structures presented and all appeared to be drawn correctly, and all data supporting the structures identified appeared correct to me. I didn't notice anything that appeared to be amiss.

D. Statistics were used appropriately for this type of investigation (e.g., "$p < 0.0001$, Tukey test"). No concerns here.

E. The conclusions appeared to me to be clearly justified. I have to say that this is one of the most complete and convincing pieces of work I have read in a long time. I have been impressed

by Dr. O'Connor's work in general (having known her work for a long time now), but this paper really raised the bar. I looked for errors, as a reviewer is supposed to do, and maybe there are some copy editing issues that I missed (again because the work is so long), but there were no large or even small errors that I noticed.

F. I would not suggest adding more data to this work. What is presented is sufficient and clearly justified as being important and relevant to the conclusions. I didn't see a need for a major revision.

G. Regarding references cited: There could be a large number of additional references added to the list, but I understand space limitations and those that were included seem to be well suited.

H. The text of the manuscript was very well written and was clear to me. I think that anyone in a field closely aligned to the work presented will find the article to be highly approachable. I think that although there is a lot of technical language specific to biochemistry/metabolism studies used throughout, it should still be understandable to a broad audience. The figures are very nice to look at. I think that good looking figures with the right use of color, which this article mostly does, really help convey the message. I mentioned above the potential issue of figure resolution in the supplementary information document. I also found something to consider for potential minor revision. The authors use, as a way to highlight important points in the figures, the colors red and green. Since about 1 in 12 males are red-green color blind, such potential readers of the article may miss the points emphasized. The information is mostly there in other ways – use of specific words – but not always. For example, in Fig. 1 "previously reported catalytic activity" is pointed out as being colored red in the figure. For a color blind person, there would be no way to identify what this activity is. This should be addressed/made more accessible.

In summary, the manuscript was a pleasure read, even if taking so long to do so as it required. It was nice to see how this type of effort can be carried out in a manner leading to clear delineation of a novel metabolic pathway in such a short time (from what I could tell). This paper should have a significant impact on several fields, from metabolism studies, to drug discovery and novel treatment development.

Signed: David Gang, Institute of Biological Chemistry, Washington State University

Response. We are very grateful to Dr. D. Gang for reviewing our work and all the comments. Regarding the colors, we have made changes in several Figures (see e.g., Figure 2 and 4, and Extended Data Figure 1, 2 and 3) to address this important aspect for color-blind people. We are also open to further color change suggestions from the editorial or publishing services, which likely will be addressed prior to publication. Figure quality dropped during the file conversion to pdf format. We will be pay extra care prior to final publication.

Referee #4:

Lombe et al., conducted an impressive series of experiments that integrated multiple approaches and discovered several novel enzymes involved in biosynthesis of Cinchona alkaloids, which

include quinine, an anti-malaria drug and a key ingredient of tonic water. The biosynthetic pathway and responsible enzymes of Cinchona alkaloids have been enigmatic for many decades, and this study finally revealed their nature and identity. The authors found that the distinctive scaffold of Cinchona alkaloids is formed via a novel quaternary amine intermediate. The discovered enzymes now enable biotechnological production of diverse Cinchona alkaloids, including unnatural halogenated derivatives, which may process new biological activity or properties. Therefore, this study provides exciting new discoveries for those working on plant natural product chemistry and biochemistry as well as broader biotechnology and pharmaceutical sciences.

Response. We are thankful for the overall positive review.

Major comments:

The overall conclusion of the study is generally supported by a large body of experimental data. However, a few areas of data presentation can be further improved.

*1. Malonyl-corynantheol (**14**) was found to be a novel functional intermediate of the pathway, which is supported by multiple data, such as the conversion of labeled malonyl-corynantheol (**14**) into cinchonium (**12**) in the feeding experiment (Supp Fig 14) and in vitro substrate specificity testing of CpMCC (Supp Fig 16b). However, it remains unclear if other intermediates are potentially involved. The Extended Fig. 1b shows the detection of a malonylated derivative metabolized from corynantheol (**11**) upon expression of CpMAT in Nicotiana leaves. However, it is unclear how this was found and if there were other new peaks produced, such as in the TIC trace. Also, does CpMAT have acetyltransferase activity, given that CpMAT is closely related to other acetyltransferases (Supp Fig 17)? Similarly, when CpMCC was downregulated by VIGS in Cinchona plantlet (Supplementary Fig. 16), were **14** and its derivatives (**14'**, **14a**, **14a'**) the major metabolites accumulated upon VIGS (upon inspection of TIC trace)? As malonyl-corynantheol (**14**) could not be detected in Cinchona plant tissues, additional descriptions will be helpful to evaluate if the major activity of CpMAT is indeed malonylation.*

Response. When transferase candidates were expressed in *N. benthamiana*, either in a batch of several genes or individually, and the substrate corynantheol (**11**) infiltrated, we observed the consumption of **11** (reflected by disappearance or reduced intensity of the peak of **11**) concomitant to the appearance of a new dominant peak corresponding to **14** only in *N. benthamiana* plants expressing *Cp*MAT. This was clearly the major product that formed upon infiltration of substrate and enzyme. In *in vitro* assays with purified recombinant *Cp*MAT, and corynantheol and malonyl-CoA, the TIC shows only one new peak, which corresponds to malonyl-corynantheol (**14**). Similar results were obtained when *Cp*MAT was expressed with other downstream (STR, SGD, DCS and DCE) and strictosidine or tryptamine and secologanin were used as substrates, leading to the formation of malonyl-dihydrocorynantheol (**14′**) as the new dominant compound. We have created a new panel in Supplementary Fig. 8 (panel d) where we show the TIC traces for these reactions *in vitro* and in *N. benthamiana*. *Cp*MAT indeed showed modest acetyltransferase activity *in vitro*, but not in *N. benthamiana*, not even when

acetyl-CoA was exogenously infiltrated along with corynantheol (**11**). We have added this information in the main text and included the data in Supplementary Fig. 8d. However, since the cyclase *Cp*MCC does not turnover acetyl-corynantheol (**21**) at detectable levels (see Supplementary Fig. 16), and given the fact that acetyl-corynantheol (**21**) is not detected in *Cinchona* plants (not even in VIGS assays), in contrast to malonyl-corynantheol (**14**), we believe that **21** is not involved in the pathway, and the acetylation observed *in vitro* is a promiscuous transferase activity, as seen with some other malonyltransferases (see *e.g.* Benke et al, *Nature* **2022**, *607*, 617-622 and Moghe et al, *Annual Review Plant Biology* **2023**, *74*, 165-194). In *Cp*MCC VIGS assays, the accumulation of malonyl-corynantheol (**14**) and dihydro and methoxylated congeners, which are usually not detectable in natural plants, strongly indicates that *Cp*MCC acts on these molecules. Nevertheless, their accumulation is not directly visible in the TIC, but only in EIC. We have not included the TICs in this figure, since no particularly striking difference is visible from TICs, making the TIC uninformative in this instance (see as example the TICs attached below). VIGS assays do not abolish, but only reduce gene expression, and therefore we are hesitant to overinterpret the exact levels of metabolites in these silencing experiments. We have also added data on the VIGS *CpMCC* expression (see Supplementary Fig. 16a). We hope all these additional data, along with the edits in the main text, and explanation now convincingly clarify the enzymatic activity of *Cp*MAT. (Please note that we plan to publish a separate report on the enzymology of *Cp*MCC, but we feel that such mechanistic work is well beyond the scope of this already data-heavy manuscript.)

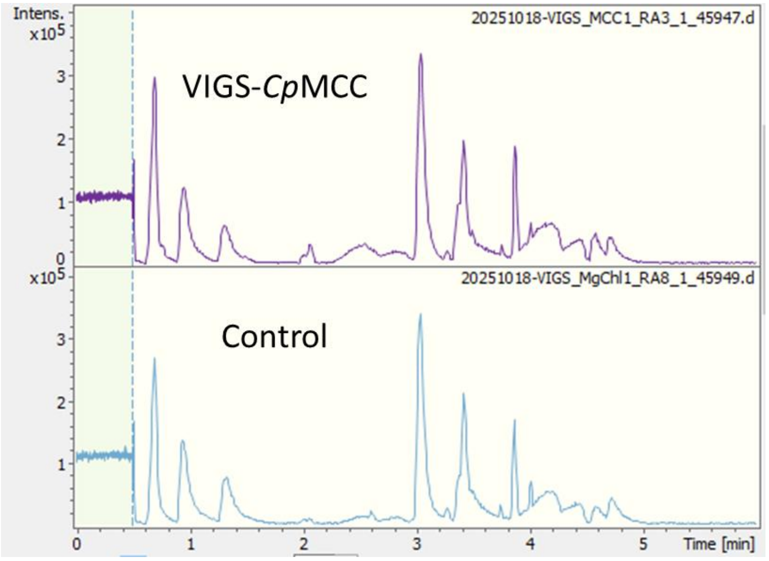

2. Fig 3f-g: it is interesting that N. benthamiana extract inhibits CpKR4 activity, which unfortunately prevents the production of the final products, dihydrocinchonidine and dihydroquinine, through heterologous pathway reconstruction in N. benthamiana. Does it inhibit the reaction or protein stability (and degrade CpKR4)? If it affects protein stability, adding a tag (e.g., YFP) could help stabilize the CpKR4 protein. Does the tissue extract of Cinchona or other plants (e.g., Arabidopsis) lack this inhibitory effect? These information will guide future metabolic engineering strategies, including plant chassis selection, for Cinchona alkaloid production.

Response. The finding on *Cp*KR4 activity is indeed interesting. The reviewer raises a good suggestion to distinguish between inhibition and protein degradation. To investigate the possibility of protein degradation by *N. benthamiana*, we have expressed His-tagged *Cp*KR4 in *N. benthamiana* and purified it. SDS-PAGE analysis shows that the protein (apparently) did not decompose. We also incubated purified recombinant *Cp*KR4 protein from *E. coli* with *N. benthamiana* extract for 6 or 24 hours and virtually no decomposition could be detected. We have added the SDS-PAGE gels to Supplementary Figure 30. Therefore, we think *N. benthamiana* extract actually inhibits the reaction.

Due to the fact that the *Cinchona* tissue extracts contain substantial quantities of the substrates (keto quinolines like cinchonidininone) and the products (final alkaloids like cinchonidine), the inhibitory effect of *Cinchona* extracts on *Cp*KR4 can only be reliably investigated using isotopically labeled keto quinoline substrates, which are unfortunately not available. This is why we have not examined whether *Cinchona* extracts inhibit *Cp*KR4.

We are aware that the *Cp*KR4 stability could be investigated through other methods, like thermal shift assays. Overall, *Cp*KR4 warrants a detailed mechanistic investigation, but, given the already large amount of data presented in this study, we would request that these investigations be left to a future study. Additionally, we also can not rule out the possibility that there is another more efficient reductase to be found. To highlight this possibility, we had included the following statement in the main text: “Although this reductase shows clear activity with the quinolines, it is possible that a reductase with higher stereoselectivity remains to be identified.”

3. While I acknowledge that a huge amount of data is already presented, some key information were still missing. In Page 11, how were the CpCiR candidates obtained for the screening of alcohol dehydrogenase that convers cinchonaminal to cinchonamine? In page 13: what were the 60 reductase candidates that were tested for the NADPH-dependent oxidoreductase, which resulted in the discovery of CpKR4? Seven additional CpKR candidates are also strongly expressed in roots and/or stem (Supp Fig. 31); were they tested for activity in vitro? Please provide these descriptions, so that readers know what exactly have been tested in this study.

Response. We appreciate the reviewer pointing out these ambiguities. We obtained the *Cp*CiR candidates by co-expression analysis. We have added this information in the main text and we have modified Supplementary Figure 21 by including a graph of candidate genes ranking based on co-expression with *Cp*CiS. We have provided the 60 tested putative reductases as an associated dataset. The seven additional *Cp*KRs that the reviewer mentions (specifically *Cp*KR1-8) were also tested, and this is now noted in the main text.

4. Fig 4c: It is exciting that a series of halogenated tryptamine substrates can be incorporated into the corresponding dihydrocinchoninone and dihydrocinchonidinone analogs. It will be helpful if the graph of Fig 4c includes the results of Fig 4a with the natural tryptamine substrate, so that relatively efficiency can be compared and discussed.

Response. We agree and we have modified Figure 4c to include data of Cinchona native tryptamine substrates (**3** and **3a**). Kindly note that for a better comparison, the new included data are from assays using tryptamine and 5-methoxytryptamine separately, but not from experiments in Figure 4a, as suggested. This is because the enzyme cascades differ in the two experiments: For experiments in Figure 4a, tryptamine-hydroxylase (*Cp*T5H) and *O*-methyltransferase (*Cp*OMT1) were included to generate *in situ* 5-OMe tryptamine (**3a**) from tryptamine (**3**), so that both **3** and **3a** convert in parallel into downstream methoxylated and non-methoxylated products, mimicking the natural process as in Cinchona plants. In Figure 4c, however, experiments are separate precursor-directed biosynthesis.

*5. The conclusion of the main text should discuss remaining knowledge and technical gaps, such as inactivity of CpKR4 in N. benthamiana and the missing enzyme responsible for the formation of dehydro congeners, corynantheal (**6**) and methoxylated analog (**6a**).*

Response. We agree that it would be ideal discussing the remaining knowledge and gaps. Unfortunately, due to manuscript length constraints, we are limited with the addition of more text. However, we have made an effort to note the knowledge gaps throughout the manuscript. The *in planta* inactivity of *Cp*KR4 had been explicitly noted, and we have now added in the conclusion the following statement to indicate that further investigations are still necessary: "Although aspects of the biosynthesis, such as the mechanism underlying the formation of the C18-C19 double bond, still remain to be resolved,.."

Minor comments

6. Page 2: Cite Supplemental Figure 1 in addition to Fig. 1.

Response. Thanks. The modification has been made. We have also called out Supplementary Fig. 2 in the same paragraph.

*7. Page 4: The structural determination of the novel intermediate cinchonium (**12**) is crucial data (Supplementary Fig. 46-51) supporting one of the major findings of the study. However, they are difficult to find them. I would be best if these data are directly cited in the main text (e.g., line 9-11 of page 4).*

Response. We agree that cinchonium (**12**) is a major finding of the study. However, as the numbering of figures must be chronological, following their appearance or citation in the text, calling out directly those NMR data figures on page 4 will lead to a new numbering for most of the Supplementary Figures. To avoid this but to still make these data relatively easy to access, we have added a table of contents and we also cite these data figures in the Methods part (page 19), which would appear online with the main text of the article. Thus, *e.g.*, for **12**, we now mention the corresponding supporting Figure data on page 19 of the Supplementary info file. We hope the referee finds this to be a reasonable compromise.

8. Fig. 3f: Please provide the list of 20 candidates as an associated table. (Also, the main text in page 9 is citing Fig. 3e, instead of 3f).

Response. We have provided the candidates list, as suggested, as a data source file for Figure 3. In the main text, we have corrected this mistake on the figure call-out. Thank you for spotting it.

9. Table S3: It will be best to provide in a fasta format with more detailed gene names, so that people can easily copy the entire table to carry out various analyses.

Response. We have added > to the sequences in Table S3 to make them FASTA format. We have also provided the list as a separate file in FASTA format as suggested. We can include just one or both in the final manuscript. Moreover, all these genes have been deposited in GenBank, accession numbers are pending and will be added to the Supplementary file before final publication. When these numbers are available, we will append them to the fasta formatted sequences in Table S3.

10. Supplementary Fig. 8: Please cite figure numbers of the underlying NMR spectra in the figure legend.

Response. We have cited the underlying NMR spectra in the figure caption.

*11. Fig 4b: Malonyl-corynantheol (**14**) was detected while the main text says otherwise (page 16, bottom). Please clarify.*

Response. We believe the referee refers to the following sentence: "In contrast, malonyl-corynantheol (**14**) was undetectable, consistent with the absence of this transient intermediate in native *C. pubescens* metabolite profiles (Fig. 4b)." This statement should be understood in the context of the preceding sentences, where we refer to the case of when *Cp*MAT downstream enzymes are added in the pathway reconstruction assays, leading to malonyl-corynantheal being completely consumed, which corroborates with the fact that in *Cinchona* plants this compound is also not detected.

However, since the sentence appears ambiguous, we have modified it as follows: "In contrast, malonyl-corynantheol (**14**) was undetectable when enzymes downstream of *Cp*MAT (*Cp*MCC, *Cp*CiS and *Cp*CiO) were present, consistent with the absence of this transient intermediate in native *C. pubescens* metabolite profiles (Fig. 4b)." We hope these modifications bring now more clarity.

Other comments:

Please note we checked the manuscript for minor typos. We added gene expression data to the *CpMCC* VIGS (see Supplementary Fig. 16a).as mentioned above. We noted that we did not mention in the main text all genes shown in Figure 4; all are now mentioned in the main text.

Editors Comments:

STATISTICS:

To the best of our knowledge, all statistical analyses are sound and in line with Nature guidelines.

REPRODUCIBILITY:

A new Reporting Summary is provided.

LENGTH:

We hope that this manuscript is not overly lengthy.

TITLE:

The title meets the formatting requirements.

SUMMARY PARAGRAPH:

This is provided and is 200 words.

MAIN TEXT:

We believe that we have adhered to the main text guidelines.

REFERENCES:

We have 46 references.

FIGURE LEGENDS:

All figure legends are less than 300 words and we believe that they fulfill the requirements. They are listed after the main text references.

METHODS:

We have put the entire Methods section in the Supplementary Information, since it contains chemical structures and reaction scheme.

ETHICS STATEMENT:

NA

MAIN TEXT STATEMENTS:

We believe that all of these statements are included in the correct place.

DATA AND CODE AVAILABILITY STATEMENTS:

NA

DISPLAY ITEMS:

Main text figures have been provided as .ppt files, with chemical structures imbedded as editable in ChemDraw (after double clicking on the respective structure or scheme). The editable format (.cdr) of the genes dot plot in Figure 3 is separately provided.

FIGURE FORMATTING:

We believe that we have adhered to the figure formatting requirements.

IMAGE PRESENTATION:

We believe that we have adhered to the guidelines.

EXTENDED DATA:

Extended data figures are provided in .tif format.

SUPPLEMENTARY INFORMATION:

We believe that we have conformed to the SI formatting requirements. An SI Guide is now provided.

SOURCE DATA (GRAPHS):

We have provided source data for Figures 3 and 4, Extended Data Figures 1, 3 and 4, Supplementary Figures 4, 11, 16, 18, 22 and 31.

RAW DATA (GELS):

Photos of unmodified gels have been added to this revised version. However, these gels are part of Supplementary Figure 30, not Figure 1. Given that these gels are not explicitly mentioned in the main text, nor do they form any part of a main text or extended data figure, we did not think that we should put these in Supplementary Figure 1. However, if this is incorrect, please let us know.

THIRD PARTY RIGHTS:

This has been added for the use of Biorender.

025-10-26998B

No response required
